# A Versatile DNA‐Encoded Library Platform for the Discovery of Highly Cooperative Chemical Inducers of Proximity

**DOI:** 10.1002/advs.77819

**Published:** 2026-09-27

**Authors:** Bingqi Tong, Sunny A. Tang, Yifan Deng, Zhihan Nan, Alison X. Gao, Gregory A. Michaud, Simone Bonazzi, Frédéric Berst, Frédéric J. Zécri, Shuang Liu, William J. Gibson, Stuart L. Schreiber

**Affiliations:** ^1^ Global Discovery Chemistry Novartis Biomedical Research Cambridge Massachusetts USA; ^2^ Chemical Biology and Therapeutics Science Broad Institute of MIT and Harvard Cambridge Massachusetts USA; ^3^ Department of Chemistry and Chemical Biology Harvard University Cambridge Massachusetts USA; ^4^ Department of Medical Oncology Dana‐Farber Cancer Institute Boston Massachusetts USA; ^5^ Discovery Sciences Novartis Biomedical Research Cambridge Massachusetts USA

**Keywords:** DNA‐encoded library, drug discovery, induced proximity, molecular glue, ternary complex cooperativity

## Abstract

Chemical‐induced proximity, particularly via molecular glues, represents a rapidly advancing therapeutic paradigm. Expanding the scope of proximity‐based therapeutics requires generalizable discovery platforms that can be easily tailored to diverse presenter proteins. Here, we report a versatile DNA‐encoded library (DEL) strategy designed to identify highly cooperative chemical inducers of proximity (CIPs) for multiple presenter proteins. By leveraging a novel precursor library constructed via on‐DNA strained allene cycloadditions, we generated three distinct CIP‐DELs biased toward Cereblon (CRBN), VHL, and FKBP12. Screening the CRBN‐focused library with BRD9 identified **B67b**, a novel compound that mediates ternary complex formation between CRBN and BRD9 with nanomolar potency and strong molecular glue‐like cooperativity (α >300). **B67b** engages BRD9 in a CRBN‐dependent manner, a characteristic of classical molecular glues, and its high cooperativity is driven by the synergistic contribution of all its structural and stereochemical components. This work establishes a scalable, generalizable framework for repurposing generic DELs into presenter‐specific screening tools, offering a powerful approach to accelerate the discovery of CIP therapeutics for a broad range of targets.

## Graphical abstract

We present a versatile DNA‐encoded library (DEL) platform designed for rapid library synthesis and subsequent screening for chemical inducers of proximity (CIPs) across various presenter proteins of choice. Applying this approach, we discovered a novel molecular glue‐like compound mediating CRBN‐BRD9 ternary complex formation with remarkable cooperativity (α >300).

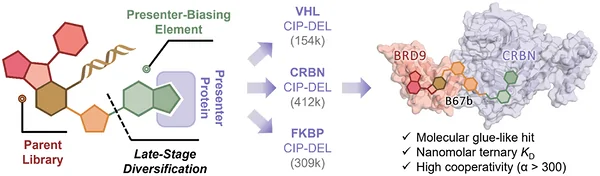

## 1 Introduction

Chemical‐induced proximity has emerged as a transformative strategy in modern therapeutics, enabling precise modulation of cellular processes through the deliberate induction of spatial closeness between specific proteins [1, 2, 3, 4]. Although many of these efforts have centered on targeted protein degradation (TPD) [5, 6, 7, 8, 9], the scope of proximity‐based approaches extends far beyond degradation to include among others immunomodulation [10, 11], targeted protein phosphorylation [12, 13], subcellular relocalization [14, 15], transcriptional rewiring [16, 17], tissue‐ or cell type‐specific targeting [18, 19], and chemical tools for probing cellular pathways [20]. These therapeutic strategies all rely on the strategic selection of appropriate “presenter proteins” (such as E3 ligases in the context of TPD), which are recruited to elicit desired biological responses [21]. Given the expanding landscape of induced proximity applications, it is imperative to develop a generalizable high‐throughput platform capable of screening chemical inducers of proximity (CIPs) that can be tailored to a wide range of presenter proteins for various therapeutic objectives.

Efforts to identify proximity‐inducing small molecules often focus on simple bifunctional compounds such as PROTACs, which benefit from a modular design. However, such compounds often suffer from suboptimal physicochemical and pharmacokinetic properties, complicating their clinical development [22]. Moreover, the need to optimize simultaneously both protein‐binding moieties and the connecting linker adds substantial complexity to medicinal chemistry campaigns [23].

In contrast, molecular glues, typically monovalent small molecules, present a compelling alternative with improved drug‐like properties. More importantly, by stabilizing protein‐protein interactions (PPIs) in a highly cooperative manner, molecular glues offer the potential for tissue‐selective targeting—owing to their presenter protein‐dependent activity—and engagement of proteins lacking well‐defined pockets that have traditionally been considered undruggable [1, 24, 25, 26]. These features make them particularly attractive for proximity‐based therapeutic modalities. Despite these advantages, molecular‐glue discovery has largely been serendipitous—often arising from mechanism‐of‐action studies of natural products or phenotypical screening hits—and rational design or screening has been a challenge. While focused chemical libraries have recently been used to identify molecular glues, the time and cost associated with library curation and screening are high [27, 28, 29, 30, 31].

**FIGURE 1:**
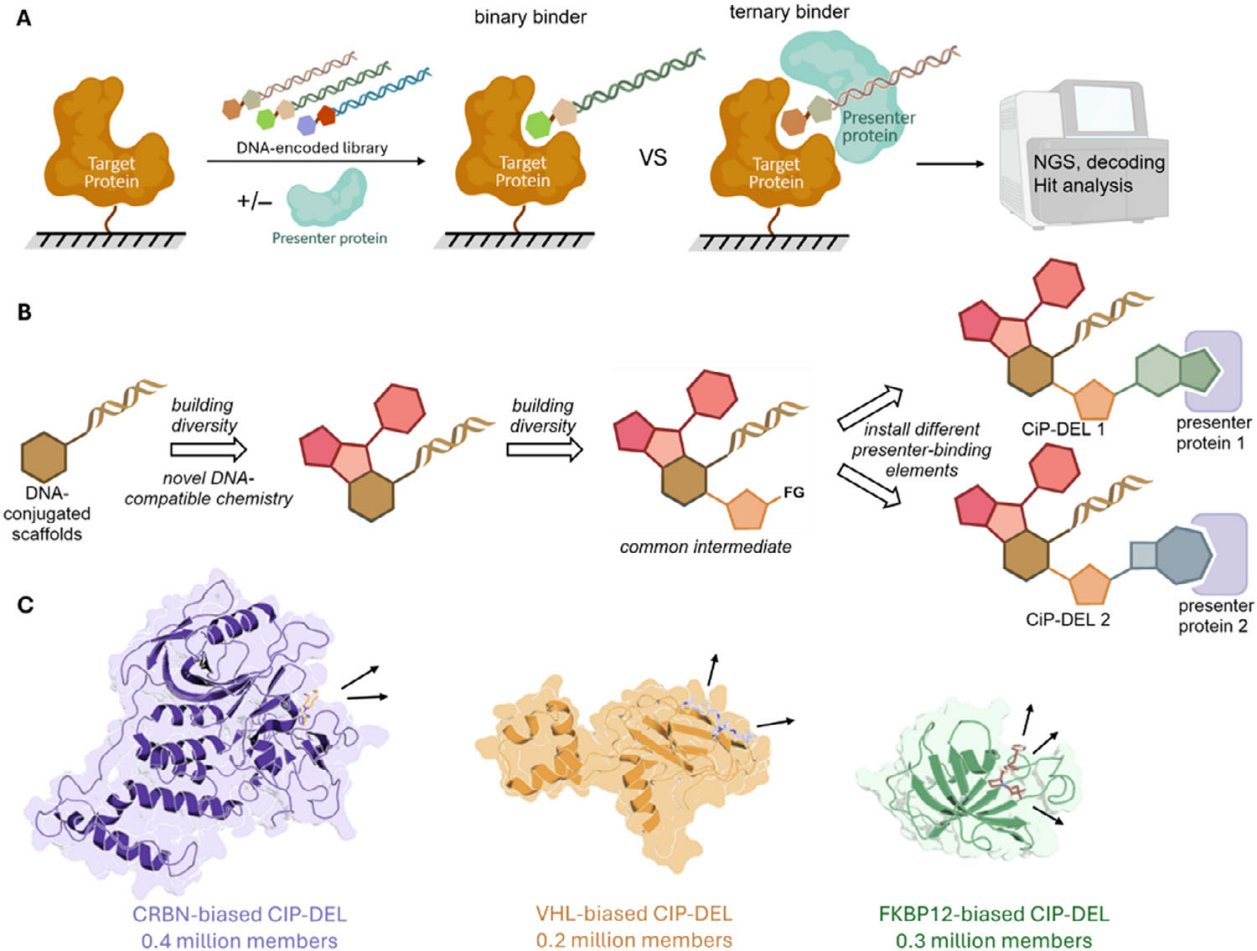
(a) CIP‐DEL screening workflow for identifying chemical inducers of proximity. The target protein is immobilized on magnetic beads and incubated with the library in the presence or absence of a presenter protein. (b) A versatile CIP‐DEL design that uses late‐stage diversification of a common DNA‐encoded library intermediate with various presenter‐binding ligands to generate multiple CIP‐DELs for diverse applications. (c) Three CIP‐DELs synthesized using CRBN‐, VHL‐, and FKBP12‐binding elements. Black arrows indicate the approximate exit vectors explored in these CIP‐DELs.

DNA‐encoded libraries (DELs) offer a unique opportunity for CIP discovery due to their combinatorial nature and ability to interrogate large areas of chemical space. The concept of utilizing DELs to discover compounds that induce productive biomolecular interactions has been explored in recent years, yet most of the efforts have followed a binder‐first strategy, in which DEL selections identify a binary ligand for a target or presenter protein that is subsequently elaborated into a bifunctional CIP. For example, this includes the conversion of DEL‐derived binders into PROTACs [32], or the identification of small molecules that bind monomeric RNase L and promote its dimerization and activation, which were then incorporated into RiboTACs for targeted RNA degradation [33, 34]. Complementing these approaches, our efforts have centered on directly identifying CIPs from DEL screens, with a focus on discovering molecular glue‐like compounds. Recognizing high cooperativity (α)—rather than molecular shape—as the defining feature of molecular glues, we previously developed a cooperativity‐focused DEL screening paradigm to identify such compounds (Figure 1a) [21, 35, 36]. Our initial chemical inducer of proximity DELs (CIP‐DELs), built on a VHL ligand scaffold, successfully yielded compounds with glue‐like behavior. Subsequent studies by our group and others extended this DEL‐based strategy to additional presenter and target proteins [37, 38, 39, 40, 41]. However, despite these advances, those libraries were typically focused on a single presenter protein, with designs built around only one of its ligands, limiting their broader versatility.

Here we report a broadly adaptable CIP‐DEL architecture that enables facile reconfiguration for multiple presenter proteins. By incorporating presenter‐biasing elements at the final stage of library synthesis, multiple CIP‐DELs can be derived from a generic precursor DEL with an appropriate functional group handle. It also allows encoding multiple ligands for the same presenter protein, increasing the likelihood of identifying optimal combinations of target‐ and presenter‐binding motifs. To demonstrate the synthetic modularity of this approach, we synthesized three distinct CIP‐DELs, collectively encompassing nearly one million members, each biased toward a different presenter protein: cereblon (CRBN), von Hippel‐Lindau (VHL), and FK506‐binding protein 12 (FKBP12) (Figure 1c). Notably, screening the CRBN‐biased CIP‐DEL yielded a molecular glue‐like compound **B67b**, which mediates the interaction between CRBN and BRD9 with high cooperativity. **B67b** exhibited CRBN‐dependent binding of BRD9 in the nanomolar range in biophysical assays and low micromolar potency in cellular assays, highlighting the potential of this approach as a versatile platform for discovering novel induced proximity‐based therapeutic agents.

## 2 Results and Discussion

### 2.1 Library Design

Our earlier VHL‐based CIP‐DELs were constructed from known VHL ligands, with DNA barcodes attached to one exit vector and library building blocks introduced through another [21]. This approach ensured that the DNA barcodes did not interfere with presenter protein binding but required two available exit vectors. A further limitation was that a new library had to be built from scratch whenever switching to a different presenter protein ligand.

To address these constraints, we redesigned the CIP‐DEL architecture by relocating the DNA barcode attachment site to the diversity‐generating region, such that the presenter protein ligands become the final‐cycle building blocks appended to the library (Figure 1b) [42]. This modification allows conversion of any generic DEL design into a CIP‐DEL, with the only requirement being the presence of a functional group handle for derivatization.

**FIGURE 2:**
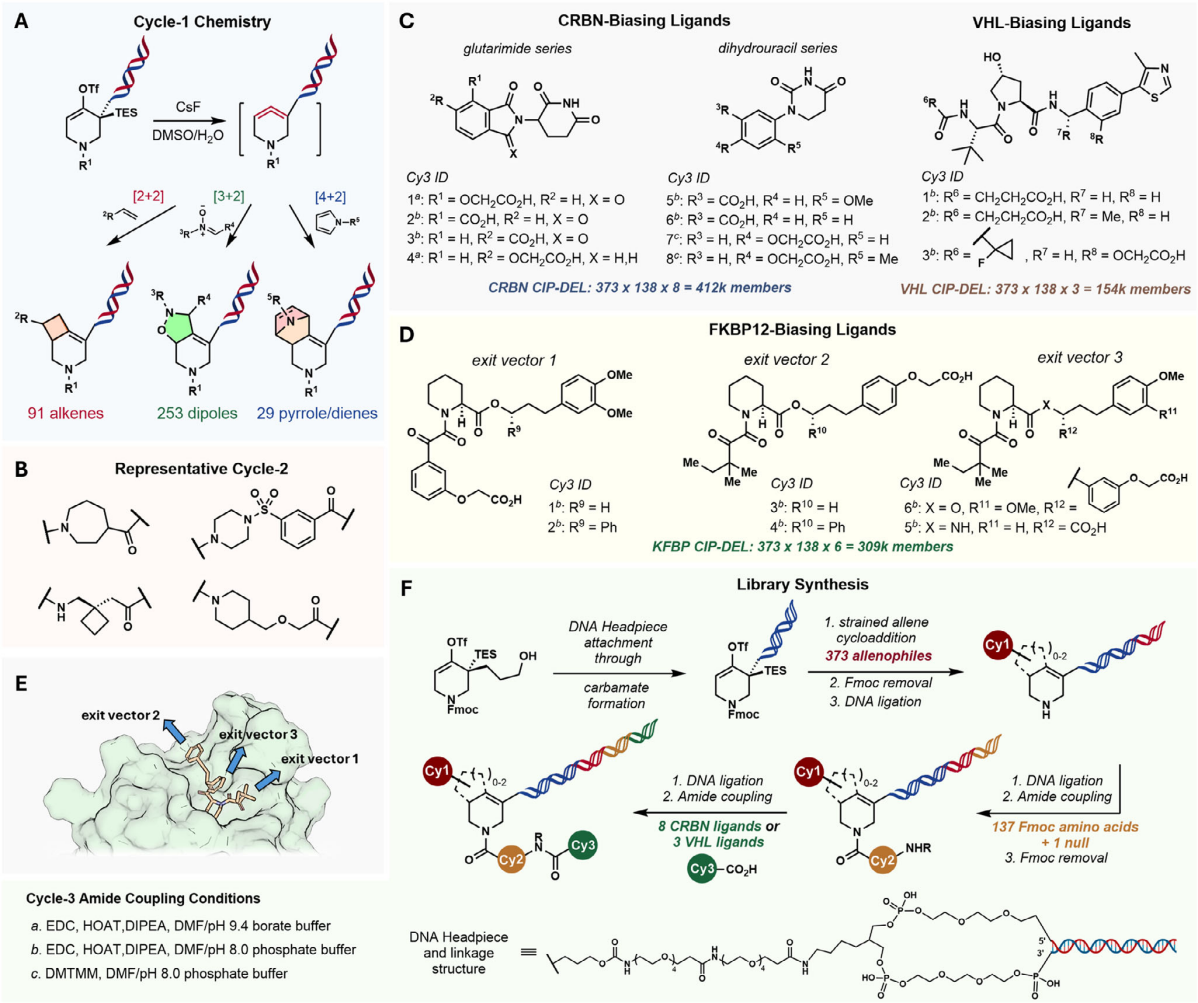
Syntheses of CRBN, VHL, and FKBP‐CIP DELs. (a) DNA‐compatible strained allene cycloaddition reactions provided diverse, novel, sp^3^‐rich compounds covalently attached to DNA barcodes. (b) Representative cycle‐2 structures derived from a diverse set of Fmoc‐amino acid building blocks. (c) CRBN and VHL ligands (cycle‐3 building blocks) used in CIP‐DEL syntheses. (d) FKBP12 ligands used in CIP‐DEL syntheses. (e) Illustration of the three exit vectors selected for the FKBP‐CIP DEL based on the X‐ray structure of FKBP12 in complex with “Holt ligand” (PDB ID: 1FKG). (f) General synthetic scheme of the library construction. Superscripts in (c),(d) indicate the amide coupling condition used for each ligand.

While this approach could be implemented with any standard DEL chemistries, we applied our previously developed on‐DNA strained allene cycloaddition to construct the parent library [43]. This reaction generates a diverse set of fused cyclic structures with high sp^3^ content, providing a marked contrast to the typically planar architectures of conventional DEL compounds (Figure 2a). For cycle‐2, we incorporated a broad set of structurally diverse, predominantly rigid Fmoc‐protected amino acids, in contrast to earlier CIP‐DEL designs, which used only a small set of structurally simple, “linker‐like” building blocks (Figure 2b). These moieties were expected to serve not only as connectors but also as potential contributors to binding. Subsequent Fmoc deprotection revealed the amino group needed for late‐stage presenter‐biasing ligand installation.

We selected cereblon (CRBN) as one of the primary presenter proteins for this proof‐of‐concept, as it is among the most extensively used E3 ligases in TPD. Recently, substantial effort has been directed toward developing CRBN binders beyond classical glutarimide‐based IMiDs, aiming to improve binding affinity, modulate physicochemical properties, reduce off‐target activities, and circumvent intellectual property constraints [44, 45]. Importantly, different CRBN ligands can bind in subtly different poses, thereby altering the protein surface and its protein–protein interaction potential. Inclusion of these differing ligands may therefore aid in the discovery of molecular glues. Our modular approach enabled the incorporation and screening of multiple CRBN ligands within a single library. To this end, we selected four glutarimide‐based ligands and four dihydrouracil‐based ligands, with varied modifications and exit vectors (Figure 2c) [46, 47, 48, 49, 50]. In parallel, we also designed a VHL‐focused library by selecting three different VHL ligands for library derivatization, encompassing both exit vectors used in our two previously reported VHL CIP‐DELs (Figure 2c) [21, 35].

To demonstrate the versatility of our CIP‐DEL platform beyond the protein degradation space, we designed an additional library biased toward FKBP12, a well‐characterized and privileged presenter protein for molecular glue discovery, owing to its high cellular abundance, good ligandability, and structural plasticity in forming diverse PPIs [24]. Leveraging three exit vectors revealed in the X‐ray structure of FKBP12 in complex with “Holt ligand”, we synthesized six FKBP12 ligands to systematically explore the scaffold's exit vector space (Figure 2d,e) [51]. The use of multiple exit vectors distinguishes this library from the previously reported FKBP CIP‐DEL and the rapafucin library, allowing it to sample a broader chemical space despite its relatively modest size (Figure S1) [27, 38].

Together, these CRBN, VHL, and FKBP12‐focused libraries demonstrate the flexibility of our late‐stage presenter protein‐biasing strategy and underscore its potential for broader applications.

### 2.2 Synthesis of CRBN, VHL, and FKBP12‐Focused CIP‐DELs

The scaffold containing the strained allene precursor, synthesized in seven steps from commercially available materials, was conjugated to the DNA headpiece via a carbamate linkage (Figure 2f). We then profiled the on‐DNA strained allene cycloaddition with over 600 “allenophiles”, of which 91 alkenes, 29 pyrroles/dienes, and 253 1,3‐dipoles achieved >70% yields, as determined by analytical LCMS. DNA conjugates from successful building blocks were scaled up, ligated with DNA barcodes, and pooled for the subsequent cycle‐2 reactions, in which 137 Fmoc‐amino acids (plus one “null” building block) were incorporated, followed by Fmoc deprotection (Figure 2f).

For cycle‐3 diversification, CRBN, VHL, or FKBP12 ligands were incorporated in high yield with EDC/HOAT‐ or DMTMM‐mediated amide coupling conditions, using either pH 8.0 phosphate buffer or pH 9.4 borate buffer (Figure 2c,d,f) [52]. Notably, the glutarimide‐based ligands 2 and 3 underwent partial hydrolysis in the more basic borate buffer, whereas dihydrouracil‐based ligands remained intact, demonstrating their superior stability [53, 54].

The final CRBN, VHL, and FKBP12‐focused CIP‐DEL comprised approximately 412, 154, and 309k members, respectively. All libraries were purified by gel electrophoresis prior to screening. Complete details regarding building block structures, validation yields, and associated DNA barcode sequences are provided in the Supporting Information (Tables S1–S6).

### 2.3 Screening of CRBN and VHL CIP‐DEL Against BRD9

BRD9 was selected as the proof‐of‐concept screening target owing to its therapeutic relevance in oncology and our prior success in identifying CIPs for this protein, making it an ideal benchmark [35, 55, 56]. His‐tagged BRD9 was immobilized on cobalt‐coated magnetic beads and incubated with either the library alone (binary condition) or with the library in the presence of 2 µM presenter protein (CRBN‐DDB1 complex or VHL‐Elongin C‐Elongin B complex, respectively; ternary condition). After washing and elution, recovered DNA barcodes were PCR‐amplified and sequenced to obtain read counts for each library member.

**FIGURE 3:**
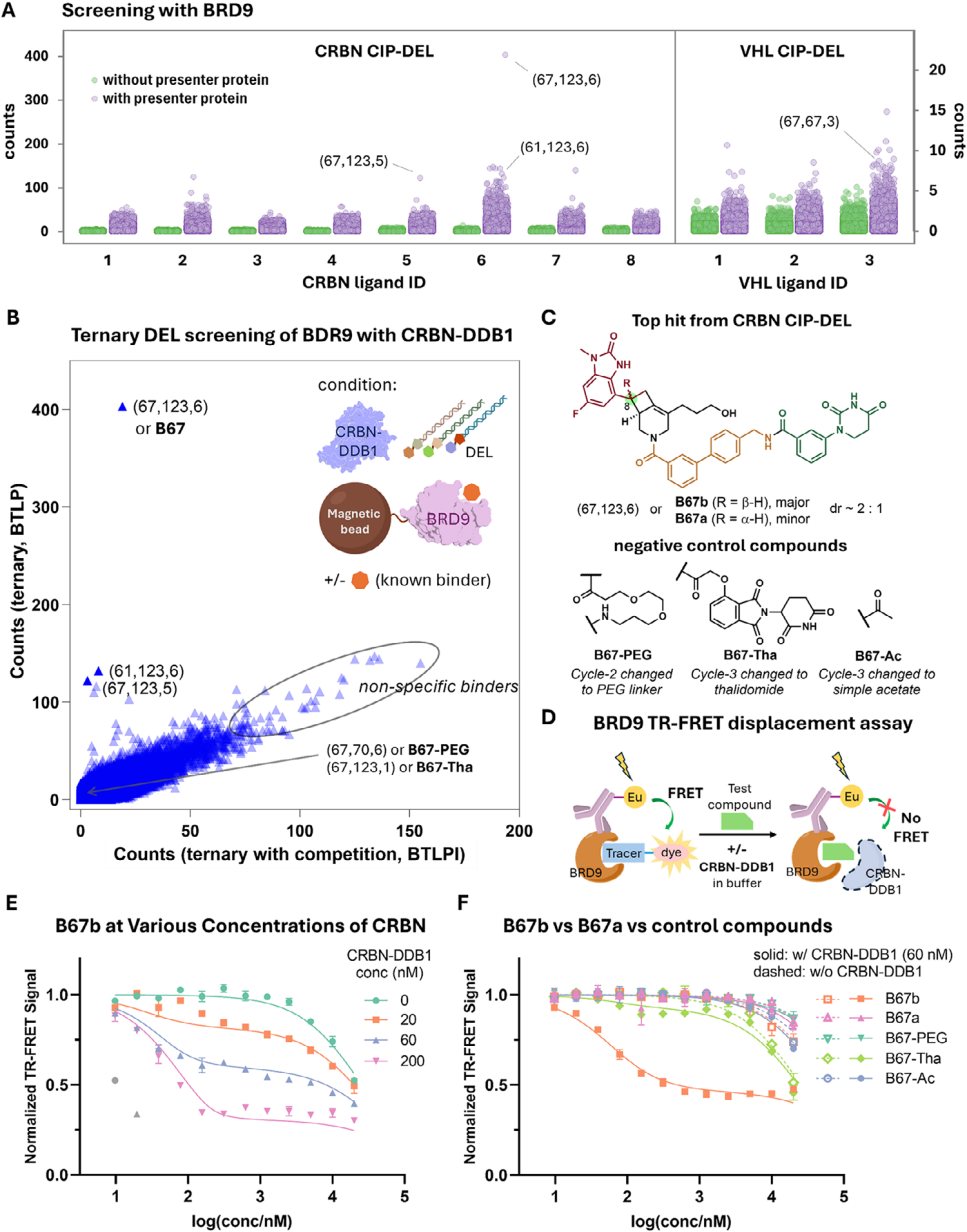
(a) Sequencing‐count distributions from CRBN‐ and VHL‐CIP‐DEL screens using BRD9 under binary and ternary screening conditions. Data points are jittered for visualization of the density. (b) Comparison of sequencing counts in the presence (*x*‐axis) or absence (*y*‐axis) of a known BRD9 binder (10 µM) under ternary screening conditions. True pocket binders (along the *y*‐axis) and non‐specific binders (along the diagonal) are highlighted. B, beads; T, target (BRD9); L, library; P, presenter protein (CRBN‐DDB1); I, known binder (I‐BRD9). (c) Chemical structures of the top positive and related control compounds. The stereocenter at C‐8 is highlighted. (d) Schematic of a TR‐FRET‐based BRD9 binding assay. (e) Dose–response curves of **B67b** in the TR‐FRET BRD9‐binding assay with varying concentrations of CRBN‐DDB1 (0, 20, 60, 200 nM). Curves fitted using equations described in the Supporting Information. (f) Dose–response curves of **B67a**, **B67b,** and other control compounds in the TR‐FRET BRD9‐binding assay in the presence (60 nM) or absence of CRBN‐DDB1. Data in (e) and (f) are normalized to the DMSO control and presented as mean ± SD (n = 2 technical replicates). Consistent results from independent replicates are shown in Figure S5.

In the CRBN CIP‐DEL screen, compound (67,123,6), hereafter referred to as **B67**, emerged as the top hit under the ternary condition. Several additional compounds sharing the same cycle‐1 (ID = 67) or cycle‐2 (ID = 123) building blocks as **B67** also appeared among the hits with high counts (Figure 3a and Figure S2a,c,f). Of particular note, compound (67,123,5), differing from **B67** by only a single methoxy substituent on the CRBN ligand moiety (Figure 2c), was also highly enriched, whereas other members of the (67,123,x) series were not. This clear structure‐activity relationship suggested a higher likelihood of true binding activity. Notably, no significant hits were observed in the binary screening format, indicating the absence of potent binary binders of BRD9 in the CRBN‐focused library (Figure 3a and Figure S2a). For the VHL CIP‐DEL screen, although overall counts were lower, several members of the (67,x,x) series also ranked near the top by sequencing count, consistent with the expectation that this cycle‐1 building block engages in the BRD9 binding pocket (Figure 3a). Mono‐synthon analysis of cycle‐1 building blocks confirmed this hypothesis (Figure S3).

Notably, the sequencing counts for individual CRBN CIP‐DEL members under the ternary screening condition were generally much higher than under the binary screening conditions, presumably due to non‐specific interaction between CRBN‐DDB1 and the beads. To differentiate genuine binders from non‐specific binders, we performed the ternary screen in the presence of I‐BRD9, a known BRD9 inhibitor, as a competitor (Figure 3b). Indeed, when counts were compared in the presence versus absence of the competitor, most high‐count compounds were largely unaffected. However, several members of the (67,x,5/6) series, including the top hit **B67**, exhibited a substantial reduction in counts in the presence of the competitor, indicative of specific engagement of BRD9's canonical pocket (Figure 3b and Figure S2b). Consistently, this series of compounds was not enriched in the no‐target control condition (Figure S2d), nor in a parallel counter‐screen of a distinct bromodomain protein BRD4 (Figure S2e), effectively ruling out non‐specific binding. The minimal variation in conditions during this competition screen enables the direct use of raw sequencing counts, while an orthogonal evaluation using a Poisson distribution‐based enrichment metric produced an identical hit profile (Figure S2a) [57]. Based on these findings, **B67** was prioritized for off‐DNA synthesis and further validation.

### 2.4 Hit Resynthesis and Initial Validation

Off‐DNA synthesis of **B67** and related analogues followed the same sequence of reactions used in library construction. In place of the DNA barcodes and carbamate linkage, a free hydroxyl group was used, although the entire propanol moiety is likely dispensable. Notably, **B67** was present in the library as a mixture of stereoisomers with a 2:1 diastereomeric ratio (dr) at C8 (Figure 3c and Figure S4a), originating from the strained allene cycloaddition reaction. We were able to separate the diastereomers during off‐DNA synthesis, and their relative stereochemistry was assigned using 2D NMRs (Figure S4b–d).

For the initial binding validation, we used a TR‐FRET‐based BRD9 binding assay performed in the absence or presence of CRBN‐DDB1 at varying concentrations, mirroring the binary and ternary DEL screening formats. A Cy5‐labeled BRD9 ligand served as tracer, and displacement by test compounds resulted in a decreased TR‐FRET signal (Figure 3d). **B67b**, the major diastereomer, exhibited dose‐ and CRBN‐DDB1‐dependent binding to BRD9, with a significant leftward shift of the binding curve observable at CRBN‐DDB1 concentrations as low as 20 nM. At 200 nM CRBN‐DDB1, the apparent IC_50_ decreased by two orders of magnitude, from 20 µM to 0.2 µM (Figure 3e and Figure S5b). The observed biphasic binding profile was consistent with ternary complex formation dominating when compound concentration was below that of CRBN‐DDB1, whereas at higher compound concentrations, binary binding accounted for the additional signal change. In striking contrast, **B67a**, the minor diastereomer differing only at a single stereocenter, displayed virtually identical binding curve with and without CRBN‐DDB1(Figure 3f and Figure S5a), highlighting the importance of stereochemistry in molecular glue‐like activity.

To demonstrate further that all structural components of **B67b** are essential for achieving highly cooperative BRD9 binding, we selected three negative control compounds, **B67‐PEG**, **B67‐Tha**, and **B67‐Ac**, by substituting the cycle‐2 building block of **B67b** with a PEG linker, or cycle‐3 building block with a thalidomide‐based ligand or an acetyl group, respectively (Figure 3c and Figure S6a). Notably, **B67‐PEG** and **B67‐Tha** were present in the library but not enriched as screening hits (Figure 3b and Figure S6b). Consistent with this, although each control compound retained weak binary BRD9 binding in the TR‐FRET assay, none exhibited CRBN‐DDB1 dependent potentiation (Figure 3f and Figure S5a). Collectively, these findings indicate that the high cooperativity of **B67b** is not incidental but instead requires the synergistic contribution of all its structural components—a feature rarely achieved by conventional bifunctional designs.

### 2.5 Cooperativity (α) Determination of **B67b**

The shift in binding curves observed in the presence of CRBN‐DDB1 indicates highly cooperative ternary complex formation by **B67b**. To calculate the cooperativity (α), we derived mathematical equations that account for both binary and ternary equilibria in our TR‐FRET assay (see Supporting Information, Section 2 for details). Binary and ternary *K*_d_ for BRD9 were obtained by global non‐linear regression fitting across four CRBN‐DDB1 concentrations (0, 20, 60, and 200 nM; Figure 3e and Figure S5b). Fitting results show that **B67b** exhibited a binary *K*_d_ of >20 µM and a ternary *K*_d_ of ∼ 60 nM for BRD9 (Figure S5c), corresponding to a cooperativity factor of >300 — approaching the values characteristic of classical molecular glues [58].

These results also demonstrate that a modified binary TR‐FRET binding assay can serve as a practical method for quantifying the cooperativity of molecular glues. This approach circumvents the need for excessive protein concentrations to fully saturate small molecules or the reliance on more complex PPI‐based assays, as required in other recent studies [59, 60, 61, 62].

### 2.6 Biophysical and Cellular Validation of **B67b**‐Mediated Ternary Complex

**FIGURE 4:**
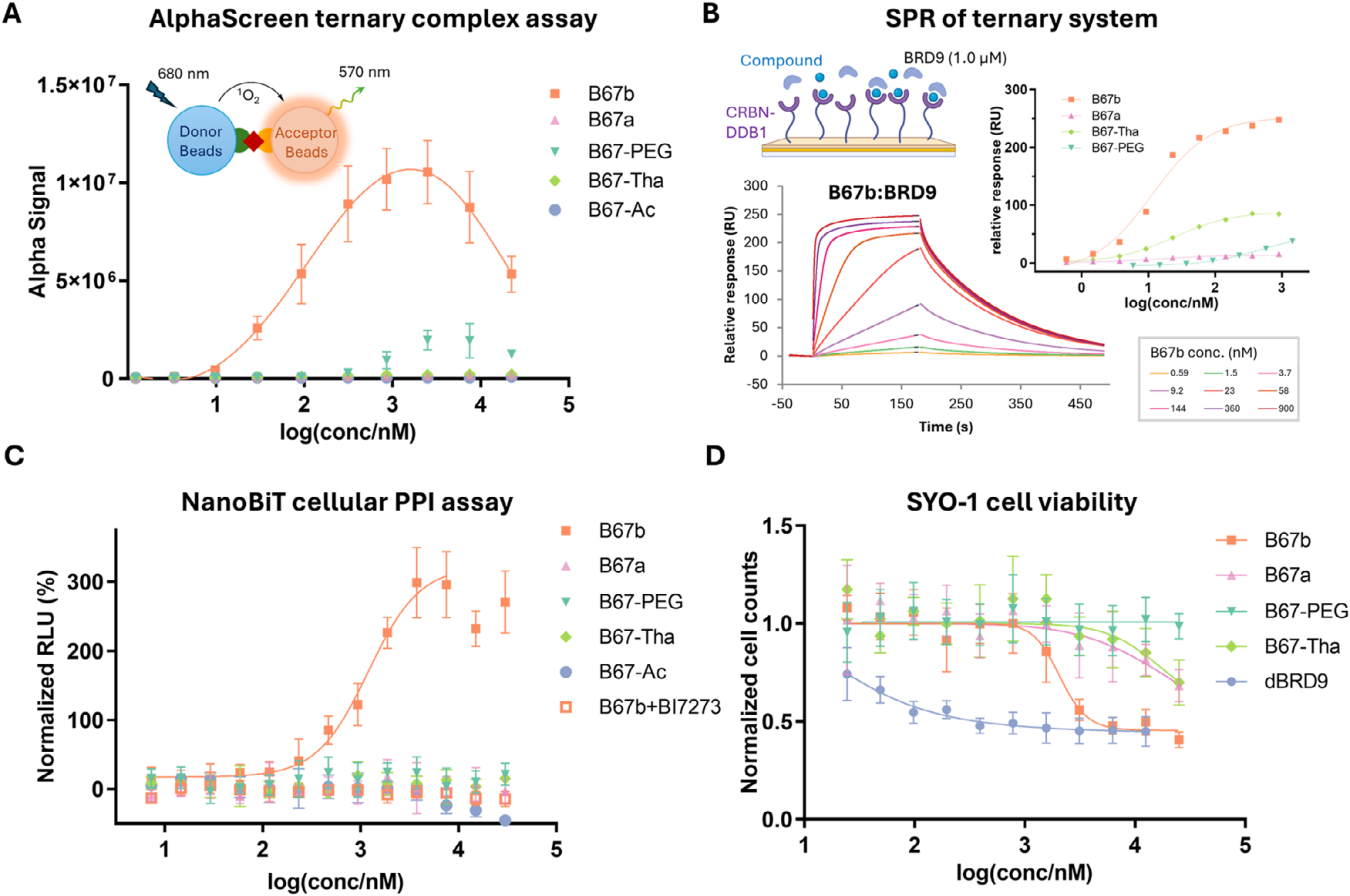
(a) AlphaScreen assay measuring compound‐induced ternary complex formation between His‐tagged CUL4A‐RBX1‐DDB1‐CRBN and biotinylated BRD9. Mean ± SD, n = 2 technical replicates. (b) (left) SPR sensorgram of **B67b** in the presence of 1.0 µM BRD9 flowing over CRBN‐DDB1 immobilized sensor chip surface; (right) dose‐dependent ternary complex formation of compounds on SPR sensor surface as indicated by relative response unit at the end of each injection. (c) Dose–response induction of ternary complex formation in HEK293T cells by **B67b** in the NanoBiT assay. Mean ± SD, n = 4 technical replicates. (d) Dose–response viability of SYO‐1 cells treated with **B67b** or control compounds for 7 days. Data normalized to DMSO control. Mean ± SD, n = 6 from two independent experiments.

We next assessed **B67b**‐induced ternary complex formation using an AlphaScreen assay with BRD9‐tagged donor beads and CRBN‐DDB1‐CUL4A‐RBX1‐tagged acceptor beads. Consistent with the TR‐FRET results, **B67b** generated a markedly higher luminescence signal than any other compound (Figure 4a). At the low BRD9 and CRBN complex concentrations used in the assay, the hook effect strongly limited the observed maximum luminescence. The higher peak signal produced by **B67b** reflects a greater ternary partition fraction driven by its high cooperativity, whereas other compounds, lacking strong binary binding potency and high cooperativity, experienced such pronounced hook effects that ternary complex formation was barely detectable. An alternative TR‐FRET‐based protein‐protein interaction assay provided similar results (Figure S7).

Next, we used surface plasmon resonance (SPR) to probe ternary complex formation. We first used the binary binding experiments to confirm that our compounds (except **B67‐Ac**) bind CRBN‐DDB1 with nanomolar potency, but displayed very weak or unmeasurable binding to BRD9 (Figure S8), consistent with TR‐FRET results. We then performed the ternary experiment by injecting compounds at varying concentrations, pre‐mixed with 1.0 µM BRD9, over the sensor surface immobilized with CRBN‐DDB1. Owing to the weak binary affinity ($K_{d}^{B}\gg c_{\mathrm{BRD}9}$), only a very small fraction of the compound was present as a binary complex with BRD9 in the sample solution. Despite that, **B67b** induced a strong response (RU ∼ 250) close to the theoretical value expected for ternary complex formation (immobilization RU = 4000, theoretical R_max_ = 300 for ternary complex, R_max_ = 17 for binary complex) at low nanomolar concentrations (apparent *K*_d_ = 11 nM), driven by the high cooperativity of **B67b** (Figure 4b). In contrast, other compounds showed markedly reduced ternary complex formation, as indicated by the lower RU value observed (Figure 4b and Figure S9).

We next evaluated **B67b**’s activity in cellular environments using NanoBiT assay in HEK293T cells co‐transfected with LgBiT‐CRBN, BRD9‐SmBiT, and DDB1. Only **B67b**, and not any control compound, produced a robust luminescence signal, with an EC_50_ of 1.1 µM (Figure 4c). Co‐treatment with the potent BRD9 inhibitor BI7273 abolished the signal, verifying that **B67b** engages BRD9 in cells at its canonical pocket.

As a key component of non‐canonical BAF chromatin remodeling complex, BRD9 underlies the growth dependency of several cancer types including synovial sarcoma, and has been pursued in clinical trials with bifunctional degraders [63, 64, 65]. In SYO‐1 synovial sarcoma cell line, we found that **B67b** inhibited cell proliferation with an IC_50_ of 2.1 µM and achieved the same maximal inhibition as the potent BRD9 degrader dBRD9 (Figure 4d). Furthermore, no anti‐proliferation effect was observed in the BRD9‐independent HCT116 cell line, supporting an on‐target mechanism (Figure S10).

### 2.7 Computational Modeling of Ternay Complex Structures

**FIGURE 5:**
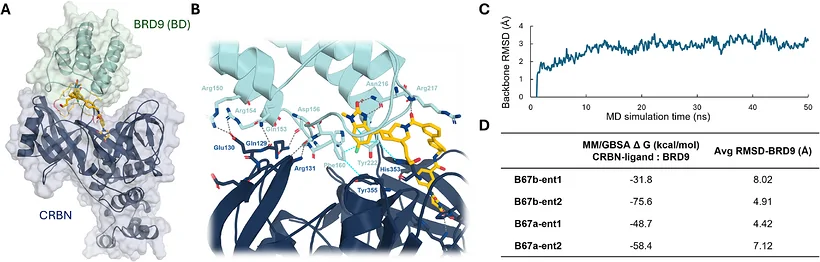
Computational modeling of the ternary complex. (a) Representative frame from the MD simulation of the CRBN‐**B67**‐ent2‐BRD9 (BD) ternary complex. (b) Close‐up view of the protein‐ligand and protein‐protein interfaces. Hydrogen bonds and salt bridges are depicted as grey dashed lines; π–π interactions are shown as cyan dashed lines. (c) Plot of the backbone root‐mean‐square deviation (RMSD) versus MD simulation time, relative to the initial structure. (d) Calculated binding free energy (Δ G) using the MM/GBSA method and average RMSD of BRD9 following alignment to CRBN.

To gain structural insight into the high cooperativity achieved by **B67b**, we computationally modeled the ternary complex for all four possible stereoisomers of **B67a/b** using a combination of Boltz‐2 (an open‐source AlphaFold3‐based structural prediction model) and molecular dynamics (MD) simulations [66, 67]. While AlphaFold3‐based models excel at predicting native protein‐protein interactions, they may not accurately capture the complex energetic landscape of the three‐body systems, particularly the conformational strain of the small molecule ligand [68]. Therefore, we used the Boltz‐2 prediction as the starting point for a 50 ns MD simulation to validate and refine the modeled structure (Figure 5 and Figure S13). Subsequent MM/GBSA free energy calculations were used to evaluate the stability of the ternary complex, revealing that one of the enantiomers of **B67b** (**B67b**‐ent2) yielded the lowest binding energy toward BRD9 (Figure 5d) [69].

The ternary complex reached a relatively stable conformation during the 50 ns MD simulation, as indicated by the backbone RMSD plot (Figure 5c). In a representative frame, both the dihydrouracil and 2,3‑dihydro‐2‑oxo‐benzimidazole moieties sit deeply within the canonical binding pockets of CRBN and BRD9, respectively. The BRD9 interaction is primarily stabilized by two hydrogen bonds with Asn216 and a π–π stacking interaction with Tyr222 (Figure 5a,b). Importantly, the predicted structure indicates that CRBN and BRD9 form an extensive protein‐protein interface mediated by loop regions from both proteins. This includes hydrogen‐bond and electrostatic interaction networks between Gln129, Glu130, and Arg131 of CRBN and Arg150, Gln153, Arg154, Asp156, and Gly159 of BRD9, alongside a dynamic π–π interaction network close to the ligand‐binding site (His353 and Tyr355 of CRBN; Phe160 and Tyr222 of BRD9).

Notably, the Gln129‐Asp156, Gln129‐Gln153, and Arg131‐Gly159 hydrogen bonds maintained high occupancy during the MD run for this specific **B67b** enantiomer, whereas they lacked significant occupancy for the other isomers, providing a structural rationale for the difference in potencies observed between **B67a** and **B67b** (Figure S13). Additionally, the free hydroxy group to which the DNA barcodes were attached is oriented toward the solvent‐exposed opening of the interface, consistent with the DEL platform constraints. While this computationally derived structural model may not perfectly replicate the actual ternary complex, it illustrates how stereochemical variations can have profound effects on the architecture and stability of a molecular‐glue mediated protein‐protein interface.

## 3 Discussion

We describe a versatile CIP‐discovery platform that enables rapid generation and screening of multiple CIP‑DELs tailored to different presenter proteins. Applying this approach, we identified **B67b**, a molecular glue‑like compound that promotes highly cooperative ternary complex formation between CRBN and BRD9. While recent studies have demonstrated the potential of DEL technologies for screening proximity‐inducing small molecules directly, these efforts have typically relied on bespoke libraries synthesized from scratch. Here we show that by leveraging the availability and maturity of traditional DELs in drug discovery, those generic library designs can be efficiently repurposed into CIP‐DELs that are capable of discovering high‑cooperativity, glue‑like compounds comparable to those obtained with prior approaches.

The term molecular glue has lacked a universally accepted definition despite its widespread usage in recent years [70, 71]. The rapid emergence of different forms of CIPs has promoted a distinction between bifunctional molecules and molecular glues based on molecular architecture (presence or absence of a linker). However, such classification overlooks the defining functional feature of molecular glues: the induction of favorable protein–protein interactions, quantifiable by cooperativity (α) [72, 73]. (Traditional linker‑free glues are, in a literal sense, also bifunctional, as they engage two proteins via distinct structural elements often joined by two linking elements that together comprise a macrocyclic ring). Our prior studies have shown that compounds with high cooperativity can emerge from bifunctional‐looking libraries, and together with the current work, this supports the broader applicability of the concept to diverse presenter proteins [35].

Although this concept has been proven feasible, achieving high cooperativity requires all molecular components to complement the protein–protein interface synergistically, necessitating the screening of large, diverse libraries to identify optimal fits. This is exemplified by the sharp structure–activity relationships (SARs) observed in our study, where a single stereochemical change from **B67b** to **B67a** completely abolishes cooperative ternary binding, despite both compounds exhibiting similarly weak binary BRD9 affinities. We infer that the 2,3‑dihydro‐2‑oxo‐benzimidazole moiety is the primary contributor to binary BRD9 binding, whereas stereochemistry within the scaffold strongly influences ternary cooperativity. These findings encourage the inclusion of sp^3^‑rich, stereochemically diverse scaffolds—such as those generated by strained allene cycloaddition—in library design, as they may better accommodate specific binding pockets or PPI geometries. Similar stereochemical diversity has proven valuable in chemoproteomics, where “stereoprobes” have repeatedly facilitated the discovery of ligandable proteins [74]. While incorporating stereoisomeric mixtures directly into the DEL efficiently harnesses this diversity, a natural result of this approach is that if an active stereoisomer forms as a minor product during on‐DNA synthesis, its sequencing signal may be diluted by the inactive major isomer. Because DEL screening is typically a hit‐discovery technology rather than a comprehensive survey of chemical space, false negatives arising from low synthetic representation are an accepted boundary of the assay. This underscores the necessity of careful off‐DNA hit synthesis and stereochemical separation to uncover the true activity of individual isomers. An alternative approach is to separate and encode individual stereoisomers during library construction, albeit at the cost of substantially increased synthetic effort [75, 76].

Our results also highlight the value of exploring diverse CRBN‑binding motifs in molecular glue design. Substituting **B67b**’s N‑aryl‑dihydrouracil CRBN recruiting moiety with a thalidomide‑based ligand abolished glue‑like activity. This contrasts with typical PROTAC design, where less emphasis is placed on E3‐recruiter diversity. Recent CRBN‑based glue studies have revealed an increasing diversity of CRBN‑binding chemotypes, yet the glutarimide headgroup remains predominant. The N‑aryl‑dihydrouracil motif offers several advantages over glutarimides, including superior stability and elimination of an epimerizable stereocenter, while also possessing the potential to achieve very high cooperativity in suitable systems [48].

It is noteworthy that, even without medicinal chemistry optimization, **B67b** already shares certain structural and physicochemical features with CFT8634, a BRD9 degrader evaluated in Phase I clinical trials (Figure S11) [65]. This is partly attributable to the incorporation of rigid cycle‑2 building blocks in our library and the cooperativity‑focused screening approach. As the BRD9‑binding moiety of **B67b** is novel and emerged directly from screening, there remains potential to enhance its binary affinity, which would further lower its nanomolar ternary *K*_d_, provided high cooperativity is preserved. Furthermore, because **B67b** was evaluated as a racemic mixture, the active enantiomer is expected to possess even greater ternary potency. Additionally, given that paralog selectivity between BRD9 and BRD7 is a well‐documented challenge in the field, evaluating and further optimizing the selectivity profile of these compounds will represent an important next step [77].

Despite potent ternary complex formation, **B67b** did not induce significant degradation of BRD9 in HEK293T cells (Figure S12). The lack of degradation may reflect suboptimal orientation of BRD9 within the ubiquitination complex, where an overly rigid ternary complex assembly could hinder ubiquitination of accessible lysines. This aligns with prior observations that the correlation between cooperativity and degradation rate is complex, underscoring the multifactorial nature of the degradation process [78, 79, 80, 81, 82]. Medicinal chemistry optimization has been shown to improve degradation profiles in similar cases [83]. In addition, while traditional DEL screening is inherently binding‐based, emerging functional DEL screening formats could be adapted for direct identification of degraders [40, 84, 85]. From a broader induced‑proximity perspective, however, the DEL‐based “binder‑first” strategy offers greater flexibility, as initial binders can be modified and fine‑tuned in function‑specific assays to modulate target activity through mechanisms beyond degradation [86]. In many cases, ternary complex formation alone can elicit a cellular effect, either through blocking enzymatic function or altering the interactome [87]. We speculate that this may underlie the anti‑proliferative effects of **B67b** in SYO‑1 cells, where ternary complex formation—without degradation—could still disrupt the incorporation of BRD9 into the non‐canonical BAF complex. We further conducted differential gene expression (DGE) analysis of **B67b**‐treated SYO‐1 cells using RNA‐seq (Figure S14). After 24 h of treatment, genes associated with E2F targets and the G2M checkpoint were downregulated, consistent with the observed growth inhibition. We also noted a moderate downregulation of the MYC signaling pathway, which is consistent with certain reported transcriptional consequences of BRD9 perturbation [88]. However, the concurrent upregulation of pathways related to the unfolded protein response and cholesterol homeostasis suggests the induction of ER and proteostatic stress. Together, these data indicate that while **B67b** elicits some BRD9‐associated changes, its overall cellular effects likely stem from complex, multifactorial mechanisms that warrant further investigation.

High cooperativity is central to one of the key promises of molecular glues: enabling the therapeutic targeting of “undruggable” proteins by potentiating otherwise weak binding interactions. Although BRD9 is a well‐ligandable protein, the limited size of our proof‑of‑concept library yielded no high‐potency binary ligand. While DEL screening routinely identifies low‐micromolar hits, detecting weaker binders—such as **B67b**—remains challenging. This difficulty stems both from the practical challenge of distinguishing weak signals from non‐specific background and from fundamental thermodynamic constraints of the affinity selection step [89]. These limitations in capturing weak binding events may be even more pronounced for less tractable targets. Nevertheless, our CIP‑DEL design and screening successfully identified ternary hits with strong enrichment despite weak binary affinities. We envision that with larger, industry‑scale libraries and diversification to additional presenter proteins, this strategy could be extended to a broader range of targets, including those within the “undruggable” proteome. Given the accessibility of million‑ to billion‑member industrial DELs, our generalizable CIP‑DEL framework could serve as a powerful platform for drug discovery.

While the discovery of **B67b** successfully validated the utility of our CIP‐DEL platform, future work will focus on demonstrating the broad applicability of this strategy across a wider array of presenter proteins. Although the VHL‐ and FKBP12‐based libraries described herein primarily serve to demonstrate the synthetic modularity of our approach, recent literature has already established that both VHL and FKBP12 are effective presenter proteins for CIP‐DEL screening [35, 39]. Building upon these precedents, we anticipate that our generalizable, late‐stage diversification strategy will significantly accelerate the expansion of molecular glue discovery to novel presenter proteins.

Although the presenter‐biased libraries developed in this study are designed to sample CRBN‐, VHL‐, and FKBP12‐associated chemical space, they naturally represent only a finite subset of possible ternary‐interface chemotypes. For example, while robust experimental validation was achieved for the CRBN–BRD9 pair, the VHL–BRD9 selections exhibited comparatively modest enrichment under the tested conditions. These findings highlight the multifaceted nature of molecular glue discovery and underscore the critical interplay of library design, substituent vectors, scaffold geometry, target surface compatibility, and screening conditions. Future efforts to expand the library size, scaffold diversity, and the target panel will further illuminate the broader potential of this approach across diverse presenter proteins.

Crucially, a key advantage of this modular platform is the ability to rapidly generate parallel libraries biased toward multiple distinct presenter proteins. This capability enables the screening of a single target with an array of presenter proteins, thereby maximizing the probability of discovering molecular glues for high‐value, often “intractable” targets. To complement this empirical approach, computational or machine learning tools might be employed in the future to rationally select the most suitable presenter proteins for those targets.

## 4 Conclusions

Induced proximity is becoming an increasingly important paradigm in chemical biology and therapeutic development, opening new avenues for precise and versatile modulation of protein functions. Yet, despite the existence of ∼600 E3 ligases, numerous molecular chaperones and transcriptional regulators in the human proteome, only a small fraction has been explored as presenter proteins [90, 91]. Our CIP‐DEL approach offers a scalable and generalizable means to expand this presenter protein repertoire. We also envision a two‑step workflow in which a traditional DEL is first used to identify binders for new presenter proteins, followed by construction of focused CIP‑DELs for screening optimal CIPs or molecular glues. Such an approach could accelerate the discovery of proximity‑inducing agents across diverse target classes, ultimately broadening the therapeutic reach of induced‑proximity strategies and enabling rapid adaptation to emerging biological insights.

## Author Contributions

**Bingqi Tong**: conceptualization, investigation, methodology, writing – original draft, writing – review and editing, visualization, validation, supervision. **Sunny A. Tang**: methodology, investigation, writing – review and editing. **Yifan Deng**: methodology, investigation, writing – review and editing. **Zhihan Nan**: methodology, investigation, writing – review and editing. **Alison X. Gao**: writing – review and editing, supervision, resources. **Gregory A. Michaud**: supervision, resources, writing – review and editing. **Simone Bonazzi**: supervision, resources, writing – review and editing. **Frédéric Berst**: writing – review and editing, supervision, resources. **Frédéric J. Zécri**: funding acquisition, resources, supervision, project administration, writing – review and editing. **Shuang Liu**: writing – review and editing, investigation, methodology, supervision, visualization. **William J. Gibson**: supervision, funding acquisition, writing – review and editing. **Stuart L. Schreiber**: conceptualization, methodology, supervision, project administration, funding acquisition, writing – review and editing, resources.

## Conflicts of Interest

The authors declare the following competing financial interest(s): S.L.S. is a shareholder and advises Magnet Biomedicine, Convergence Bio, and Exo Therapeutics; he advises Eisai Co., Ltd. W.J.G. is on the scientific advisory board and has received consulting fees from Esperion Therapeutics and consulting fees from Belharra Therapeutics, Boston Clinical Research Institute, Faze Medicines, ImmPACT‐Bio, and nference.

## Supporting information




**Supporting File**: advs77819‐sup‐0001‐SuppMat.pdf.

## Data Availability

The data that supports the findings of this study are available in the supplementary material of this article.
