# Supplementary material for: A Versatile DNA‐Encoded Library Platform for the Discovery of Highly Cooperative Chemical Inducers of Proximity
Source: Adv Sci (Weinh). 2026 Sep 27:e77819. Online ahead of print. doi: 10.1002/advs.77819 (PMC13616243; doi:10.1002/advs.77819)
Supplement: Supplementary file 1 [file ADVS-9999-e77819-s001.pdf]

Supporting Information

A Versatile DNA-Encoded Library Platform for the Discovery of Highly Cooperative Chemical Inducers of Proximity

Bingqi Tong[1,2,3,4], Sunny A. Tang[3], Yifan Deng[1,2,3], Zhihan Nan[2,3], Alison X. Gao[1], Gregory A. Michaud[5], Simone Bonazzi[1], Frédéric Berst[6], Frédéric J. Zécri[1], Shuang Liu[2,3], William J. Gibson[2,4], Stuart L. Schreiber[2,3]

[1] Global Discovery Chemistry, Novartis Biomedical Research, Cambridge, MA, 02139, USA

[2] Chemical Biology and Therapeutics Science, Broad Institute of MIT and Harvard, Cambridge, MA, 02142, USA

[3] Department of Chemistry and Chemical Biology, Harvard University, Cambridge, MA, 02138, USA

[4] Department of Medical Oncology, Dana-Farber Cancer Institute, Boston, MA, 02215, USA

[5] Discovery Sciences, Novartis Biomedical Research, Cambridge, MA, 02139, USA

[6] Global Discovery Chemistry, Novartis Biomedical Research, Novartis Campus, Basel, CH-4002, Switzerland

Table of Contents

1. Supplementary Figures **3**

Figure S1. Chemical space coverage of different FKBP12-focused libraries 3

Figure S2. Screening analysis of CRBN CIP-DEL with BRD9 4

Figure S3. Mono-synthon analysis of VHL CIP-DEL screening with BRD9 5

Figure S4. Separation and characterization of diastereomers from the strained allene cycloaddition reaction of cycle-1 building block ID 67 6

Figure S5. Replicates of TR-FRET BRD9 binding assay and fitting results. 7

Figure S6. Full chemical structures and sequencing counts information of compounds synthesized off-DNA 8

Figure S7. TR-FRET protein-protein interaction assay 8

Figure S8. SPR sensorgrams and dose-response binding curves for binary binding 9

Figure S9. SPR sensorgrams under ternary conditions 10

Figure S10. CellTilter-Glo viability assay. 10

Figure S11. Chemical structures and calculated physicochemical properties of B67b-truncated and CFT8634 11

Figure S12. HiBiT assay measuring BRD9 degradation by B67b in HEK293T cells 11

Figure S13. Molecular dynamics simulations of ternary complex 12

Figure S14. RNA-seq differential gene expression analysis 12

2. Mathematical Modeling of Ternary Complex Equilibrium in TR-FRET Assays **13**

3. Protocols for DEL Screenings and Validation Assays **15**

4. General Synthetic Information **20**

4.1 General information for off-DNA synthesis 20

4.2 General information for on-DNA synthesis 20

5. DEL Synthesis **21**

5.1 Off-DNA synthesis of the strained allene precursor 21

5.2 Attachment of strained allene scaffold onto DNA headpiece 23

5.3 Building block validation for cycle-1 (strained allene cycloadditions) 25

5.4 Building block validation for cycle-2 (amide coupling reactions) 26

5.5 Cycle-3 building blocks (presenter protein-biasing elements) data (Table S1) 27

5.6 Synthesis of DEL common intermediate 32

5.7 Cycle-3 diversification of the library intermediate 33

6. Synthesis of DEL Screening Positive and Control Compounds **35**

7. Supplementary References **43**

Appendix I. Building block validation data (Table S2-S5) **44**

Appendix II. List of final included building blocks and associated DNA barcodes (Table S6) **60**

Appendix III. NMR spectra of compounds **72**

1. Supplementary Figures

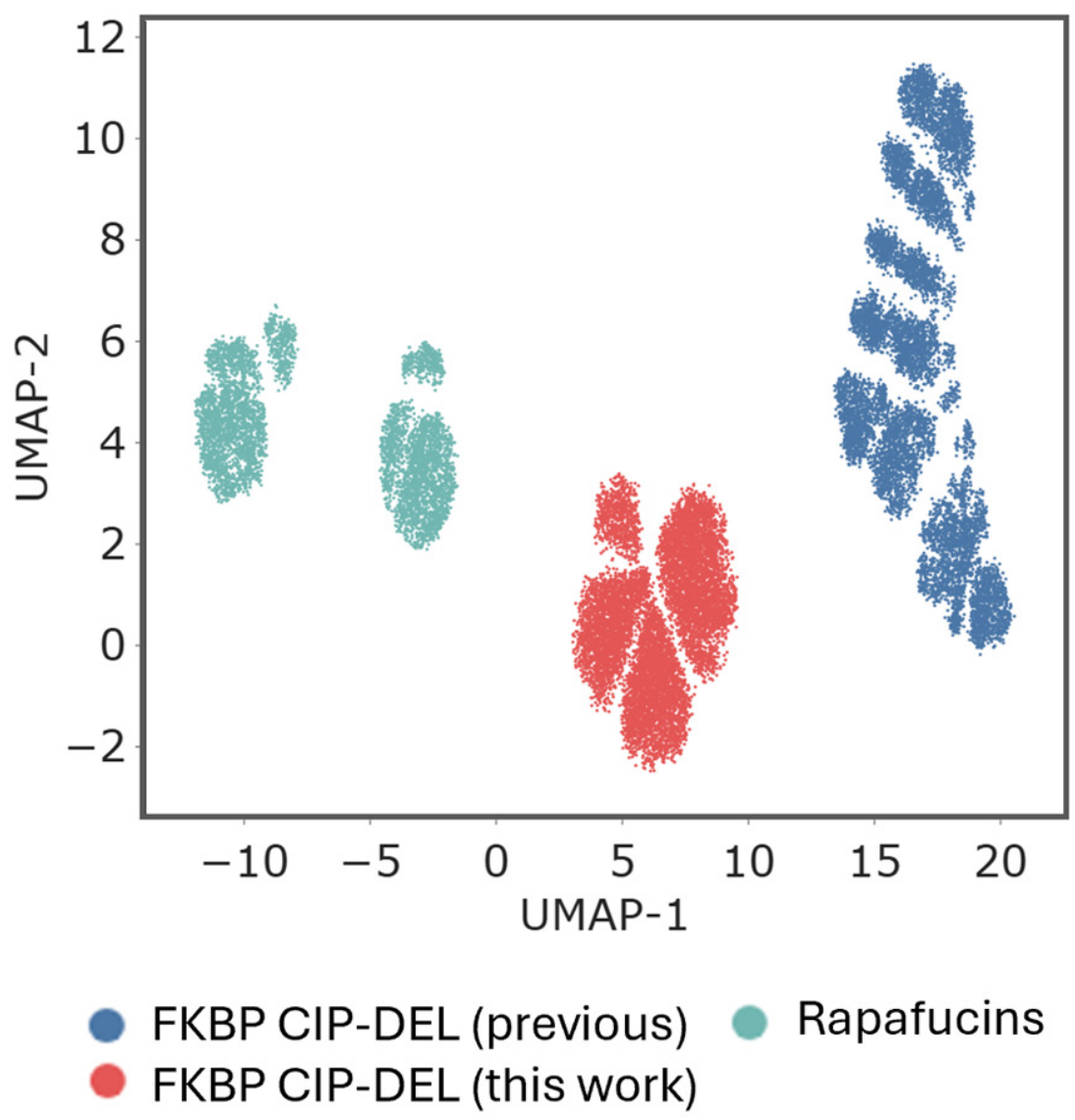

Figure S1. Chemical space coverage of different FKBP12-focused libraries, illustrated by the UMAP plot of Morgan fingerprints.

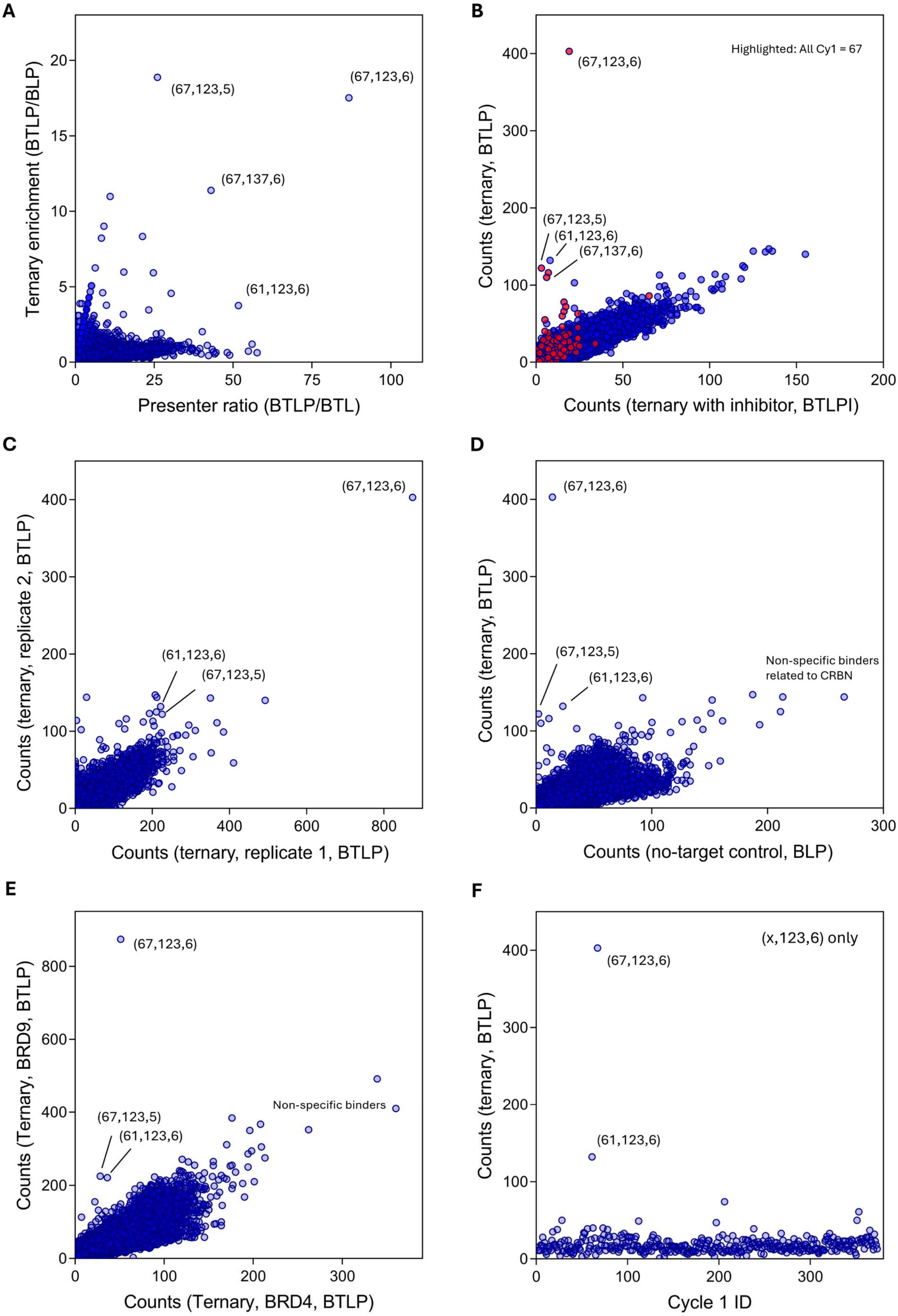
A
Ternary enrichment (BTLP/BLP)
Presenter ratio (BTLP/BTL)
(67,123,5)
(67,123,6)
(67,137,6)
(61,123,6)
B
Highlighted: All Cy1 = 67
(67,123,6)
(67,123,5)
(61,123,6)
(67,137,6)
Counts (ternary, BTLP)
Counts (ternary with inhibitor, BTLPI)
C
(67,123,6)
(61,123,6)
(67,123,5)
Counts (ternary, replicate 2, BTLP)
Counts (ternary, replicate 1, BTLP)
D
(67,123,6)
(67,123,5)
(61,123,6)
Non-specific binders related to CRBN
Counts (ternary, BTLP)
Counts (no-target control, BLP)
E
(67,123,6)
(67,123,5)
(61,123,6)
Non-specific binders
Counts (Ternary, BRD9, BTLP)
Counts (Ternary, BRD4, BTLP)
F
(x,123,6) only
(67,123,6)
(61,123,6)
Counts (ternary, BTLP)
Cycle 1 ID

Figure S2. Screening analysis of CRBN CIP-DEL with BRD9 shown as scatter plots. (a) Ternary enrichment ratio (BTLP/BLP, y-axis) versus presenter ratio (BTLP/BTL, x-axis), where the top right quadrant indicates both higher ternary enrichment and ternary/binary ratio. Both ratios are calculated as the lower bound of 95% confidence interval, based on Poisson statistics.[1-3] (b) Ternary counts (BTLP, y-axis) versus ternary counts in the presence of competitive inhibitor I-BRD9 (BTLPI, x-axis), same as Figure 3b but with all members of cycle-1 ID = 67 highlighted, showing the predominance of this series among hits. (c) ternary counts (BTLP) from two independent screens performed on different days, demonstrating good reproducibility. (d) Ternary counts (BTLP, y-axis) versus no-target control counts (BLP, x-axis), separating true binders from non-specific binders caused by the addition of CRBN-DDB1, similar to the competitor control experiment in Figure 3b. (e) Ternary counts in the BRD9 screen (BTLP, y-axis) versus ternary counts in the BRD4 screen (BTLP, x-axis), confirming the target-specificity of the BRD9 hits. (f) Ternary counts (BTLP, y-axis) of (x,123,6) compound series from CRBN CIP-DEL in the BRD9 screening, demonstrating the dependency on the cycle-1 stained allene cycloaddition building blocks. B, beads; T, target (BRD9); L, library; P, presenter protein (CRBN-DDB1); I, inhibitor (I-BRD9).

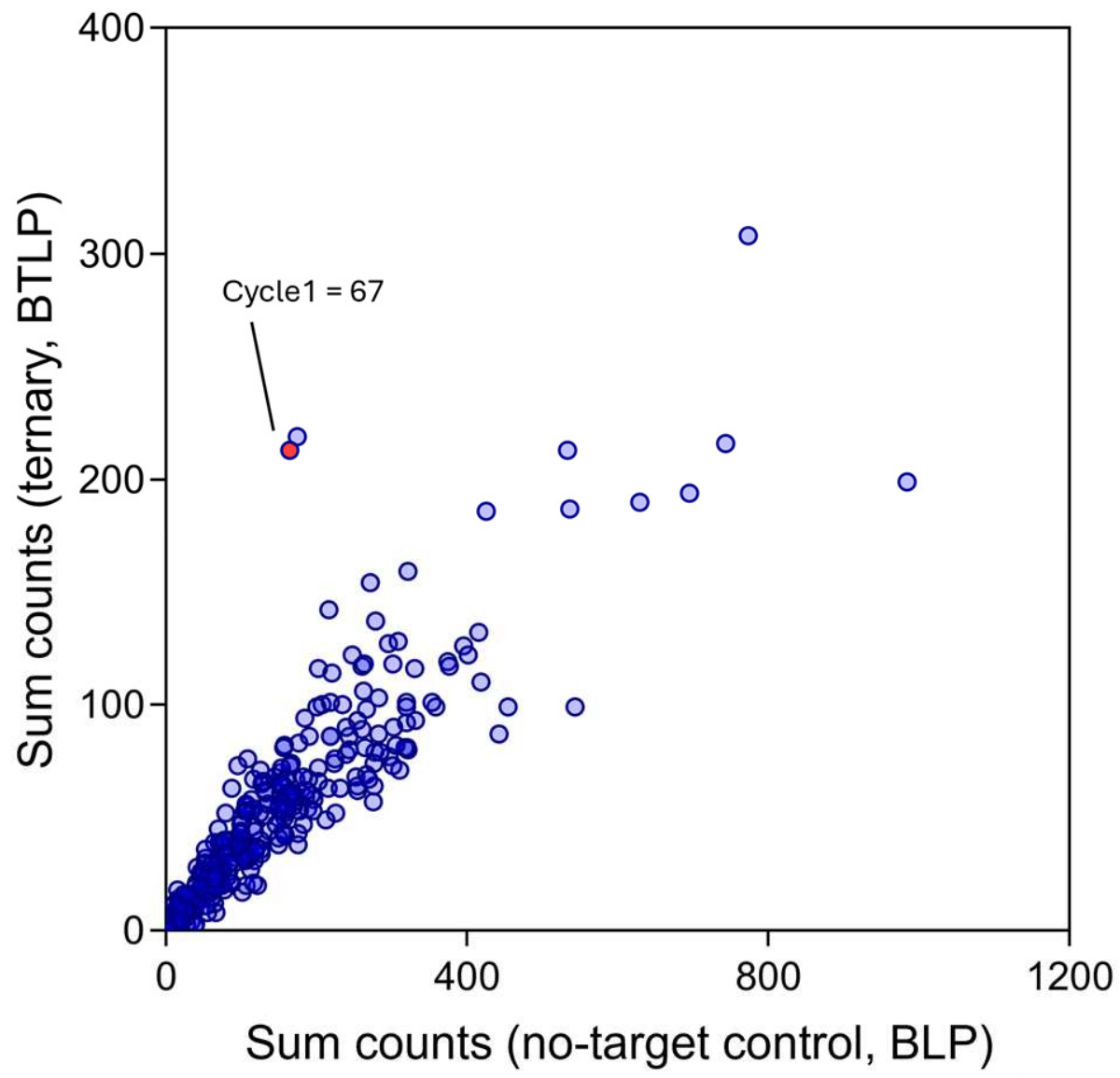

Figure S3. Mono-synthon analysis of VHL CIP-DEL screening with BRD9. Counts of library members sharing the same cycle-1 ID were summed and plotted as ternary (BTLP, y-axis) versus no-target control (BLP, x-axis). Cycle-1 ID = 67 stands out from the background (diagonal), indicating overall higher enrichment under ternary conditions compared with other library members. B, beads; T, target (BRD9); L, library; P, presenter protein (VCB).

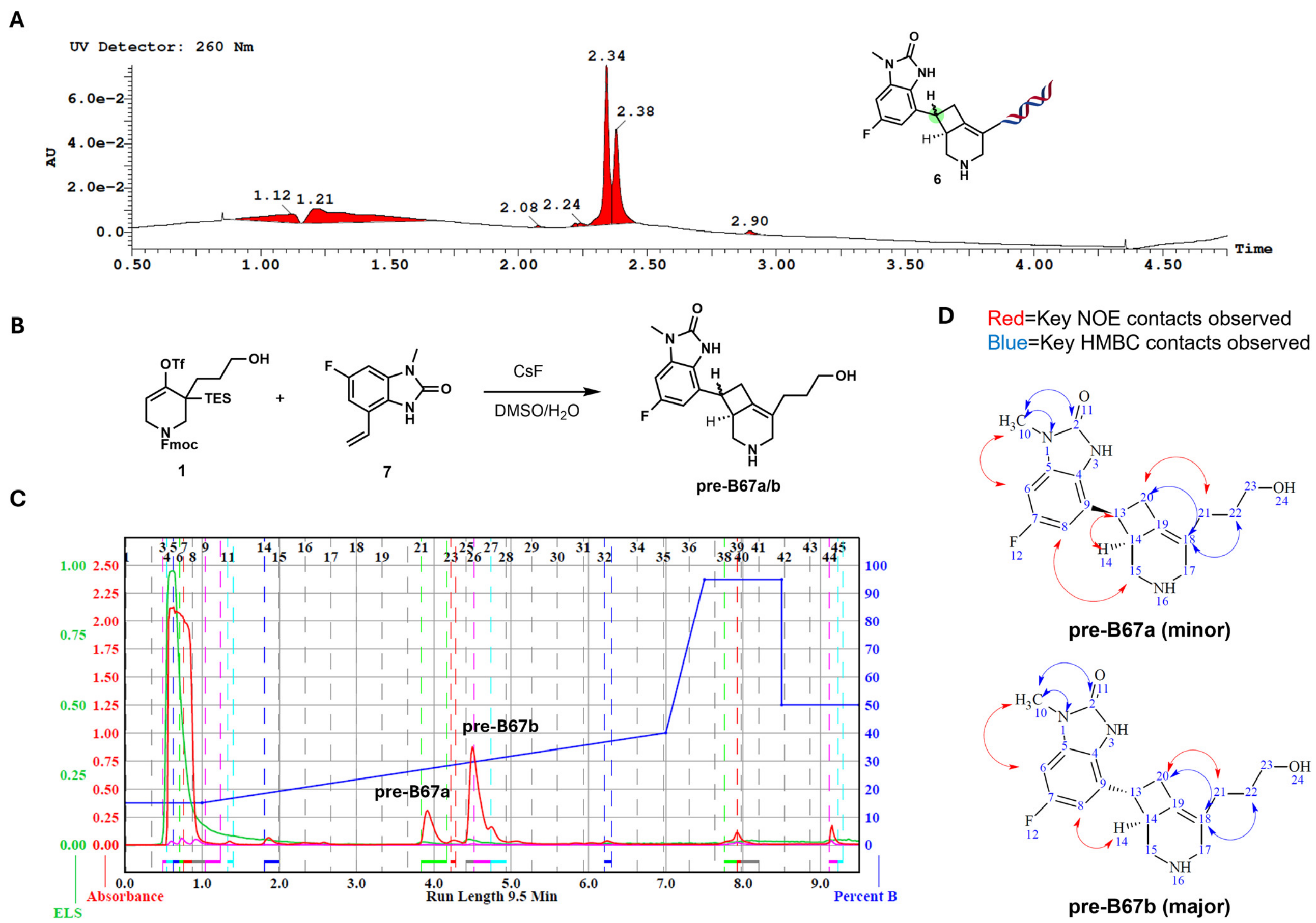

Figure S4. Separation and characterization of diastereomers from the strained allene cycloaddition reaction of cycle-1 building block ID 67. (a) UPLC UV trace (260 nm) of the DNA-conjugate after cycloaddition step, which is the precursor of hit compound in the final library, showing a mixture of diastereomers. (b) Reaction scheme of the cycloaddition step during off-DNA synthesis of **B67a/b**. (c) UV trace of the preparative HPLC separating the two diastereomers during off-DNA synthesis of **B67a/b**. (d) Key 2D-NMR correlations confirming the structure assignments of **B67a/b**. Full 2D spectra are provided in Appendix III.

A

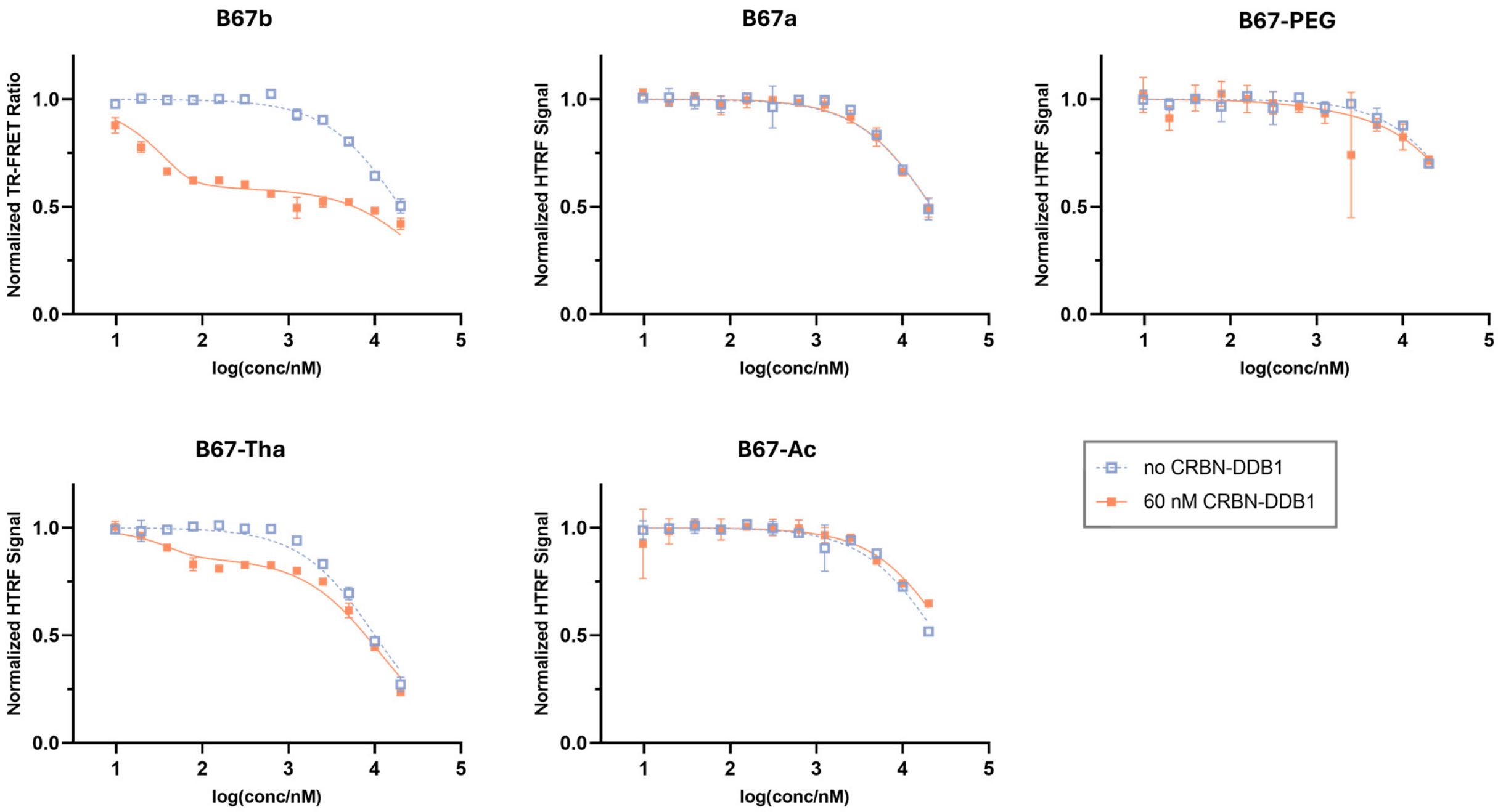

B

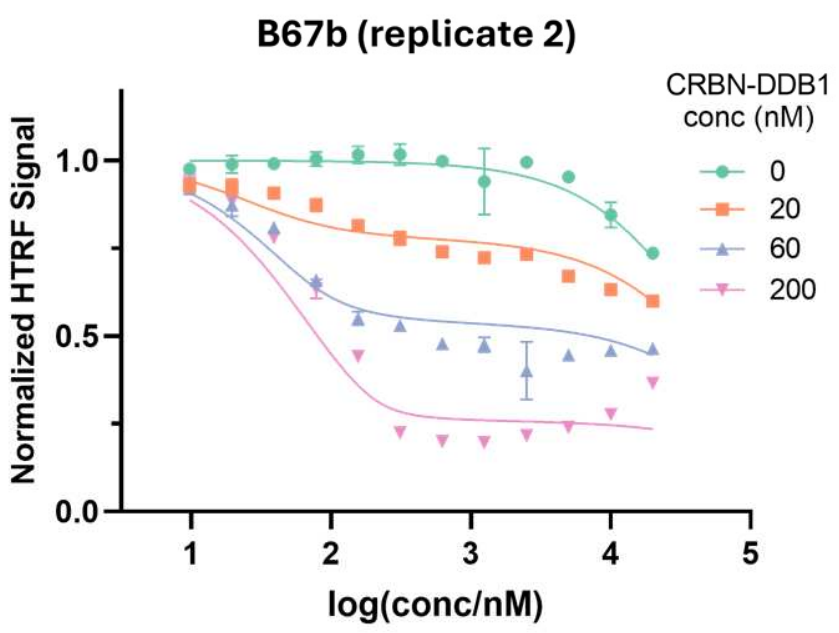

C

Fitting results of ternary equilibrium system

	Replicate 1	Replicate 2	Replicate 3*	Replicate 4*
K_d^B(BRD9) /μM	24.7 (21.6 – 28.4)	50.4 (40.0 – 66.1)	20.8 (18.1 – 24.0)	53.8 (43.9 – 68.0)
K_d^T(BRD9) /nM	81.5 (76.3 – 87.2)	64.8 (60.3 – 69.6)	68.1 (62.5 – 74.4)	58.1 (53.9 – 62.6)
R squared	0.9632	0.9561	0.9639	0.9716
α (K_d^B/K_d^T)	303	779	305	927

* Replicate 3 and 4 contains only two concentrations CRBN-DDB1 (0 and 60 nM).

Figure S5. Replicates of TR-FRET BRD9 binding assay and fitting results. (a) Dose-response curves of each compound in the presence (60 nM) or absence of CRBN-DDB1, with each compound plotted individually. (b) Dose-response curves of **B67b** at various CRBN-DDB1 concentrations (0, 20, 60, and 200 nM). Data in (a) and (b) are normalized to DMSO control and presented as mean ± SD, n = 2 technical replicates. (c) Summary of nonlinear fitting results using the equations described in Supporting Information Section 2. Data and fitting curves from replicates 1-4 were presented in Figure 3e, Figure S5b, Figure S5a, and Figure 3f, respectively.

A

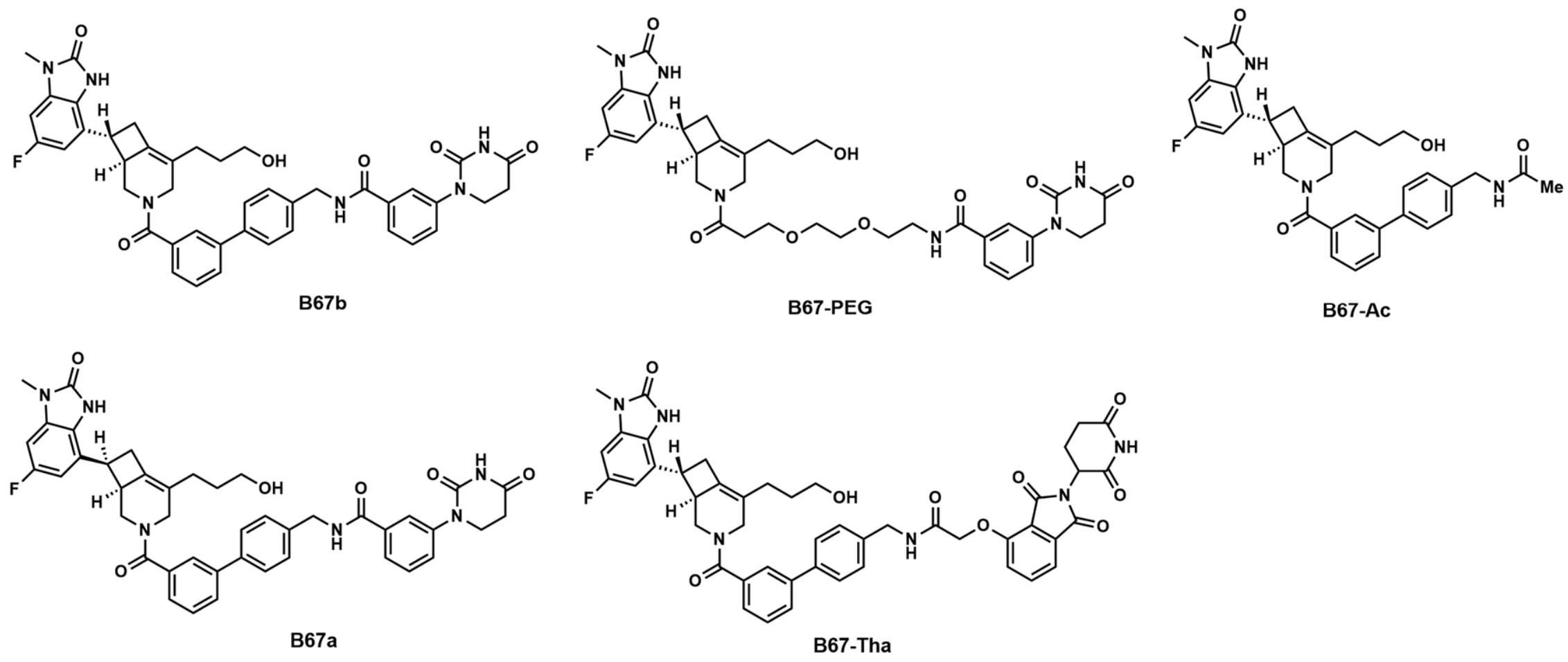

B

Compound	Cycle IDs	Beads only	No-target control	Binary	Ternary	Ternary with inhibitor
**B67a/b**	(67,123,6)	5	14	1	403	19
**B67-PEG**	(67,70,6)	0	2	0	6	0
**B67-Tha**	(67,123,1)	1	4	0	3	2

Figure S6. Full chemical structures (a) and sequencing counts (b) information of compounds synthesized off-DNA. Only one enantiomer was drawn for each compound.

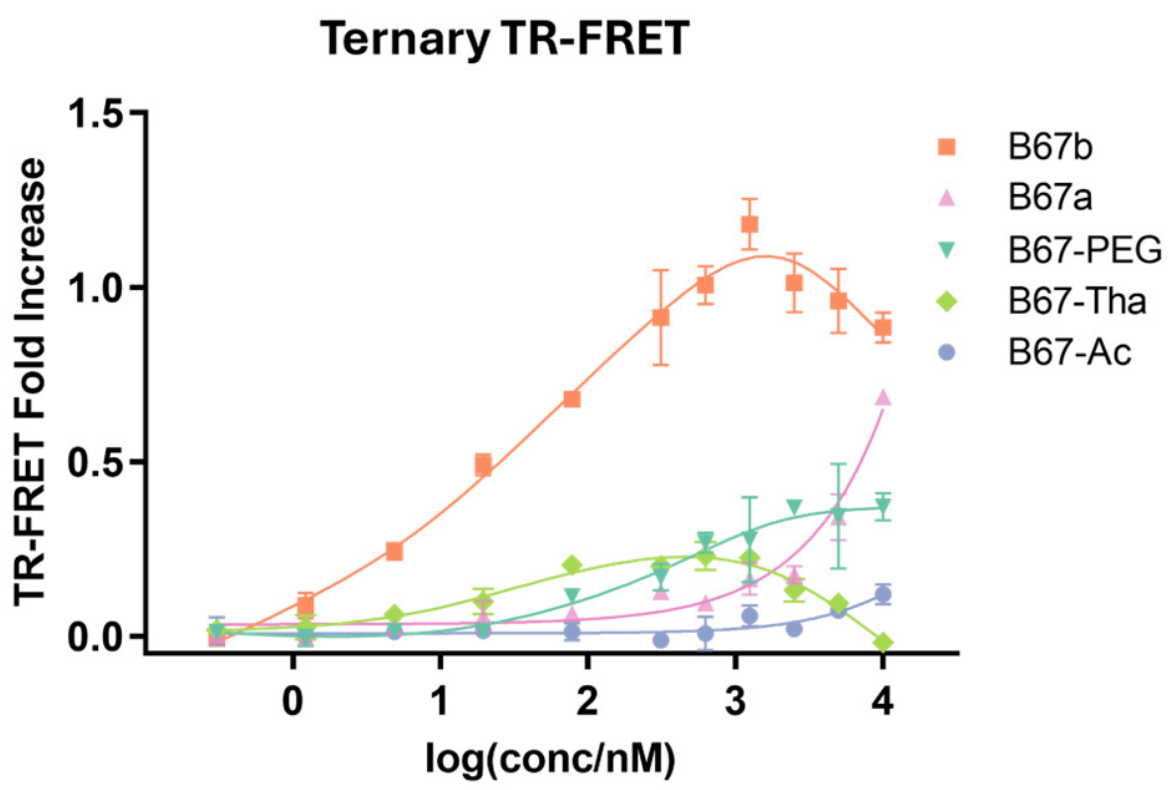

Figure S7. TR-FRET protein-protein interaction assay measuring compound-induced ternary complex formation between His-tagged CUL4A-RBX1-DDB1-CRBN and biotinylated BRD9 using ULight-labeled streptavidin and europium-labeled anti-6X His antibody. Data are normalized to DMSO control and presented as mean ± SD, n = 2 technical replicates.

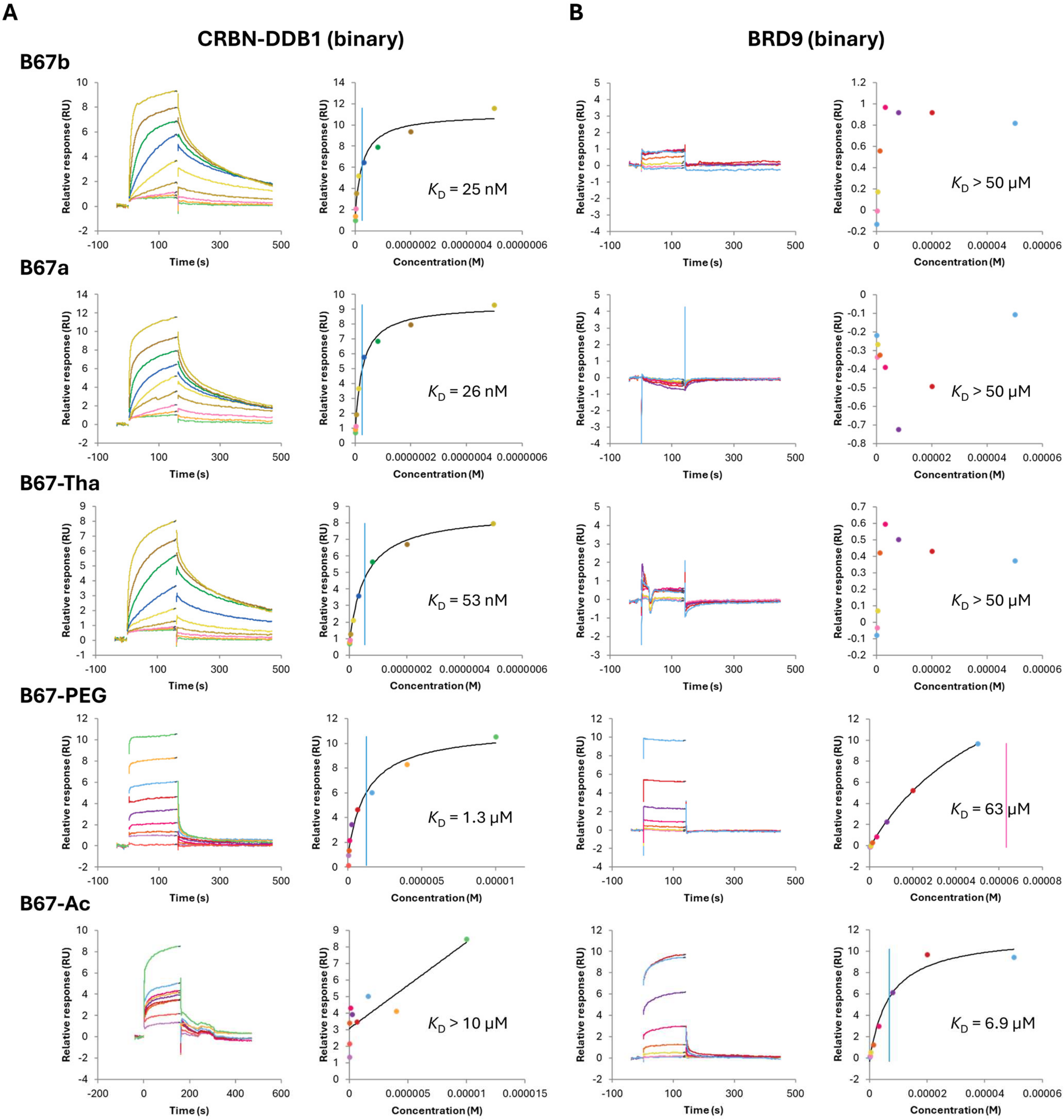

Figure S8. SPR sensorgrams and dose-response binding curves of each compound for binary binding to immobilized CRBN-DDB1 (a) and BRD9 (b). K_d values were calculated using steady state fitting in Biacore Evaluation Software.

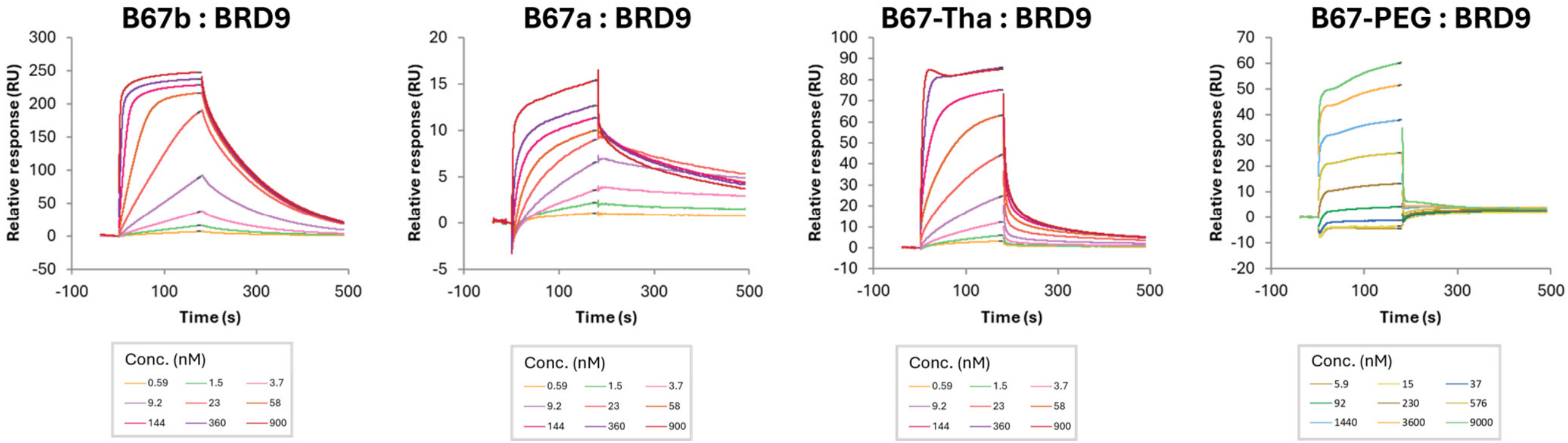

Figure S9. SPR sensorgrams under ternary conditions with CRBN-DDB1 immobilized on the sensor surface and the compound in the presence of 1.0 μM BRD9 as analyte.

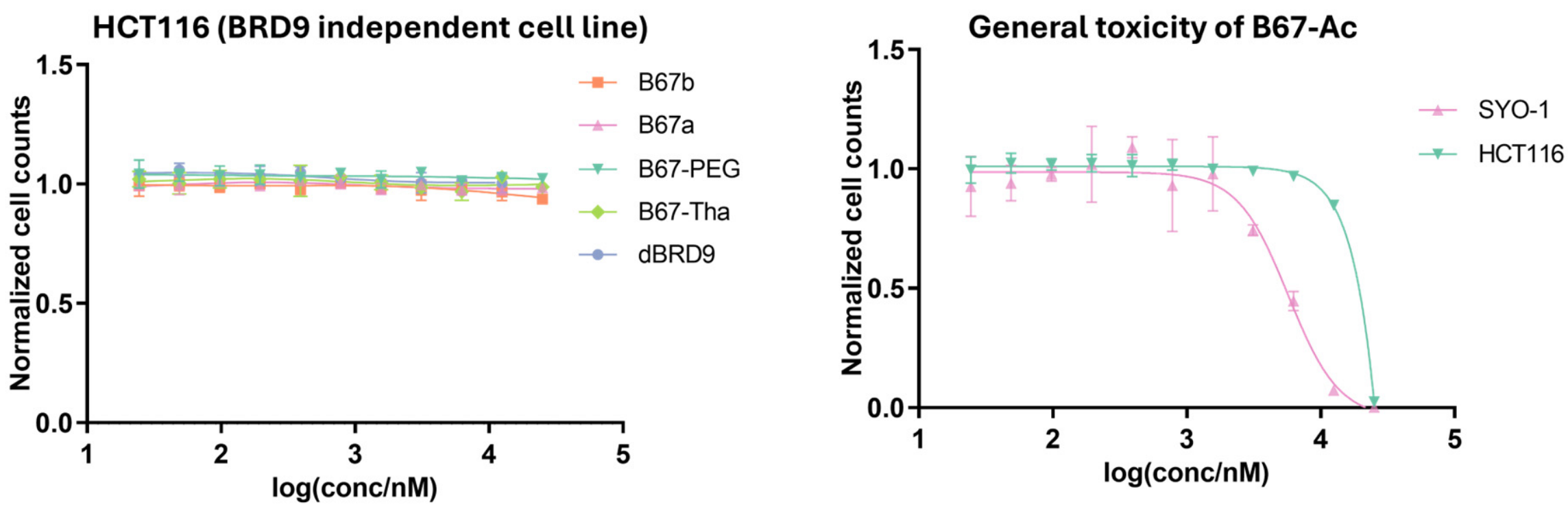

Figure S10. CellTilter-Glo viability assay. (a) Dose-response viability of HCT116 cells treated with **B67b** or control compounds, showing no anti-proliferation activity. (b) **B67-Ac** exhibits strong general toxicity at high concentrations in both SYO-1 and HCT116 cells, indicating the effect is unrelated to BRD9. Data are normalized to DMSO control and presented as mean ± SD, n = 2 technical replicates.

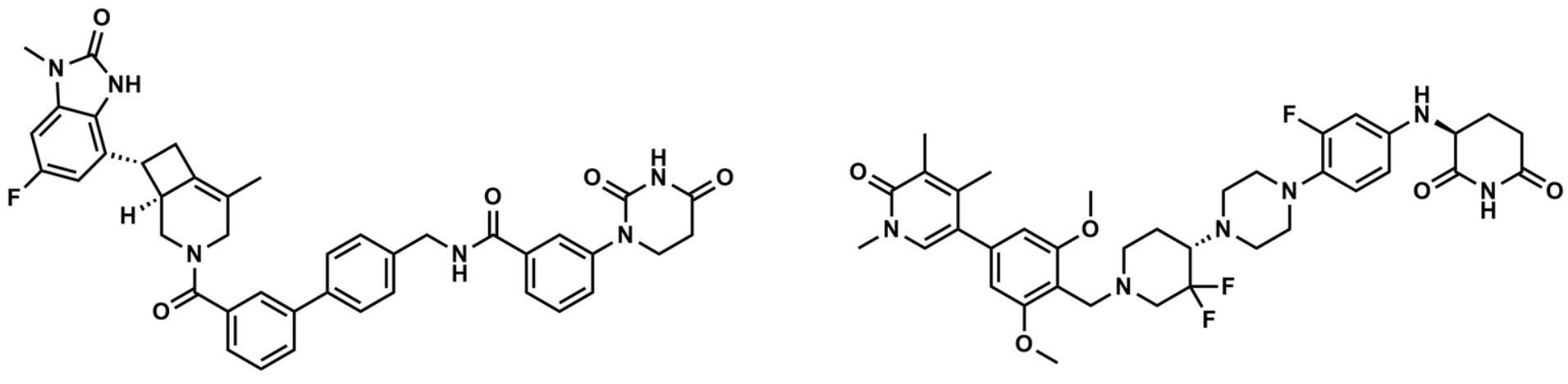

	Molecular Weight	# Rotatable Bonds	# H-Bond Donor	# H-Bond Acceptor	SlogP	TPSA	sp3 fraction
**B67b-truncated**	712.8	7	3	6	5.6	137	0.24
**CFT8634**	710.8	9	2	10	4.1	108	0.49

Figure S11. Chemical structures and calculated physicochemical properties of B67b-truncated and CFT8634.

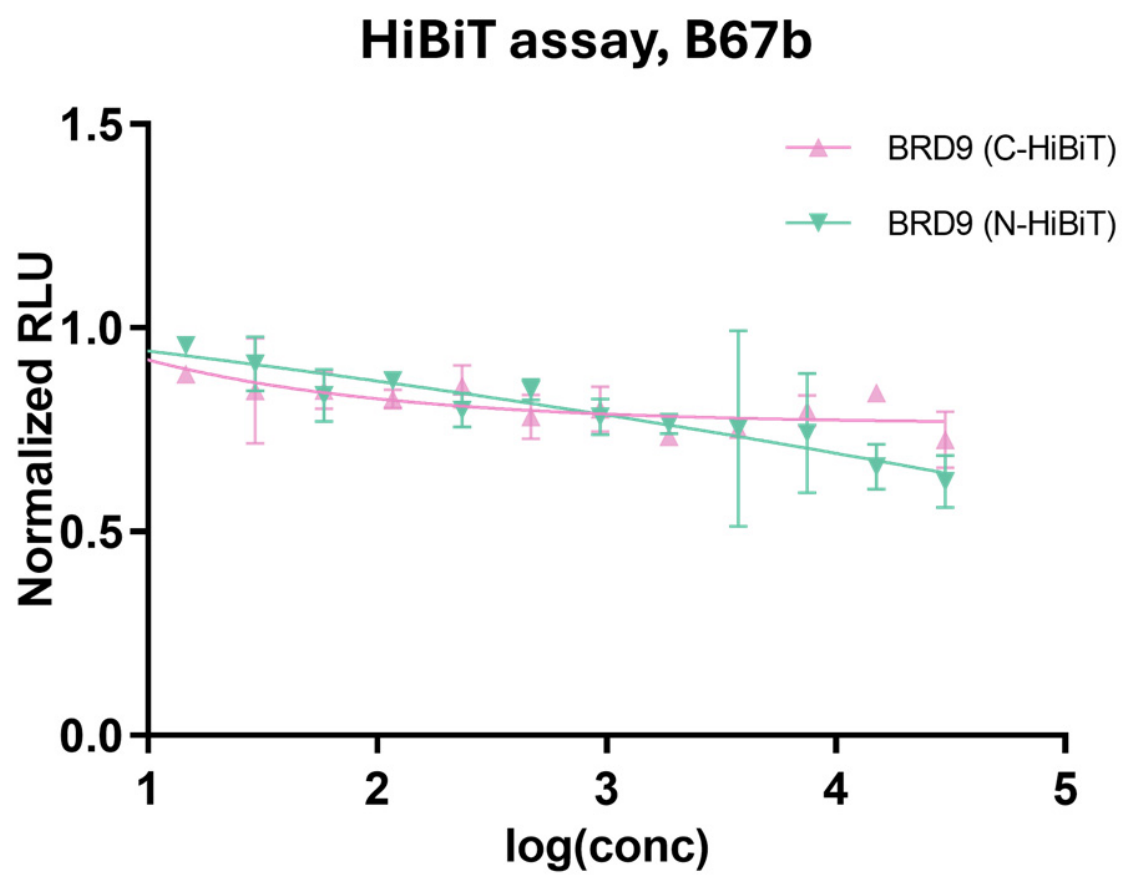

Figure S12. HiBiT assay measuring BRD9 degradation by **B67b** in HEK293T cells using both N- and C-terminus HiBiT constructs, showing no significant degradation. Data are normalized to DMSO control and presented as mean ± SD, n = 2 technical replicates.

A

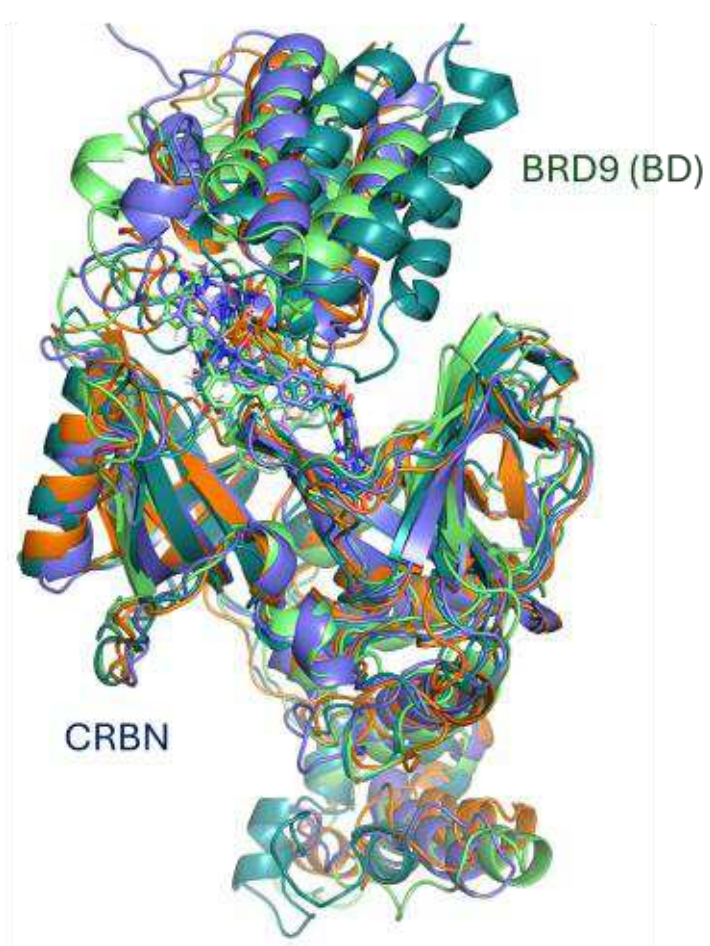

B

List of all interface hydrogen bonds with occupancy >20% during MD simulations

Compounds	Acceptor	Donor	Occupancy (%)
B67-ent1	No H-bond observed >20% occupancy		
B67-ent2	ASP_156@O	GLN_129@NE2	76.9
	GLY_159@O	ARG_131@NH2	58
	GLN_129@O	GLN_153@NE2	54.7
	GLY_159@O	ARG_131@NH1	49.8
B67a-ent1	No H-bond osberved >20% occupancy		
B67a-ent2	PRO_157@O	ARG_131@NH2	37.5
	GLU_377@OE2	ARG_217@NH2	30.8
	GLU_377@OE1	ARG_217@NH1	29.6

Figure S13. Molecular dynamics simulations of ternary complex. (a) Overlay of the ternary complex structures of the representative frames for each B67a/b stereoisomer. Green: B67b-ent1; orange: B67b-ent2; cyan: B67a-ent1; purple: B67a-ent2. (b) List of all hydrogen bonds with occupancy >20%

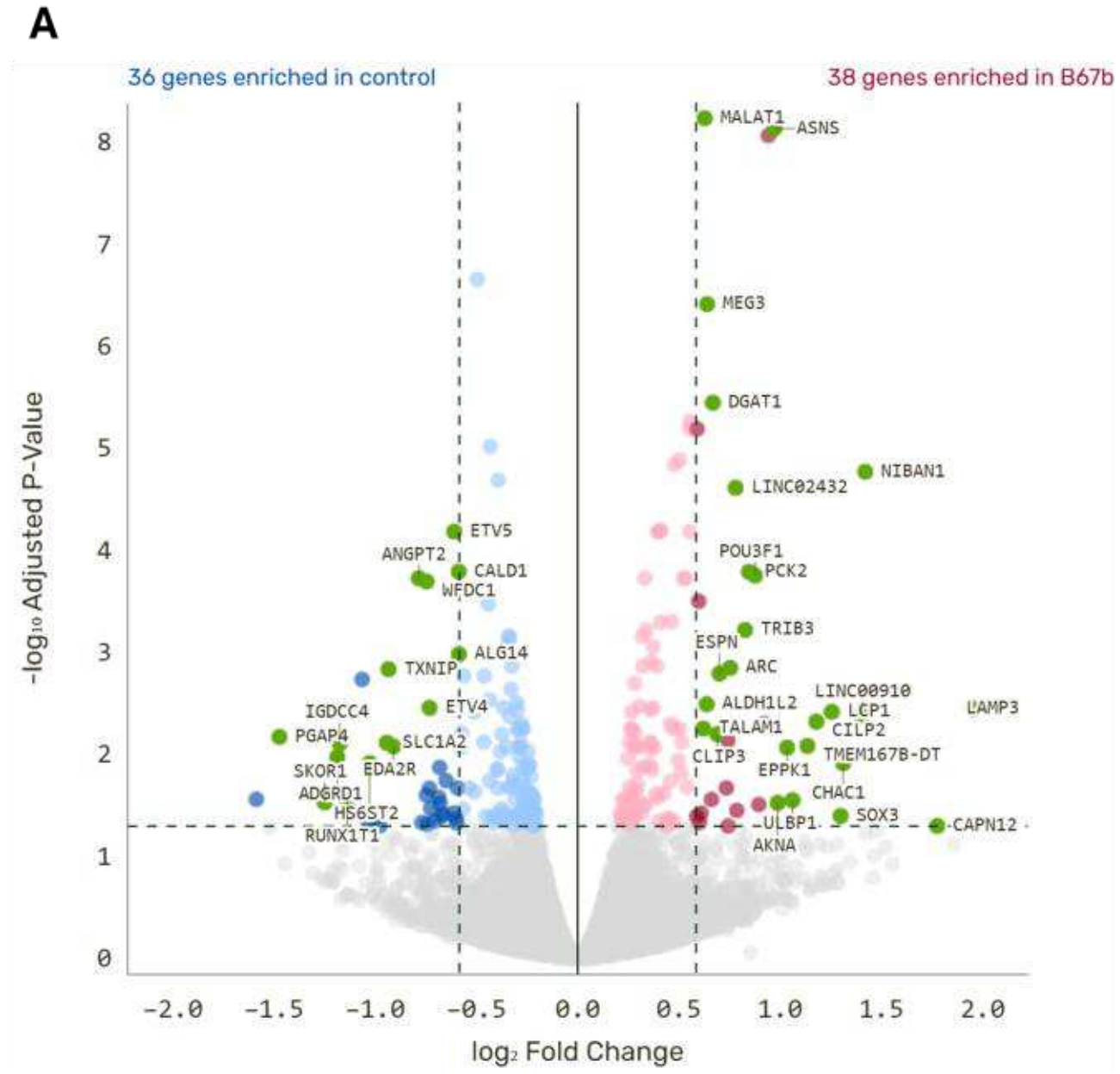

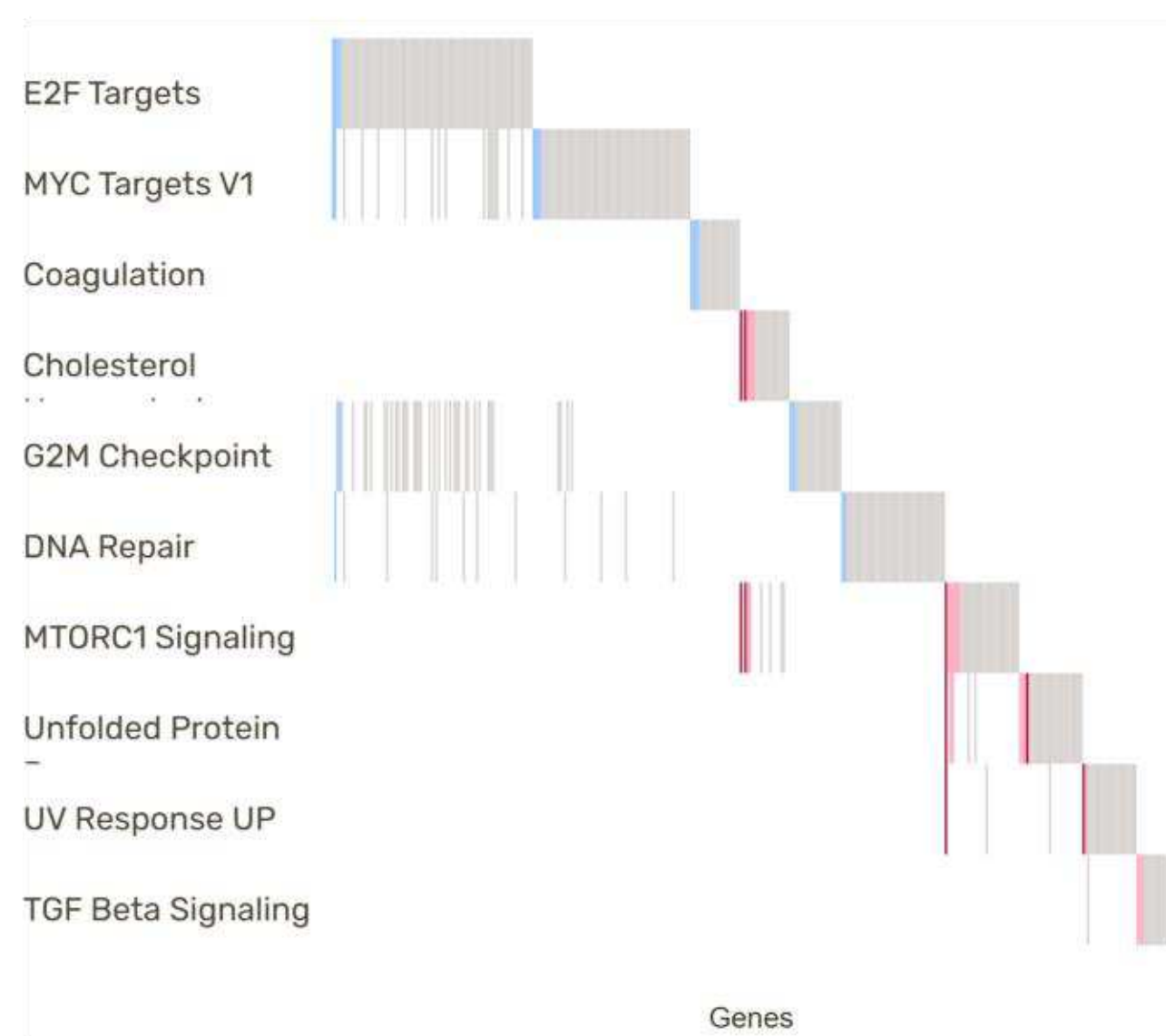

Figure S14. RNA-seq differential gene expression analysis of SYO-1 cells upon 24 h treatment of B67b at 5 μM (n = 2). (a) Volcano plot of the enriched genes versus DMSO control. Dashed lines represents fold-change of 1.5 and p-Value of 0.05. (b) Top-enriched pathways Sorted on Adj p-Value in ascending order.

2. Mathematical Modeling of Ternary Complex Equilibrium in TR-FRET Assays

T: target protein (BRD9)
Pre: presenter protein (CRBN)
L: ligand (test compounds)
*L**: tracer ligand with fluorescence dye
"*c*" denotes total input concentration and the bracket "[ ]" denotes exact species concentration.

The equilibrium of all the species in the assay system can be expressed as:

$$[L][T] = K_d^B \cdot [T \cdot L] \tag{1}$$

$$[L^*][T] = K_d^{L^*}[T \cdot L^*] \tag{2}$$

$$[Pre][L] = K_d^{pre}[Pre \cdot L] \tag{3}$$

$$[Pre \cdot L][T] = K_d^T [T \cdot L \cdot Pre] \tag{4}$$

$$c_T = [T] + [T \cdot L] + [T \cdot L^*] + [T \cdot L \cdot Pre] \tag{5}$$

$$c_L = [L] + [T \cdot L] + [Pre. L] + [T \cdot L \cdot Pre] \tag{6}$$

$$c_{Pre} = [Pre] + [Pre \cdot L] + [T \cdot L \cdot Pre] \tag{7}$$

$$c_{L^*} = [L^*] + [T \cdot L^*] \tag{8}$$

Substitute (1), (2), (4) into (5), we get:

$$c_T = \frac{K_d^{L^*}}{[L^*]}[T \cdot L^*] + \frac{[L]}{K_d^B} \cdot \frac{K_d^{L*}}{[L^*]}[T \cdot L^*] + [T \cdot L^*] + \frac{[Pre \cdot L]}{K_d^T} \cdot \frac{K_d^{L^*}}{[L^*]}[T \cdot L^*]$$

Thus

$$[T \cdot L^*] = \frac{c_T}{\left(\frac{K_d^{L^*}}{[L^*]} \cdot \left(1 + \frac{[L^*]}{K_d^{L*}} + \frac{[L]}{K_d^B} + \frac{[Pre \cdot L]}{K_d^T}\right)\right)} \tag{9}$$

By combining (8) and (9), $[T \cdot L^*]$ can be accurately solved as a function of $[L]$ and $[Pre \cdot L]$:

$$[T \cdot L^*] = \frac{1}{2} \times \left(c_T + c_{L*} + K_d^{L^*} \cdot A - \sqrt{(c_T + c_{L*} + K_d^{L*} \cdot A)^2 - 4c_T c_{L*}}\right)$$
$$\text{where } A = 1 + \frac{[L]}{K_d^B} + \frac{[Pre \cdot L]}{K_d^T} \tag{10}$$

The equation (10) is a precise solution of equation (9). However, we could use approximations to simply equation (9) and make further calculation easier. Since target concentration c_T in our assay conditions is very small, we have $c_{L^*} \gg c_T$, thus $[L^*] \approx [L^*]_0 \approx c_{L^*}$, and put it in equation (9), we have:

$$[T \cdot L^*] = \frac{c_T}{\left(\frac{K_d^{L^*}}{c_{L^*}} \cdot \left(1 + \frac{c_{L*}}{K_d^{L*}} + \frac{[L]}{K_d^B} + \frac{[Pre \cdot L]}{K_d^T}\right)\right)} \tag{11}$$

and

$$[T \cdot L^*]_0 \approx \frac{c_T}{\frac{K_d^{L^*}}{c_{L^*}} \cdot \left(1 + \frac{c_{L^*}}{K_d^{L^*}}\right)} \tag{12}$$

where $[L^*]_0$ and $[T \cdot L^*]_0$ denote the concentration without testing compounds ($c_L = 0$).

Thus, the normalized TR-FRET signal

$$y = \frac{[T \cdot L^*]}{[T \cdot L^*]_0} \approx \frac{\left(1 + \frac{c_{L^*}}{K_d^{L^*}}\right)}{\left(1 + \frac{c_{L^*}}{K_d^{L^*}} + \frac{[L]}{K_d^B} + \frac{[Pre \cdot L]}{K_d^T}\right)} \tag{13}$$

To calculate $[L]$ and $[Pre \cdot L]$, we can ignore $[T \cdot L] + [T \cdot L \cdot Pre]$ in equation (6) under our assay condition, because: (a) In high c_L range, $c_L \gg c_T > [T \cdot L] + [T \cdot L \cdot Pre]$; (b) In low c_L range, $c_L \sim c_T \gg [T \cdot L] + [T \cdot L \cdot Pre]$ (low target occupancy at low c_L). Thus, equation (6) can be simplified to

$$c_L \approx [L] + [Pre \cdot L] \tag{14}$$

Combine (14) with (3), $[L]$ and $[Pre \cdot L]$ can be solved:

$$[L] = \frac{1}{2}\left(c_L - K_d^{Pre} - c_{Pre} + \sqrt{\left(K_d^{\mathrm{pre}} + c_{\mathrm{pre}} - c_L\right)^2 + 4c_L K_d^{Pre}}\right) \tag{15}$$

Combine equations (13), (14) and (15), the normalized TR-FRET signal (y) can be written as a function of compound concentration (c_L), thus a nonlinear regression fitting could be performed. In our assay setup, $c_{L^*} = 33$ nM, $c_{Pre} = 0, 20, 60$ *or* 200 nM, $K_d^{L^*} = 0.68$ µM and $K_d^{Pre} = 25$ nM (for **B67b**) were determined by SPR. K_d^B and K_d^T were then fitted globally with least squares regression method.

Fitting results were shown in Figure S5c. Fitting curves of replicate 1-4 were presented in Figure 3e, Figure S5b, Figure S5a, and Figure 3f, respectively. Replicates 3 and 4 contain only two concentrations of CRBN-DDB1 (0 and 60 nM). The observed differences in K_d^B between replicate 1&3 and replicate 2&4 are likely due to the variation between protein batches. The binary bindings were very weak, thereby easily influenced by subtle experimental variations, whereas K_d^T was largely unaffected, yielding reliable and stable fitted values. K_d^{Pre} could also be fitted from the assay data (instead of using SPR-measured value of 25 nM) resulting in a slightly higher fitted value. Regardless, the fitting results of K_d^B and K_d^T is not sensitive to the moderate change of K_d^{Pre}, indicating that their fitting results are robust.

3. Protocols for DEL Screening and Validation Assays

Recombinant Proteins

N-terminal His-tagged BRD9, N-terminal biotinylated BRD9, tagless BRD9, tagless VCB (VHL-elongin C-elongin B) and tagless FKBP12 were prepared according to literature procedures.[3,4] N-terminal biotinylated CRBN-DDB1 and tagless CRBN-DDB1 were generous gifts from Novartis. CUL4A-RBX1-DDB1-CRBN (N-terminal 10-His tagged Cullin-4A, untagged RBX1, untagged DDB1 and untagged CRBN) was purchased from R&D Systems (E3-650-025).

DEL Screening

DEL screenings were performed as previously described, using a KingFisher Duo Prime (Thermo Scientific) in a 96-well Deepell plate (Thermo Scientific 95040452) at room temperature.[3] The binding buffer (B Buffer) consists of 25 mM HEPES pH 7.4, 150 mM NaCl, 0.05% Tween-20 (w/v), and the selection buffer (S Buffer) consists of 25 mM HEPES, pH 7.4, 150 mM NaCl, 0.05% Tween-20 (w/v), 0.3 mg/mL Ultrapure Salmon Sperm DNA (ThermoFisher Scientific 15632011). His-Tag Dynabeads (Invitrogen 10103D, 10 µL per sample) were washed three times with B Buffer and incubated with His-tagged target protein (2.0 µM in 100 µL B Buffer) for 1h with medium mixing. The beads were then washed once with B Buffer (200 µL) and twice with S buffer (200 µL, 3 min each, medium mixing). After that, the beads were incubated (1h, medium mixing) with the DNA-encoded library (10 million copies per library member, 100 µL in S Buffer), in the presence or absence of a presenter protein (CRBN-DDB1, VCB, or FKBP12). For the BRD9 inhibitor competition experiment, 10 µM I-BRD9 (final 0.1% DMSO) was also added. The beads were subsequently washed once with S Buffer (200 µL) and twice with B Buffer (200 µL, 3 min each, medium mixing). Bound DEL members were eluted by heating the beads in B Buffer (100 µL) at 90 °C for 10 min. 20 µL of the resulting supernatant was restriction digested with StuI (0.1 µL, NEB R0187) in 1× SmartCutter buffer (56.5 µL, NEB B7204S) per sample (37 °C, 1 h) and cleaned up using the ChargeSwitch PCR Clean-Up Kit (Thermo Scientific CS12000). 19 µL per sample of the eluted DEL barcodes were PCR amplified using i5 indexing primer (3 µL of 10 µM stock), i7 indexing primer (3 µL of 10 µM stock), and Platinum Hot Start PCR Master Mix (25 µL of 2×, Invitrogen 13000012). The PCR method was as follows: 95 °C for 2 min; 15-25 cycles of 95 °C for 15 s, 55 °C for 15 s, 72 °C for 30 s; final extension at 72 °C for 7 min; hold at 4 °C. PCR products were pooled in equimolar amounts and purified using 2% E-Gel EX Agarose Gel (ThermoFisher Scientific G401002) and the QIAquick Gel Extraction Kit (Qiagen 28704). The purified DNA sample was sequenced on Illumina HiSeq or NovaSeq instruments with a 50-cycle single-read and two 8-cycle index reads using the following custom primers: Read 1 Primer CTTAGCTCCCAGCGACCTGCTT CAATGTCGGATAGTG; i7-Index Primer: CTGATGGAGGTAGAAGCCGCAGTGAGCATGGT; i5-Index Primer: CACTATCCGACATTGAAGCAGGTCGCTGGGAGCTAAG. The sequencing data was analyzed as previously described.[2]

TR-FRET BRD9 binding assays

The TR-FRET BRD9 binding assay was carried out in white 384-well assay plates (Greiner 784075) with a final assay volume of 15 µL. All reagents were diluted in the assay buffer containing 20 mM TRIS, pH 7.0, 20 mM NaCl, 0.005% Tween-20, and 0.05% Bovine serum albumin (BSA). Mixtures of His-tagged BRD9 (final 2.7 nM) and CRBN-DDB1 (final 0, 20, 60 or 200 nM) were plated at 5 µL per well. Compounds were added from serially diluted DMSO stocks (40 nL per well, final top concentration 20 µM) using an Echo 655 acoustic liquid handler. Cy5-labelled tracer ligand (5 µL, final concentration 33 nM) and Europium-labeled anti-6X His antibody (Revvity AD0111, 5 µL, final concentration 0.5 nM)

were added to each well, and the plate was incubated for 90 minutes under cover. The FRET signals were measured on a PHERAstar FS plate reader (BMG Labtech) with the following settings: channel A 337/665 nm excitation/emission, channel B 337/620 nm excitation/emission, delay time 50 µs, integration time 400 µs. The TR-FRET ratio was calculated as the 665/620 nm intensity ratio on a per-well basis and then normalized to DMSO-treated wells. Data were analyzed in GraphPad Prism v9 using the equations described in the Results section.

AlphaScreen Assays

AlphaScreen assays were carried out in 384-well AlphaPlate (Revvity 6008350) with a final assay volume of 15 µL. All reagents were diluted in the assay buffer containing 25 mM HEPES, pH 7.4, 150 mM NaCl, 0.01% Tween-20, and 0.1% BSA. A mixture of biotinylated BRD9 (final concentration 25 nM) and His-tagged CUL4A-RBX1-DDB1-CRBN complex (final concentration 15 nM) was plated at 10 µL per well. Compounds were added from serially diluted DMSO stocks (50 nL, final top concentration 22.5 µM) using an Echo 655 acoustic liquid handler. Under low-light conditions, a 5 µL mixture of diluted streptavidin donor beads (Revvity 6760619C, final 0.02 mg/mL) and nickel chelate (Ni-NTA) acceptor beads (Revvity 6760619C, final 0.02 mg/mL) was added to each well. The plate was incubated for 1 h at room temperature in the dark before luminescence readings were taken on a PHERAstar FS plate reader (BMG Labtech) equipped with an AlphaScreen optic module (Ex AS/Em AS filters) with the following settings: 0.3 s excitation time, 0.34 s integration start, and 0.6 s integration time. Data were analyzed using GraphPad Prism v9.

TR-FRET ternary complex assay

The ternary TR-FRET assay was carried out in white 384-well assay plates (Greiner 784075) with a final assay volume of 15 µL. All reagents were diluted in the assay buffer containing 20 mM TRIS, pH 7.0, 20 mM NaCl, 0.005% Tween-20, and 0.05% BSA. A mixture of biotinylated BRD9 (final concentration 50 nM) and His-tagged CUL4A-RBX1-DDB1-CRBN complex (final concentration 5 nM) was plated at 10 µL per well. Compounds were added from serial diluted DMSO stocks (40 nL per well, final top concentration 20 µM) using an Echo 655 acoustic liquid handler. A 5 µL mixture of diluted ULight-labeled Streptavidin (Revvity TRF0102, final 50 nM) and Europium-labeled anti-6X His antibody (Revvity AD0111, final 0.5 nM) was then added to each well, and the plate was incubated for 2 hours at room temperature. FRET signals were measured on a PHERAstar FS plate reader (BMG Labtech) with the same settings as described for the TR-FRET BRD9 binding assay. Data were analyzed using GraphPad Prism v9.

Surface Plasmon Resonance (SPR)

Binary binding for CRBN. The experiments were performed on a Biacore 8K instrument (Cytiva) at 15 °C using a running buffer containing 25 mM HEPES, pH 7.5, 150 mM KCl, 1 mM Dithiothreitol (DTT), 0.05% Tween-20, and 2% DMSO. Biotinylated CRBN-DDB1 was immobilized to 3800-4000 RU on a Series S SA sensor chip (Cytiva BR100531) that had been preconditioned with 1 M NaCl and 40 mM NaOH. Compounds were 2.5-fold serially diluted with the running buffer (containing 2% DMSO) from a top concentration of 500 nM (for **B67a**, **B67b**, **B67-Tha**) or 10 µM (for **B67-PEG**, **B67-Ac**). The compound solutions were injected at 35 µL/min with 160 s contact time and 300 dissociation time using the multi-cycle method. The syringe was washed with 50% DMSO/H_2O between injections. K_d values were calculated using the steady state affinity mode in Biacore Evaluation Software.

***Binary binding for BRD9**.* The experiments were performed on a Biacore 8K instrument (Cytiva) at 25 °C using a running buffer containing 20 mM HEPES, pH 7.5, 100 mM NaCl, 0.2 mM tris(2-carboxyethyl)phosphine (TCEP) 0.05% Tween-20, and 2% DMSO. His-tagged BRD9 was captured and covalently linked to a Series S NTA sensor chip (Cytiva BR100532) at ~ 400 RU by sequential injections (10 µL/min) of following solutions: 300 mM EDTA (60 s), 0.5 mM $NiCl_2$ (100 s), 3 mM EDTA (60 s), EDC/NHS (1:1, Cytiva BR100050, 400 s), His-BRD9 (flow cell 2 only), 100 mM Tris pH 7.5 (3 x 60 s). Compounds were 2.5-fold serially diluted with the running buffer (2% DMSO) from a top concentration of 50 µM. The compound solutions were injected at 40 µL/min with 140 s contact time and 300 dissociation time using the multi-cycle method. The syringe was washed with 50% DMSO/H_2O between injections. K_d values were calculated using the steady state affinity mode in Biacore Evaluation Software.

***Ternary binding**.* The experiments were performed on a Biacore 8K instrument (Cytiva) at 15 °C using a running buffer containing 25 mM HEPES, pH 7.5, 150 mM KCl, 1 mM Dithiothreitol (DTT), 0.05% Tween-20, and 2% DMSO. Biotinylated CRBN-DDB1 was immobilized to ~ 4000 RU on a Series S SA sensor chip (Cytiva BR100531) that had been preconditioned with 1 M NaCl and 40 mM NaOH. Compounds were 2.5-fold serially diluted with the running buffer (2% DMSO) from a top concentration of 1.0 µM (for **B67a**, **B67b**, **B67-Tha**) or 10 µM (for **B67-PEG**). Then tagless BRD9 (10 µM in 2% DMSO buffer) was added to each sample to a final concentration of 1.0 µM. The sample solutions were injected at 30 µL/min with 180 s contact time and 300 dissociation time using the multi-cycle method. The syringe was washed with 50% DMSO/H_2O between injections. K_d (apparent) values were calculated using the steady state affinity mode in Biacore Evaluation Software.

NanoBiT assay

HEK293T cells (5 mL) were plated at 0.625 × 10^6 cells/mL in DMEM (Gibco 11995065) supplemented with 10% fetal bovine serum (FBS) in a T25 flask. A mixture of CMV-driven BRD9(E135-A252)-SmBiT plasmid (2.5 µg), LgBiT-CRBN plasmid (2.5 µg), DDB1 plasmid (5 µg), OptiMEM I (500 µL, Gibco 31985070), and FuGENE HD (30 µL, Promega E2312) was incubated for 10 min at room temperature before being added to the cells. After 24 h of incubation at 37 °C, 5% CO_2, the transfected cells were trypsinized and normalized to 0.50 × 10^6 cells/mL in DMEM (supplemented with 10% FBS). MLN4924 (in DMSO, final concentration 2 µM) was then added to the cells. The cells were plated into a 1536-well TC-treated white plate (Greiner 782073) at 5 µL (approximately 2500 cells) per well using a single tip dispenser (GNF Systems One Tip Dispenser) and incubated for 16 h at 37 °C, 5% CO_2. Compounds were added from serially diluted DMSO stocks (20 nL per well, final top concentration 30 µM) using an Echo 655 acoustic liquid handler. For BRD9 ligand competition experiments, BI-7273 (2.5 nL of 5 mM DMSO stock, final 2.5 µM) was added at the same time as the compound treatment using the Echo 655 acoustic liquid handler. After 2h of incubation at 37 °C, 5% CO_2, plates were removed from the incubator and cooled at room temperature for 15 minutes. Nano-Glo reagent (Promega N2012, Nano-Glo substrate diluted 1:25 in a 1:1 mixture of Nano-Glo LCS buffer and phosphate-buffered saline) was added at 5 µL per well using the GNF single tip dispenser. After an additional 15 min incubation at room temperature, luminescence signals were measured on a PHERAstar FS plate reader (BMG Labtech). Luminescence values for each compound-treated sample were normalized using the equation (well value-NC)/NC × 100%, where NC represents the average neutral control (DMSO) well value. Data were analyzed using GraphPad Prism v9.

HiBiT assay

HEK293T cells (2.5 mL) were plated at 0.625 × 10^6 cells/mL in DMEM (Gibco 11995065) supplemented with 10% FBS in a 6-well TC-treated plate (Corning 3516). Plasmids of HSVtk-driven BRD9 (M1-T597) fused with a HiBiT tag at either N- or C-terminal (2.5 µg), SmBiT-CRBN plasmid (2.5 µg), OptiMEM I (250 µL, Gibco 31985070), and FuGENE HD (15 µL, Promega E2312) were incubated for 10 min at room temperature before being added to the cells. After 24 h of incubation at 37 °C, 5% CO_2, the transfected cells were trypsinized and normalized to 0.50 × 10^6 cells/mL in DMEM (supplemented with 10% FBS). The cells were plated into a 1536-well TC-treated white plate (Greiner 782073) at 5 µL (approximately 2500 cells) per well using a single tip dispenser (GNF Systems One Tip Dispenser) and incubated for 8 h at 37 °C, 5% CO_2. Compounds were dispensed from serially diluted DMSO stocks (20 nL per well, final top concentration 30 µM) using an Echo 655 acoustic liquid handler. After 15 h of incubation at 37 °C, 5% CO_2, the plates were removed from the incubator and cooled at room temperature for 15 minutes. Nano-Glo HiBiT lytic reagent (Promega N3040, LgBiT Protein and Lytic Substrate diluted at 1:100 and 1:50, respectively, in Lytic Buffer) was added at 5 µL per well using the GNF single tip dispenser. After an additional 15 min incubation at room temperature, luminescence signals were measured on a PHERAstar FS plate reader (BMG Labtech) and normalized to DMSO-treated samples. Data were analyzed using GraphPad Prism v9.

CellTiter-Glo cell viability assay

SYO-1 cells were cultured in DMEM (Gibco 11995065) supplemented with 10% FBS. HCT116 cells were cultured in RPMI with GlutaMAX (Gibco 61870036) supplemented with 10% FBS. Cells were normalized to 6.2 × 10^3 cells/mL and plated at 40 µL (approximately 250 cells) per well in white 384-well cell culture plates (Greiner 781080) using a single tip dispenser (GNF Systems One Tip Dispenser). Compounds were dispensed from serially diluted DMSO stocks (100 nL per well, final top concentration 25 µM) using an Echo 655 acoustic liquid handler. The plates were mixed at 1500 rpm for 30 seconds on a plate shaker (Eppendorf) and incubated at 37 °C, 5% CO2 for 7 days. After incubation, plates were removed from the incubator and allowed to cool down at room temperature for 15 minutes. CellTiter-Glo reagent (30 µL, Promega G7571) was dispensed into each well using the GNF single tip dispenser and the plates were incubated at room temperature for an additional 15 minutes before luminescence was measured on a PHERAstar FS plate reader (BMG Labtech). Luminescence signals were normalized to DMSO-treated wells and analyzed using GraphPad Prism v9.

RNA-Seq

SYO-1 cells were seeded in 6-well culture plates at a density of 0.2 million cells per well in 2 mL of DMEM (Gibco, 11995065) supplemented with 10% FBS. After 24 h of incubation at 37 °C and 5% CO_2, 1.0 µL of compound B67b stock solution (10 mM in DMSO; 5 µM final concentration) or a DMSO vehicle control was added to the cells. Cells were then incubated for another 24 h at 37 °C and 5% CO_2. Following treatment, the cells were trypsinized, pelleted by centrifugation (5 min at 300 x g), and washed with cold PBS. The resulting cells pellets were lysed in 150 µL DNA/RNA Shield (Zymo Research, R1100). RNA sequencing and subsequent data analysis was performed by Plasmidsaurus.

Computational modeling of ternary complex structures

Boltz-2 Structure Prediction. Initial coordinates for the CRBN-ligand-BRD9 ternary complexes (incorporating four stereoisomers) were generated using Boltz-2. The CRBN (residues N48–I426) and BRD9 (residues E135–A242) constructs were input as FASTA sequences. The small-molecule ligands were supplied in SDF format with explicit hydrogens. All other structural prediction parameters were maintained at their default settings.

System Preparation and Parameterization. The protein components of the Boltz-2 predicted structures were prepared and parameterized using PDBFixer to add missing atoms and hydrogens at pH 7.0, followed by the application of the AMBER ff14SB force field. Ligand topologies and parameters were generated utilizing the OpenFF 2.0.0 (Sage) small molecule force field via the Open Force Field Toolkit. Each ternary complex was solvated in a dodecahedron box of TIP3P water molecules with a minimum buffer distance of 10.0 Å (1.0 nm) from the solute. The systems were neutralized and set to a physiological ionic strength of 0.15 M NaCl using the OpenMM SystemGenerator framework. Additionally, hydrogen mass repartitioning (to 4.0 amu) was applied to the system to permit a 4 fs integration time step during production molecular dynamics.

MD Simulations. All energy minimization, equilibration, and production molecular dynamics simulations were performed using the OpenMM toolkit. Initial energy minimization was performed to remove steric clashes and relax the solvent. Initial velocities were directly assigned corresponding to a temperature of 300 K, followed by 1 ns of equilibration. Temperature regulation was achieved using a Langevin middle integrator with a collision frequency of 1.0 ps^{-1} under the NVT ensemble. Production MD simulations were executed for 50 ns per isomer. A time step of 4.0 fs was employed, enabled by constraining all bonds involving hydrogen atoms and applying hydrogen mass repartitioning (4.0 amu) to the system. Long-range electrostatics were treated using the Particle Mesh Ewald (PME) method, and a non-bonded cutoff of 10.0 Å was applied. Trajectory frames were saved every 100 ps for subsequent analysis

Trajectory Analysis and Structural Dynamics. Trajectory processing and geometric analyses were performed using cpptraj (AmberTools). To evaluate interfacial stability, the Root Mean Square Deviation (RMSD) of the BRD9 Cα backbone was calculated relative to the initial frame following alignment of the CRBN backbone. Intermolecular interactions were quantified by calculating fractional occupancies. Hydrogen bonds were defined by standard geometric criteria (donor-acceptor distance ≤ 3.0 Å). Salt bridges were identified using a ≤ 4.5 Å distance cutoff between acidic (Asp, Glu) and basic (Arg, Lys, His) side-chain heavy atoms. Aromatic (π-π) stacking interactions were captured using a ≤ 5.0 Å distance cutoff between aromatic rings.

MM/GBSA Thermodynamic Analysis. Relative binding free energies (ΔG) were calculated using the MM/GBSA method via MMPBSA.py on a stable, solvent-stripped subset of the production trajectory. Configurational entropy contributions were omitted; consequently, the resulting ΔG values serve exclusively as relative energetic rankings to evaluate the thermodynamic preference of the stereoisomers.

Structural Clustering and Visualization. To identify a representative structural conformation, K-means clustering was applied to the Cα backbone of the ternary complexes using cpptraj. The centroid frame of the most populated cluster was extracted to map the hydrogen bond networks and interfacial salt bridges. All structural figures were rendered using PyMOL.

4. General synthetic information

4.1 General information for off-DNA synthesis

Unless otherwise noted, all reactions were performed in glass vials capped with silicon septa. Anhydrous tetrahydrofuran (THF), dichloroemthane (DCM), *N,N*-dimethylformamide (DMF), dimethyl sulfoxide (DMSO), methanol (MeOH), triethylamine (Et_3N), and *N*,*N*-Diisopropylethylamine (DIPEA) were purchased from Sigma Aldrich (with Sure/Seal™) and used as received. Other chemicals were purchased from various suppliers and used as received.

Thin layer chromatography (TLC) was performed using silica gel 60 F_{254} glass plates (EMD Millipore) and visualized with UV light and/or staining with a solution of $KMnO_4$. Column chromatography was performed using Teledyne ISCO CombiFlash systems with RediSep Rf columns.

Ultra-performance liquid chromatography – mass spectrometry (UPLC-MS) was performed using Waters ACQUITY systems equipped with ACQUITY UPLC BEH C18 1.7 µM, 2.1 x 30 mm columns kept at 50°C. UV signals were monitored with ACQUITY UPLC PDA detectors (210 – 400 nm) and light scattering signals were recorded with ACQUITY ELSD detectors. Acidic gradients consisted of water (solvent A) and acetonitrile (solvent B) mobile phases both modified with 0.1% formic acid and basic gradients consisted of the same solvents modified with 5 mM ammonium hydroxide. Linear mobile phase gradients ran from 2% to 98% solvent B over a runtime of 2 mins and at a flowrate of 1.0 mL/min. High-resolution mass spectra (HRMS) were obtained with a Waters Xevo G2 QToF detector using positive mode electron spray ionization (ESI) and scan times of 0.2 sec.

Preparative high-performance liquid chromatography (prep-HPLC) was performed on a Teledyne ACCQPrep HP150 system equipped with UV (214 nm and 254 nm) and Evaporative Light Scattering (ELS) detectors. Separations were performed on a 30 mm x 50 mm, 5 µm column using focused gradients of water (solvent A) and acetonitrile (solvent B) modified with 0.1% formic acid or 10 mM ammonium hydroxide (75 mL/min flowrate).

NMR Spectra were recorded on Bruker spectrometers with ^{1}H-NMR recorded at 400 MHz or 600 MHz and ^{13}C-NMR at 101 MHz or 151 MHz. Data were acquired at 25 °C using the software ICON-NMR under TopSpin program control and processed with MestReNova. ^{1}H-NMR and ^{13}C-NMR spectra were referenced to residual solvent resonance (ppm): $CDCl_3$ 7.26/77.16; d_6-DMSO 2.50/39.52; MeOD 3.31/49.00 ; CD_2Cl_2 5.32/53.84. Chemical shifts are expressed in ppm and referenced to residual solvent peaks with multiplicities reported as: s = singlet, d = doublet, t = triplet, q = quartet, m = multiplet, and br = broad signal. Coupling constants are reported in Hertz (Hz).

4.2 General information for on-DNA synthesis

Unless otherwise noted, all chemicals, purchased from various sources, were used as received without further purification. Nuclease-free water (Invitrogen AM9932) was used for dissolving DNA and preparing buffers. All on-DNA reactions were performed in DNase-free polypropylene plastics (PCR strip tubes, microcentrifuge tubes, conical tubes or 96-well plates, depending on the reaction scale).

DNA headpiece (AOP-HP) and forward primer sequences were ordered from LGC Biosearch Technologies as lyophilized powders. All cycle tag duplexes (DNA barcodes) were ordered from LGC Biosearch Technologies as pre-annealed, 1 mM solution in water. Library closing sequences were ordered from Integrated DNA Technologies.

The general EtOH precipitation protocol is as follows: To a reaction of a given volume, aq. NaCl (5 M, 5% v/v) and EtOH (500% v/v, unless otherwise noted) were added. The reaction vessel was briefly vortexed and incubated on dry ice for 30 to 60 min. After centrifugation (10 min at 17500 x g or 40 min at 3750 x g), the supernatant was removed by pipetting or decanting into another vessel. For 96-well plate, the supernatants were removed by covering the plate with layers of paper towels and flipping the plate. To wash the pellet, 80% aq. EtOH (300% v/v, unless otherwise noted) was added. The mixture was vortexed for approximately 2 min and chilled again on dry ice for 30 to 60 min. After centrifugation (10 min at 17500 x g or 40 min at 3750 x g), the supernatant was removed in the same way as described above. The washing procedure was repeated with 100% EtOH (300% v/v, unless otherwise noted) and the obtained pellet was dried in air or carefully under vacuum.

On-DNA reactions were monitored by UPLC-MS with ion-pairing reagents. The UPLC-MS was performed on a Waters ACQUITY system equipped with a C18 column (ACQUITY Oligonucleotide BEH C18 1.7 µm, 2.1 × 50 mm, part #186003949) kept at 50 °C. UV signal was recorded with an ACQUITY TUV detector (260 nm, sampling rate 20 points/s). Mass spectra of eluting species were recorded with a Waters SQ Detector 2 connected to a ZSpray™ source (negative ion mode, scan time 0.2 s, scanning range 500–3000 Da). MS spectra were deconvoluted with MassLynx MaxEnt1 algorithms. Solvent A was 250 mM hexafluoroisopropanol (Oakwood Chemical) and 8 mM triethylamine (Sigma-Aldrich) in water. Solvent B was methanol. The standard method used a linear gradient ran from 5% to 50% solvent B over 4 min at 0.5 mL/min flow rate (total run time 6.0 min). Samples were diluted in water and injected at 1-10 µL.

Micro-preparative HPLC of oligonucleotide conjugates were performed on an Agilent Infinity system equipped with an Infinity 1260 Bio Quat Pump, an Infinity 1260 HiP Bio ALS autosampler, an Infinity 1260 DAD, an Infinity 1260 Bio FC-AS fraction collector, and a Waters XBridge BEH C18 OBD, 130 Å, 5 µm, 10 × 150 mm column. Material was injected as aqueous solutions (up to 100 µL or 500 µg per injection) and eluted with focused gradient of solvent A (50 mM triethylammonium acetate in water) and solvent B (MeOH). Fraction collection was triggered by UV (260 nm).

5. DEL Synthesis

5.1 Off-DNA synthesis of the strained allene precursor

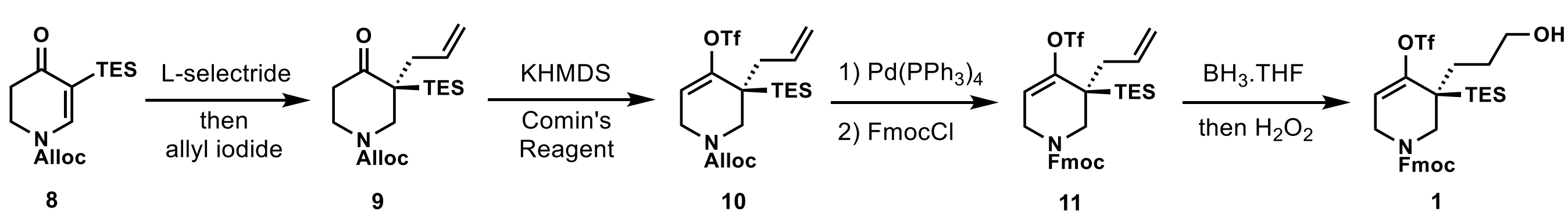

Scheme S1. Synthesis of the strained allene scaffold **1**.

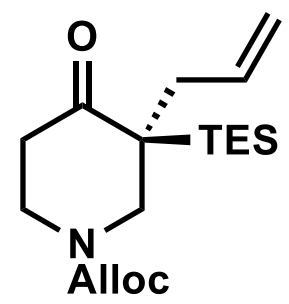

9

Compound **9**: To a 40 mL glass vial under positive pressure of nitrogen was added allyl 4-oxo-5-(triethylsilyl)-3,4-dihydropyridine-1(2H)-carboxylate (**8**)[5] (296 mg, 1.00 mmol, 1 equiv.) and THF (10 mL). The solution was cooled to -78 °C and L-Selectride (1.0 M in THF, 1.40 mL, 1.40 mmol, 1.4 equiv.) was added. After 1 h at -78 °C, DMPU (604 µL, 5.00 mmol, 5 equiv.) was added, followed by allyl iodide (550 µL, 6.00 mmol, 6 equiv.). The reaction was immediately warmed up to -30 °C, then allowed to continue to warm up slowly to -5 °C over 1 h. The reaction was quenched with saturated aq. NH_4Cl and extracted 3 times with diethyl ether. The combined organic layer was dried over Na_2SO_4 and concentrated *in vacuo*. The residue was purified by flash column chromatography (5-20% EtOAc/Heptane) to afford compound **9** (167 mg, 49%). ^{1}H NMR (400 MHz, $CDCl_3$) δ 6.01 – 5.83 (m, 1H), 5.63 (dtd, *J* = 16.3, 9.3, 5.8 Hz, 1H), 5.28 (d, *J* = 17.2 Hz, 1H), 5.19 (d, *J* = 10.3 Hz, 1H), 4.99 (d, *J* = 17.6 Hz, 1H), 4.95 (d, *J* = 9.2 Hz, 1H), 4.57 (d, *J* = 5.5 Hz, 2H), 3.98 (d, *J* = 14.1 Hz, 1H), 4.00 – 3.84 (m, 1H), 3.78 – 3.57 (m, 1H), 3.42 – 3.21 (m, 1H), 2.86 (dd, *J* = 13.9, 5.8 Hz, 1H), 2.58 (ddd, *J* = 15.4, 11.6, 7.5 Hz, 1H), 2.46 – 2.31 (m, 1H), 2.15 – 1.96 (m, 1H), 0.96 (t, *J* = 7.9 Hz, 9H), 0.68 (qd, *J* = 7.9, 4.0 Hz, 6H); ^{13}C NMR (101 MHz, $CDCl_3$) δ 211.0, 210.6, 155.0, 134.6, 133.0, 118.1, 117.8, 117.5, 66.2, 50.4, 49.9, 46.2, 42.1, 41.7, 39.8, 37.6, 7.9, 2.4; HRMS (ESI) *calcd* for $[C_{18}H_{31}NNaO_3Si]^+$ ($[M+Na]^+$): *m/z* 360.1965, found: 360.1983.

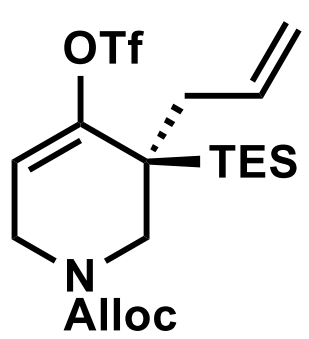

10

Compound **10**: To a 20 mL glass vial under positive pressure of nitrogen was added compound **9** (253 mg, 0.750 mmol, 1 equiv.) and THF (8.0 mL). The solution was cooled to -78 °C and KHMDS (1.0 M in THF, 1.50 mL, 1.50 mmol, 2.0 equiv.) was added dropwise. After 1 h at -78 °C, Comins' Reagent (441 mg, 1.12 mmol, 1.5 equiv.) in 0.8mL THF was added, and the mixture was stirred at -78 °C for 1 h. The reaction was then warmed up to -10 °C over 10 min before being quenched with saturated aq. NH_4Cl. The mixture was extracted 3 times with diethyl ether. The combined organic layer was dried over Na_2SO_4 and concentrated *in vacuo*. The residue was purified by flash column chromatography (5-15% EtOAc/Heptane) to afford compound **10** (233 mg, 66%). ^{1}H NMR (400 MHz, $CDCl_3$) δ 5.93 (ddt, *J* = 16.4, 10.8, 5.6 Hz, 1H), 5.77 (dddd, *J* = 16.9, 10.1, 8.4, 6.1 Hz, 1H), 5.72 – 5.62 (m, 1H), 5.30 (d, *J* = 17.2 Hz, 1H), 5.23 (d, *J* = 10.4 Hz, 1H), 5.09 (d, *J* = 16.5 Hz, 1H), 5.04 (d, *J* = 10.2 Hz, 1H), 4.67 – 4.50 (m, 2H), 4.30 – 4.12 (m, 1H), 3.90 (d, *J* = 18.0 Hz, 1H), 3.79 (d, *J* = 13.6 Hz, 1H), 3.50 (d, *J* = 13.6 Hz, 1H), 2.49 (dd, *J* = 14.5, 6.0 Hz, 1H), 2.26 (dd, *J* = 14.4, 8.4 Hz, 1H), 1.01 (t, *J* = 7.9 Hz, 9H), 0.72 (qd, *J* = 8.4, 8.0, 1.8 Hz, 6H); ^{13}C NMR (101 MHz, $CDCl_3$) δ 154.8, 153.1, 152.6, 133.5, 133.2, 132.9, 118.43, 118.42 (q, *J* = 321 Hz, *C*F3), 118.2, 117.8, 110.4, 109.8, 66.5, 47.2, 42.2, 36.8, 35.5, 8.0, 2.8; HRMS (ESI) *calcd* for $[C_{19}H_{31}F_3NO_5SSi]^+$ ($[M+H]^+$): *m/z* 470.1639, found: 470.1669.

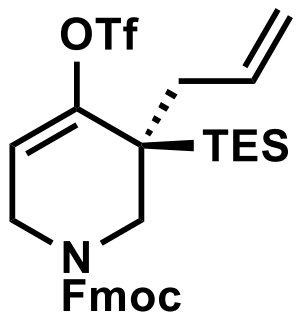

11

Compound **11**: To a 20 mL glass vial under positive pressure of nitrogen was added compound **10** (123 mg, 0.262 mmol, 1 equiv.) and DCM (4.0 mL). The solution was cooled to 0 °C and a solution of $Pd(PPh_3)_4$ (30.3 mg, 0.0262 mmol, 0.1 equiv.) and 1,3-dimethylbarbituric acid (61.3 mg, 0.393 mmol, 1.5 equiv.) in DCM (1.0 mL) was added. After 5 min, FmocCl (203 mg, 0.786 mmol, 3 equiv.) and $NaHCO_3$ (220 mg, 2.62 mmol, 10 equiv.) were added at 0 °C and the reaction was stirred at the same temperature for 1.5 h. The reaction was quenched with water and extracted 3 times with DCM. The combined organic layer was dried over Na_2SO_4 and concentrated *in vacuo*. The residue was purified by flash column chromatography (50-75% DCM/Heptane) to afford compound **11** (133 mg, 84%). ^{1}H NMR (400 MHz, $CDCl_3$) δ 7.77 (d, *J* = 7.5 Hz, 2H), 7.57 (d, *J* = 7.4 Hz, 2H), 7.41 (t, *J* = 7.5 Hz, 2H), 7.32 (t, *J* = 7.5 Hz,

2H), 5.84 – 5.67 (m, 0.6H), 5.69 (t, *J* = 3.5 Hz, 1H), 5.64 – 5.46 (m, 0.4H), 5.17 – 4.80 (m, 2H), 4.69 – 4.35 (m, 2H), 4.31 – 4.06 (m, 2H), 3.95 – 3.41 (m, 2.6 H), 3.22 – 3.04 (d, *J* = 12.8 Hz, 0.4 H), 2.55 – 2.32 (m, 1H), 2.32 – 1.98 (m, 1H), 0.99 (d, *J* = 8.1 Hz, 9H), 0.69 (dt, *J* = 34.5, 8.0 Hz, 6H); ^{13}C NMR (101 MHz, $CDCl_3$) δ 155.0, 153.1, 152.4, 144.00, 143.96, 141.5, 133.5, 133.2, 127.90, 127.86, 127.25, 127.22, 125.0, 124.8, 120.2, 118.6, 118.4 (q, *J* = 321 Hz, *C*F3), 117.9, 110.4, 109.6, 67.8, 67.0, 47.5, 47.2, 42.2, 37.1, 36.8, 35.5, 8.0, 2.8, 2.7; HRMS (ESI) *calcd* for $[C_{30}H_{36}F_3NNaO_5SSi]^+$ ($[M+Na]^+$): *m/z* 630.1928, found: 620.1948.

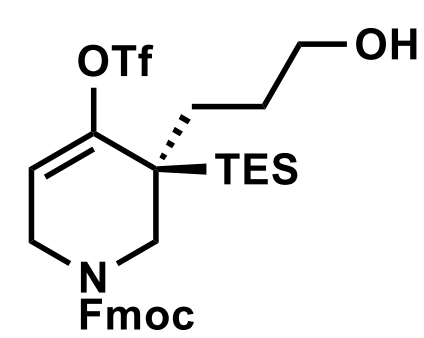

1

Compound **1**: To a 20-mL glass vial under positive pressure of nitrogen was added compound **11** (248 mg, 0.408 mmol, 1 equiv.) and THF (5.0 mL). BH_3-THF (1.0 M in THF, 1.22 mL, 1.22 mmol, 3.0 equiv.) was added dropwise with the cooling of a room temperature water bath. After 1.5 h, the reaction was cooled to 0 °C and H_2O_2 (30 wt%, 834 μL, 8.16 mmol, 20 equiv.) was added dropwise followed by NaOH (1.0 M aq., 1.22 mL, 1.22 mmol, 3.0 equiv.). The mixture was allowed to stir for another 2 h at room temperature. The reaction was then quenched with saturated aq. $Na_2S_2O_3$ at 0 °C and extracted 3 times with diethyl ether. The combined organic layer was dried over Na_2SO_4 and concentrated *in vacuo*. The residue was purified by flash column chromatography (25-40% EtOAc/Heptane) to afford compound **1** (163 mg, 64%). ^{1}H NMR (600 MHz, DMSO) δ 7.89 (d, *J* = 7.6 Hz, 2H), 7.64 (d, *J* = 7.5 Hz, 2H), 7.42 (td, *J* = 7.5, 2.8 Hz, 2H), 7.33 (td, *J* = 7.4, 2.5 Hz, 2H), 5.88 – 5.70 (m, 1H), 4.68 – 4.49 (m, 1H), 4.47 – 4.31 (m, 1H), 4.28 (t, *J* = 5.8 Hz, 1H), 4.19 – 4.03 (m, 1H), 3.98 – 3.85 (m, 0.5H), 3.75 – 3.66 (m, 0.5H), 3.66 – 3.58 (m, 0.5H), 3.54 – 3.46 (m, 0.5H), 3.28 – 3.13 (m, 2H), 3.06 – 2.95 (m, 0.5H), 1.69 – 1.06 (m, 4H), 1.00 – 0.83 (m, 9H), 0.73 – 0.46 (m, 6H); ^{13}C NMR (151 MHz, DMSO) δ 154.3, 154.1, 152.5, 152.0, 143.8, 140.8, 127.7, 127.1, 125.0, 124.6, 120.1, 117.8 (q, *J* = 320 Hz, *C*F3), 111.4, 111.2, 67.0, 66.1, 61.1, 46.7, 41.8, 34.2, 28.6, 27.6, 7.7, 2.1, 1.9. HRMS (ESI) *calcd* for $[C_{30}H_{39}F_3NO_6SSi]^+$ ($[M+H]^+$): *m/z* 626.2214, found: 626.2225.

5.2 Attachment of strained allene scaffold onto DNA headpiece

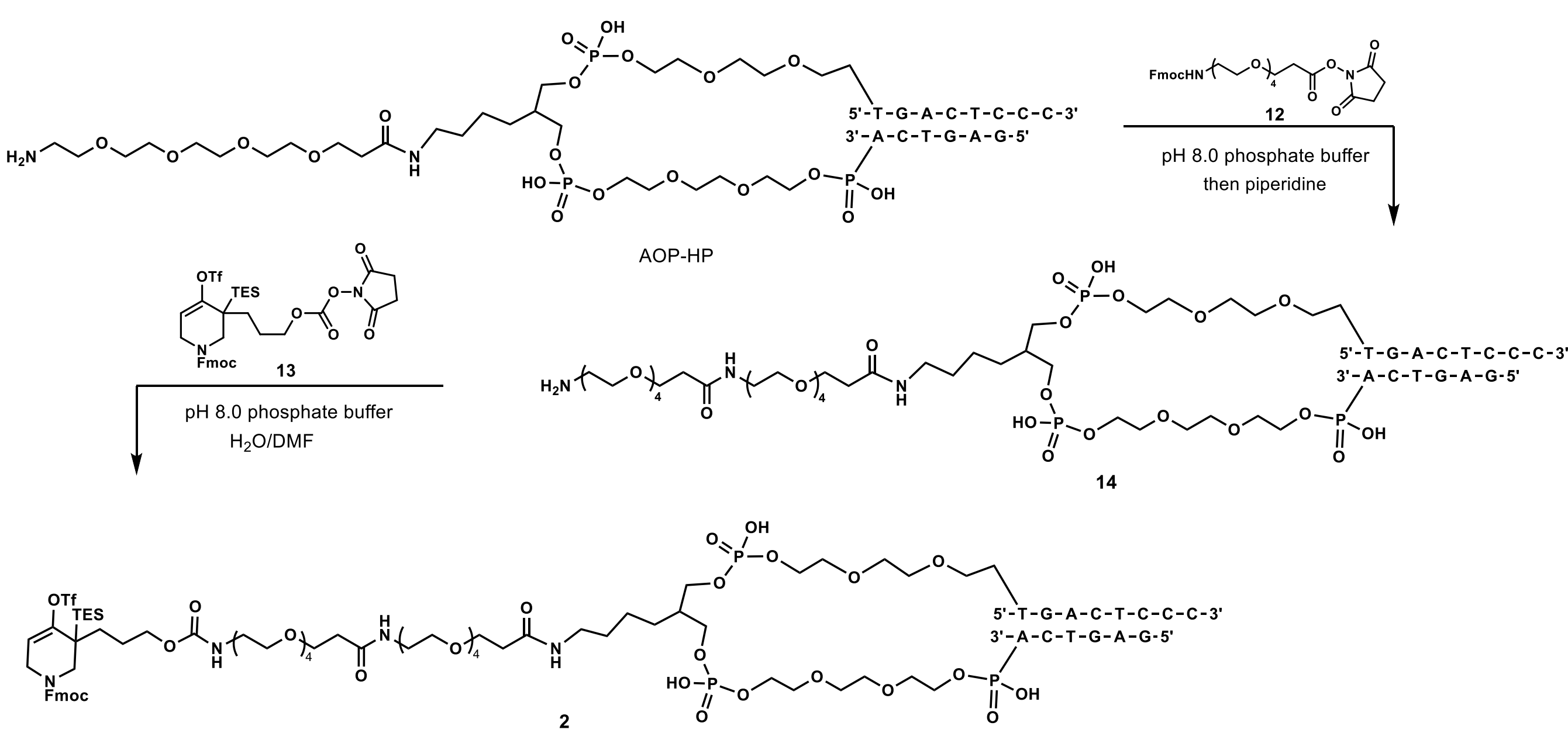

Scheme S2. Attachment of strained allene scaffold **1** onto DNA headpiece

DNA conjugate **14**: To a 15 mL conical tube containing AOP-HP (1.0 mM in pH 8.0, 0.1M phosphate buffer) (2.00 mL, 2.00 µmol, 1 equiv.) was added the NHS ester **12** (100 mM in DMF, 600µL, 60 µmol, 30 equiv.). The tube was vortexed until the mixture became mostly clear. The reaction was incubated at room temperature for 1 h when LCMS indicated the complete conversion. The product was isolated by EtOH precipitation following the general protocol (see Section 4.2). The pellet was re-dissolved in 2.0 mL nuclease-free water and piperidine (50% v/v in water, 500 µL) was added. The mixture was vortexed and incubated at room temperature for another 20 min. The product was then precipitated with EtOH following the general protocol. The pellet was air-dried and re-dissolved in pH 8.0 phosphate buffer to a final volume of 735 µL and quantified with Nanodrop (2.2 µmol, 110% yield). MS (ESI-) expected 5431Da, observed 5430 Da.

NHS carbonate **13**: To a 1-dram vial was added sequentially compound **1** (14.3 mg, 0.0229 mmol, 1 equiv.), N.N'-disuccinimidyl carbonate (17.6 mg, 0.0686 mmol, 3.0 equiv.), DCM (0.5 mL) and Et_3N (16µL, 0.11 mmol, 5.0 equiv.). The reaction was allowed to stir at room temperature for 12 h. The crude reaction mixture was directly loaded onto a short silica column and quickly eluted with 30-50% EtOAc/Heptane (the product will hydrolyze slowly on silica gel). The fractions were concentrated *in vacuo* to yield NHS carbonate **13** (12.6mg, 72%), which was directly used for DNA attachment reactions.

DNA conjugate **2**: To a 15 mL conical tube was added the DNA conjugate **14** (2.2 µmol in 735µL pH 8.0 phosphate buffer) and the NHS carbonate **13** (11.0 mg, 14.3 µmol, 6.5 equiv.) dissolved in 1.5 mL DMF. The mixture was vortexed, briefly spun down, and incubated at room temperature for 2 h. Some precipitate formed during the reaction, which was removed by centrifuge. EtOH precipitation was then performed to the supernatant. The resulting DNA pellet was dissolved in water and further purified by microprep-HPLC (split into 26 injections). The HPLC method used a 10-95%B gradient elution over 20 min, with solvent A being 50 mM triethylammonium acetate in water and solvent B methanol. The combined fractions were concentrated and desalted using a 3kDa MWCO ultra centrifugal filter tube (Millipore Amicon UFC9003). The product was collected and diluted to a total volume of 1.00 mL and then quantified by Nanodrop (8.6 µg/uL, 1.3 µmol, 61% yield). The product was characterized by analytical UPLC-MS: expected 6083 Da, observed 6082 Da.

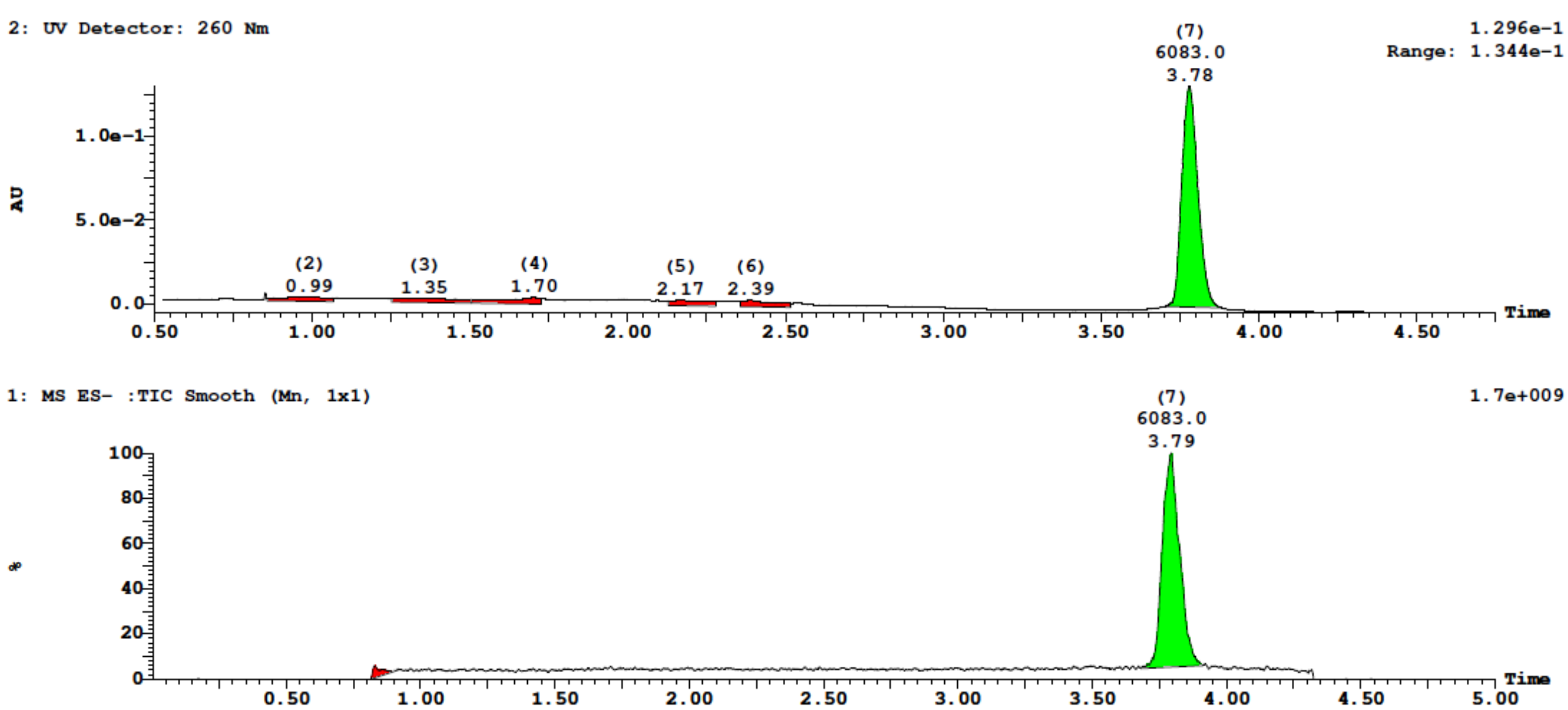

Figure S15. UPLC-MS trace of DNA conjugate **2**.

5.3 Building block validation for cycle-1 (strained allene cycloadditions)

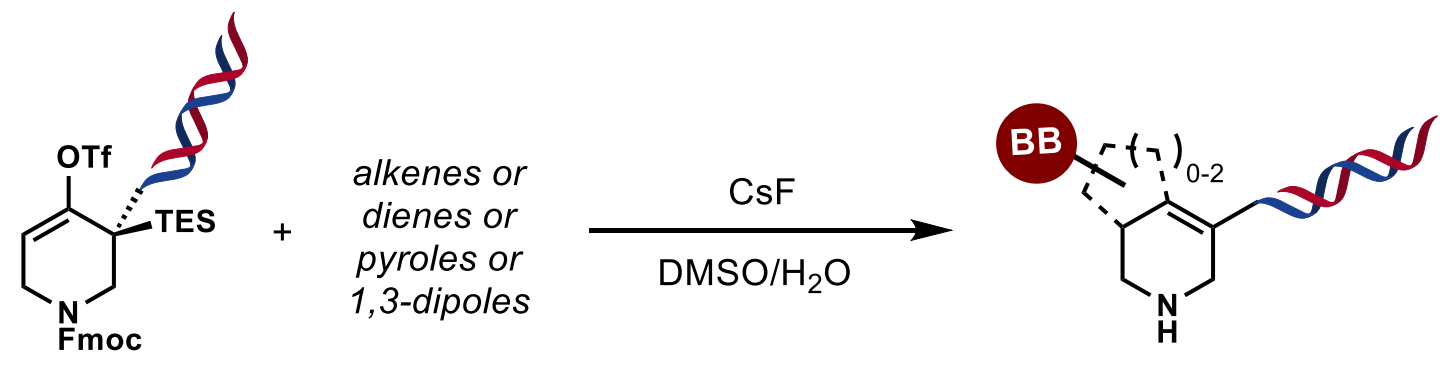

2 15

All the alkene, diene building blocks were dissolved in DMSO to make 150 mM stock solutions. For ones that did not fully dissolve, the suspensions were vortexed immediately before use. Nitrones and other 1,3-dipoles were synthesized by the condensations of aldehydes and hydroxylamine building blocks (detailed below).

General procedure for strained allene cycloaddition reaction validations:

To 96-well PCR plates was added the building block stock solution (7.2µL per well, 150 mM in DMSO, 1.1 µmol, 5000 equiv.), followed by the DNA-scaffold conjugate **2** (0.4µL per well, 0.54 mM in H_2O, 0.22 nmol, 1 equiv.). After that, a solution of CsF in pH 10 carbonate buffer (0.4µL per well, 1.0 M, 0.4 µmol, 2000 equiv.) was added using a multichannel pipette and the reaction was immediately mixed with the pipette. The plate was then heat-sealed with aluminum foil, vortexed, briefly spun down, and incubated at room temperature. After 2 h, 1.0µL of piperidine (50% v/v in water) was added to each well and the plate was incubated at room temperature for another 20 min. The reactions were precipitated by adding 120µL EtOH to each well and the DNA pellets were washed with 100µL EtOH. The resulting pellets were air-dried, dissolved in 25µL water per well, and analyzed by UPLC-MS (10µL injection).

General procedure for synthesizing 1,3-dipoles:

Hydroxylamine building blocks (0.2 M in EtOH, 6.8µL per well, 1.4 µmol) and aldehyde building blocks (0.22 M in EtOH, 6.8µL per well, 1.5 µmol) were combined in 96-well PCR plates. Each row used a different hydroxylamine, and each column used a different aldehyde. For hydroxylamines that were HCl salts, the solution was treated with triethylamine resin before use. Acetic acid (0.1 M in EtOH, 1.5µL per well) was added to the mixture. The plates were heat-sealed with aluminum foil, vortexed, briefly spun down, and incubated at 60 °C for 4.5 h in a thermocycler. After the reaction, the solvents were removed in a vacuum desiccator, and the residue was re-dissolved in 9.0µL DMSO to make (theoretical) 150 mM stock solutions of 1,3-dipoles, which were used directly in the [3+2] cycloaddition reactions described above.

Validation results:

A list of the building blocks tested and the corresponding yields (based on LCMS analysis) was provided in Appendix I, Table S2-S4. A yield cutoff of 70% is applied to determine the building blocks to be included in the library. In summary, 53 of 175 alkenes, 45 of 168 pyrroles/dienes, and 130 of 385 1,3-dipoles (228/728 total) showed satisfactory yields. Additional cycle-1 building blocks were included according to previously reported data.[4] A total of 434 cycle-1 building blocks were proceeded to actual-scale library synthesis, as described in Section 5.6.2. Full list of building blocks that were eventually included in the final DEL is provided in Appendix II, Table S6.

5.4 Building block validation for cycle-2 (amide coupling reactions)

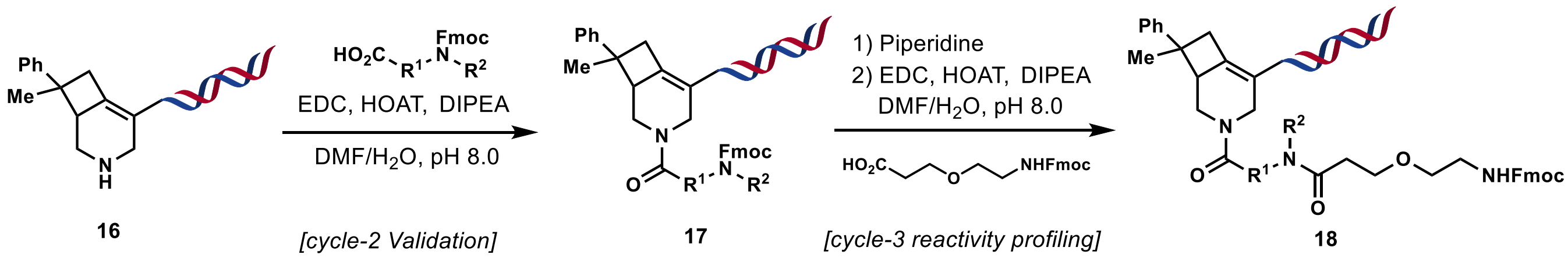

Scheme S3. Building block validation for cycle-2 amide coupling reactions.

To 96-well PCR plates were added the Fmoc-amino acid building block stock solutions (150 mM in DMF, 2.5µL per well, 0.38 µmol). A freshly prepared mixture of EDC (100 mM in DMF, 2.5µL per well, 0.25 µmol), HOAT (100 mM in DMF, 2.5µL per well, 0.25 µmol) and DIPEA (150 mM in DMF, 2.5µL per well, 0.38 µmol) were then added to each well. The plates were heat-sealed with aluminum foils, vortexed, briefly spun down, and incubated at room temperature for 30 min to 2 h (note: it was found that shorter incubate time at this step improved the product yields for certain Fmoc-amino acids; thus 30 min incubation time was employed in the actual-scale library synthesis). 2µL each of the above "activation mixture" (corresponding to 200 equiv. of activated acid) was transferred new 96-well plates, and the DNA substrate **16** (0.25 mM in pH 8.0 phosphate buffer, 1.0µL per well, 0.25 nmol, equiv.) was added to each well. The plates were heat-sealed with aluminum foils, vortexed, briefly spun down, and incubated at room temperature for 12 h. The reactions were then precipitated with 120µL EtOH per well, and the resulting pellets were re-dissolved in 0.7µL phosphate buffer and treated with 2.0µL freshly made "activation mixture" again for another 12 h, following the same procedure describe above (this second addition helped improve the convention for certain building blocks). Finally, the reactions were precipitated with 120µL EtOH, and the pellets were washed with 100µL EtOH, air-dried, and dissolved in 30µL water for UPLC-MS analysis (10µL injection).

To test whether the amino group (after Fmoc deprotection) in each cycle-2 building block would be reactive enough to undergo amide coupling reaction in cycle-3, a further amide coupling validation was performed as follows: The remaining material (DNA conjugates **17**) in the plates after UPLC-MS injections were allowed to dry in air. 10µL piperidine (10% v/v in water) was added to each well and the plates were incubated at room temperature for 30 min. EtOH precipitation was performed and the pellets were re-dissolved in 1.0µL phosphate buffer (pH 8.0). 2.0µL (corresponding to 50 nmol, or ~ 300 equiv.) of "activation mixture" made from 3-(2'-(Fmoc-amino)ethoxy)propanoic acid (representative building block) following the aforementioned protocol. The plates were sealed, vortexed, spun down, and incubated for 24 h. EtOH precipitation was then performed, and the pellets were dissolved in 15µL water for UPLC-MS analysis (10µL injection).

The validation results were provided in Appendix I, Table S5. A cycle-2 yield cutoff of 70%, combined with passing the amine reactivity test (for cycle-3), was applied to select the cycle-2 building blocks. For amine reactivity profiling, accurate yields were not determined. Instead, the presence of significant amount of the desired product in UPLC-MS is used as the criterion for including a building block in the final library. In summary, 137 of 199 Fmoc-amino acids tested were included in the final library (Appendix II, Table S6).

5.5 Cycle-3 building blocks (presenter protein-biasing elements) data

5.5.1 Amide coupling protocols

Condition a (EDC, borate buffer): To a PCR tube was added the acid building block solution (150 mM in DMF, 0.75μL, 112 nmol, 112 equiv.) and a freshly prepared mixture of EDC (120 mM in DMF, 0.75μL, 90 nmol, 90 equiv.), HOAT (120 mM in DMF, 0.75μL, 90 nmol, 90 equiv.), and DIPEA (150 mM, 0.75μL, 112 nmol, 112 equiv.). The reaction was incubated at room temperature for 20-30 min, before addition of the DNA-conjugated amine substrate (1.0 mM in pH 9.4 borate buffer, 1.0μL, 1.0 nmol, 1 equiv.). The mixture was vortexed, briefly spun down, and incubated at room temperature for 12 h. The DNA was isolated by EtOH (180μL) precipitation and dissolved in water for UPLC-MS analysis.

Condition b (EDC, phosphate buffer): The reaction protocol is the same as "condition a", except that the DNA substrate was dissolved in pH 8.0 phosphate buffer instead of pH 9.4 borate buffer.

Condition c (DMTMM, phosphate buffer): The acid building block (150 mM in DMF, 1.5μL, 225 nmol, 320 equiv.) and DMTMM (300 mM in water, 0.5μL, 150 nmol, 215 equiv.) were mixed in a PCR tube. After 10 min, additional DMF (1.5μL) was added (to keep the optimal DMF/H_2O ratio), followed by 0.5μL DNA-conjugated amine substrate (1.4 mM in pH 8.0 phosphate buffer, 0.5μL, 0.7 nmol, 1 equiv.). The reaction was vortexed, briefly spun down, and incubated at room temperature for 2 h. The DNA was isolated by EtOH (180μL) precipitation and dissolved in water for UPLC-MS analysis.

5.5.2 Validation yields

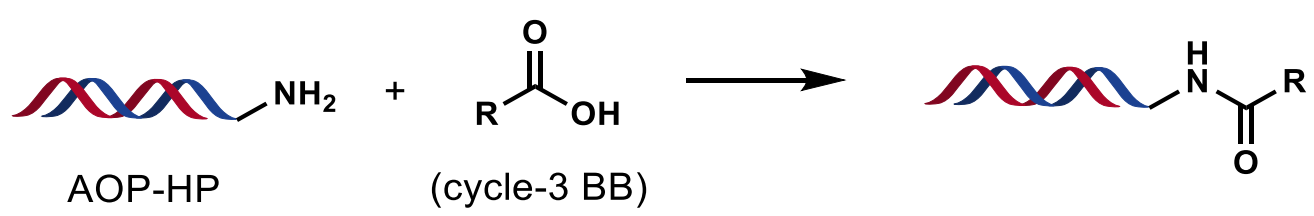

Table S1. Validation yields of cycle-3 presenter-binding ligands

Library	Cycle ID	Structure (SMILE)	Condition	Yield (%)
CRBN CIP-DEL	1	O=C1N(C(CCC(N2)=O)C2=O)C(c3c1c(OCC(O)=O)ccc3)=O	a	97
	2	O=C1N(C(CCC(N2)=O)C2=O)C(c3c1c(C(O)=O)ccc3)=O	b	93
	3	O=C1N(C(CCC(N2)=O)C2=O)C(c3c1cc(C(O)=O)cc3)=O	b	95
	4	O=C1N(C(CCC(N2)=O)C2=O)Cc3c1cc(OCC(O)=O)cc3	a	84
	5	O=C1N(c2c(OC)ccc(C(O)=O)c2)CCC(N1)=O	b	89
	6	O=C1N(c2cccc(C(O)=O)c2)CCC(N1)=O	b	93
	7	O=C1N(c2ccc(OCC(O)=O)cc2)CCC(N1)=O	c	97
	8	O=C1N(c2c(C)ccc(OCC(O)=O)c2)CCC(N1)=O	c	96
VHL CIP-DEL	1	O=C([C@H](C(C)(C)C)NC(CCC(O)=O)=O)N1C[C@H](O)C[C@H]1C(NCc2ccc(c3c(C)ncs3)cc2)=O	b	87
	2	O=C([C@H](C(C)(C)C)NC(CCC(O)=O)=O)N1C[C@H](O)C[C@H]1C(N[C@@H](C)c2ccc(c3c(C)ncs3)cc2)=O	b	90
	3	O=C([C@H](C(C)(C)C)NC(C1(F)CC1)=O)N2C[C@H](O)C[C@H]2C(NCc3ccc(c4c(C)ncs4)cc3OCC(O)=O)=O	b	89
FKBP CIP-DEL	1	O=C(C(c1cccc(OCC(O)=O)c1)=O)N2CCCC[C@H]2C(OCCCc3ccc(OC)c(OC)c3)=O	b	95
	2	O=C(C(c1cccc(OCC(O)=O)c1)=O)N2CCCC[C@H]2C(O[C@@H](c3ccccc3)CCc4ccc(OC)c(OC)c4)=O	b	99
	3	O=C(C(C(CC)(C)C)=O)N1CCCC[C@H]1C(OCCCc2ccc(OCC(O)=O)cc2)=O	b	95

	4	O=C(C(C(CC)(C)C)=O)N1CCCC[C@H]1C(O[C@@H](c2ccccc2)CCc3ccc(OCC(O)=O)cc3)=O	b	98
	5	OC([C@H](NC([C@H]1N(C(C(C(CC)(C)C)=O)=O)CCCC1)=O)CCc2ccc(OC)cc2)=O	b	75
	6	O=C(C(C(CC)(C)C)=O)N1CCCC[C@H]1C(O[C@@H](c2cc(OCC(O)=O)ccc2)CCc3ccc(OC)c(OC)c3)=O	b	99

5.5.3 Compound data

All the ligands were obtained from various vendors or from in-house collections. All structures were confirmed with NMRs before use.

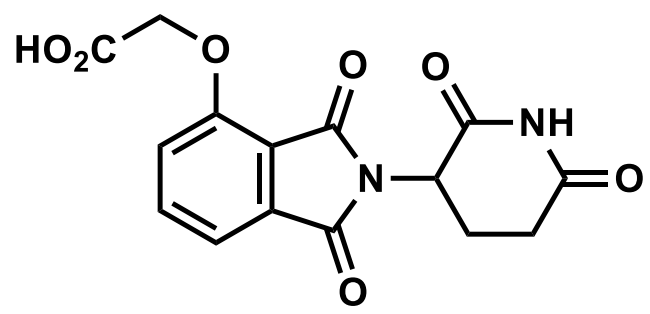

CRBN-L1: ^{1}H NMR (400 MHz, DMSO) δ 13.25 (brs, 1H), 11.11 (s, 1H), 7.79 (dd, *J* = 8.5, 7.2 Hz, 1H), 7.47 (d, *J* = 7.1 Hz, 1H), 7.39 (d, *J* = 8.6 Hz, 1H), 5.10 (dd, *J* = 12.8, 5.4 Hz, 1H), 4.98 (s, 2H), 2.89 (ddd, *J* = 17.3, 14.0, 5.5 Hz, 1H), 2.65 – 2.53 (m, 2H), 2.11 – 1.98 (m, 1H); ^{13}C NMR (101 MHz, DMSO) δ 172.8, 169.9, 169.5, 166.7, 165.2, 155.2, 136.8, 133.2, 119.9, 116.3, 115.7, 65.0, 48.8, 31.0, 22.0.

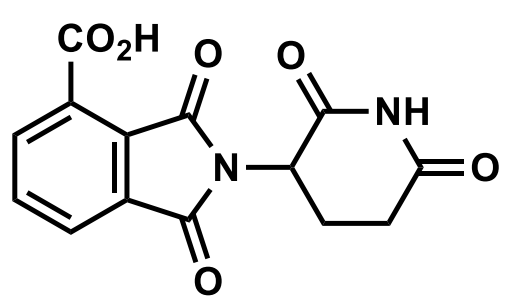

CRBN-L2: ^{1}H NMR (400 MHz, DMSO) δ 13.74 (brs, 1H), 11.14 (s, 1H), 8.04 (dd, *J* = 7.2, 1.4 Hz, 1H), 8.02 – 7.92 (m, 2H), 5.16 (dd, *J* = 12.7, 5.4 Hz, 1H), 2.89 (ddd, *J* = 16.6, 13.7, 5.3 Hz, 1H), 2.66 – 2.51 (m, 2H), 2.16 – 2.00 (m, 1H); ^{13}C NMR (101 MHz, DMSO) δ 172.8, 169.8, 166.6, 166.3, 165.2, 135.1, 133.4, 131.9, 131.4, 127.5, 125.0, 49.1, 30.9, 21.9.

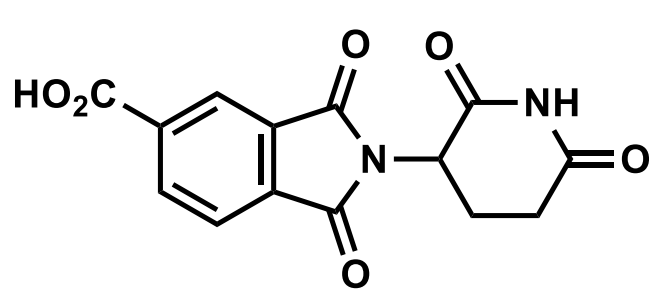

CRBN-L3: ^{1}H NMR (400 MHz, DMSO) δ 13.78 (brs, 1H), 11.16 (s, 1H), 8.40 (dd, *J* = 7.7, 1.4 Hz, 1H), 8.33 – 8.23 (m, 1H), 8.05 (d, *J* = 7.8 Hz, 1H), 5.20 (dd, *J* = 12.8, 5.4 Hz, 1H), 2.90 (ddd, *J* = 16.5, 13.6, 5.2 Hz, 1H), 2.66 – 2.52 (m, 2H), 2.17 – 2.00 (m, 1H); ^{13}C NMR (101 MHz, DMSO) δ 172.7, 169.7, 166.40, 166.36, 165.7, 136.7, 135.7, 134.5, 131.7, 123.9, 123.4, 49.2, 30.9, 21.9.

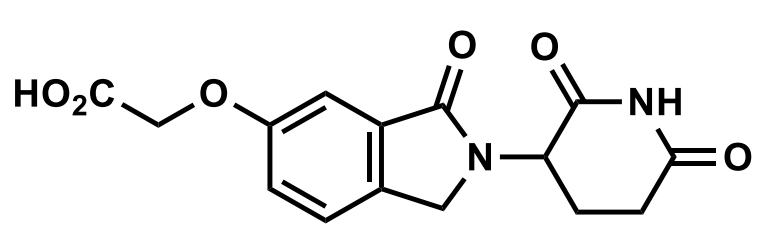

CRBN-L4: ^{1}H NMR (400 MHz, DMSO) δ 13.05 (brs, 1H), 10.98 (s, 1H), 7.51 (d, *J* = 8.3 Hz, 1H), 7.21 (dd, *J* = 8.3, 2.5 Hz, 1H), 7.16 (d, *J* = 2.5 Hz, 1H), 5.11 (dd, *J* = 13.3, 5.1 Hz, 1H), 4.79 (s, 2H), 4.38 (d, *J* = 17.0 Hz, 1H), 4.25 (d, *J* = 16.9 Hz, 1H), 2.91 (ddd, *J* = 17.2, 13.6, 5.4 Hz, 1H), 2.59 (ddt, *J* = 17.4, 4.1, 2.1 Hz, 1H), 2.39 (qd, *J* = 13.3, 4.5 Hz, 1H), 2.00 (dtd, *J* = 12.7, 5.3, 2.2 Hz, 1H); ^{13}C NMR (101 MHz, DMSO) δ 172.9, 171.0, 170.1, 167.9, 158.0, 134.6, 132.9, 124.6, 120.0, 106.9, 64.8, 51.7, 46.8, 31.2, 22.5.

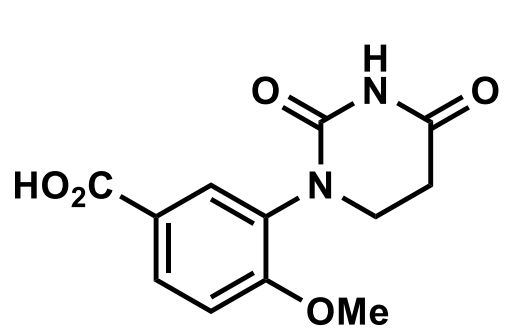

CRBN-L5: ^{1}H NMR (400 MHz, DMSO) δ 12.77 (brs, 1H), 10.35 (s, 1H), 7.91 (dd, *J* = 8.6, 2.3 Hz, 1H), 7.82 (d, *J* = 2.2 Hz, 1H), 7.21 (d, *J* = 8.8 Hz, 1H), 3.88 (s, 3H), 3.60 (t, *J* = 6.6 Hz, 2H), 2.70 (t, *J* = 6.7 Hz, 2H); ^{13}C NMR (101 MHz, DMSO) δ 170.8, 166.6, 158.5, 152.2, 130.6, 130.5, 129.8, 123.0, 112.1, 56.2, 44.2, 31.1.

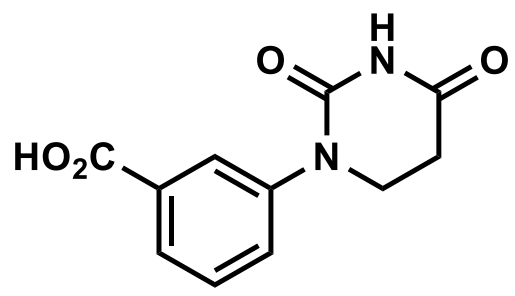

CRBN-L6: ^{1}H NMR (400 MHz, DMSO) δ 13.08 (brs, 1H), 10.43 (s, 1H), 7.89 (t, *J* = 1.9 Hz, 1H), 7.79 (dt, *J* = 7.6, 1.5 Hz, 1H), 7.58 (ddd, *J* = 8.0, 2.3, 1.3 Hz, 1H), 7.52 (t, *J* = 7.8 Hz, 1H), 3.84 (t, *J* = 6.6 Hz, 2H), 2.72 (t, *J* = 6.6 Hz, 2H); ^{13}C NMR (101 MHz, DMSO) δ 170.6, 166.9, 152.2, 142.2, 131.3, 129.5, 128.9, 126.5, 126.0, 44.3, 31.0.

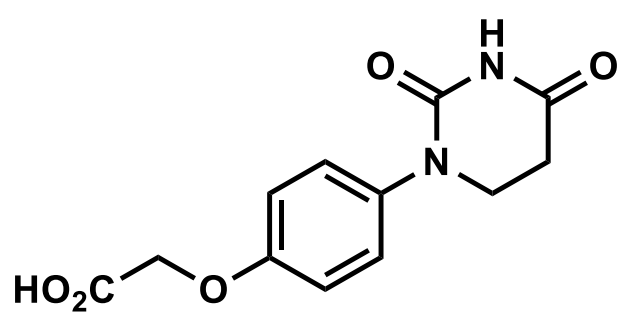

CBRN-L7: ^{1}H NMR (400 MHz, DMSO) δ 13.01 (brs, 1H), 10.30 (s, 1H), 7.26 – 7.20 (m, 2H), 6.95 – 6.89 (m, 2H), 4.67 (s, 2H), 3.71 (t, *J* = 6.7 Hz, 2H), 2.69 (t, *J* = 6.7 Hz, 2H); ^{13}C NMR (101 MHz, DMSO) δ 170.6, 170.2, 155.7, 152.3, 135.3, 126.8, 114.4, 64.6, 44.9, 31.1.

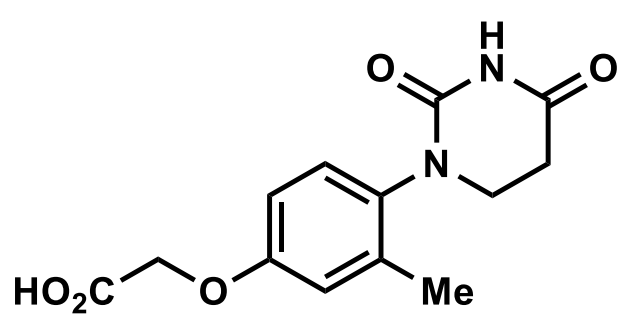

CRBN-L8: ^{1}H NMR (400 MHz, DMSO) δ 12.97 (brs, 0.2H), 10.32 (s, 1H), 7.16 (d, *J* = 8.4 Hz, 1H), 6.88 (d, *J* = 2.7 Hz, 1H), 6.79 (dd, *J* = 8.4, 2.6 Hz, 1H), 4.63 (s, 2H), 3.76 (ddd, *J* = 12.2, 9.0, 5.4 Hz, 1H), 3.53 – 3.44 (m, 1H), 2.79 – 2.63 (m, 2H), 2.09 (s, 3H); ^{13}C NMR (101 MHz, DMSO) δ 170.8, 170.1, 156.5, 151.6, 141.5, 131.0, 127.8, 113.6, 113.3, 64.7, 44.5, 31.1, 16.6.

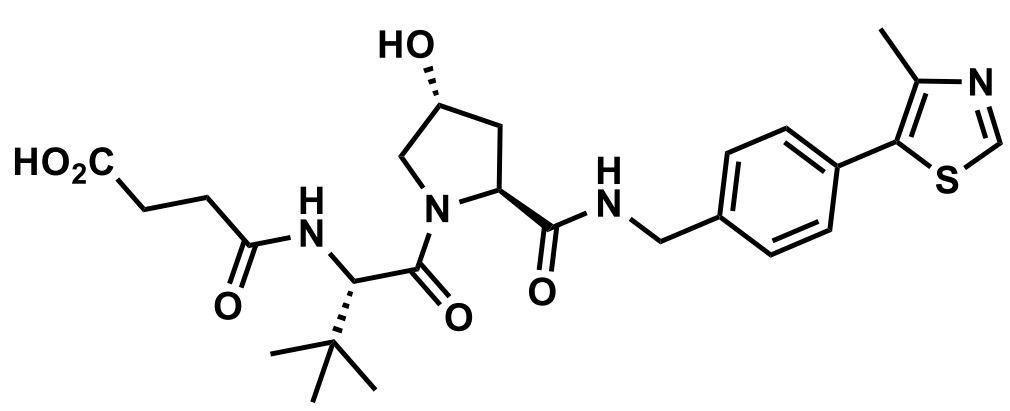

VHL-L1: ^{1}H NMR (400 MHz, DMSO) δ 12.04 (brs, 1H), 8.98 (s, 1H), 8.57 (t, *J* = 6.1 Hz, 1H), 7.94 (d, *J* = 9.3 Hz, 1H), 7.49 – 7.32 (m, 4H), 5.12 (brs, 1H), 4.53 (d, *J* = 9.3 Hz, 1H), 4.48 – 4.39 (m, 2H), 4.38 – 4.32 (m, 1H), 4.21 (dd, *J* = 15.4, 5.0 Hz, 1H), 3.70 – 3.59 (m, 2H), 2.58 – 2.32 (m, 4H), 2.44 (s, 3H), 2.02 (td, *J* = 9.5, 7.7, 5.2 Hz, 1H), 1.90 (ddd, *J* = 12.9, 8.6, 4.6 Hz, 1H), 0.93 (s, 9H); ^{13}C NMR (101 MHz, DMSO) δ 173.8, 171.9, 170.9, 169.6, 151.5, 147.7, 139.5, 131.2, 129.6, 128.6, 127.4, 68.9, 58.7, 56.43, 56.36, 41.6, 37.9, 35.4, 29.7, 29.2, 26.4, 16.0.

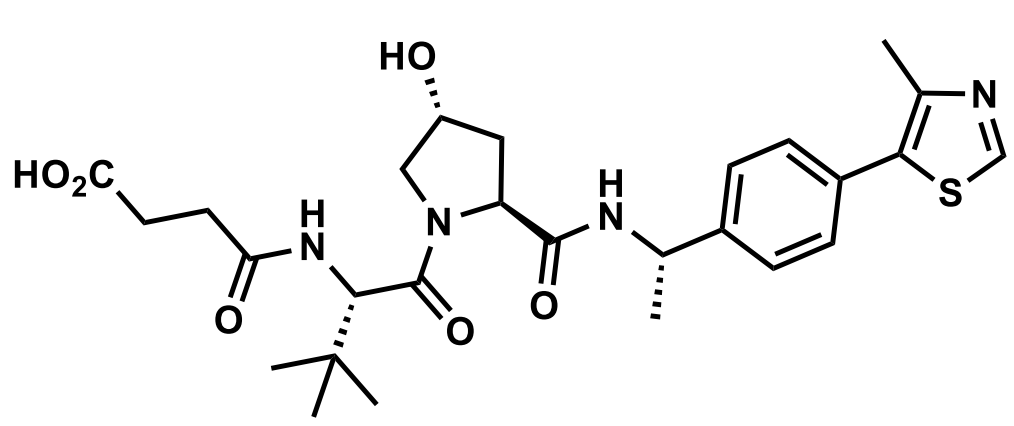

VHL-L2: ^{1}H NMR (400 MHz, DMSO) δ 12.03 (brs, 1H), 8.98 (s, 1H), 8.38 (d, *J* = 7.8 Hz, 1H), 7.88 (d, *J* = 9.3 Hz, 1H), 7.53 – 7.34 (m, 4H), 5.10 (brs, 1H), 4.91 (p, *J* = 6.9 Hz, 1H), 4.50 (d, *J* = 9.3 Hz, 1H), 4.42 (t, *J* = 8.0 Hz, 1H), 4.32 – 4.24 (m, 1H), 3.65 – 3.53 (m, 2H), 2.54 – 2.47 (m, 1H), 2.45 (s, 3H), 2.44 – 2.31 (m, 3H), 2.08 – 1.96 (m, 1H), 1.78 (ddd, *J* = 13.0, 8.5, 4.7 Hz, 1H), 1.37 (d, *J* = 7.0 Hz, 3H), 0.93 (s, 9H); ^{13}C NMR (101 MHz, DMSO) δ 173.9, 170.8, 170.6, 169.4, 151.5, 147.8, 144.7, 131.1, 129.7, 128.8, 126.4, 68.8, 58.6, 56.5, 56.2, 47.7, 37.7, 35.3, 29.7, 29.2, 26.4, 22.4, 16.0.

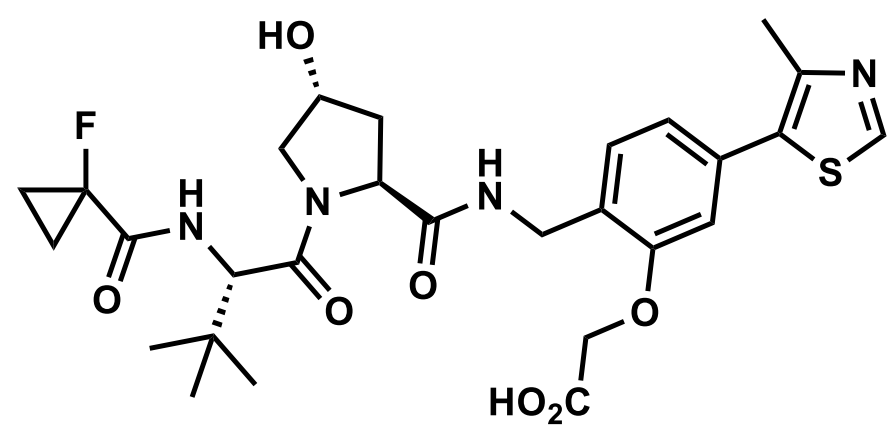

VHL-L3: ^{1}H NMR (400 MHz, DMSO) δ 13.10 (brs, 1H), 8.98 (d, *J* = 2.6 Hz, 1H), 8.53 (td, *J* = 6.1, 2.5 Hz, 1H), 7.43 (dd, *J* = 7.8, 2.5 Hz, 1H), 7.29 (dt, *J* = 9.5, 2.8 Hz, 1H), 7.01 – 6.91 (m, 2H), 4.81 (d, *J* = 2.6 Hz, 2H), 4.60 (dd, *J* = 9.3, 2.4 Hz, 1H), 4.51 (td, *J* = 8.3, 2.5 Hz, 1H), 4.42 – 4.21 (m, 3H), 3.71 – 3.57 (m, 2H), 2.44 (d, *J* = 2.6 Hz, 3H), 2.09 (dd, *J* = 12.7, 8.0 Hz, 1H), 1.98 – 1.86 (m, 1H), 1.44 – 1.29 (m, 2H), 1.27 – 1.16 (m, 2H), 0.96 (s, 9H); ^{13}C NMR (101 MHz, DMSO) δ 171.9, 170.3, 168.9, 168.2, 168.0, 155.0, 151.5, 147.9, 131.1, 130.7, 127.9, 127.2, 121.4, 112.0, 79.3, 77.0, 68.9, 64.9, 58.8, 56.7, 56.6, 37.9, 37.3, 36.1, 26.2, 15.9, 13.04, 12.93, 12.77, 12.67.

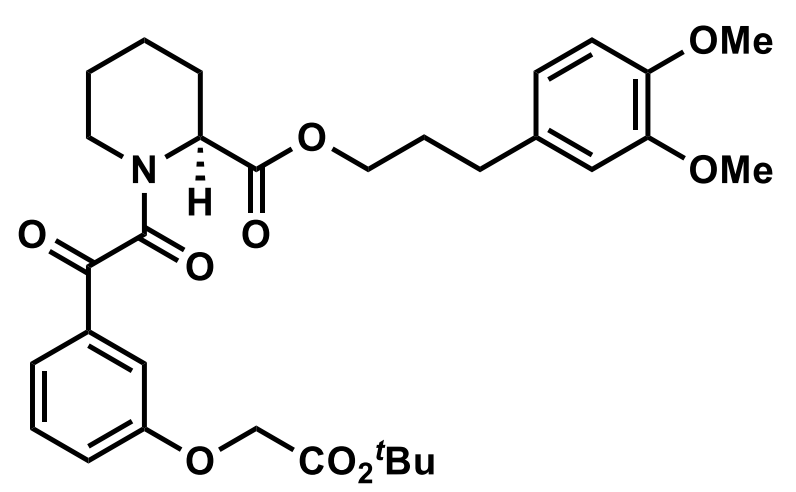

FKBP-L1-tBu: ^{1}H NMR (400 MHz, $CDCl_3$) δ 7.68 – 7.62 (m, 1H), 7.56 – 7.53 (m, 1H), 7.45 – 7.39 (m, 2H), 7.23 (dd, *J* = 8.3, 2.7 Hz, 1H), 6.80 (d, *J* = 8.5 Hz, 1H), 6.75 – 6.70 (m, 2H), 5.42 – 5.37 (d, *J* = 4.6 Hz, 1H), 4.59 (d, *J* = 2.8 Hz, 2H), 4.25 (t, *J* = 6.5 Hz, 2H), 3.87 (s, 3H), 3.86 (s, 3H), 3.47 (dd, *J* = 13.4, 3.9 Hz, 1H), 3.24 (td, *J* = 13.0, 3.4 Hz, 1H), 2.68 (t, *J* = 7.6 Hz, 2H), 2.43 – 2.33 (m, 1H), 2.03 (dt, *J* = 8.7, 6.6 Hz, 2H), 1.86 – 1.75 (m, 2H), 1.66 – 1.50 (m, 2H), 1.49 (s, 9H), 1.43 – 1.31 (m, 1H); ^{13}C NMR (101 MHz, $CDCl_3$) δ 191.5, 170.5, 167.7, 167.5, 158.6, 149.1, 147.5, 134.6, 133.6, 130.4, 128.8, 127.6, 123.7, 122.7, 120.4, 113.5, 111.8, 111.4, 82.8, 65.8, 65.1, 56.1, 56.0, 51.8, 44.4, 31.8, 30.6, 28.2, 26.5, 25.0, 21.3. Mixture of amide bond rotamers. Only the resonances of major rotamer are listed. HRMS (ESI) *calcd* for $[C_{31}H_{39}NNaO_9]^+$ ($[M+Na]^+$): *m/z* 592.2517, found: 592.2499.

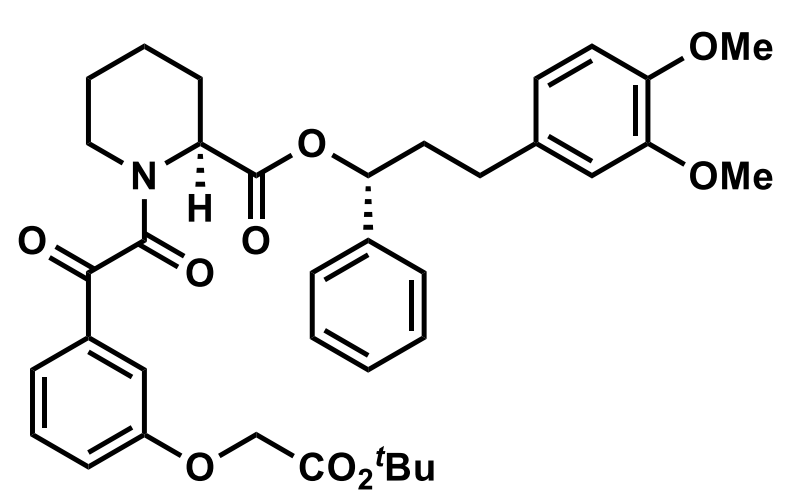

FKBP-L2-tBu: ^{1}H NMR (400 MHz, $CDCl_3$) δ 7.62 (dt, *J* = 7.6, 1.5 Hz, 1H), 7.53 (dd, *J* = 2.9, 1.5 Hz, 1H), 7.41 – 7.35 (m, 4H), 7.35 – 7.27 (m, 2H), 7.22 (ddd, *J* = 8.2, 3.0, 1.2 Hz, 1H), 6.78 (d, *J* = 8.0 Hz, 1H), 6.72 – 6.63 (m, 2H), 5.88 (dd, *J* = 7.9, 5.8 Hz, 1H), 5.44 (d, *J* = 4.8 Hz, 1H), 4.56 (d, *J* = 1.3 Hz, 2H), 3.85 (s, 3H), 3.84 (s, 3H), 3.45 (dt, *J* = 13.3, 3.4 Hz, 1H), 3.16 (td, *J* = 12.9, 3.5 Hz, 1H), 2.64 (ddd, *J* = 18.2, 9.0, 4.3 Hz, 1H), 2.60 – 2.49 (m, 1H), 2.47 – 2.39 (m, 1H), 2.35 (dddd, *J* = 13.7, 9.6, 6.0, 4.1 Hz, 1H), 2.21 – 2.11 (m, 1H), 1.83 – 1.73 (m, 2H), 1.63 – 1.51 (m, 2H), 1.49 (s, 9H), 1.40 – 1.29 (m, 2H); ^{13}C NMR (101 MHz, $CDCl_3$) δ 191.4, 169.8, 167.7, 167.2, 158.7, 149.0, 147.5, 139.8, 134.6, 133.6, 130.4, 128.8, 128.5, 126.8, 123.9, 122.7, 120.3, 113.4, 111.9, 111.4, 82.8, 77.3, 65.8, 56.1, 56.0, 51.8, 44.5, 38.2, 31.5, 28.2, 26.5, 25.0, 21.3. Mixture of amide bond rotamers. Only the resonances of major rotamer are listed. HRMS (ESI) *calcd* for $[C_{37}H_{43}NNaO_9]^+$ ($[M+Na]^+$): *m/z* 668.2830, found: 668.2802.

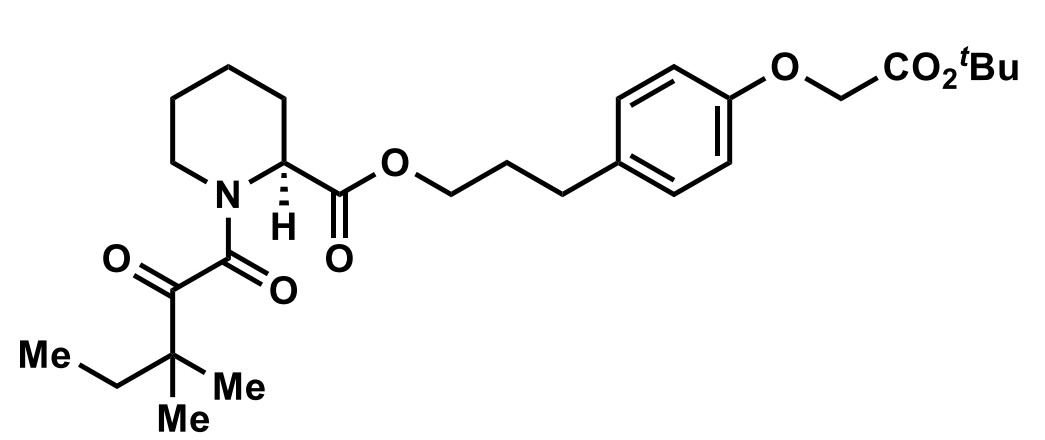

FKBP-L3-tBu: ^{1}H NMR (400 MHz, $CDCl_3$) δ 7.11 – 7.05 (m, 2H), 6.86 – 6.79 (m, 2H), 5.26 (dd, *J* = 6.3, 2.0 Hz, 1H), 4.48 (s, 2H), 4.16 (t, *J* = 6.0 Hz, 2H), 3.39 (ddd, *J* = 13.4, 4.2, 2.3 Hz, 1H), 3.22 (td, *J* = 12.9, 3.2 Hz, 1H), 2.63 (dd, *J* = 8.0, 7.3, 2H), 2.34 – 2.26 (m, 1H), 1.94 (dq, *J* = 8.7, 6.6 Hz, 2H), 1.82 – 1.60 (m, 5H), 1.58 – 1.44 (m, 1H), 1.48 (s, 9H), 1.44 – 1.34 (m, 1H), 1.24 (s, 3H), 1.20 (s, 3H), 0.89 (t, *J* = 7.4 Hz, 3H); ^{13}C NMR (101 MHz, $CDCl_3$) δ 208.0, 170.5,

168.3, 167.6, 156.5, 134.0, 129.5, 114.8, 82.4, 66.0, 64.9, 51.4, 46.9, 44.2, 32.6, 31.3, 30.5, 28.2, 26.6, 25.1, 23.8, 23.1, 21.4, 8.9. Mixture of amide bond rotamers. Only the resonances of major rotamer are listed. HRMS (ESI) *calcd* for $[C_{24}H_{33}NNaO_7]^+$ ($[M+Na-{}^tBu]^+$): *m/z* 470.2149, found: 470.2142.

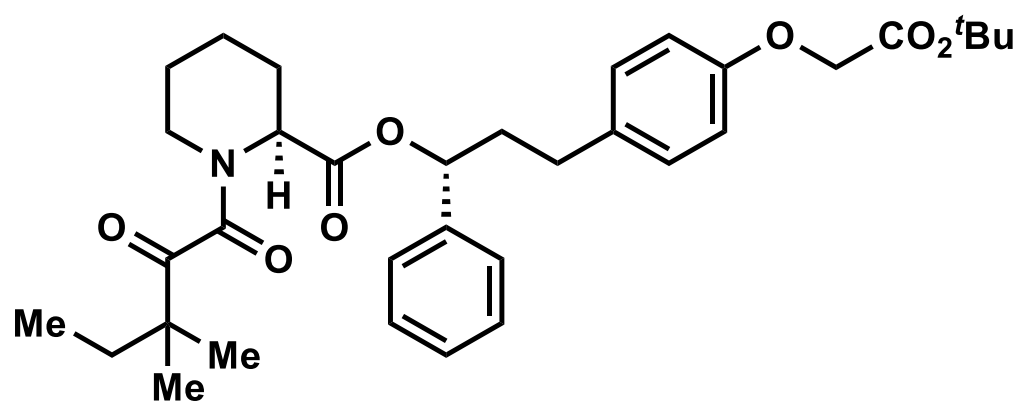

FKBP-L4-tBu: ^1H NMR (400 MHz, $CDCl_3$) δ 7.38 – 7.28 (m, 5H), 7.05 (d, *J* = 8.9, 2H), 6.81 (d, *J* = 9.0, 2H), 5.80 (dd, *J* = 7.9, 5.8 Hz, 1H), 5.31 (d, *J* = 5.4 Hz, 1H), 4.48 (s, 2H), 3.35 (ddd, *J* = 13.0, 4.2, 2.1 Hz, 1H), 3.12 (td, *J* = 13.0, 3.3 Hz, 1H), 2.64 – 2.46 (m, 2H), 2.40 – 2.32 (m, 1H), 2.29 – 2.18 (m, 1H), 2.06 (ddt, *J* = 13.7, 10.2, 5.9 Hz, 1H), 1.80 – 1.56 (m, 5H), 1.52 – 1.44 (m, 1H), 1.48 (s, 9H), 1.38 – 1.28 (m, 1H), 1.23 (s, 3H), 1.20 (s, 3H), 0.89 (t, *J* = 7.5 Hz, 3H); ^{13}C NMR (101 MHz, $CDCl_3$) δ 207.8, 169.7, 168.2, 167.2, 156.3, 139.7, 133.9, 129.3, 128.6, 128.3, 126.7, 114.6, 82.3, 76.9, 65.8, 51.3, 46.7, 44.1, 38.0, 32.5, 30.8, 28.1, 26.4, 25.0, 23.6, 23.0, 21.2, 8.8. Mixture of amide bond rotamers. Only the resonances of major rotamer are listed. HRMS (ESI) *calcd* for $[C_{34}H_{45}NNaO_7]^+$ ($[M+Na]^+$): *m/z* 602.3088, found: 602.3067.

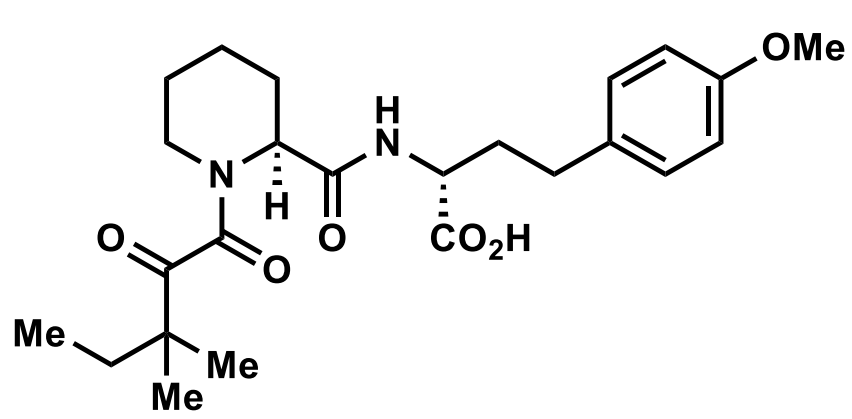

FKBP-L5: ^1H NMR (400 MHz, DMSO) δ 8.29 (d, *J* = 7.6 Hz, 0.6H), 8.25 (d, *J* = 7.8 Hz, 0.4H), 7.13 – 7.03 (m, 2H), 6.89 – 6.78 (m, 2H), 5.06 – 4.99 (m, 0.6H), 4.29 – 4.21 (m, 0.4H), 4.18 – 4.06 (m, 1.4H), 3.71 (s, 3H), 3.57 – 3.47 (m, 0.6H), 3.25 – 3.13 (m, 1H), 2.57 (tt, *J* = 9.0, 4.6 Hz, 1H), 2.52 – 2.43 (m, 1H), 2.25 – 2.13 (m, 1H), 2.03 – 1.92 (m, 1H), 1.92 – 1.81 (m, 1H), 1.74 – 1.54 (m, 5H), 1.45 – 1.24 (m, 2H), 1.14 (s, 0.6x3H), 1.12 (s, 0.6x3H), 1.11 (s, 0.4x6H), 0.80 (t, *J* = 7.5, 3H); ^{13}C NMR (101 MHz, DMSO) δ 208.1, 208.0, 173.5, 173.4, 169.9, 169.6, 166.8, 166.5, 157.6, 132.7, 129.3, 113.8, 56.0, 55.0, 51.33, 51.27, 50.6, 46.1, 43.8, 33.2, 32.7, 32.2, 32.1, 30.6, 28.6, 27.8, 24.6, 24.3, 23.1, 23.0, 22.9, 22.8, 20.3, 20.2, 8.68, 8.63. Mixture of amide bond rotamers. HRMS (ESI) *calcd* for $[C_{24}H_{34}N_2NaO_6]^+$ ($[M+Na]^+$): *m/z* 469.2309, found: 469.2298.

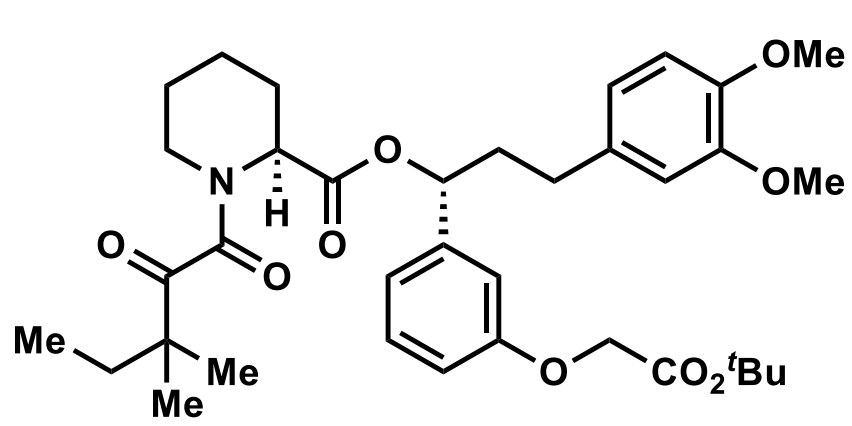

FBKP-L6-tBu: ^1H NMR (400 MHz, $CDCl_3$) δ 7.27 (t, *J* = 7.8 Hz, 1H), 6.92 – 6.85 (m, 1H), 6.83 (dd, *J* = 8.0, 2.5 Hz, 1H), 6.80 – 6.75 (m, 1H), 6.71 – 6.64 (m, 2H), 5.77 (dd, *J* = 7.8, 5.9 Hz, 1H), 5.31 (d, *J* = 5.4 Hz, 1H), 4.52 (s, 2H), 3.86 (s, 3H), 3.85 (s, 3H), 3.36 (dt, *J* = 13.2, 3.1 Hz, 1H), 3.14 (td, *J* = 13.0, 3.2 Hz, 1H), 2.55 (qdd, *J* = 14.0, 9.8, 5.9 Hz, 2H), 2.42 – 2.32 (m, 1H), 2.31 – 2.17 (m, 1H), 2.11 – 1.98 (m, 1H), 1.81 – 1.57 (m, 5H), 1.55 – 1.40 (m, 1H), 1.48 (s, 9H), 1.39 – 1.29 (m, 1H), 1.23 (s, 3H), 1.21 (s, 3H), 0.89 (t, *J* = 7.5 Hz, 3H); ^{13}C NMR (101 MHz, $CDCl_3$) δ 208.0, 169.8, 168.0, 167.4, 158.2, 149.0, 147.5, 141.5, 133.6, 129.9, 120.3, 120.0, 114.4, 113.4, 111.8, 111.4, 82.5, 76.8, 65.9, 56.1, 56.0, 51.4, 46.8, 44.3, 38.1, 32.6, 31.4, 28.2, 26.6, 25.1, 23.7, 23.2, 21.3, 8.9. Mixture of amide bond rotamers. Only the resonances of major rotamer are listed. HRMS (ESI) *calcd* for $[C_{36}H_{49}N_2NaO_9]^+$ ($[M+Na]^+$): *m/z* 662.3300, found: 662.3279.

5.6 Synthesis of DEL common intermediate

5.6.1 DEL barcoding scheme design

We used the same barcoding scheme as previously described[2,6], shown below:

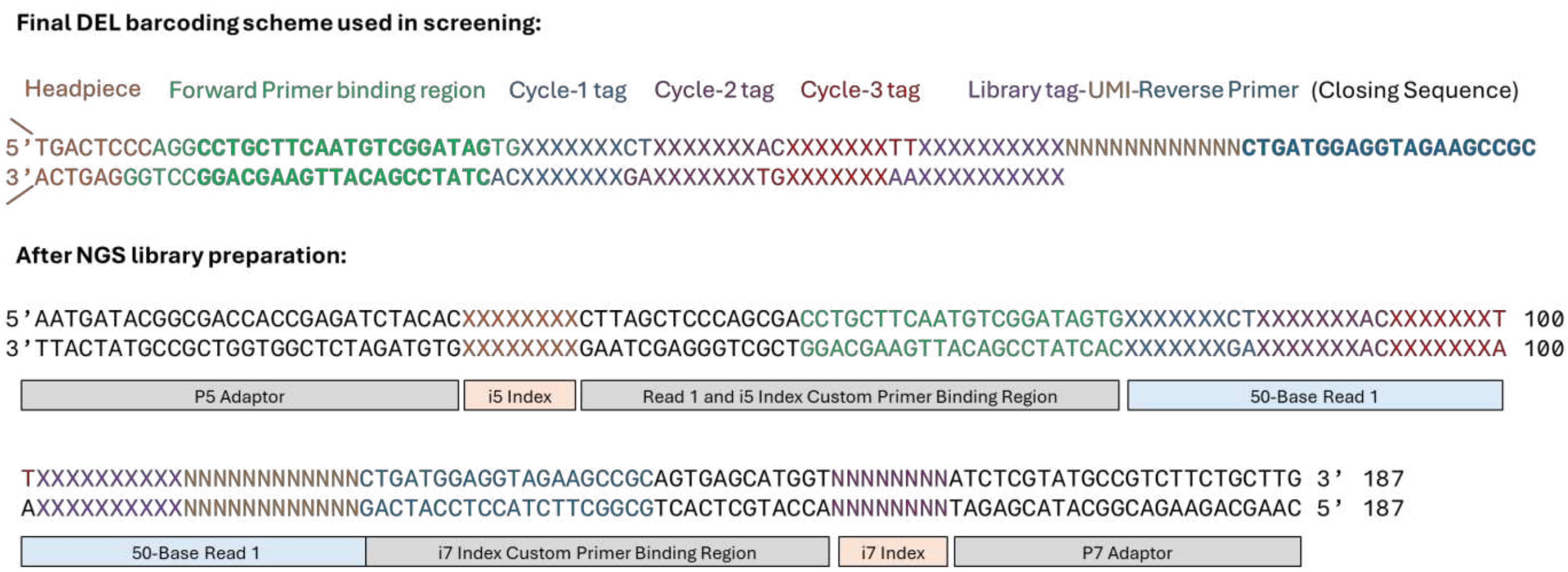

Figure S16. DEL and NGS Library barcoding schemes.

5.6.2 Cycle-1 chemistry

To five 96-well PCR plates (maximum volume 250µL) were added DNA-conjugate **2** (1.90 mM in water, 1.6µL per well, 3.0 nmol, 1 equiv.), followed by the building block stock solutions (150 mM in DMSO, 28µL per well, 4.2 µmol, 1400 equiv.). CsF solution (1.0 M in pH 10 carbonate buffer, 1.6µL per well, 1.6 µmol, 530 equiv.) was added and immediately mixed with pipette. The plates were heat-sealed with aluminum foil, vortexed, briefly spun down, and incubated at room temperature for 1.5 h. Piperidine (50% v/v in water, 5.0µL per well) was then added to each well, and the reactions were vortexed and incubated for another 20 min. The reactions were worked up by EtOH precipitation following the general protocol described before. The resulting pellets were dissolved in 25µL water. To perform reaction QC, 0.5µL of the above sample solution was taken for each well and diluted in 10µL water, which was analyzed by UPLC-MS (9µL injection volume). The remaining material was used for the subsequent ligation step. 373 out of 434 building blocks passed QC and proceeded to the subsequent steps of the library synthesis. A complete list of 373 cycle-1 building blocks is provided in Appendix II.

5.6.3 DNA ligation of forward primer binding site and cycle-1 barcodes

Due to the slight variation in cycle-1 reaction yields, the forward primer binding site ("Fwd") duplex was used as limiting reagent (2.0 nmol per well) to normalize the amount of each cycle-1 product.

To the products of cycle-1 chemical reactions (dissolved in ~25µL water) in their original plates, a freshly prepared mixture consisting of Fwd duplex (0.88 mM in water, 2.3µL, 2.0 nmol), T4 DNA ligase (30 Weiss Units/uL, 0.10µL) and 10x T4 DNA ligation buffer (7.0µL) per well was added. The plates were sealed, gently vortexed and briefly spun down. After incubating at room temperature for 1 h, the cycle-1 barcodes (1.0 mM duplex in water, 2.4µL per well, 2.4 nmol) were added to the corresponding wells. The plates were sealed, gently vortexed, briefly spun down and incubated at room temperature

for 16 h. The conversion of the ligation was checked with 4% agarose E-gel (Invitrogen, G401004). For reactions that were not complete (presumably due to residue chemicals inhibiting the enzyme), EtOH precipitation was performed and the resulting material was resubjected to the same ligation conditions. Upon completion of all ligation reactions, 5 M NaCl (2μL per well) was added, and the material in all wells were pooled together in a 50 mL canonical tube. EtOH precipitation was performed to isolate the DNA pellet.

5.6.4 Cycle-2 barcodes ligation

The DNA pellet from cycle-1 was dissolved in 2270μL water and split into 138 wells in two 96-well plates at 16.0μL per well (theoretically 5.2 nmol per well). A freshly prepared mixture of T4 DNA ligase (30 Weiss Units/uL, 0.35μL) and 10x T4 DNA ligation buffer (7.0μL) was added to each well, followed by cycle-2 barcodes (1.0 mM duplex in water, 11.5μL per well, 11.5 nmol). The plates were sealed, gently vortexed, briefly spun down, and incubated at room temperature for 12 h. The conversion of the ligation was checked with 4% agarose E-gel. Upon completion of all ligation reactions, EtOH precipitation was performed to isolate the products.

5.6.5 Cycle-2 chemistry

The DNA pellets from the above ligation step in their original wells were dissolved in 0.05 M pH 8.0 phosphate buffer (14.0μL per well). In another set of new 96-well plates, each Fmoc-amino acids (150 mM in DMF, 8.0μL) was combined with a freshly prepared mixture of EDC (100 mM in DMF, 8.0μL per well), HOAT (100 mM in DMF, 8.0μL per well) and DIPEA (150 mM in DMF, 8.0μL per well). The plates were sealed, vortexed, briefly spun down and incubated at room temperature for 30 min. 28.0μL (corresponding to ~ 130 equiv.) per well of this “activation mixture” was transferred to the DNA substrate plates. The plates were sealed, vortexed, briefly spin down and incubated at room temperature for 16 h. The DNA material was precipitated with EtOH (200μL per well) and the pellets were washed once with EtOH (200μL per well). The resulting DNA pellets were subjected to the same amide coupling condition again with freshly made acid-activation mixture. After another 16 h, the DNA was precipitated with EtOH (200μL per well). To the air-dried pellets, piperidine (10% v/v aq, 20μL per well) was added in each well. The plates were sealed, vortexed, and briefly spun down. The material in all wells was pooled together in a 50 mL canonical tube. EtOH precipitation was performed to obtain the DNA pellet.

The DNA pellet was dissolved in water to make a final volume of 1535μL (theoretical concentration 0.47 mM). This material served as the common intermediate that was to be derivatized to different libraries. Aliquots were desalted by ultracentrifuge with 3kDa MWCO filters before use.

5.7 Cycle-3 diversification of the library intermediate

5.7.1 Synthesis of CRBN CIP-DEL

The ligation and chemical reactions were carried out in 0.5 mL microcentrifuge tubes or individual wells in a 96-well plate (250μL well volume). For each of the 8 cycle-3 building blocks (CRBN ligands), 3.7μL (theoretically 0.47 mM, 1.74 nmol) of common library intermediate **4** (cycle-2 product) was used. To this DNA substrate was added a freshly made ligation mixture consisting of T4 DNA ligase (30 Weiss Units perμL, 0.20μL per well), 10x T4 DNA ligation buffer (6.0μL per well), and water (11.0μL per well),

followed by corresponding cycle-3 DNA barcode (1.0 mM duplex in water, 4.2μL). The reactions were sealed, gently vortexed, and briefly spun down. After 4 h at room temperature, the closing sequence (library tag/UMI/reverse primer) DNA duplex (0.95 mM in water, 5.3μL) was added. The reactions were sealed, gently vortexed, briefly spun down, and incubated at room temperature for another 12 h. The progress of the ligation reactions was monitored with 4% agarose E-gel. Upon the completion of the ligations, EtOH precipitation was performed to isolate the ligation products.

The DNA pellets of the ligation products proceeded to the amide coupling reaction with corresponding CRBN ligands in their original wells or tubes. The amide coupling conditions used for each CRBN ligand and the general procedure are shown in Table S1 (Section 5.5.2). For EDC-based conditions, the DNA pellets were dissolved in water (1.5μL) and phosphate or borate buffer (1.5μL). EDC (120 mM in DMF, 1.5μL), HOAT (120 mM in DMF, 1.5μL), DIPEA (150 mM in DMF, 1.5μL), and acid building block (CRBN ligand) (150 mM in DMF, 1.5μL) were mixed at room temperature for 20 min, before being added to the DNA substrates. The reactions were sealed, vortexed, briefly spun down, and incubated at room temperature for 16 h. EtOH precipitation was performed, and the resulting DNA pellets were subjected to the same amide coupling condition for another 16 h, followed by EtOH precipitation. For the DMTMM-based condition, the DNA pellets were dissolved in phosphate buffer (1.5μL). A mixture of DMTMM (300 mM aq., 1.5μL) and acid building block (150 mM in DMF, 4.5μL) was incubated at room temperature for 15 min before being added to the DNA substrates, along with DMF (4.5μL). The reactions were sealed, vortexed, briefly spun down, and incubated at room temperature for 16 h, followed by EtOH precipitation.

The DNA pellets were dissolved in water and pooled for purification. Different CRBN ligands were grouped into three pools (cycle ID = 1,4; 2,3; 5-8) based on their relative stability and were purified separately.

5.7.2 Library purification by prep cell

The final DEL purifications were performed by preparative gel electrophoresis using a prep cell system (Bio-Rad Model 491 with buffer circulation).[5] Either small gel tube (28 mm ID, Bio-Rad 1702932, for < 20 nmol crude material) or large gel tube (37 mm ID, Bio-Rad 1702933, for > 20 nmol scale) was used. The gel was cast with 4% agarose (Lonza 50080) in 1x TBE buffer to roughly 10 cm height and stored at 4 °C for at least 2 h before use. The prep cell was assembled according to manufacturer's instructions. 1x TBE was used as running buffer except for the sub-library pool of Cycle-3 ID = 2 and 3, for which TAE buffer (pH adjusted to 7.5 with acetic acid) was used. Running buffer was circulated and cooled by submerging the long circulation loop tubing in ice bath. The crude DEL samples were loaded with was loaded in Novex™ Hi-Density TBE Sample Buffer (5X, Invitrogen LC6678), and electrophoresis was run in constant power mode at 12 W (for small gel tube) or 15 W (for large gel tube). Fraction collection was performed using a Masterflex L/S easy-load II (Cole Parmer, 77200-50) running at 1 mL/min and BioFrac™ fraction collector (Bio-Rad) with a fraction time window of 1-2 min. Total electrophoresis time was roughly 4-5 h. Fractions were analyzed with 4% agarose E-gel. Acceptably clean product-containing fractions were pooled, then concentrated and desalted by ultrafiltration using 10kDa MWCO centrifugal filters (Millipore UFC9010). The purified libraries were collected and quantified by Qubit (sub-library CRBN-1&4: 0.25 nmol, 7.3%; sub-library CRBN-2&3: 0.47 nmol, 14%; sub-library CRBN-5-8: 1.3 nmol, 18%). Sub-libraries were pooled in equal equivalents before screening.

5.7.3 Synthesis of VHL CIP-DEL

The VHL CIP-DEL was synthesized following the same ligation and amide coupling reaction protocols as CRBN CIP-DEL. EDC-based condition (phosphate buffer) was used for all three VHL ligands. For each VHL ligand, 2.1μL of common library intermediate **4** (0.47 mM, 1.0 nmol theoretical), 0.067μL T4 DNA ligase (30 Weiss Unit/uL), 3.0μL T4 ligation buffer (10x), 2.3μL water, 2.5μL cycle-3 DNA barcode duplex (1.0 mM in water, 2.5 nmol), and 3.7μL closing sequence DNA duplex (0.95 mM in water, 3.5 nmol) was used in the ligation step. For the amide coupling step, 1.1μL EDC (120 mM in DMF), 1.1μL HOAT (120 mM in DMF), 1.1μL DIPEA (150 mM in DMF), 1.1μL VHL ligand (150 mM in DMF), and 1.5μL phosphate buffer (pH 8.0) was used for each reaction. A portion of the crude VHL CIP-DEL (1/5, 0.6 nmol theoretical) was purified by 4% agarose E-gel (Invitrogen G401004) and cleaned up with QIAquick Gel Extraction Kit (Qiagen 28704) according to manufacturer's instructions. The purified library was quantified by Qubit (0.090 nmol, 15% overall yield).

5.7.4 Synthesis of FKBP CIP-DEL

The FKBP CIP-DEL was synthesized following the same ligation and amide coupling reaction protocols as CRBN CIP-DEL. EDC-based condition (phosphate buffer) was used for all six FKBP12 ligands. The *t*-Bu ester groups were deprotected by treatment of TFA in DCM (1:10) at 0 °C for 10-24 h. For each FKBP12 ligand, 5.5μL of common library intermediate **4** (0.47 mM, 2.6 nmol theoretical), 0.20μL T4 DNA ligase (30 Weiss Unit/uL), 8.0μL T4 ligation buffer (10x), 8μL water, 6.4μL cycle-3 DNA barcode duplex (1.0 mM in water, 6.4 nmol), and 8.2μL closing sequence DNA duplex (0.95 mM in water, 7.7 nmol) was used in the ligation step. For the amide coupling step, 2.0μL EDC (120 mM in DMF), 2.0μL HOAT (120 mM in DMF), 2.0μL DIPEA (150 mM in DMF), 2.0μL FKBP12 ligand (150 mM in DMF), and 2.6μL phosphate buffer (pH 8.0) was used for each reaction. The crude library was purified by prep cell gel electrophoresis following the same procedure described above. The purified library was quantified by Qubit (2.4 nmol, 16% overall yield).

6. Synthesis of DEL screening positive and control compounds

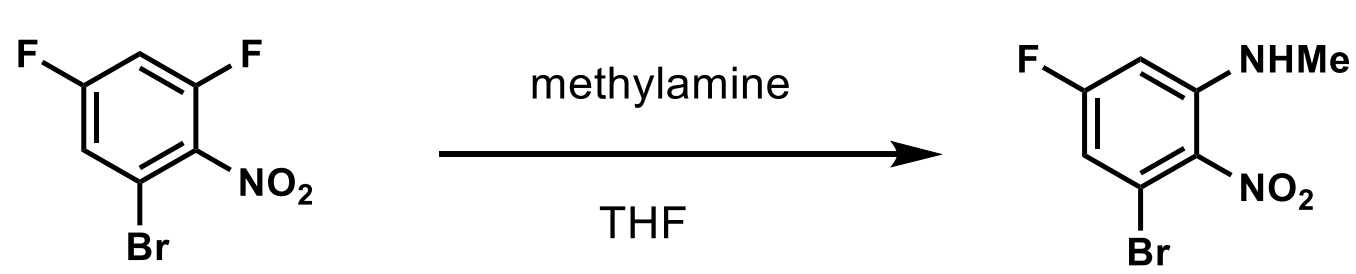

19 **20**

Compound **20**: 1-bromo-3,5-difluoro-2-nitrobenzene (4.00 g, 16.8 mmol, 1 equiv.) was dissolved in THF (45 mL) in a round-bottom flask. The solution was cooled to 0 °C and methylamine (40 wt% aq., 2.2 mL, 25 mmol, 1.5 equiv.) was added dropwise. After 1h, the reaction was concentrated *in vacuo* then re-dissolved in DCM (50 mL) and washed twice with water (2 x 10 mL). The DCM layer was dried over Na_2SO_4 and concentrated *in vacuo* to yield compound **20** (4.0 g, 96%) as an orange solid. This material is used directly in the next step without further purification. ^{1}H NMR (400 MHz, $CDCl_3$) δ 6.71 (dd, *J* = 7.8, 2.5 Hz, 1H), 6.42 (dd, *J* = 10.8, 2.5 Hz, 1H), 6.29 (brs, 1H), 2.91 (d, *J* = 4.9 Hz, 3H); ^{13}C NMR (101 MHz, $CDCl_3$) δ 164.4 (d, *J* = 257 Hz), 146.8 (d, *J* = 257 Hz), 132.9, 118.75 (d, *J* = 14 Hz), 110.0 (d, *J* = 27 Hz), 98.6 (d, *J* = 26 Hz), 30.4. LRMS (ESI) *calcd* for $[C_7H_5BrFN_2O_2]^-$ ($[M-H]^-$): *m/z* 246.95, found: 246.9.

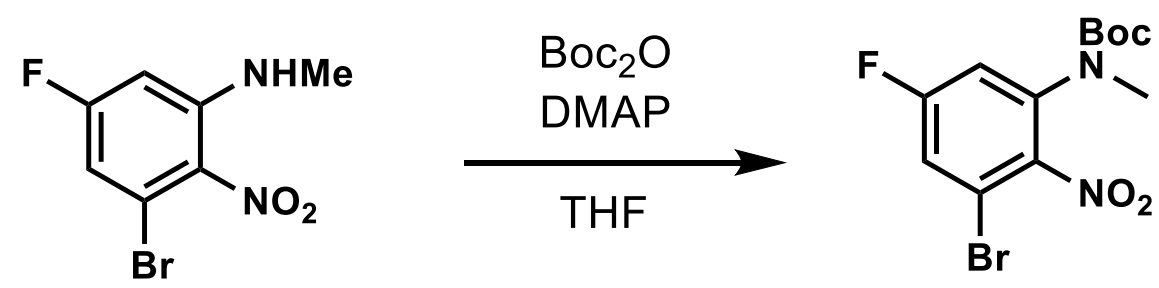

20

21

Compound **21**: To a round-bottom flask was added compound **20** (3.95 g, 15.9 mmol, 1 equiv.), DMAP (194 mg, 1.59 mmol, 0.1 equiv.) and THF (40 mL). Boc_2O (4.50 g, 20.6 mmol, 1.3 equiv.) was then added and the reaction was allowed to stir at room temperature for 12 h. The solvents were removed *in vacuo*, and the residue was directly purified by flash column chromatography (5-15% EtOAc/Heptane) to afford compound **21** (4.85 g, 88%). ^{1}H NMR (400 MHz, $CDCl_3$) δ 7.33 (dd, *J* = 7.5, 2.7 Hz, 1H), 7.01 (dd, *J* = 8.3, 2.9 Hz, 1H), 3.13 (s, 3H), 1.34 (s, 9H); ^{13}C NMR (101 MHz, $CDCl_3$) δ 162.1 (d, *J* = 129 Hz), 153.0, 146.6, 139.2 (d, *J* = 11 Hz), 119.9 (d, *J* = 25 Hz), 116.1 (d, *J* = 23 Hz), 114.9 (d, *J* = 12 Hz), 82.2, 37.5, 27.9. LRMS (ESI) *calcd* for $[C_7H_5BrFN_2O_2]^+$ ($[M\text{-}Boc+H]^+$): *m/z* 248.97, found: 248.9.

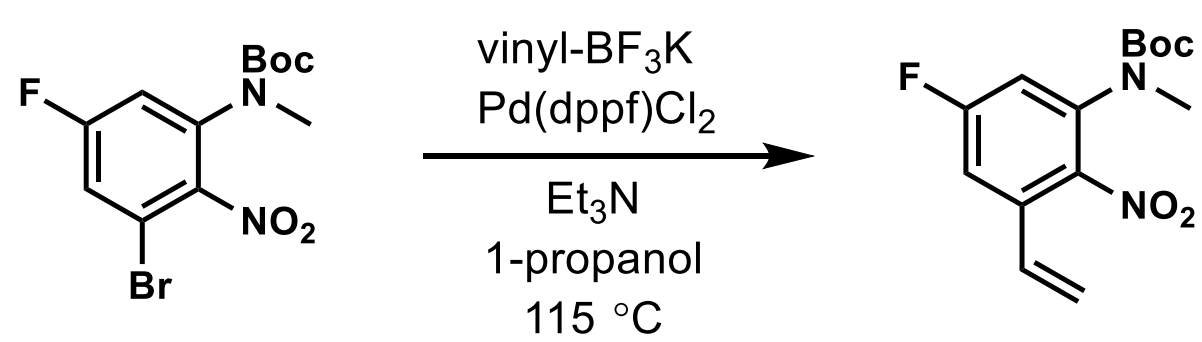

21

22

Compound **22**: To a round-bottom flask was added compound **21** (4.27 g, 12.2 mmol, 1 equiv.), potassium trifluoro(vinyl)borate (1.80 g, 13.5 mmol, 1.1 equiv.) and $Pd(dppf)Cl_2$ (0.49 g, 0.40 mmol, 0.04 equiv.). The flask was evacuated and backfilled with N_2. 1-Propanol (30 mL) and triethylamine (5.1 mL, 37 mmol, 3.0 equiv.) were added and the mixture was heated at 115 °C for 3 h. The reaction was cooled to room temperature, filtered through celite, and concentrated *in vacuo*. The residue was purified by flash column chromatography (40-100% DCM/Heptane) to afford compound **22** (1.85 g, 51%). ^{1}H NMR (400 MHz, $CDCl_3$) δ 7.21 (dd, *J* = 8.9, 2.7 Hz, 1H), 6.92 (d, *J* = 7.2 Hz, 1H), 6.66 (dd, *J* = 17.3, 11.0 Hz, 1H), 5.80 (d, *J* = 17.2 Hz, 1H), 5.50 (d, *J* = 11.0 Hz, 1H), 3.15 (s, 3H), 1.32 (s, 9H); ^{13}C NMR (101 MHz, $CDCl_3$) δ 162.7 (d, *J* = 255 Hz), 153.2, 144.3 (d, *J* = 4 Hz), 138.4 (d, *J* = 10 Hz), 133.8, 129.4, 121.1, 115.6 (d, *J* = 23 Hz), 112.2 (d, *J* = 24 Hz), 81.8, 37.5, 27.9. LRMS (ESI) *calcd* for $[C_{10}H_{10}FN_2O_4]^+$ ($[M\text{-}tBu+H]^+$): *m/z* 241.0619, found: 241.0.

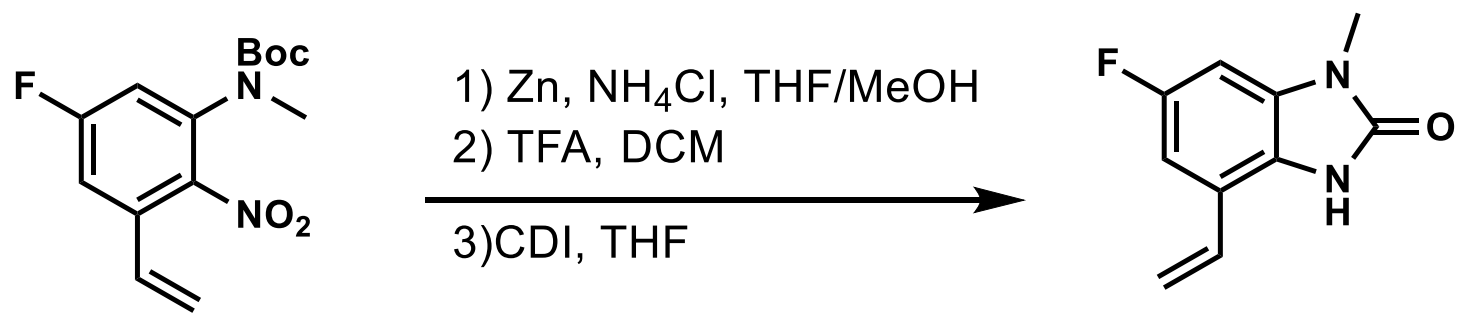

22

7

Compound **7**: To a round-bottom flask was added compound **22** (1.72 g, 5.80 mmol, 1 equiv.), THF (10 mL), MeOH (10 mL), and Zinc dust (2.66 g, 40.6 mmol, 7 equiv.). Saturated aq. NH_4Cl (~ 7 M, 8.3 mL, 58 mmol, 10 equiv.) was added dropwise. After stirring at room temperature for 3 h, the reaction was filtered through celite and concentrated *in vacuo*. The residue was re-dissolved in DCM, washed with brine, and concentrated *in vacuo* to provide the crude nitro reduction product. This crude product was

dissolved in DCM (18 mL) and TFA (6 mL) was added. After 2 h at room temperature, the mixture was concentrated *in vacuo*, and the residue was directly used in the next step. The crude Boc-deprotection product was dissolved in THF (20 mL) and carbonyldiimidazole (1.41 g, 8.7 mmol, 1.5 equiv.) was added. The reaction was allowed to stir at room temperature for 12 h, before being concentrated *in vacuo*. The residue was purified by flash column chromatography (0-10% MeOH/DCM) to afford compound **7** (861 mg, 77% over 3 steps). ^{1}H NMR (400 MHz, $CDCl_3$) δ 10.54 (s, 1H), 6.91 (dd, *J* = 10.9, 2.4 Hz, 1H), 6.85 (ddd, *J* = 17.6, 10.8, 1.3 Hz, 1H), 6.63 (dd, *J* = 8.0, 2.4 Hz, 1H), 5.83 (d, *J* = 17.6 Hz, 1H), 5.51 (d, *J* = 11.1 Hz, 1H), 3.41 (s, 3H); ^{13}C NMR (101 MHz, $CDCl_3$) δ 159.0 (d, *J* = 238 Hz), 156.5, 132.1 (d, *J* = 12 Hz), 130.4 (d, *J* = 2 Hz), 122.10, 120.9 (d, *J* = 8 Hz) , 117.32, 104.8 (d, *J* = 24 Hz), 95.4 (d, *J* = 29 Hz), 27.2. HRMS (ESI) *calcd* for $[C_{10}H_{10}FN_2O]^+$ ($[M+H]^+$): *m/z* 193.0772, found: 193.0776.

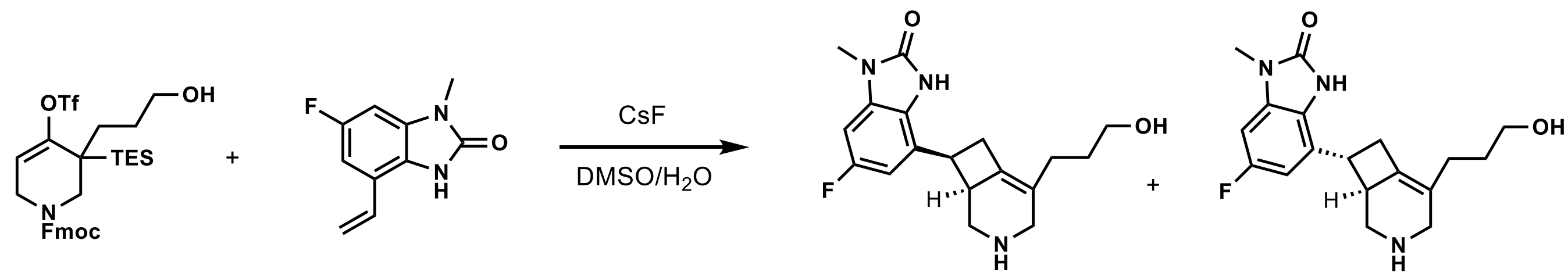

1 7 pre-B67a pre-B67b

Compound **pre-B67a/b**: Strained allene precursor **1** (50 mg, 0.080 mmol, 1 equiv.) and the alkene building block **7** (35 mg, 0.18 mmol, 2.3 equiv.) were dissolved in DMSO (2 mL) in a 2-dram glass vial. To this solution was added CsF (5.0 M aq., 0.12 mL, 0.6 mmol, 7.5 equiv.), and the reaction was stirred at room temperature for 5 h. The reaction was then diluted with H_2O (2 mL) and DCM (2 mL), and the phases were separated. The aqueous layer containing the products was taken, and the organic layer was extracted twice with water (2 x 0.5 mL). The aqueous layers were washed twice with DCM (2 x 1 mL). The combined aqueous layers were directly injected and purified by prep-HPLC (15-40% MeCN in water, 10 mM ammonium hydroxide). The fractions were lyophilized to afford major isomer **pre-B67b** (3.2 mg, 12%) and minor isomer **pre-B67a** (1-2 mg, ~ 5%, based on LCMS).

Major isomer **pre-B67b**: ^{1}H NMR (600 MHz, MeOD) δ 6.90 (dd, *J* = 11.1, 2.4 Hz, 1H), 6.81 (dd, *J* = 8.5, 2.3 Hz, 1H), 3.58 (t, *J* = 6.5 Hz, 2H), 3.35 (s, 3H), 3.41 – 3.17 (m, 6H), 2.92 (t, *J* = 10.6 Hz, 1H), 2.62 – 2.41 (m, 1H), 2.28 – 2.15 (m, 1H), 2.02 (dt, *J* = 14.8, 7.7 Hz, 1H), 1.70 (tdd, *J* = 10.0, 7.5, 5.0 Hz, 1H), 1.64 (dtt, *J* = 13.4, 6.7, 3.0 Hz, 1H); ^{13}C NMR (151 MHz, MeOD) δ 160.5 (d, *J* = 242 Hz), 157.5, 132.9 (d, *J* = 13 Hz), 130.7, 127.6 (d, *J* = 8 Hz), 124.2, 123.3, 106.5 (d, *J* = 24 Hz), 95.3 (d, *J* = 29 Hz), 62.4, 48.4, 47.19, 47.15, 40.1, 39.2, 31.9, 27.3, 26.7. 2D-NMR spectra available in Appendix III. HRMS (ESI) *calcd* for $[C_{18}H_{23}FN_3O_2]^+$ ($[M+H]^+$): *m/z* 332.1769, found: 332.1784.

Minor isomer **pre-B67a**: ^{1}H NMR (600 MHz, MeOD) δ 6.83 (dd, *J* = 8.4, 2.4 Hz, 1H), 6.75 (dd, *J* = 11.3, 2.4 Hz, 1H), 3.88 (td, *J* = 8.9, 2.3 Hz, 1H), 3.63 – 3.56 (m, 2H), 3.56 – 3.50 (m, 1H), 3.36 (s, 3H), 3.35 – 3.31 (m, 1H), 3.29 – 3.24 (m, 1H), 3.11 – 3.02 (m, 2H), 2.69 (dd, *J* = 11.7, 6.5 Hz, 1H), 2.30 (dt, *J* = 15.3, 8.2 Hz, 1H), 2.02 (ddd, *J* = 14.6, 9.5, 5.7 Hz, 1H), 1.78 – 1.68 (m, 1H), 1.64 (ddt, *J* = 13.3, 9.5, 6.5 Hz, 1H), 1.56 (dd, *J* = 11.8, 10.2 Hz, 1H); ^{13}C NMR (151 MHz, MeOD) δ 160.4 (d, *J* = 237 Hz), 157.4, 132.61, 132.58 (d, *J* = 12 Hz), 124.9, 124.5 (d, *J* = 8 Hz), 124.2 (d, *J* = 2 Hz), 106.3 (d, *J* = 26 Hz), 95.4 (d, *J* = 29 Hz), 62.6, 47.0, 45.3, 42.9, 36.8, 34.7, 32.0, 27.2, 26.5. 2D-NMR spectra available in Appendix III. HRMS (ESI) *calcd* for $[C_{18}H_{23}FN_3O_2]^+$ ($[M+H]^+$): *m/z* 332.1769, found: 332.1782.

Higher yields (45% and 14% yield for major and minor isomers, respectively) could be achieved by using Boc-protected strained allene precursor instead of Fmoc under same condition, followed by TFA-mediated Boc deprotection.

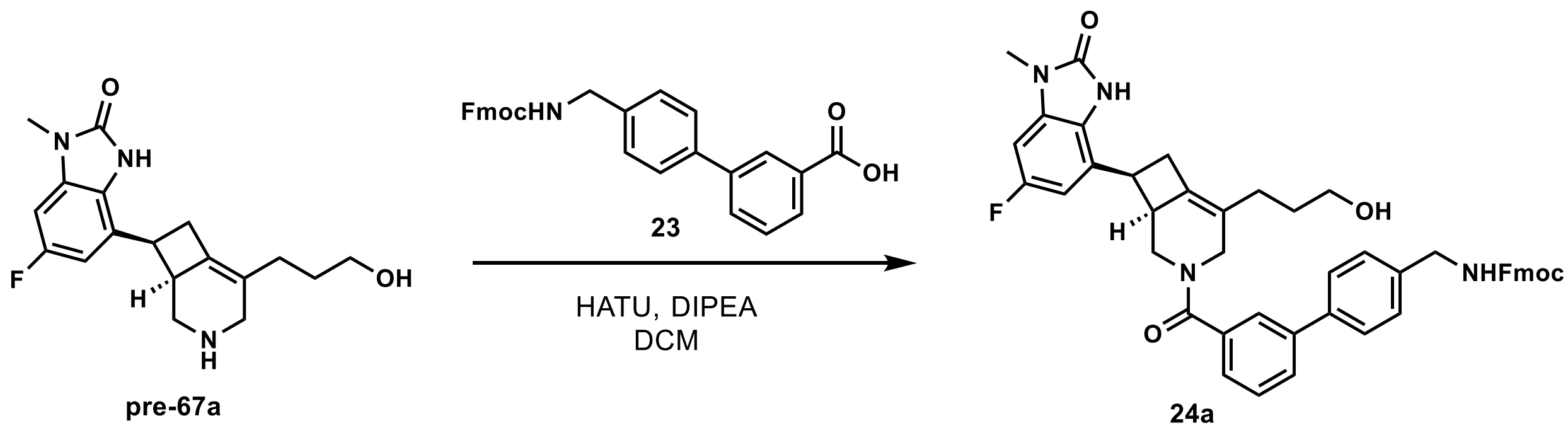

Compound **24a**: To a 1-dram glass vial was added Fmoc-amino acid **23** (3.8 mg, 8.6 μmol, 2.6 equiv.), HATU (3.0 mg, 8.0 μmol, 2.4 equiv.), DCM (0.20 mL), and DIPEA (4.6μL, 26 μmol, 8.0 equiv.). The mixture was stirred at room temperature for 2 h when all the solids dissolved. A portion of this activation mixture (0.10 mL, corresponding to 1.3 equiv. acid, 1.2 equiv. HATU, and 4.0 equiv. DIPEA) was added to another 1-dram glass vial containing compound **pre-67a** (1.1 mg, 3.3 μmol, 1 equiv.) and DCM (0.15 mL). The reaction was stirred at room temperature for 2 h before being quenched with saturated aq. NH_4Cl and extracted 3 times with DCM (3 x 1 mL). The combined organic layers were dried over Na_2SO_4 and concentrated *in vacuo*. The residue was purified by flash column chromatography (40-60% Acetone/DCM) to afford compound **24a** (1.0 mg, 39%). ^{1}H NMR (400 MHz, CD_2Cl_2) δ 11.06 – 10.65 (br, 1H), 7.85 – 7.73 (m, 2H), 7.72 – 7.53 (m, 3H), 7.47 – 7.23 (m, 7H), 7.13 (s, 4H), 6.75 – 6.62 (m, 2H), 5.45 (brs, 1H), 4.64 – 4.43 (m, 3H), 4.43 – 4.33 (m, 2H), 4.32 – 4.24 (m, 1H), 3.85 (t, *J* = 9.5 Hz, 1H), 3.75 – 3.53 (m, 3H),3.44 – 3.35 (m, 1H), 3.40 (s, 3H), 3.35 – 3.25 (m, 1H), 3.20 (d, *J* = 18.3 Hz, 1H), 3.05 (d, *J* = 14.9 Hz, 1H), 2.32 – 2.09 (m, 2H), 1.92 – 1.53 (m, 3H); ^{13}C NMR (101 MHz, CD_2Cl_2) δ 170.4, 159.5 (d, *J* = 237 Hz), 157.0, 156.7, 144.5, 141.7, 141.6, 138.7, 138.6, 135.1, 131.5, 131.1 (d, *J* = 15 Hz), 128.2, 128.1, 127.8, 127.5, 127.4, 127.0, 126.2, 125.4, 124.2, 123.1 (d, *J* = 8 Hz), 123.0, 120.3, 106.4 (d, *J* = 24 Hz), 94.8 (d, *J* = 28 Hz), 66.9, 62.6, 47.8, 45.8, 45.5, 44.8, 44.6, 36.0, 32.4, 31.2, 27.4, 25.9. HRMS (ESI) *calcd* for $[C_{47}H_{44}FN_4O_5]^+$ ($[M+H]^+$): *m/z* 763.3290, found: 763.3263.

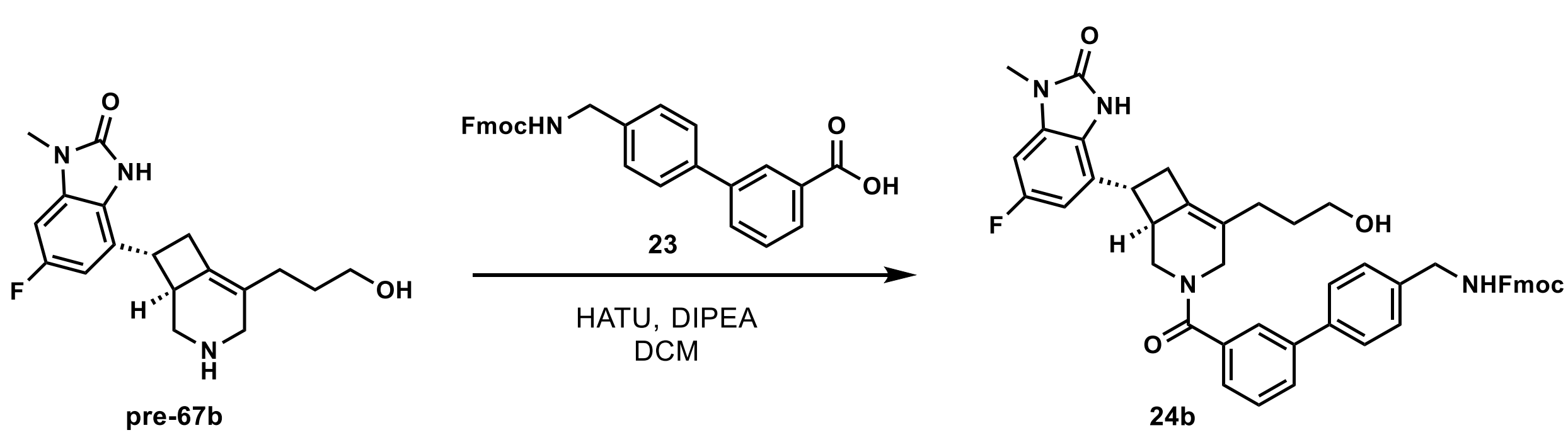

Compound **24b**: To a 1-dram glass vial was added Fmoc-amino acid **23** (4.6 mg, 10 μmol, 1.3 equiv.), HATU (3.6 mg, 9.4 μmol, 1.2 equiv.), DCM (0.20 mL), and DIPEA (5.5μL, 31 μmol, 4.0 equiv.). The mixture was stirred at room temperature for 2 h when all the solids dissolved. This activation mixture

was then transferred to another 1-dram glass vial containing compound **pre-67b** (2.6 mg, 7.9 μmol, 1 equiv.) and DCM (0.15 mL). The reaction was stirred at room temperature for 4 h before being quenched with saturated aq. NH_4Cl and extracted 3 times with DCM (3 x 1 mL). The combined organic layers were dried over Na_2SO_4 and concentrated *in vacuo*. The residue was purified by flash column chromatography (40-60% Acetone/DCM) to afford compound **24b** (1.4 mg, 23%). ^{1}H NMR (400 MHz, $CDCl_3$) δ 10.71 – 10.51 (br, 1H), 7.76 (d, *J* = 7.6 Hz, 2H), 7.66 – 7.18 (m, 14H), 0.86 (d, *J* = 12.2 Hz, 0.4H), 6.70 (d, *J* = 10.8 Hz, 0.6H), 6.66 – 6.55 (m, 1H), 5.59 – 5.19 (br, 1H), 5.04 – 4.88 (m, 0.4H), 4.76 (d, *J* = 17.9 Hz, 0.6H), 4.47 (d, *J* = 6.6 Hz, 2H), 4.44 – 4.28 (m, 2H), 4.22 (t, *J* = 6.8 Hz, 1H), 4.13 – 3.97 (m, 0.6H), 4.03 – 3.93 (m, 0.4H), 3.88 – 3.76 (m, 0.4H), 3.75 – 3.57 (m, 2.6H), 3.47 – 3.26 (m, 2.4H), 3.36 (s, 3H), 3.26 – 3.14 (m, 0.6H), 3.07 – 2.97 (m, 0.6H), 3.00 – 2.83 (m, 1H), 2.79 – 2.66 (m, 0.4H), 2.32 – 1.97 (m, 2H), 1.88 – 1.56 (m, 2H); ^{13}C NMR (101 MHz, $CDCl_3$) δ 171.2, 170.9, 159.1 (d, *J* = 239 Hz), 156.68, 156.64, 156.55, 156.43, 144.0, 142.1, 141.5, 139.3, 138.3, 131.4 (d, *J* = 13 Hz), 130.9, 129.2, 128.1, 127.8, 127.5, 127.2, 125.9, 125.1, 123.1, 122.1, 121.9, 120.1, 106.0 (d, *J* = 24 Hz), 94.5 (d, *J* = 28 Hz), 66.8, 62.0, 50.4, 50.0, 47.4, 46.5, 45.2, 44.8, 44.7, 38.9, 38.4, 37.7, 37.2, 30.5, 27.2, 26.0, 25.6. HRMS (ESI) *calcd* for $[C_{47}H_{44}FN_4O_5]^+$ ($[M+H]^+$): *m/z* 763.3290, found: 763.3257.

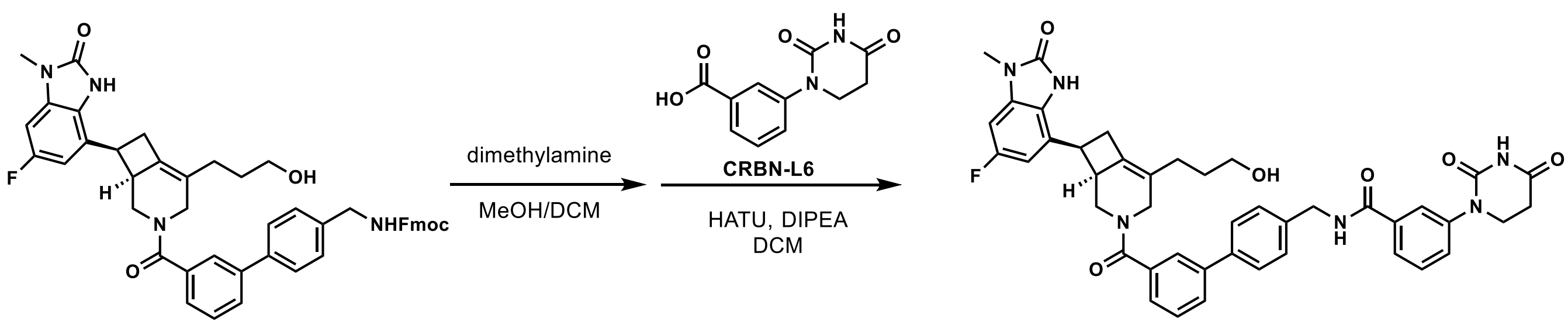

24a B67a

Compound **B67a**: To a 1-dram glass vial was added compound **24a** (2.0 mg, 2.6 μmol, 1 equiv.), DCM (0.05 mL), and dimethylamine (2.0 M in MeOH, 0.25 mL, 0.50 mmol, 190 equiv.). After stirring at room temperature for 6 h, the reaction was concentrated *in vacuo* and the residue was further dried under high vacuum overnight before being used for the next step.

The deprotected amine was dissolved in DCM (0.15 mL) in a 1-dram glass vial. The carboxylic acid **CRBN-L6** (1.1 mg, 4.7 μmol, 1.8 equiv.), HATU (1.5 mg, 3.9 μmol, 1.5 equiv.), and DIPEA (1.8μL, 10 μmol, 4.0 equiv.) were mixed and stirred in DCM (0.10 mL) for 1 h, and the mixture was transferred to the vial containing the amine substrate. After stirring at room temperature for 8 h, the reaction was concentrated *in vacuo* and directly purified by prep-HPLC (20-40% MeCN in water, 0.1% formic acid). The fractions were lyophilized to afford compound **B67a** (1.6 mg, 81%). ^{1}H NMR (400 MHz, MeOD) δ 8.55 (s, 1H), 7.84 (d, *J* = 2.2 Hz, 1H), 7.79 (d, *J* = 6.9 Hz, 1H), 7.72 – 7.58 (m, 4H), 7.58 – 7.51 (m, 2H), 7.49 – 7.33 (m, 4H), 6.91 – 6.72 (m, 2H), 4.69 – 4.59 (m, 0.6H), 4.64 (s, 2H), 4.31 (dd, *J* = 11.6, 6.2 Hz, 0.4H), 3.98 (t, *J* = 8.3 Hz, 0.4H), 3.97 – 3.88 (m, 0.4H), 3.93 (t, *J* = 6.7 Hz, 2H), 3.82 (t, *J* = 8.5 Hz, 0.6H), 3.78 – 3.70 (m, 0.4H), 3.70 – 3.61 (m, 0.4H + 0.6x2H), 3.61 – 3.55 (m, 0.6H), 3.55 – 3.44 (m, 0.6H + 0.6H + 0.4x2H), 3.38 (s, 0.6x3H), 3.40 – 3.29 (m, 1H), 3.27 (s, 0.6x3H), 3.10 (d, *J* = 14.4 Hz, 1H), 2.84 (t, *J* = 6.7 Hz, 2H), 2.41 (dt, *J* = 15.4, 7.9 Hz, 0.6H), 2.26 – 2.16 (m, 1H), 2.15 (t, *J* = 11.1 Hz, 0.6H), 2.01 – 1.95 (m, 0.4H), 1.95 – 1.86 (m, 0.4H), 1.86 – 1.69 (m, 1H), 1.64 – 1.49 (m, 1H); ^{13}C NMR (101 MHz, MeOD) δ 173.2, 172.8, 169.4, 160.4 (d, *J* = 237 Hz), 157.5, 157.3, 154.6, 143.9, 143.7, 143.3, 140.2, 140.0, 136.7, 136.1, 133.4, 132.70, 132.6, 132.4, 130.8, 130.4, 129.8, 129.3, 128.6, 128.3, 128.2, 128.1, 126.5, 125.6, 124.8, 124.2, 123.9, 123.5, 106.3 (d, *J* = 25 Hz), 95.7 (d, *J* = 29 Hz),

62.5, 62.4, 51.5, 46.8, 46.4, 45.7, 45.2, 44.6, 44.3, 42.0, 36.5, 36.2, 34.2, 33.9, 32.10, 32.04, 31.96, 28.1, 27.2, 26.9, 26.7, 26.2. HRMS (ESI) *calcd* for $[C_{43}H_{42}FN_6O_6]^+$ ($[M+H]^+$): *m/z* 757.3144, found: 757.3148.

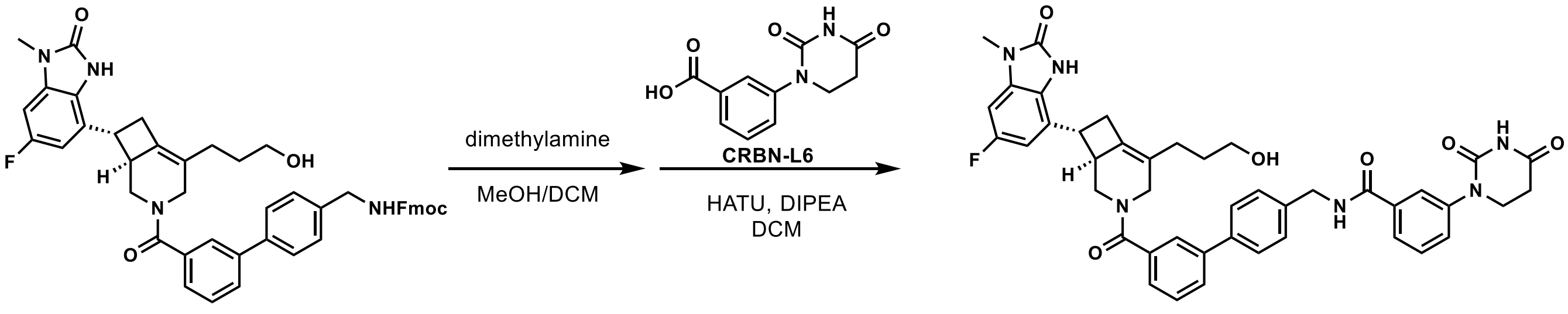

24b B67b

Compound **B67b**: To a 1-dram glass vial was added compound **24b** (5.7 mg, 6.7 μmol, 1 equiv.), DCM (0.20 mL), and dimethylamine (2.0 M in MeOH, 0.20 mL, 0.40 mmol, 60 equiv.). After stirring at room temperature for 6 h, the reaction was concentrated *in vacuo* and the residue was further dried under high vacuum overnight before being used for the next step.

The deprotected amine was dissolved in DCM (0.15 mL) in a 1-dram glass vial. The carboxylic acid **CRBN-L6** (2.8 mg, 12 μmol, 1.8 equiv.), HATU (3.8 mg, 10 μmol, 1.5 equiv.), and DIPEA (4.7μL, 27 μmol, 4.0 equiv.) were mixed and stirred in DCM (0.30 mL) for 1 h, and the mixture was transferred into the vial containing the amine substrate. After stirring at room temperature for 8 h, the reaction was concentrated *in vacuo* and directly purified by prep-HPLC (20-45% MeCN in water, 0.1% formic acid). The fractions were lyophilized to afford compound **B67b** (2.8 mg, 55%). ^{1}H NMR (400 MHz, MeOD) δ 7.89 – 7.81 (m, 1H), 7.81 – 7.75 (m, 1H), 7.75 – 7.56 (m, 4H), 7.56 – 7.39 (m, 6H), 7.03 – 6.72 (m, 2H), 4.89 – 4.79 (m, 0.4H), 4.78 – 4.69 (m, 0.6H), 4.62 (d, *J* = 9.5 Hz, 2H), 4.58 (s, 1H), 4.08 – 3.91 (m, 0.6+2x0.4H), 3.92 (t, *J* = 6.6 Hz, 2H), 3.69 – 3.59 (m, 1.6H), 3.54 – 3.47 (m, 1H), 3.48 – 3.21 (m, 3H), 3.37 (s, 0.6x3H), 3.30 (s, 0.4x3H), 3.10 – 2.90 (m, 1.6H), 2.83 (t, *J* = 6.7 Hz, 2H), 2.83 – 2.76 (m, 0.4H), 2.38 – 2.25 (m, 0.4H), 2.23 – 2.08 (m, 1H), 2.07 – 1.93 (m, 0.6H), 1.88 – 1.66 (m, 1H), 1.65 – 1.47 (m, 1H); ^{13}C NMR (101 MHz, MeOD) δ 173.3, 173.0, 172.8, 169.4, 160.5 (d, *J* = 241 Hz), 157.5, 154.6, 144.0, 143.8, 143.3, 140.2, 140.0, 139.9, 136.6, 136.2, 136.1, 132.9 (d, *J* = 13 Hz), 131.6, 130.9, 130.80, 130.75, 130.4, 129.8, 129.3, 129.2, 128.8, 128.25, 128.20, 128.1, 127.2 (d, *J* = 8 Hz), 126.9 (d, *J* = 8 Hz), 126.5, 125.6, 123.9, 123.5, 123.4, 106.6 (d, *J* = 25 Hz), 106.5 (d, *J* = 24 Hz), 95.5 (d, *J* = 29 Hz), 62.4, 62.3, 51.8, 50.9, 47.8, 46.4, 46.2, 46.0, 44.3, 39.5, 38.9, 38.7, 38.4, 32.1, 31.9, 31.8, 28.1, 27.2, 26.92, 26.90, 26.4. HRMS (ESI) *calcd* for $[C_{43}H_{42}FN_6O_6]^+$ ($[M+H]^+$): *m/z* 757.3144, found: 757.3138.

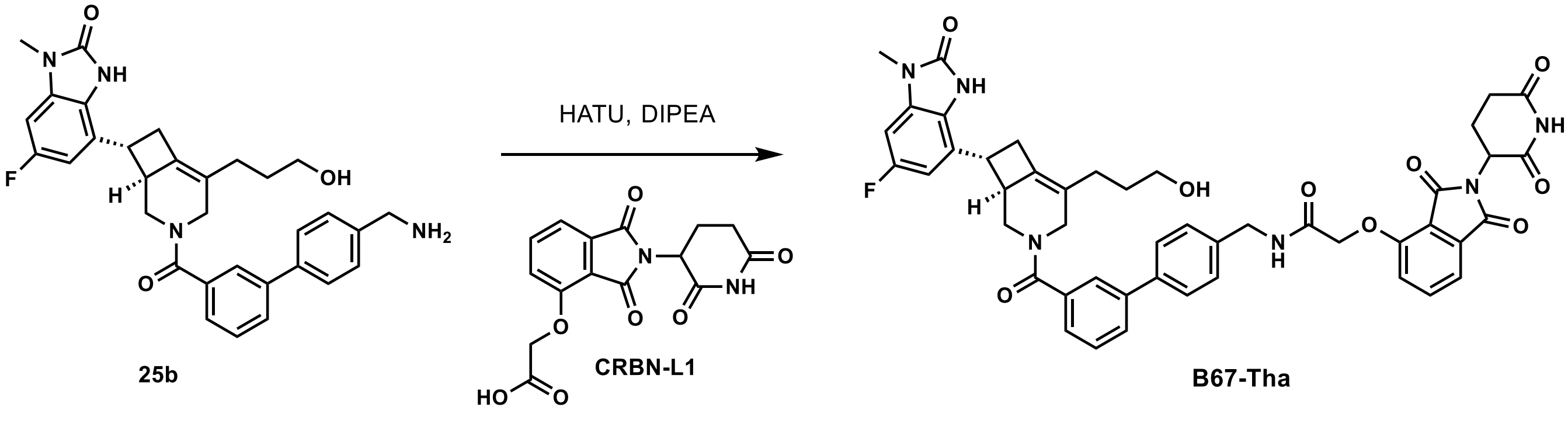

25b B67-Tha

Compound **B67-Tha**: The crude deprotected amine **25b** (described above in **B67b** synthesis, 6.7 μmol, 1 equiv.) was dissolved in DCM (0.15 mL) in a 1-dram glass vial. The carboxylic acid **CRBN-L1** (4.0 mg, 12 μmol, 1.8 equiv.), HATU (3.8 mg, 10 μmol, 1.5 equiv.), and DIPEA (4.7μL, 27 μmol, 4.0 equiv.) were mixed and stirred in DCM (0.30 mL) for 1 h, and the mixture was transferred into the vial containing the amine substrate **25b**. After stirring at room temperature for 8 h, the reaction was concentrated *in vacuo* and directly purified by prep-HPLC (25-45% MeCN in water, 0.1% formic acid). The fractions were lyophilized to afford compound **B67-Tha** (2.9 mg, 50%). ^{1}H NMR (400 MHz, MeOD) δ 7.83 – 7.75 (m, 1H), 7.75 – 7.60 (m, 3H), 7.60 – 7.49 (m, 3H), 7.49 – 7.35 (m, 4H), 6.98 (d, *J* = 11.2 Hz, 0.4H), 6.87 (d, *J* = 10.9 Hz, 0.6H), 6.82 (d, *J* = 8.8 Hz, 0.4H), 6.76 (d, *J* = 8.3 Hz, 0.6H), 5.13 – 4.95 (m, 1H), 4.90 – 4.68 (m, 1H), 4.87 – 4.78 (s, 2H), 4.61 – 4.49 (m, 2H), 4.11 – 3.89 (m, 0.4x2H+0.6H), 3.70 – 3.58 (m, 1.6H), 3.51 (t, *J* = 6.8 Hz, 1H), 3.49 – 3.30 (m, 2H), 3.37 (s, 0.4x3H), 3.30 (s, 0.6x3H), 3.33 – 3.22 (m, 1H), 3.10 – 2.90 (m, 1.6H), 2.86 – 2.52 (m, 0.4H+2H+1H), 2.38 – 2.24 (m, 0.6H), 2.23 – 1.97 (m, 0.6H+0.4x2H+1H), 1.85 – 1.67 (m, 1H), 1.63 – 1.51 (m, 1H); ^{13}C NMR (101 MHz, MeOD) δ 174.4, 174.2, 173.2, 172.9, 171.24, 171.18, 169.9, 168.2, 167.7, 160.4 (d, *J* = 238 Hz), 157.4, 156.2, 143.8, 143.5, 140.3, 139.2, 138.9, 138.2, 136.3, 136.0, 134.8, 132.8 (d, *J* = 13 Hz), 131.6, 130.73, 130.69, 129.4, 129.3, 128.7, 128.21, 128.14, 128.08, 126.9, 123.8, 123.4, 123.3, 121.9, 119.3, 118.0, 106.5 (d, *J* = 25 Hz), 95.4 (d, *J* = 29 Hz), 69.6, 62.4, 62.2, 51.7, 50.9, 50.5, 47.8, 46.2, 46.0, 43.6, 43.5, 39.4, 38.9, 38.7, 38.3, 38.2, 32.1, 31.85, 31.76, 27.3, 26.9, 26.3, 23.54, 23.48 (a small amount of CD_2Cl_2 was added to the NMR sample for solubility). HRMS (ESI) *calcd* for $[C_{47}H_{44}FN_6O_9]^+$ ($[M+H]^+$): *m/z* 855.3148, found: 855.3159.

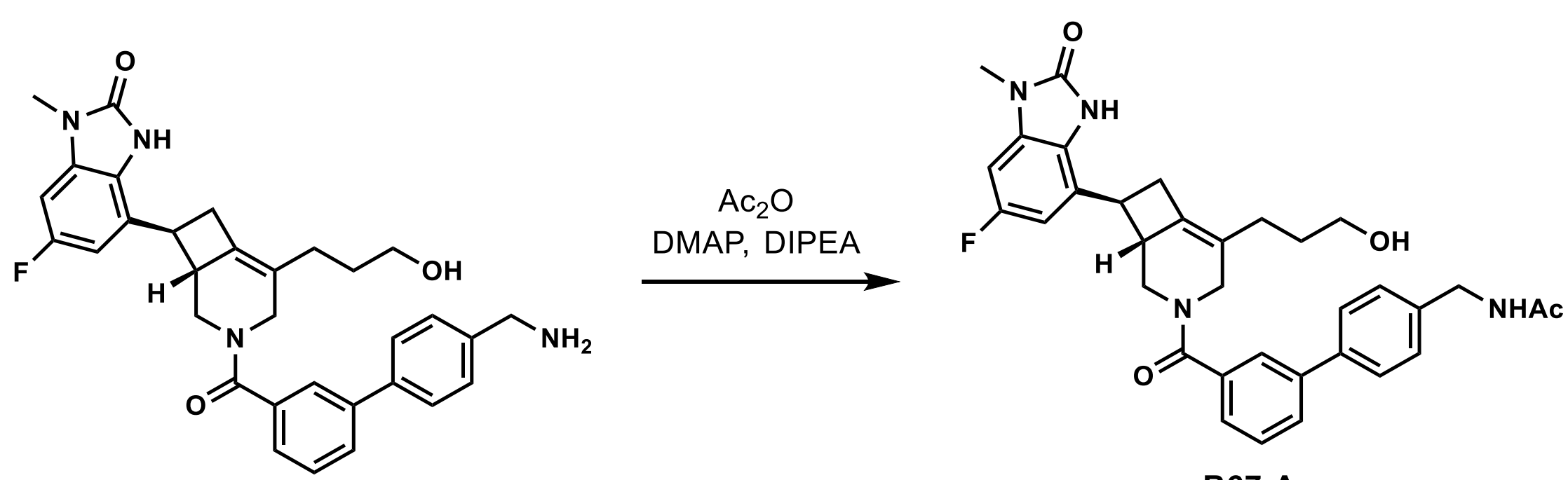

25b B67-Ac

Compound **B67-Ac**: The crude deprotected amine **25b** (described above in **B67b** synthesis, 6.7 μmol, 1 equiv.) was dissolved in DCM (0.30 mL) in a 1-dram glass vial. To this solution was added DMAP (0.1 mg, 0.7 μmol, 0.1 equiv.), DIPEA (4.7μL, 27 μmol, 4.0 equiv.), and acetic anhydride (0.8μL, 9 μmol, 1.3 equiv.). After stirring at room temperature for 8 h, the reaction was concentrated *in vacuo*. The residue was dissolved in methanol (0.4 mL) and NaOH (1.0 M aq., 20μL) was added to hydrolyze the extra acetylation of the primary hydroxyl group. After stirring for 30 min, the mixture was directly injected and purified by prep-HPLC (20-45% MeCN in water, 0.1% formic acid). The fractions were lyophilized to afford compound **B67-Ac** (2.0 mg, 51%). ^{1}H NMR (400 MHz, MeOD) δ 7.67 – 7.45 (m, 4H), 7.43 (d, *J* = 7.8 Hz, 0.4x2H), 7.37 (d, *J* = 7.9 Hz, 0.6x2H), 7.32 – 7.22 (m, 2H), 6.89 (d, *J* = 11.0 Hz, 0.4H), 6.81 – 6.71 (m, 0.4H+0.6H), 6.68 (d, *J* = 8.3 Hz, 0.6H), 4.79 – 4.71 (m ,0.4H), 4.64 (d, *J* = 17.3 Hz, 0.6H), 4.31 (s, 0.4x2H), 4.29 (s, 0.6x2H), 3.98 – 3.80 (m, 0.4x2H+0.6H), 3.60 – 3.49 (m, 1.6H), 3.45 – 3.37 (m, 1H), 3.38 – 3.21 (m, 2H), 3.28 (s, 0.4x3H), 3.22 (s, 0.6x3H), 3.24 – 3.15 (m, 1H), 3.01 – 2.80 (m, 1.6H), 2.72 (t, *J* = 10.5 Hz, 0.4H), 2.26 – 2.17 (m, 0.4H), 2.12 – 1.99 (m, 1H), 1.96 – 1.85 (m, 0.6H+3H), 1.66 (tt, *J* = 14.5, 6.9 Hz, 1H), 1.54 – 1.38 (m, 1H); ^{13}C NMR (101 MHz, MeOD) δ 173.3, 173.2, 173.0, 160.5 (d, *J*

= 238 Hz), 157.5, 144.0, 143.8, 140.2, 139.9, 139.8, 136.2, 136.1, 132.9 (d, *J* = 13 Hz), 131.6, 130.8, 129.26, 129.22, 128.8, 128.2, 128.1, 127.2 (d, *J* = 8 Hz), 126.9 (d, *J* = 9 Hz), 123.9, 123.5, 123.4, 106.5 (d, *J* = 25 Hz), 95. 5 (d, *J* = 29 Hz), 62.4, 62.3, 51.8, 50.9, 47.8, 46.2, 46.0, 43.9, 39.5, 39.0, 38.7, 38.4, 31.9, 31.8, 27.2, 26.92, 26.90, 26.4, 22.6. HRMS (ESI) *calcd* for $[C_{34}H_{36}FN_4O_4]^+$ ($[M+H]^+$): *m/z* 583.2715, found: 583.2722.

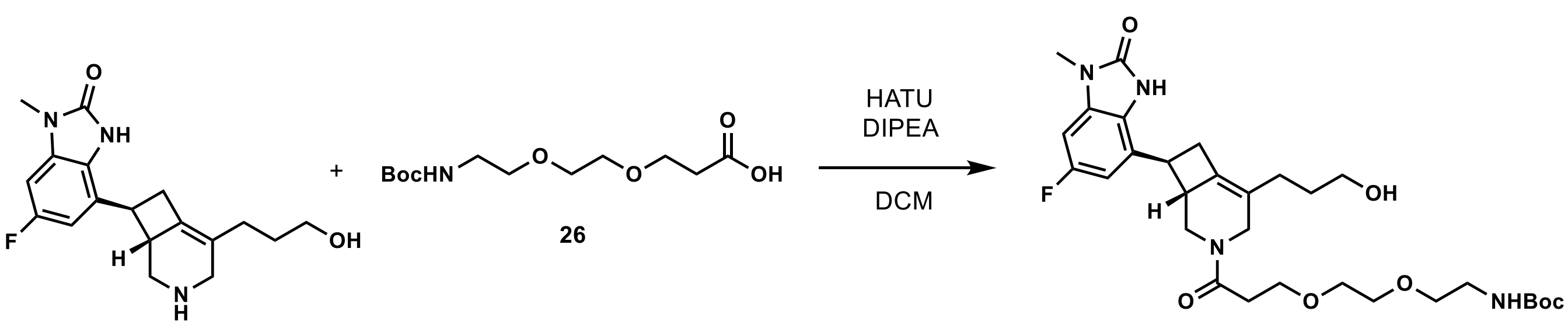

Compound **27b**: To a 1-dram glass vial was added Boc-amino acid **26** (10 mg, 37 µmol, 4.0 equiv.), HATU (12 mg, 32 µmol, 3.4 equiv.), DCM (0.30 mL), and DIPEA (13µL, 74 µmol, 8.0 equiv.), and the mixture was stirred at room temperature for 1 h. This activation mixture was then transferred to another 1-dram glass vial containing compound **pre-B67b** (3.1mg, 9.3 µmol, 1 equiv.) and DCM (0.20 mL). After stirring at room temperature for 12 h, the reaction was filtered through a 0.45 µm filter and the filter was washed with DCM. The crude mixture was concentrated *in vacuo*, and the residue was purified by flash column chromatography (20-30% MeOH/DCM), followed by prep-HPLC (20-50% MeCN in water, 10 mM ammonium hydroxide). The fractions were lyophilized to afford compound **27b** (3.7 mg, 67%). ^{1}H NMR (400 MHz, MeOD) δ 6.97 (dd, *J* = 11.1, 2.4 Hz, 0.6H), 6.92 (dd, *J* = 11.1, 2.4 Hz, 0.4H), 6.85 – 6.80 (m, 1H), 4.80 (dd, *J* = 11.8, 6.1 Hz, 0.4H), 4.67 – 4.59 (m, 0.6H), 4.31 – 4.24 (m, 0.6H), 4.25 (dd, *J* = 12.6, 5.8 Hz, 0.4H), 3.86 – 3.78 (m, 0.4H), 3.80 – 3.73 (m, 2H), 3.63 – 3.52 (m, 6H), 3.49 (t, *J* = 5.6 Hz, 0.4x2H), 3.44 (t, *J* = 5.6 Hz, 0.6x2H), 3.44 – 3.36 (m, 1.6H), 3.363 (s, 0.6x3H), 3.360 (s, 0.4x3H), 3.34 – 3.25 (m, 1.4H), 3.23 – 3.13 (m, 0.6H), 3.21 (t, *J* = 5.6 Hz, 0.4x2H), 3.16 (t, *J* = 5.6 Hz, 0.6x2H), 2.98 (dd, *J* = 12.8, 9.9 Hz, 0.6H), 3.02 – 2.89 (m, 1H), 2.79 – 2.58 (m, 2H), 2.52 (dd, *J* = 11.9, 10.1 Hz, 0.4H), 2.25 (dt, *J* = 15.0, 7.7 Hz, 1H), 2.10 (dt, *J* = 14.8, 7.6 Hz, 1H), 1.80 – 1.62 (m, 2H), 1.41 (s, 9H); ^{13}C NMR (101 MHz, MeOD) δ 172.9, 160.5 (d, *J* = 236 Hz), 158.4, 157.5, 132.9 (d, *J* = 13 Hz), 131.6, 130.7, 127.26 (d, *J* = 8 Hz), 127.23 (d, *J* = 8 Hz), 123.9, 123.44, 123.38 (d, *J* = 7 Hz), 106.73 (dd, *J* = 25 Hz), 106.54 (dd, *J* = 25 Hz), 95.44 (d, *J* = 29 Hz), 95.41 (d, *J* = 29 Hz), 80.1, 71.51, 71.47, 71.25, 71.16, 71.09, 70.99, 68.5, 68.4, 62.38, 62.36, 49.1, 48.8, 48.4, 47.9, 45.8, 44.9, 41.3, 39.3, 39.1, 38.6, 38.4, 35.3, 35.1, 31.90, 31.87, 28.8, 27.3, 26.8, 26.7. HRMS (ESI) *calcd* for $[C_{30}H_{43}FN_4NaO_7]^+$ ($[M+Na]^+$): *m/z* 613.3008, found: 613.2985.

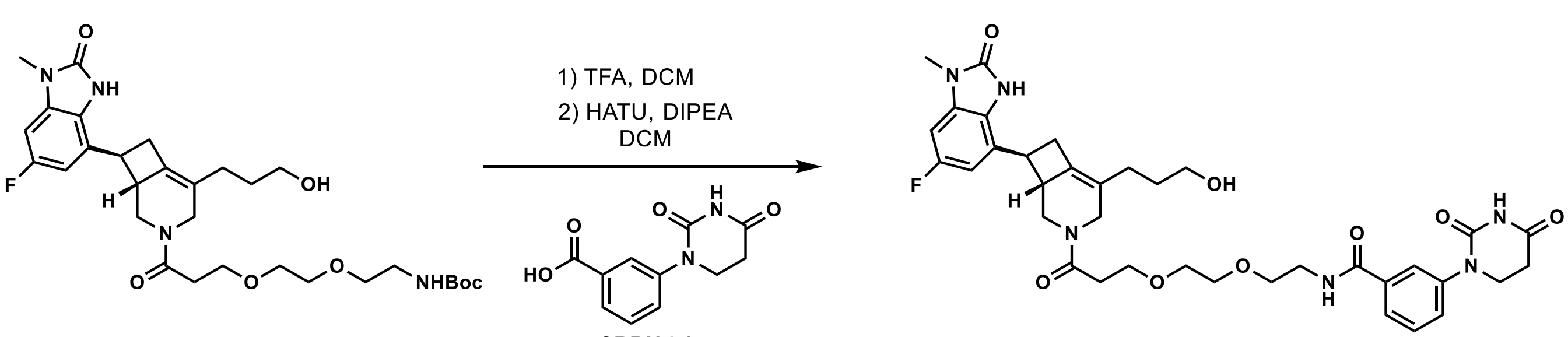

Compound **B67-PEG**: To a 1-dram glass vial was added compound **27b** (3.7 mg, 6.3 μmol, 1 equiv.) and DCM (0.3 0 mL). TFA (0.10 mL) was added dropwise and the reaction was stirred at room temperature for 1 h. The reaction was then concentrated *in vacuo*, and the residue was further dried under high vacuum for 5 h. The crude amine was then dissolved in DCM (0.20 mL) in another 1-dram vial. The carboxylic acid **CRBN-L6** (2.0 mg, 8.8 μmol, 1.4 equiv.), HATU (2.9 mg, 7.5 μmol, 1.2 equiv.), and DIPEA (4.4μL, 25 μmol, 4.0 equiv.) were mixed and stirred in DCM (0.30 mL) for 1 h, and the mixture was transferred to the vial containing the amine substrate. After stirring at room temperature for 2 h, the reaction was concentrated *in vacuo* and directly purified by prep-HPLC (15-35% MeCN in water, 10 mM ammonium hydroxide). The fractions were lyophilized to afford compound **B67-PEG** (2.6 mg, 59%). ^{1}H NMR (400 MHz, MeOD) δ 7.83 – 7.77 (m, 1H), 7.74 – 7.67 (m, 1H), 7.53 – 7.32 (m, 3H), 6.96 (dt, *J* = 11.1, 1.9 Hz, 0.6H), 6.90 (dt, *J* = 11.1, 1.9 Hz, 0.4H), 6.81 (dt, *J* = 8.5, 1.9 Hz, 1H), 4.73 (dd, *J* = 12.0, 6.1 Hz, 0.4H), 4.59 (d, *J* = 17.6 Hz, 0.6H), 4.26 – 4.17 (m, 0.6H), 4.19 (dd, *J* = 12.1, 6.0 Hz, 0.4H), 3.90 (t, *J* = 6.8 Hz, 2H), 3.81 – 3.76 (m, 0.4H), 3.76 – 3.71 (m, 2H), 3.68 – 3.49 (m, 10H), 3.40 – 3.22 (m, 1.6H), 3.36 (d, *J* = 1.7 Hz, 0.4x3H), 3.35 (d, *J* = 1.4 Hz, 0.6x3H), 3.34 – 3.22 (m, 1.4H), 3.18 – 2.97 (m, 2H), 3.05 – 2.92 (m, 1H), 2.92 (t, *J* = 11.3, 0.6H), 2.82 (t, *J* = 6.6 Hz, 2.6H), 2.73 – 2.53 (m, 2H), 2.46 (t, *J* = 11.0 Hz, 0.4H), 2.22 (dt, *J* = 14.8, 7.5 Hz, 1H), 2.15 – 2.02 (m, 1H), 1.77 – 1.59 (m, 2H); ^{13}C NMR (101 MHz, DMSO) δ 172.87, 172.84, 172.78, 169.45, 169.35, 160.5 (d, *J* = 237 Hz), 157.5, 154.5, 143.3, 136.70, 136.66, 132.9 (d, *J* = 13 Hz), 131.6, 130.7, 130.3, 130.2, 129.75, 129.72, 129.4, 127.4, 127.25 (d, *J* = 9 Hz), 126.46, 126.43, 125.5, 123.9, 123.44, 123.37 (d, *J* = 8 Hz), 106.74 (d, *J* = 25 Hz), 106.55 (d, *J* = 24 Hz), 95.46 (d, *J* = 29 Hz), 95.42 (d, *J* = 29 Hz), 71.6, 71.5, 71.4, 71.2, 70.5, 70.4, 68.5, 68.4, 62.4, 58.6, 49.1, 48.8, 47.9, 46.4, 45.7, 44.9, 41.11, 41.05, 39.3, 39.1, 38.5, 38.3, 35.3, 35.1, 32.1, 31.89, 31.86, 30.7, 28.8, 27.3, 26.8, 26.7. HRMS (ESI) *calcd* for $[C_{36}H_{44}FN_6O_8]^+$ ($[M+H]^+$): *m/z* 707.3199, found: 707.3160.

7. Supplementary References

1. Lim, K. S.; Reidenbach, A. G.; Hua, B. K.; Mason, J. W.; Gerry, C. J.; Clemons, P. A.; Coley, C. W. Machine learning on DNA-encoded library count data using an uncertainty-aware probabilistic loss function. *Journal of Chemical Information and Modeling* **2022**, *62*(10), 2316-2331.
2. Mason, J. W.; Chow, Y. T.; Hudson, L.; Tutter, A.; Michaud, G.; Westphal, M. V.; *et al*. DNA-encoded library-enabled discovery of proximity-inducing small molecules. *Nature Chemical Biology* **2024**, *20*(2), 170-179.
3. Liu, S., Tong, B., Mason, J. W., Ostrem, J. M., Tutter, A., Hua, B. K., ... & Schreiber, S. L. (2023). Rational screening for cooperativity in small-molecule inducers of protein–protein associations. *Journal of the American Chemical Society* **2023**, *145*(42), 23281-23291.
4. Tan, Z. Y.; Adade, J. K.; Gu, X.; Hecht, C. J.; Salcius, M.; Tong, B.; Liu, S.; Hwang, S.; Zécri, F. J.; Graham, D. B.; *et al*. Development of an FKBP12-recruiting chem[2]ical-induced proximity DNA-encoded library and its application to discover an autophagy potentiator. *Cell Chem. Biol.* **2025**, *32* (3), 498–510.
5. Westphal, M. V.; Hudson, L.; Mason, J. W.; Pradeilles, J. A.; Zécri, F. J.; Briner, K.; Schreiber, S. L. Water-compatible cycloadditions of oligonucleotide-conjugated strained allenes for DNA-encoded library synthesis. *Journal of the American Chemical Society* **2020**, *142*(17), 7776-7782.
6. Hudson, L.; Mason, J. W.; Westphal, M. V.; Richter, M. J.; Thielman, J. R.; Hua, B. K.; *et al*. Diversity-oriented synthesis encoded by deoxyoligonucleotides. *Nature Communications* **2023**, *14*(1), 4930.

Appendix I: Building block validation data

Table S2. Building block validation Data of Alkenes, Dienes, and Pyrroles for Cycle-1

Structure	yield	Included?
Alkenes		
C=CN1N=C(C)C=CC1=O	100%	Y
C/C(=C\C#N)/c1ccccn1	88%	Y
C=Cc1cnc(nc1)N(C)C	87%	Y
C=Cc1cnc(N)nc1	100%	Y
CC(=O)/C=C/c1ccccc1	95%	Y
C=Cc1cccc2NC=Cc12	0%	N
CC(=C)C1C=NNC(=O)C=1	100%	Y
C=Cc1ccc(Cl)nn1	100%	Y
CN(C)/C=C/c1ccncc1	0%	N
C=Cc1cnc(C#N)c(F)c1	56%	N
Oc2ccc1C=CC(=O)c1c2	0%	N
C2COc1ccccc1C=2	0%	N
COc2ccc1CC(=C)c1c2	0%	N
O=C2C=Cc1ccccc1C2=O	0%	N
CC(=C)c1nccc2NC=Cc12	95%	Y
O=C1CCC(=C1)c2ccccc2	47%	N
C=Cc2ccc1NC(=O)Cc1c2	90%	Y
O=C1C=C(CN1)c2ccccc2	0%	N
C=Cc1cccc2CC(=O)Nc12	100%	Y
C=Cc1ncnc2c1N=CN2C	85%	Y
O=C1OCC=C1c2ccccc2	78%	Y
C=Cc1ncnc2c1C=NN2C	60%	N
CC2(C)C=Cc1cccnc1O2	0%	N
COc2ccc1C=CCOc1c2	0%	N
C=C1CCCc2c(F)cccc12	100%	Y
C=C(C1CC1)c2ccc(F)cc2	0%	N
C=Cc1cc(F)cc2NN=Nc12	100%	Y
COC(=O)/C=C/c1ccncc1	85%	Y
Fc1ccccc1C2COCC=2	0%	N
C=Cc1cc(cnn1)C(=O)OC	0%	N
C=Cc1cc(ccc1OC)OC	100%	Y
CC1C=C(Cl)c2ccccc12	0%	N
O=S2(=O)C=Cc1ccccc12	68%	N
C=Cc1cncc(F)c1C(C)F	100%	Y
N#CC2=Nc1ccccc1C=CO2	0%	N
C=Cc1ccc(cc1)C2=CNC=N2	100%	Y
CN2C=Cc1ccccc1C2C#N	0%	N
C=CC2NC(=O)c1ccccc1N=2	97%	Y
C(=C/c1ccccc1)\C2NN=NN=2	0%	N
O=C1CCC/C/1=C\c2ccccc2	80%	Y
N#C/C(=C\C(N)=O)/c1ccccn1	100%	Y

CN2C=Cc1ccccc1CC2=O	0%	N
O=C1NCC/C/1=C\c2ccccc2	0%	N
CC(=C)c2ccc(NC1CC1)nc2	100%	Y
[O-][N+](=O)c1cccc2C=CCCc12	20%	N
COc2ccc1OCCC=Cc1c2	0%	N
COC(=O)N/C=C/c1ccncc1	15%	N
N#C/C=C/C2COc1ccccc1C=2	90%	Y
C=Cc1cnc(nc1)c2ccccn2	0%	N
COc1cccc2C(C#N)=CCCc12	100%	Y
CC1=NNC(=O)/C/1=C/c2ccccc2	0%	N
Cc1ccc(/C=C/c2nnn[nH]2)cc1	0%	N
C=CN2N=C(C)c1ccccc1C2=O	24%	N
CC(=C)C2=Nc1ccccc1C(=O)O2	0%	N
OC2C=C(/C=C\c1ccccc1)ON=2	0%	N
C(=C/c1ccccn1)\C2CCCCC2	0%	N
COc2ccc1C=CNC(=O)Cc1c2	0%	N
CC(=O)/C=C/c2ccc1OCOc1c2	100%	Y
COc2ccc1C=CC(C)(C)Oc1c2	0%	N
C=Cc1cc(F)cc2c1NC(=O)N2C	100%	Y
C=C(c1ccc(C#N)cc1)N2C=NC=N2	100%	Y
O=S2(=O)NC=Cc1ccccc1N2	0%	N
N#C/C(=C\c1ccccc1)/C(=O)C2CC2	100%	Y
C/C=C/c1ccccc1S(N)(=O)=O	0%	N
C=Cc3ccc2NC(=O)C1(CCC1)c2c3	92%	N
C=CC1NN=NN=1	0%	N
C=CN1N=CC(C#N)=C1N	99%	Y
CC(=O)/C=C/C1NC=CN=1	26%	N
CO/C=C/C1=NC=CN1C	<50%	N
COC(=O)/C=C/C1=CNC=N1	<5%	N
CO/C=C/C1C=C(C)N(C)N=1	0%	N
COC1=NN(C)C(=O)N1C=C	0%	N
C/C=C/C2=Nc1ccccc1O2	20%	N
C=CN1C(=O)N(C)N=C1Cl	0%	N
CN1C=CC(=N1)C2CCC(=O)C=2	0%	N
N#C/C=C/C1=CNc2ccccc12	0%	N
CC(=C)N2C(=O)Nc1ccccc12	80%	Y
Cl/C=C\C2Nc1ccccc1N=2	0%	N
OC1=CC(C=C(Cl)Cl)=NN1	<5%	N
CCOC(=O)/C=C/C1=CN=CN1C	67%	N
C=C(c1ccccc1O)N2C=CN=C2	98%	Y
COC(=O)C1=NNC=C1C(=C)Cl	0%	N
CN(C)/C=C/C2=Nc1ccccc1O2	0%	N
CO/C=C/C2=C1C=CC=CC1=NN2C	0%	N
[O-][N+](=O)/C=C\C2=Cc1ccccc1O2	0%	N
CN(C)/C=C/C2=Nc1ncccc1O2	0%	N
C=CN3c1ccccc1c2ccccc23	0%	N
CC(=C)C2=Cc1c(Cl)ncnc1N2	0%	N

CCCCCC(O)/C=C\C1NC=CN=1	0%	N
C=C(c1ccc(C#N)cc1)N2C=CN=C2	100%	Y
C=CN2C(=O)Oc1cc(Cl)cnc12	10%	N
CC3=NC2C=CCc1ccccc1C=2O3	0%	N
OC2C=CC1=NC=NN1c3ccccc23	50%	N
C=CN1C(C)=CC(=O)N1c2ccccc2	0%	N
CO/C=C/C1=CNN=C1c2ccccc2	0%	N
C=CC(=O)N1CC=C(C1)C2=CC=NN2C	85%	Y
O=C(/C=C/C1=CC=CO1)N2CCCCC2	100%	Y
C=CN1C=NC2=C1C(=O)N(C)C(=O)N2C	70%	Y
C=C(c1ccccc1)N2C=CN=C2	16%	N
CCOC1=CN=C(C=C(Cl)Cl)O1	0%	N
CN1N=C(C(Cl)=C1C=C)C(F)(F)F	0%	N
C=C(Cl)C1=C(Cl)N=C2C=CC=CN12	0%	N
Fc3ccc2/C(=C\C1=CN=CN1)/CCc2c3	0%	N
COC(=O)/C=C(\N1C=CN=C1)/C(F)(F)F	0%	N
C(=C/C1=CNc2ccccc12)\c3ccncc3	10%	N
CC1=NC=CN1C(=C)c2ccccc2	3%	N
CC(C)N1NC(C=C(Cl)Cl)=CC1=O	32%	N
C(=C/C2=Cc1ccccc1O2)/c3ccncc3	23%	N
CC(C)S(=O)(=O)/C(/C#N)=C/C1=CNC=N1	40%	N
CCN(C)CCC1=C(C=C)Nc2ccccc12	100%	Y
O=C2Nc1ccc(F)cc1/C/2=C/C3=CN=CN3	45%	N
CN3C2Oc1ccccc1C=2C=CS3(=O)=O	40%	N
O=C(NC1=O)[C@@]2([H])[C@]1([H])CC=CC2	0%	N
N#CC1=CCCCC1	5%	N
O=C1OC(C=C)=NC1(C)C	0%	N
CN1CC(C(OC)=O)=CCC1.Br	0%	N
OC(C1=CC=CC=C1)CC=C	20%	N
C=CCN1CCOCC1	0%	N
O=C(N1CCOCC1)C=C	84%	Y
O=C1C=C[C@@H]2[C@H]1OC(C)(C)O2	0%	N
CC1([N+]([O-])=CCC1)C	100%	Y
O=S(N1CC=CC1)(C2=CC=C(OC)C=C2)=O	0%	N
C1CCOC=C1	0%	N
OC1(C=C)COC1	5%	N
O=C(N1CCC=C1)OCC2=CC=CC=C2	17%	N
C=CCS(=O)(N)=O	16%	N
O=C1C=CCO1	0%	N
CC(CC1)=CCC1C(C)=C	18%	N
CC(OC(C)(O1)C)=CC1=O	0%	N
O=C1OCCC=C1	50%	N
O=C1C=CCCC1	97%	Y
O=C1CC=CC1	70%	Y
O=C1C=CCC1	20%	N
C=CCNC(NC1=CC=CC=C1)=O	0%	N
CC(C1C(C)(C2C1)C)=CC2=O	2%	N

O=C1NC2C=CC1C2	66%	Y
O[C@H]1[C@@H]2C=C[C@@H](C2)C1	0%	N
O=S(NCC=C)(C(C=C1)=CC=C1C)=O	30%	N
O[C@@H]1C=CO[C@H](CO)[C@H]1O	0%	N
O=C(N1O)C(C2CC3C=C2)C3C1=O	21%	N
OCC1=CC[C@@H](C(C)=C)CC1	5%	N
O=C(OC)C1=CCCC1	85%	Y
C1=CCOCOC1	0%	N
CC(C=C)(O)CC	0%	N
C1(N2CCOCC2)=CCCCC1	5%	N
O1C=CCC1	0%	N
C1COC=CO1	0%	N
OCC1OC=CCC1	0%	N
OC1CC=CC1	9%	N
N#CC1CC(C1)=C	<50%	N
O=C(N1CC(CC1)=C)OC(C)(C)C	0%	N
O=C(N1CC(C1)=C)OC(C)(C)C	0%	N
C=C1CCCC1	0%	N
CC1(C)C(C2)CCC2C1=C	0%	N
C1=CCCC1	0%	N
O=S1(CC=CC1)=O	0%	N
C=CC#N	<5%	N
O=C(OC)[C@@H](C1)C=C[C@@H]1NC(OC(C)(C)C)=O	0%	N
O=C1OCC2C(O3)C=CC3C21	66%	Y
OC1=CC=C(/C=C/C2=CC=CC=C2)C=C1	55%	N
O=C(OCC)C=CC1=CC=CC=C1	95%	Y
N#C/C(C#N)=C\C1=CC=CC=C1	90%	Y
O=C(C(C=C1)=CC=C1Cl)C=CC2=CC=CC=C2	100%	Y
N#CC=CC1=CC=CC=C1	43%	N
NC(C=CC1=CC=CC=C1)=O	100%	Y
O=C1NC(/C(S1)=C\C2=CC(O)=CC=C2)=O	0%	N
N#CC1=CC=C2OC(C)(C)C=CC2=C1	10%	N
O=S(C1=C2C=CC(N)=C1)(C=C2)=O	100%	Y
O=[N+](/C=C/C1=CC=C2C(OCO2)=C1)[O-]	0%	N
C=C(C1=CN=CC(Br)=C1)C	100%	Y
OC1=CC=C(/C=C/CO)C=C1OC	85%	Y
C=CC1=CC=NC=C1	85%	Y
C=C(C1=CC=CC=C1)C(OCC)=O	80%	Y
BrC1=CC=CC2=C1CC=C2	85%	Y
O=C1C=CC2=C1C=C(Br)C=C2	0%	N
Dienes		
N#CC1C=CC=CC=C1	80%	N
CCOC(/C=C/C=C/C)=O	80%	Y
C1(/C=CC=C1)=C2SCCS/2	<5%	N
C=CC=COC(C)=O	0%	N
C=C(O[Si](C)(C)C)C=C	0%	N
Pyrroles		

ClC(C=C1)=NC=C1CN2C=CC=C2	15%	N
O=[N+]([O-])C(C=CC=C1)=C1N2C=CC=C2	10%	N
O=S(N1C(C(C)=O)=CC=C1)(C2=CC=CC=C2)=O	10%	N
OC1=CC=C(N2C=CC=C2)C=C1	70%	Y
CC(C1=CN(S(C2=CC=CC=C2)(=O)=O)C=C1)=O	50%	N
O=S(N1C=CC=C1)(C2=CC=C(C)C=C2)=O	5%	N
CN1C(C#N)=CC(Br)=C1C	0%	N
CC(C1=CC=C(N2C(C)=CC=C2C)C=C1)=O	23%	N
O=CC1=CC=CN1C2=CC=CC=N2	<5%	N
BrC1=CC(N2C=CC=C2)=CC=C1	50%	N
COC(CC1=CC=CN1C)=O	<50%	N
CC(=O)C1=CC=CN1C	0%	N
O=C2CC(=O)C1=CC=CN1C2	<5%	N
CC2C1=CC=CN1CCN2C	20%	N
CCCN2CCN1C=CC=C1C2	15%	N
O=C2C1=CC=CN1c3cnccc23	<5%	N
CC2(C)C(=O)C=C(O)C1=CC=CN12	0%	N
O=C2Nc1ccccc1N3C=CC=C23	60%	Y
CCC(CC)N2CN1C=CC=C1C2=O	0%	N
CN1C=CC=C1C2(O)CCN(C)CC2	84%	Y
CN(C)CCNC(=O)C1=CC=CN1C	57%	N
O=C3CC1=CC=CN1c2ccccc2N3	90%	Y
O=C2Nc1ccccc1CN3C=CC=C23	20%	N
O=C2CNc1ccccc1N3C=CC=C23	0%	N
O=C2Nc1ccccc1CC3=CC=CN23	70%	Y
O=C2NCC1=CC=CN1c3ccccc23	75%	Y
CN(C)C2C1=CC=CN1c3ccccc23	0%	N
O=C2COc1ccccc1N3C=CC=C23	0%	N
O=C2NC1CCCCC1CN3C=CC=C23	0%	N
CN2CCN1C=CC=C1C23CCCCC3	65%	N
CN3CCN2CCCN1C=CC=C1C2C3	0%	N
CCOC(=O)C2CCN1C=CC=C1C2=O	?	N
CS(=O)(=O)N2CCC1=CC=CN1CC2	0%	N
C1CC[C@@]24[C@@H](C1)CCN2CCN3C=CC=C34	0%	N
CN2CCN1C=CC=C1C23CCCCC3=O	25%	N
O=C3c1ccccc1C4C2=CC=CN2CCN34	80%	Y
CN(C)C2=Nc1ccccc1CN3C=CC=C23	0%	N
CN(C)C2=NCc1ccccc1N3C=CC=C23	32%	N
COc1ccc2c(c1)NC(C)(C)C3=CC=CN23	35%	N
CN1C=CC=C1CN2CCS(=O)(=O)CC2	0%	N
O=C3CC1CC4(CCC1)C2=CC=CN2CCN34	100%	Y
O=C3C[C@@H]1CCCC[C@@]14C2=CC=CN2CCN34	100%	Y
C4CC[C@]23C1=CC=CN1CCCN2CC[C@@H]3C4	?	N
CN1CCC24C(C1)CCN2CCN3C=CC=C34	0%	N
CN(C)CC1=CC=CN1c2cccc(Cl)c2	0%	N
O=C3c1ccccc1C4C2=CC=CN2CCCN34	1%	N
O=C3Nc1ccccc1C4C2=CC=CN2CCN34	~70%	N

C1CN(CCO1)C3C2=CC=CN2c4ccccc34	0%	N
Cc1ccc3c(c1)OC(C)(C)C(=O)C2=CC=CN23	0%	N
CNC(=O)N3CC1=CC=CN1Cc2ccccc23	20%	N
COc1ccc2c(c1)N(C)C(C)(C)C3=CC=CN23	17%	N
CN(C)CCN1C=CC=C1C(=O)c2ccncc2	10%	N
CN1C=CC=C1C2CCCCN2C(=O)C3CC3	0%	N
CN1C=CC=C1C2=NN=C(O2)C3(C)CCOCC3	0%	N
O=C(C1=CC=CN1c2ccccc2)c3ccccn3	0%	N
C1C=CN(C=1)c2cnccn2	90%	Y
C(c1cccnc1)N2C=CC=C2	10%	N
CC1=NN=C(S1)N2C=CC=C2	0%	N
N#Cc1ccccc1N2C=CC=C2	76%	Y
C(CN1C=CC=C1)c2ccccn2	5%	N
CN1N=CC(C#N)=C1N2C=CC=C2	75%	Y
N#Cc2cccc(CN1C=CC=C1)c2	70%	Y
C1C=CN(C=1)C2=NNc3ccccc23	85%	Y
C1C=CN(C=1)c3ccc2N=COc2c3	95%	Y
C1CC1c2cc(ncn2)N3C=CC=C3	90%	Y
C1C=CN(C=1)C3C=CC2=NN=CN2N=3	15%	Y
CC(=O)c1ccc(cc1)N2C=CC=C2	100%	Y
CC(O)c1ccc(cc1)N2C=CC=C2	90%	Y
[O-][N+](=O)c1ccc(cc1)N2C=CC=C2	80%	Y
C1C=CN(C=1)c3ccc2nccnc2c3	5%	N
O=C2Nc1ccccc1N2N3C=CC=C3	1%	N
COc1ccnc(c1C#N)N2C=CC=C2	1%	N
OC1CCc2cc(ccc12)N3C=CC=C3	100%	Y
CC(=O)Nc1ccccc1N2C=CC=C2	1%	N
C1C=CN(C=1)C3=Nc2ccccc2S3	95%	Y
C1C=CN(C=1)C3=Nc2cccnc2S3	95%	Y
COC(=O)c1ccncc1N2C=CC=C2	10%	N
O=C1CCCN1CC#CCN2C=CC=C2	70%	Y
C1C=CN(C=1)c2ccc(cc2)C3C=CNN=3	95%	Y
CON(C)C(=O)CC(C)(C)N1C=CC=C1	20%	N
O=C2c1ccccc1N=CN2N3C=CC=C3	0%	N
CC1=COc2cc(c(C)cc12)N3C=CC=C3	10%	N
O=C1NC(=O)c2cc(ccc12)N3C=CC=C3	20%	N
Cc2cc(C)c1C(=NNc1n2)N3C=CC=C3	25%	N
O=C2COc1ccc(cc1N2)N3C=CC=C3	1%	N
CC(C)(O)Cc1ccc(cc1)N2C=CC=C2	90%	Y
OCCC(O)c1ccc(cc1)N2C=CC=C2	95%	Y
CC1C(=O)N(C)C(=O)N(C)C=1N2C=CC=C2	0%	N
CNC(=O)N2CCCC(CN1C=CC=C1)C2	60%	Y
C1C=CN(C=1)c2ccnc(n2)c3cccnc3	?	N
C1C=CN(C=1)c2ccnc(c2)c3ncccn3	0%	N
COCCNC(=O)CC(C)(C)N1C=CC=C1	20%	N
C(C1NN=NN=1)c2ccc(cc2)N3C=CC=C3	0%	N
COc1ccc(C#N)cc1C(=O)N2C=CC=C2	100%	Y

O=C2Nc1ccccc1CCC2N3C=CC=C3	60%	N
C1C=CN(C=1)c2ccccc2N3CCCCC3	0%	N
O=C(NC1CC1)c2ccc(nc2)N3C=CC=C3	95%	Y
CC(C)NC(=O)c1cccc(c1)N2C=CC=C2	40%	N
CC(CO)c1cc(C)c(cc1O)N2C=CC=C2	0%	N
COc1nc(NC(C)C)nc(n1)N2C=CC=C2	100%	Y
CS(=O)(=O)Nc1ccccc1N2C=CC=C2	30%	N
CCOC(=O)C1SC(=NC=1C)N2C=CC=C2	85%	Y
C1C=CN(C=1)c2ccc(cc2)Oc3ncccn3	95%	Y
OCC1C=NN(C=1N2C=CC=C2)c3ccccc3	0%	N
O=C(c1ccc(Cl)cc1Cl)N2C=CC=C2	90%	Y
CC(C)N2N=C(C)c1cc(cnc12)N3C=CC=C3	1%	N
COc1cc(cc(c1)C(F)(F)F)N2C=CC=C2	0%	N
C1C=CN(C=1)c3ccc2CC4(Cc2c3)OCCO4	95%	Y
O=C(N1CCCC1)C2(CCCCC2)N3C=CC=C3	0%	N
CN2CCC(c1cc(Cl)ccc12)N3C=CC=C3	25%	N
O=C(c1ccc(cc1)N2C=CC=C2)c3ccccn3	95%	Y
CN(C)CCc1ccc(c(Cl)c1)N2C=CC=C2	10%	N
CC(C)S(=O)(=O)c1ccc(cc1)N2C=CC=C2	20%	N
CC1ON=C(C=1C(=O)N2C=CC=C2)c3ccccc3	85%	Y
O=C(c1ccccc1)N2CCC(CC2)N3C=CC=C3	15%	N
C(CN1CCC(CC1)N2C=CC=C2)c3ccccc3	0%	N
C(CN1CCCC(C1)N2C=CC=C2)c3ccccc3	0%	N
CN1CCN(CC1)c2ccccc2CN3C=CC=C3	0%	N
O=C(c1ccc(cc1)N2C=CC=C2)N3CCOCC3	0%	N
COCCN1CC(CCS1(=O)=O)N2C=CC=C2	0%	N
CCN(CC)CCOc1ccccc1N2C=CC=C2	10%	N
FC(F)(F)C(c1ccc(Cl)cc1)N2C=CC=C2	0%	N
O=C(Nc1cc(Cl)ccc1N2C=CC=C2)C3CC3	0%	N
O=C2c1cccnc1Oc3ccc(cc23)N4C=CC=C4	80%	Y
CN3CCc2cc(c1ccccc1c2C3)N4C=CC=C4	15%	N
O=C(Nc1ccccc1N2C=CC=C2)c3cccnc3	0%	N
O=C(Oc1cccc(c1)N2C=CC=C2)c3ccccc3	80%	Y
OCC(O)COc1ccc(Cl)cc1N2C=CC=C2	0%	N
CC(=O)NC2=Cc1cc(ccc1OC2=O)N3C=CC=C3	0%	N
COC(=O)c2cc(O)c1ccc(cc1n2)N3C=CC=C3	40%	N
C1C=CN(C=1)c2cc(nc3ccccc23)c4ccccc4	40%	N
CN1CCC(O)(CC1)c2ccccc2CN3C=CC=C3	15%	N
COc1ccc(cc1)NC(=O)CC(C)(C)N2C=CC=C2	30%	N
C(CN1CCOCC1)Oc2ccccc2N3C=CC=C3	15%	N
CN2N=CC(C(=O)NC1CCCCC1)=C2N3C=CC=C3	0%	N
C(c1ccccc1)N3C(=Nc2ccccc23)N4C=CC=C4	40%	N
O=C(c1cc(F)ccc1N2C=CC=C2)N3CCOCC3	0%	N
O=C(Nc1ccccc1)Nc2ccccc2N3C=CC=C3	0%	N
CC1=CC(=NN1Cc2cccc(c2)N3C=CC=C3)C(N)=O	0%	N
CN1CCC(CC1)C3=Nc2ccccc2N3N4C=CC=C4	0%	N
CC(=O)c1ccc(c(c1)N2C=CC=C2)N3CCN(C)CC3	0%	N

O=C(CN1CCOCC1)Nc2ccccc2N3C=CC=C3	0%	N
CC(C)N1C[C@@H](O)[C@@H](C1)Oc2ccccc2N3C=CC=C3	5%	N
CC(=O)Nc1ccccc1N(CCN(C)C)N2C=CC=C2	75%	Y
Cc1cc(nc(n1)N(c2ccccc2)N3C=CC=C3)C4CC4	0%	N
O=S(=O)(Nc1ccc(cc1)N2C=CC=C2)c3ccccc3	60%	N
O=C(NC1CC1)N4CCCN3C=NC(CN2C=CC=C2)=C3C4	0%	N
CC(=O)C1C=CN(C)C=1	0%	N
N#CC1C=CN(C=1)c2ccc(cc2)[N+]([O-])=O	0%	N
OCC2C=CN(Cc1ccc(CO)cc1)C=2	50%	N
CN(C)C(=O)C1C=C(NN=1)C2C=CN(C)C=2	0%	N
CNC(=O)c1ccccc1N2C=CC(CO)=C2	0%	N
CSc1nccc(n1)N2C=CC(=C2)C(C)=O	30%	N
COc1ccc(cc1)N2C=CC(CC(O)CO)=C2	100%	Y
COC(=O)C1C=CN(C=1)c2cc(Cl)ccc2F	0%	N
CN(C)C(Cc1ccccc1)C(=O)C2C=CN(C)C=2	25%	N
CN(O)C(=O)/C=C/C1C=CN(C=1)C2CCCCCC2	60%	N
CN1C=CC(=C1)C2=CC(=NN2)C(=O)Nc3cccnc3	40%	N
Cc1cccc(C)c1N2C=CC(=C2)C3CC(=O)N(C)O3	0%	N
CN1C=CC(=C1)C2=CC(=NN2)C(=O)Nc3cccc(F)c3	90%	Y
COc1ccc(cc1OC)N2C=CC(CCNC(C)=O)=C2	100%	Y
CN(C)c1cccc(c1)Nc2nccc(n2)C3C=CN(C)C=3	78%	Y
O=S(=O)(C1C=CN(C=1)c2cccnn2)N3CCOCC3	1%	N

Table S3. Building Block Information of Aldehydes and Hydroxylamines for Nitrone Syntheses

Aldehyde ID	Structure	Hydroxylamine ID	Structure
Ald-1	COc1ccnc(C=O)n1	HA-1	ONC
Ald-2	Cn1cc(-c2n[nH]cc2C=O)cn1	HA-2	ONC(C)(C)C
Ald-3	N#Cc1cccc(C=O)n1	HA-3	ONC1CCCCC1
Ald-4	COc1cncc(CCC=O)c1	HA-4	ONC1CS(CC1)(=O)=O
Ald-5	CC(C)(C)OC(=O)N1CC(C=O)C1	HA-5	ONCc1ccccn1
Ald-6	CC(C)(C=O)N1CCOCC1	HA-6	O=C1CCNN1
Ald-7	N#Cc1ccc(CCC=O)cc1	HA-7	ONCc1c(Cl)cc(Cl)cc1
Ald-8	NS(=O)(=O)c1cc(C=O)co1	HA-8	O=C(CNO)N1CCOCC1
Ald-9	O=Cc1cncc2ccccc12		
Ald-10	Cn1cc(CCC=O)cn1		
Ald-11	O=Cc1cc(-c2ccccn2)n[nH]1		
Ald-12	O=CC1CC2(CC2)C1		
Ald-13	CS(=O)(=O)c1ccc(C=O)o1		
Ald-14	O=Cc1cnc(N2CCCC2)nc1		
Ald-15	COCC1(C=O)CCC1		
Ald-16	NS(=O)(=O)c1ccc(C=O)cc1		
Ald-17	O=CC1(c2ccccc2)CCOC1		
Ald-18	O=CC1(F)CC1		
Ald-19	O=CCc1ccc(F)cc1Cl		
Ald-20	O=CC1C[C@@H]2CC[C@H]1O2		
Ald-21	O=CC1(C(F)F)CC1		
Ald-22	N#CC1(C=O)CC1		
Ald-23	O=Cc1ccc2nccnc2c1		
Ald-24	O=Cc1nccn1Cc1ccccc1		
Ald-25	O=Cc1cc(C(F)(F)F)[nH]n1		
Ald-26	Cn1cc(-c2ncc(C=O)cn2)cn1		
Ald-27	O=Cc1ccc2cn[nH]c2c1		
Ald-28	O=Cc1nc(-c2ccccc2)c[nH]1		
Ald-29	Cc1ccc2occ(C=O)c(=O)c2c1		
Ald-30	O=CC1CCCOC1	**Aldehyde ID**	**Structure**
Ald-31	O=Cc1ccnn1-c1ccccn1	Ald-42	N#Cc1ccc2[nH]cc(C=O)c2c1
Ald-32	O=Cc1ccnc(-c2ccccc2)n1	Ald-43	CCCCC=O
Ald-33	CC(C)(C=O)S(C)(=O)=O	Ald-44	O=CC1CCOC1
Ald-34	O=Cc1ccc(-n2ccnc2)nc1	Ald-45	O=CC[C@H](O)[C@H](O)CO
Ald-35	CN(C)c1ncnc(Cl)c1C=O	Ald-46	O=CCCc1ccccc1
Ald-36	CS(=O)(=O)n1cccc1C=O	Ald-47	O=CCC1CCCCC1
Ald-37	Cn1cc(-c2cccc(C=O)c2)cn1	Ald-48	CC(C=O)c1ccccc1
Ald-38	O=CCC1CCCOC1	Ald-49	CCC(C=O)CC
Ald-39	CC1(C=O)COC1	Ald-50	O=CC1CC1
Ald-40	COC(C)(C)CC=O	Ald-51	O=CC1CCOCC1
Ald-41	O=Cc1ccc([N+](=O)[O-])cn1	Ald-52	O=CC1COC1

Table S4. Validation Data of Nitrones for Cycle-1

	HA-1	HA-2	HA-3	HA-4	HA-5	HA-6	HA-7	HA-8
**Ald-1**	90%	20%	10%	100%	0%	83%	100%	0%
**Ald-2**	0%	0%	<50%	50%	50%	<5%	50%	0%
**Ald-3**	0%	0%	0%	0%	0%	0%	0%	0%
**Ald-4**	0%	<5%	<5%	40%	40%	30%	60%	0%
**Ald-5**	90%	70%	90%	85%	95%	15%	95%	0%
**Ald-6**	0%	0%	0%	0%	0%	0%	0%	0%
**Ald-7**	15%	0%	20%	40%	40%	0%	30%	0%
**Ald-8**	0%	60%	80%	70%	70%	80%	70%	0%
**Ald-9**	80%	70%	70%	90%	97%	69%	90%	0%
**Ald-10**	<5%	0%	0%	0%	<50%	0%	0%	0%
**Ald-11**	0%	0%	0%	0%	0%	88%	0%	0%
**Ald-12**	0%	0%	0%	0%	0%	0%	0%	0%
**Ald-13**	N.D.	10%	60%	90%	80%	90%	85%	0%
**Ald-14**	80%	15%	35%	95%	80%	70%	83%	0%
**Ald-15**	N.D.	N.D.	0%	0%	0%	0%	0%	0%
**Ald-16**	100%	95%	92%	100%	95%	95%	96%	0%
**Ald-17**	N.D.	0%	0%	0%	0%	0%	0%	0%
**Ald-18**	0%	0%	0%	0%	0%	0%	0%	0%
**Ald-19**	0%	0%	0%	0%	40%	0%	0%	0%
**Ald-20**	0%	0%	0%	0%	0%	0%	0%	0%
**Ald-21**	0%	0%	0%	0%	0%	0%	0%	0%
**Ald-22**	N.D.	0%	0%	0%	0%	0%	0%	0%
**Ald-23**	30%	<50%	12%	86%	42%	95%	45%	0%
**Ald-24**	63%	<5%	71%	90%	N.D.	95%	87%	0%
**Ald-25**	0%	74%	80%	77%	50%	85%	90%	30%
**Ald-26**	0%	40%	55%	95%	81%	82%	93%	0%
**Ald-27**	100%	N.D.	53%	100%	95%	45%	90%	0%
**Ald-28**	0%	15%	12%	55%	N.D.	100%	40%	0%
**Ald-29**	0%	0%	0%	60%	0%	0%	0%	0%
**Ald-30**	0%	0%	0%	0%	0%	0%	0%	0%
**Ald-31**	88%	39%	48%	77%	85%	90%	100%	0%
**Ald-32**	80%	90%	100%	100%	100%	80%	100%	0%
**Ald-33**	0%	0%	0%	0%	0%	0%	0%	0%
**Ald-34**	55%	80%	95%	75%	100%	86%	95%	0%
**Ald-35**	0%	0%	0%	0%	0%	0%	0%	0%
**Ald-36**	0%	0%	0%	0%	<5%	0%	0%	0%
**Ald-37**	50%	N.D.	75%	70%	75%	0%	81%	0%
**Ald-38**	0%	0%	0%	0%	0%	0%	0%	0%
**Ald-39**	0%	0%	0%	0%	0%	0%	0%	0%
**Ald-40**	0%	0%	0%	0%	0%	0%	0%	0%
**Ald-41**	0%	N.D.	0%	0%	0%	0%	0%	0%
**Ald-42**	0%	0%	0%	0%	40%	0%	0%	0%
**Ald-43**	90%	0%	90%	100%	90%	20%	100%	N.P.
**Ald-44**	75%	N.P.	N.P.	100%	N.P.	N.P.	N.P.	

**Ald-45**	90%		100%	100%		75%	100%
**Ald-46**	90%		90%	100%		95%	100%
**Ald-47**	100%		100%	100%		100%	100%
**Ald-48**	75%		100%	100%		100%	95%
**Ald-49**	60%		90%	100%		95%	90%
**Ald-50**	75%		N.D.	100%		95%	90%
**Ald-51**	90%		100%	100%		80%	95%
**Ald-52**	90%		100%	100%		90%	100%

* Nitrones that were included in the DEL synthesis were highlighted by color.
* N.D. Not determined (low yield); N.P. Experiment not performed.

Table S5. Building Block Validation Data for Cycle-2 Amide Coupling Reactions

Structure	Yield	Included?
OC(=O)C1(CCOCC1)NC(=O)OCC3c2ccccc2c4ccccc34	45%	N
OC(=O)CC4(CNC(=O)OCC2c1ccccc1c3ccccc23)CCC4	97%	Y
OC(=O)CCCCNC(=O)OCC2c1ccccc1c3ccccc23	100%	Y
OC(=O)C(NC(=O)OCC2c1ccccc1c3ccccc23)C4CCC4	100%	Y
CC1(CCCCC1NC(=O)OCC3c2ccccc2c4ccccc34)C(O)=O	0%	N
OC(=O)C1=CC(=NN1)C2CN(C2)C(=O)OCC4c3ccccc3c5ccccc45	100%	Y
OC(=O)C4CCC(NC(=O)OCC2c1ccccc1c3ccccc23)c5ccccc45	65%	N
OC(=O)CC(NC(=O)OCC2c1ccccc1c3ccccc23)C4=CC=CS4	83%	Y
OC(=O)c1cccc3c1NC2CCN(CC=23)C(=O)OCC5c4ccccc4c6ccccc56	95%	Y
COc1cc(ccc1OCC(O)=O)C(C)NC(=O)OCC3c2ccccc2c4ccccc34	32%	N
OC(=O)COc4ccc(CCNC(=O)OCC2c1ccccc1c3ccccc23)cc4	90%	Y
OC(=O)C1CCCN1C2CCN(CC2)C(=O)OCC4c3ccccc3c5ccccc45	0%	N
OC(=O)CCCN(Cc1ccc(Cl)cc1)C(=O)OCC3c2ccccc2c4ccccc34	0%	N
OC(=O)C(NC(=O)OCC2c1ccccc1c3ccccc23)c4cc(F)ccc4F	0%	N
OC(=O)CN(CC(F)(F)F)C(=O)OCC2c1ccccc1c3ccccc23	38%	N
COc1ccc(cc1)C(NC(=O)OCC3c2ccccc2c4ccccc34)C(O)=O	95%	Y
OC(=O)Cc4ccc(CCNC(=O)OCC2c1ccccc1c3ccccc23)cc4	90%	Y
OC(=O)CCC1(CCOC1)NC(=O)OCC3c2ccccc2c4ccccc34	91%	Y
OC(=O)C5=CSC(Cc1ccc(cc1)NC(=O)OCC3c2ccccc2c4ccccc34)=N5	100%	Y
OC(=O)CN4C=CC(NC(=O)OCC2c1ccccc1c3ccccc23)=N4	1%	N
OC(=O)C4=CN(CCCCNC(=O)OCC2c1ccccc1c3ccccc23)N=N4	100%	Y
CC(NC(=O)OCC2c1ccccc1c3ccccc23)C4=NC=C(S4)C(O)=O	97%	Y
OC(=O)C26CC1CC(CC(C1)(C2)NC(=O)OCC4c3ccccc3c5ccccc45)C6	30%	N
OC(=O)COCCNC(=O)OCC2c1ccccc1c3ccccc23	77%	Y
CCC1(CCCN1C(=O)OCC3c2ccccc2c4ccccc34)C(O)=O	55%	N
OC(=O)C1CCCN(CC1)C(=O)OCC3c2ccccc2c4ccccc34	80%	Y
OC(=O)C1CC2C=CC1N(C2)C(=O)OCC4c3ccccc3c5ccccc45	79%	Y
CN4C=C(C(O)=O)C(CNC(=O)OCC2c1ccccc1c3ccccc23)=N4	100%	Y
OC(=O)C5CC1(CN(C1)C(=O)OCC3c2ccccc2c4ccccc34)ON=5	0%	N
C[C@@H]1C[C@@H](C[C@H](C)N1C(=O)OCC3c2ccccc2c4ccccc34)C(O)=O	88%	N*
CC4OC(CN(C)C(=O)OCC2c1ccccc1c3ccccc23)=CC=4C(O)=O	95%	Y
OC(=O)C5CCC4CCN(C(=O)OCC2c1ccccc1c3ccccc23)C4C5	75%	Y
CC(C)(NC(=O)OCC2c1ccccc1c3ccccc23)C4=NOC=C4C(O)=O	71%	Y
CN(CCc1ccccc1C(O)=O)C(=O)OCC3c2ccccc2c4ccccc34	77%	Y
COc4ccc(CNC(=O)OCC2c1ccccc1c3ccccc23)cc4C(O)=O	100%	Y
OC(=O)CN4CCCCC(NC(=O)OCC2c1ccccc1c3ccccc23)C4=O	98%	Y
OC(=O)c2ccc1OCCN(Cc1c2)C(=O)OCC4c3ccccc3c5ccccc45	100%	Y
OC(=O)C1CN(CC1c2ccccc2)C(=O)OCC4c3ccccc3c5ccccc45	32%	N
OC(=O)C1=CN(N=N1)C2CCCN(CC2)C(=O)OCC4c3ccccc3c5ccccc45	100%	Y
OC(=O)CCCCCNC(=O)OCC2c1ccccc1c3ccccc23	100%	Y
OC(=O)c5ccc1c(C=CN1CCCNC(=O)OCC3c2ccccc2c4ccccc34)c5	86%	Y
OC(=O)c1ccc(cc1)NC(=O)OCC3c2ccccc2c4ccccc34	90%	N*
OC(=O)C5=CN=C(CC1CCN(CC1)C(=O)OCC3c2ccccc2c4ccccc34)S5	87%	Y
OC(=O)C1=CSC(=N1)c5cccc(CNC(=O)OCC3c2ccccc2c4ccccc34)c5	88%	Y

OC(=O)CC1=CSC(=N1)C2CCN(CC2)C(=O)OCC4c3ccccc3c5ccccc45	100%	Y
COc3ccc2NC1C(C(O)=O)N(CCC=1c2c3)C(=O)OCC5c4ccccc4c6ccccc56	0%	N
CC1(CC1)C(NC(=O)OCC3c2ccccc2c4ccccc34)C(O)=O	75%	Y
OC(=O)CC(C1CCN(CC1)C(=O)OCC3c2ccccc2c4ccccc34)c5ccc(F)cc5	70%	Y
OC(=O)C1CN(CC12CCCC2)C(=O)OCC4c3ccccc3c5ccccc45	8%	N
OC(=O)C1CC12CCN(C2)C(=O)OCC4c3ccccc3c5ccccc45	100%	Y
OC(=O)c1ccc(nn1)N2CCN(CC2)C(=O)OCC4c3ccccc3c5ccccc45	90%	Y
OC(=O)C1CCN1C(=O)OCC3c2ccccc2c4ccccc34	23%	N
OC(=O)C2Sc1ccccc1C=2CNC(=O)OCC4c3ccccc3c5ccccc45	77%	Y
OC(=O)C1(CCCN(C1)C(=O)OCC3c2ccccc2c4ccccc34)C(F)(F)F	0%	N
OC(=O)c1cccc(c1)S(=O)(=O)N2CCN(CC2)C(=O)OCC4c3ccccc3c5ccccc45	84%	Y
OC(=O)C1CCN(C1)C(=O)OCC3c2ccccc2c4ccccc34	69%	Y
OC(=O)C1COC2(C1)CCN(CC2)C(=O)OCC4c3ccccc3c5ccccc45	80%	Y
OC(=O)C1SC=CC=1NC(=O)OCC3c2ccccc2c4ccccc34	20%	N
CN2CCC1(CN(CC1C2)C(=O)OCC4c3ccccc3c5ccccc45)C(O)=O	0%	N
OC(=O)COCC1CCN(CC1)C(=O)OCC3c2ccccc2c4ccccc34	100%	Y
CN1N=CC5=C1CN(C(=O)OCC3c2ccccc2c4ccccc34)C5C(O)=O	80%	Y
OC(=O)c1ccc(cc1)C2CCN(CC2)C(=O)OCC4c3ccccc3c5ccccc45	87%	Y
OC(=O)C4CC(O)(CNC(=O)OCC2c1ccccc1c3ccccc23)C4	85%	Y
OC(=O)C1(CCS(=O)(=O)CC1)NC(=O)OCC3c2ccccc2c4ccccc34	0%	N
CC4SC(CNC(=O)OCC2c1ccccc1c3ccccc23)=NC=4C(O)=O	98%	Y
OC(=O)C1CC(=O)N(C1)C2CN(C2)C(=O)OCC4c3ccccc3c5ccccc45	0%	N
CN1C=C(C=C1C(O)=O)NC(=O)OCC3c2ccccc2c4ccccc34	95%	Y
OC(=O)C1CC5(CN1C(=O)OCC3c2ccccc2c4ccccc34)CCOCC5	93%	Y
OC(=O)C1C=CN(N=1)C2CCN(CC2)C(=O)OCC4c3ccccc3c5ccccc45	100%	Y
CN(CCOc1ccc(nc1)C(O)=O)C(=O)OCC3c2ccccc2c4ccccc34	100%	Y
Cc4ccc(NC(=O)OCC2c1ccccc1c3ccccc23)c(C)c4C(O)=O	0%	N
OC(=O)CC4(CNC(=O)OCC2c1ccccc1c3ccccc23)CC4	100%	Y
OC(=O)C(CNC(=O)OCC2c1ccccc1c3ccccc23)Oc4ccc(F)cc4	0%	N
OC(=O)c5ccc4NC(CCNC(=O)OCC2c1ccccc1c3ccccc23)=Nc4c5	100%	Y
OC(=O)CC1(CCC1)NC(=O)OCC3c2ccccc2c4ccccc34	100%	Y
OC(=O)CSCCNC(=O)OCC2c1ccccc1c3ccccc23	100%	Y
OC(=O)c1ccc(cc1)N2CCC(CC2)NC(=O)OCC4c3ccccc3c5ccccc45	100%	Y
OC(=O)c1ccc(nc1)N2CCC(CC2)NC(=O)OCC4c3ccccc3c5ccccc45	84%	Y
CN(C(=O)OCC2c1ccccc1c3ccccc23)C(C)(C)C(O)=O	0%	N
CCC(C)(CNC(=O)OCC2c1ccccc1c3ccccc23)C(O)=O	0%	N
CC1SC=C(C(O)=O)C=1NC(=O)OCC3c2ccccc2c4ccccc34	94%	Y
OC(=O)c1ccc2c(c1)OCCN2C(=O)OCC4c3ccccc3c5ccccc45	89%	N*
OC(=O)C1=CN=C(O1)N2CCN(CC2)C(=O)OCC4c3ccccc3c5ccccc45	90%	Y
OC(=O)C5COC1(CN(C1)C(=O)OCC3c2ccccc2c4ccccc34)C5	80%	Y
OC(=O)CC(NC(=O)OCC2c1ccccc1c3ccccc23)c4ccncc4	100%	Y
OC(=O)C1(O)CCN(CC1)C(=O)OCC3c2ccccc2c4ccccc34	0%	N
OC(=O)c4cccc(CNC(=O)NCCNC(=O)OCC2c1ccccc1c3ccccc23)c4	95%	Y
OC(=O)C(NC(=O)OCC2c1ccccc1c3ccccc23)C4CCOC4	100%	Y
CCC(NC(=O)OCC2c1ccccc1c3ccccc23)(C(O)=O)c4ccccc4	30%	N
OC(=O)c2cc(cc1OCCOc12)NC(=O)OCC4c3ccccc3c5ccccc45	100%	Y
OC(=O)C1CCCCN1C(=O)OCC3c2ccccc2c4ccccc34	65%	Y

CC(C)N(CC(O)=O)C(=O)OCC2c1ccccc1c3ccccc23	82%	Y
OC(=O)C2C1CC(CC1)C2NC(=O)OCC4c3ccccc3c5ccccc45	85%	Y
OC(=O)C2c1ccccc1OCCN2C(=O)OCC4c3ccccc3c5ccccc45	0%	N
OC(=O)c2ccc1CN(CCOc1c2)C(=O)OCC4c3ccccc3c5ccccc45	70%	Y
OC(=O)C1CCC1NC(=O)OCC3c2ccccc2c4ccccc34	75%	Y
OC(=O)C[C@@H]2Cc1ccccc1CN2C(=O)OCC4c3ccccc3c5ccccc45	71%	Y
OC(=O)C2=Cc1cc(ccc1O2)NC(=O)OCC4c3ccccc3c5ccccc45	95%	Y
OC(=O)c4cccc(CCNC(=O)OCC2c1ccccc1c3ccccc23)c4	92%	Y
OC(=O)[C@H](CC1=CNc2ccc(O)cc12)NC(=O)OCC4c3ccccc3c5ccccc45	94%	Y
OC(=O)CCOCCOCCNC(=O)OCC2c1ccccc1c3ccccc23	100%	Y
OCC5CS[C@@H]4[C@H](NC(=O)OCC2c1ccccc1c3ccccc23)C(=O)N4C=5C(O)=O	N.D.	N
OC(=O)[C@H]1CCCN1C(=O)OCC3c2ccccc2c4ccccc34	84%	Y
OC(=O)C[C@H]1CCCN(C1)C(=O)OCC3c2ccccc2c4ccccc34	79%	Y
OC(=O)c2ccc1CN(Cc1c2)C(=O)OCC4c3ccccc3c5ccccc45	60%	N
OC(=O)[C@H]1CCCN(C1)C(=O)OCC3c2ccccc2c4ccccc34	79%	Y
OC(=O)CC1CCC(C1)NC(=O)OCC3c2ccccc2c4ccccc34	100%	Y
OC(=O)C(CN1C=CC(=N1)C(F)(F)F)NC(=O)OCC3c2ccccc2c4ccccc34	10%	N
C[C@H](OC(C)(C)C)[C@@H](NC(=O)OCC2c1ccccc1c3ccccc23)C(O)=O	83%	Y
OC(=O)C1(COC1)NC(=O)OCC3c2ccccc2c4ccccc34	0%	N
OC(=O)C1=NNC=C1NC(=O)OCC3c2ccccc2c4ccccc34	90%	Y
OC(=O)CNC(=O)OCC2c1ccccc1c3ccccc23	80%	Y
OC(=O)C(Cc1ccccc1)NC(=O)OCC3c2ccccc2c4ccccc34	26%	N
OC(=O)C[C@H](O)CNC(=O)OCC2c1ccccc1c3ccccc23	89%	Y
OC(=O)CCN(OCc1ccccc1)C(=O)OCC3c2ccccc2c4ccccc34	100%	Y
OC(=O)C=C1CN(C1)C(=O)OCC3c2ccccc2c4ccccc34	90%	Y
OC(=O)CCN(C(=O)OCC2c1ccccc1c3ccccc23)C4CC4	100%	Y
OC(=O)C(CC1=CNC=N1)NC(=O)OCC3c2ccccc2c4ccccc34	68%	N
OC(=O)C4CC(CNC(=O)OCC2c1ccccc1c3ccccc23)ON=4	17%	N
OC(=O)C1(CCCC1)NC(=O)OCC3c2ccccc2c4ccccc34	59%	N
OC(=O)CCOC1CN(C1)C(=O)OCC3c2ccccc2c4ccccc34	100%	Y
OC(=O)CN(C(=O)OCC2c1ccccc1c3ccccc23)C4CC4	80%	Y
OC(=O)c4ccc(CNC(=O)OCC2c1ccccc1c3ccccc23)nc4	90%	Y
OC(=O)c1ccc(cc1Cl)NC(=O)OCC3c2ccccc2c4ccccc34	90%	N*
OC(=O)[C@@H]1CCCN1C(=O)OCC3c2ccccc2c4ccccc34	75%	Y
CCC(C(O)=O)N(C)C(=O)OCC2c1ccccc1c3ccccc23	55%	N
CC(C)(CCC(O)=O)NC(=O)OCC2c1ccccc1c3ccccc23	100%	Y
NC(=O)C[C@H](NC(=O)OCC2c1ccccc1c3ccccc23)C(O)=O	18%	N
OC(=O)C1CCCCCCN1C(=O)OCC3c2ccccc2c4ccccc34	78%	Y
OC(=O)c5ccc1c(CCN1C(=O)OCC3c2ccccc2c4ccccc34)c5	82%	Y
OC(=O)C2Cc1ccccc1N2C(=O)OCC4c3ccccc3c5ccccc45	70%	N*
OC(=O)CN(C(=O)OCC2c1ccccc1c3ccccc23)c4ccccc4	77%	N*
OC(=O)c4cccc(CNC(=O)OCC2c1ccccc1c3ccccc23)c4	98%	Y
OC(=O)CC1CC2CCC(C1)N2C(=O)OCC4c3ccccc3c5ccccc45	90%	Y
OC(=O)C(NC(=O)OCC2c1ccccc1c3ccccc23)C4CC4	90%	Y
OC(=O)C[C@@H]1CCCN1C(=O)OCC3c2ccccc2c4ccccc34	90%	Y
OC(=O)C4=CSC(CCNC(=O)OCC2c1ccccc1c3ccccc23)=N4	100%	Y
OC(=O)C[C@H]1CCCN1C(=O)OCC3c2ccccc2c4ccccc34	90%	Y

OC(=O)[C@@H]4CC[C@@H](CNC(=O)OCC2c1ccccc1c3ccccc23)CC4	99%	Y
OC(=O)CN6CN(c1ccccc1)C2(CCN(CC2)C(=O)OCC4c3ccccc3c5ccccc45)C6=O	9%	N
CC1=NN(CC(O)=O)C(C)=C1CNC(=O)OCC3c2ccccc2c4ccccc34	0%	N
OC(=O)CCNC(=O)C1CCCN1C(=O)OCC3c2ccccc2c4ccccc34	100%	Y
CC(NC(=O)OCC2c1ccccc1c3ccccc23)(C(O)=O)C4CC4	0%	N
CC(C)(CC(O)=O)NC(=O)OCC2c1ccccc1c3ccccc23	100%	Y
OC(=O)CCCCCCCCCCCNC(=O)OCC2c1ccccc1c3ccccc23	85%	Y
OC(=O)C1=CSC(=N1)C2CCN(CC2)C(=O)OCC4c3ccccc3c5ccccc45	100%	Y
OC(=O)c1cccc(c1)NC(=O)OCC3c2ccccc2c4ccccc34	90%	Y
OC(=O)CNC(=O)CNC(=O)OCC2c1ccccc1c3ccccc23	90%	Y
C=CC[C@@H](NC(=O)OCC2c1ccccc1c3ccccc23)C(O)=O	77%	Y
OC(=O)C1C=NN(C=1)C2CCCN(C2)C(=O)OCC4c3ccccc3c5ccccc45	100%	Y
OC(=O)C4(CNC(=O)OCC2c1ccccc1c3ccccc23)CC4	82%	Y
OC(=O)C1NN=C2CCN(CC=12)C(=O)OCC4c3ccccc3c5ccccc45	100%	Y
OC(=O)CCCNC(=O)OCC2c1ccccc1c3ccccc23	100%	Y
CO[C@@H](CNC(=O)OCC2c1ccccc1c3ccccc23)C(O)=O	29%	N
OC(=O)C1C=CCN1C(=O)OCC3c2ccccc2c4ccccc34	88%	Y
OC(=O)[C@@H]2CSC(c1ccc(cc1)C(F)(F)F)N2C(=O)OCC4c3ccccc3c5ccccc45	40%	N
OC(=O)CC1CN(C1)C(=O)OCC3c2ccccc2c4ccccc34	98%	Y
OC(=O)C1(CCC1)C2CCN(CC=2)C(=O)OCC4c3ccccc3c5ccccc45	1%	N
OC(=O)C1CCN(CC1)C(=O)OCC3c2ccccc2c4ccccc34	88%	Y
OC(=O)C2C1CCCC1CN2C(=O)OCC4c3ccccc3c5ccccc45	85%	Y
OC(=O)C=C1CCN(CC1)C(=O)OCC3c2ccccc2c4ccccc34	90%	Y
OC(=O)CONC(=O)OCC2c1ccccc1c3ccccc23	15%	N
OC(=O)CCNC(=O)OCC2c1ccccc1c3ccccc23	100%	Y
[N-]=[N+]=N[C@H]1C[C@H](C(O)=O)N(C1)C(=O)OCC3c2ccccc2c4ccccc34	90%	Y
OC(=O)c4ccc(CONC(=O)OCC2c1ccccc1c3ccccc23)cc4	100%	Y
OC(=O)[C@@H]2C[C@@H]1CCC[C@@H]1N2C(=O)CNC(=O)OCC4c3ccccc3c5ccccc45	78%	Y
OC(=O)CCSCCCNC(=O)OCC2c1ccccc1c3ccccc23	81%	Y
OC(=O)CC1COCCC1NC(=O)OCC3c2ccccc2c4ccccc34	86%	Y
OC(=O)C1CCCC(C1)NC(=O)OCC3c2ccccc2c4ccccc34	25%	N
OC(=O)CC1COCCN1C(=O)OCC3c2ccccc2c4ccccc34	92%	Y
OC(=O)COCCOCCNC(=O)OCC2c1ccccc1c3ccccc23	100%	Y
OC(=O)c1ccccc1CNC(=O)OCC3c2ccccc2c4ccccc34	80%	Y
OC(=O)Cc4ccc(CNC(=O)OCC2c1ccccc1c3ccccc23)cc4	98%	Y
OC(=O)CCCCCCCCNC(=O)OCC2c1ccccc1c3ccccc23	100%	Y
OC(=O)/C=C/c1ccc(cc1)NC(=O)OCC3c2ccccc2c4ccccc34	95%	N*
OC(=O)c1ccc(cc1)c5ccc(CNC(=O)OCC3c2ccccc2c4ccccc34)cc5	60%	N
OC(=O)c1cccc(c1)c5ccc(CNC(=O)OCC3c2ccccc2c4ccccc34)cc5	79%	Y
OC(=O)c1ccccc1NC(=O)OCC3c2ccccc2c4ccccc34	20%	N
OC(=O)CCOCCNC(=O)OCC2c1ccccc1c3ccccc23	100%	Y
OC(=O)C1CN(CC1C(F)(F)F)C(=O)OCC3c2ccccc2c4ccccc34	0%	N
OC(=O)C12CCCC1CN(C2)C(=O)OCC4c3ccccc3c5ccccc45	0%	N
OC(=O)CC1CS(=O)(=O)CCN1C(=O)OCC3c2ccccc2c4ccccc34	88%	N*
OC(=O)CC1CC(C1)NC(=O)OCC3c2ccccc2c4ccccc34	100%	Y
OC(=O)CN1CCCN(CC1)C(=O)OCC3c2ccccc2c4ccccc34	68%	N
OC(=O)c1ccnc(c1)N2CCC(CC2)NC(=O)OCC4c3ccccc3c5ccccc45	80%	Y

OC(=O)C[C@@H]1CCCN(C1)C(=O)OCC3c2ccccc2c4ccccc34	100%	Y
OC(=O)C1=CSC(=N1)C2CCN(C2)C(=O)OCC4c3ccccc3c5ccccc45	100%	Y
C[C@H](NC(=O)OCC2c1ccccc1c3ccccc23)C(=O)N4[C@@H](COC4(C)C)C(O)=O	0%	N
OC(=O)CC1CN(CCO1)C(=O)OCC3c2ccccc2c4ccccc34	95%	Y
OC(=O)C1CN(C1)C(=O)OCC3c2ccccc2c4ccccc34	100%	Y
OC(=O)C1(CC1)NC(=O)OCC3c2ccccc2c4ccccc34	97%	Y
OC(=O)CC1(CCOCC1)NC(=O)OCC3c2ccccc2c4ccccc34	100%	Y
OC(=O)c4ccc(CNC(=O)OCC2c1ccccc1c3ccccc23)cc4	100%	Y
OC(=O)[C@@H]1CCCN(C1)C(=O)OCC3c2ccccc2c4ccccc34	84%	Y
OC(=O)C4COC(CNC(=O)OCC2c1ccccc1c3ccccc23)C4	80%	Y
OC(=O)C[C@@H](O)CNC(=O)OCC2c1ccccc1c3ccccc23	100%	Y
OC(=O)CCN1CCN(CC1)C(=O)OCC3c2ccccc2c4ccccc34	80%	Y
OC(=O)[C@H](CNC(=O)OCC2c1ccccc1c3ccccc23)NS(=O)(=O)c4ccccc4	0%	N
COC4CN(C(=O)OCC2c1ccccc1c3ccccc23)C(C)(C4)C(O)=O	0%	N

* Building blocks excluded due to low reactivity of deprotected amino group.

Appendix II: List of Building Blocks Included in the Final DELs

Table S6. List of building blocks included in the final DELs and associated DNA barcodes

Cycle #	Cycle ID	Structure	Barcode Sequence
1	1	CCOP(=O)(OCC)C1CC2=C(C)CNCC21	TACGACCCT
1	2	COc1cccc(C2CC3=C(C)CNCC32)c1	GCCGATTCT
1	3	CC1=C2CC(c3cc(C)ccc3C)C2CNC1	GCATACGCT
1	4	CC1=C2CC(c3ccccc3Br)C2CNC1	AGCCTGACT
1	5	CC1=C2CC(c3ccc(Cl)cc3)C2CNC1	GAACAGGCT
1	6	COC(=O)c1ccc(C2CC3=C(C)CNCC32)cc1	AGGCCATCT
1	7	CC1=C2CC(c3ccc(O)cc3)C2CNC1	CTTCGGTCT
1	8	CC(=O)OCc1ccc(C2CC3=C(C)CNCC32)cc1	GTAGACCCT
1	9	CC1=C2CC(c3ccc(CN(C)C)cc3)C2CNC1	GATTCCGCT
1	10	CC1=C2CC(c3cccc([N+](=O)[O-])c3)C2CNC1	TAGCTCCCT
1	11	COP(=O)(OC)C1CC2=C(C)CNCC21	ACAAGGCCT
1	12	CC1=C2CC(c3ccc(C#N)cc3)C2CNC1	GACAGGTCT
1	13	CC1=C2CC(c3ccc(C#N)cc3)C2CNC1	AGTAGCGCT
1	14	CC1=C2CC(c3cccc(C(F)(F)F)c3)C2CNC1	ATGGTCGCT
1	15	NS(=O)(=O)c1ccc(C2CC3CNCC(C)=C32)cc1	CTGGAAGCT
1	16	CC1=C2CC(c3cccc(Br)c3)C2CNC1	GCCTGATCT
1	17	Nc1ccccc1C1(C)CC2=C(C)CNCC21	AGCGGTTCT
1	18	CC1=C2CC(c3cccc(C=O)c3)C2CNC1	ACCACTCCT
1	19	CC1=C2CC(c3ccc(Oc4ccccc4)cc3)C2CNC1	CGTCAAGCT
1	20	CC1=C2CC(c3scnc3C)C2CNC1	CCTAACGCT
1	21	N(C(=O)C1CC2CNCC(C)=C21)C(CO)(CO)CO	TAGGCTCCT
1	22	CC1=C2CC(C)(C(=O)OCCO)C2CNC1	CGATGTGCT
1	23	CC1=C2CC3(CCOC3=O)C2CNC1	TACCGGACT
1	24	CC1=C2CC(C)(C(=O)OC(C)(C)C)C2CNC1	GTAACGGCT
1	25	NC(=O)C1(C)CC2=C(C)CNCC21	TTGGCAGCT
1	26	N(C(=O)C1CC2CNCC(C)=C21)C(C)C	CTATGGCCT
1	27	N(CCCN(C)C)C(=O)C1CC2CNCC(C)=C21	TAAGGCGCT
1	28	N(Cc1ccccc1)C(=O)C1CC2CNCC(C)=C21	TGCGTGTCT
1	29	N(C(C)=O)C1(C(=O)OC)CC2=C(C)CNCC21	GATGGAGCT
1	30	CC1=C2CC(C)(C(=O)OCCN(C)C)C2CNC1	CGGATTCCT
1	31	CC1=C2CC(C(=O)OCCCN(C)C)C2CNC1	AGCCAGTCT
1	32	CC1=C2CC(c3ccc(Br)cc3)C2CNC1	TGTCGCACT
1	33	CC1=C2CC(c3ccccc3Cl)C2CNC1	GAGTCACCT
1	34	CC1=C2CC(c3ccc([N+](=O)[O-])cc3)C2CNC1	AGCAACCCT
1	35	CC1=C2CC(c3ccc4ccccc4c3)C2CNC1	GTCAAGGCT
1	36	N(C(C)=O)C1CC2CNCC(C)=C21	CTCTAGCCT
1	37	CC1=C2CC(c3ccc(C)cc3)C2CNC1	ATGCCTCCT
1	38	CC1=C2CC(C)(c3ccccc3)C2CNC1	TCTCGGACT
1	39	CC1=C2CC(n3ccnc3)C2CNC1	GACACGACT
1	40	N(CC)C(=O)C1CC2CNCC(C)=C21	TGGAGCACT
1	41	CC1=C2CC(C(=O)N3CCOCC3)C2CNC1	TTCACGCCT
1	42	N(CC)C(=O)C1(C)CC2=C(C)CNCC21	GCATTGGCT

1	43	CC1=C2CC(n3nc(C)ccc3=O)C2CNC1	ACCGTAGCT
1	44	CC1=C2C(C#N)C(C)(c3ccccn3)C2CNC1	GTCGTCACT
1	45	CC1=C2CC(c3cnc(N(C)C)nc3)C2CNC1	TCGGAACCT
1	46	Nc1ncc(C2CC3CNCC(C)=C32)cn1	ACCAACGCT
1	47	CC(=O)C1C2=C(C)CNCC2C1c1ccccc1	GTTCCGACT
1	48	CC1=C2CC(C)(c3cn[nH]c(=O)c3)C2CNC1	AATCGGCCT
1	49	CC1=C2CC(c3ccc(Cl)nn3)C2CNC1	ATCGCACCT
1	50	CC1=C2CC(C)(c3nccc4[nH]ccc34)C2CNC1	GCGGAATCT
1	51	N1C(=O)Cc2cc(C3CC4CNCC(C)=C43)ccc21	CGATAGGCT
1	52	N1C(=O)Cc2cccc(C3CC4CNCC(C)=C43)c21	GACACTGCT
1	53	CC1=C2CC(c3ncnc4c3ncn4C)C2CNC1	GAACCAGCT
1	54	CC1=C2C(CNC1)C1(c3ccccc3)C(=O)OCC21	GTCCGTACT
1	55	CC1=C2CC3(CCCc4c(F)cccc43)C2CNC1	AGCCAACCT
1	56	CC1=C2CC(c3cc(F)cc4[nH]nnc34)C2CNC1	CTCCAGACT
1	57	COC(=O)C1C2=C(C)CNCC2C1c1ccncc1	CTACGCACT
1	58	COc1ccc(OC)c(C2CC3=C(C)CNCC32)c1	AGATGCCCT
1	59	CC1=C2CC(c3cncc(F)c3C(C)F)C2CNC1	AGAGACGCT
1	60	CC1=C2CC(c3ccc(-c4c[nH]cn4)cc3)C2CNC1	GACGCTTCT
1	61	CC1=C2CC(c3nc4ccccc4c(=O)[nH]3)C2CNC1	TTCGCCACT
1	62	NC(=O)C1C2=C(C)CNCC2C1(C#N)c1ccccn1	GAGGATGCT
1	63	N(c1ccc(C2(C)CC3=C(C)CNCC32)cn1)C1CC1	ATGTGCCCT
1	64	CC1=C2C(C#N)C(C3=Cc4ccccc4OC3)C2CNC1	AACGGCACT
1	65	COc1cccc2c1CCC1C3=C(C)CNCC3C21C#N	GCCTAGACT
1	66	CC(=O)C1C2=C(C)CNCC2C1c1ccc2c(c1)OCO2	GTAGGTGCT
1	67	CC1=C2CC(c3cc(F)cc4c3[nH]c(=O)n4C)C2CNC1	ACTCGCACT
1	68	CC1=C2CC(c3ccc(C#N)cc3)(n3cncn3)C2CNC1	GACCTGTCT
1	69	CC1=C2C(CNC1)C(C#N)(C(=O)C1CC1)C2c1ccccc1	CTGATGCCT
1	70	Nc1c(C#N)cnn1C1CC2CNCC(C)=C21	TGTGCGACT
1	71	CC1=C2CC(c3ccccc3O)(n3ccnc3)C2CNC1	GAGCACACT
1	72	CC1=C2CC(c3ccc(C#N)cc3)(n3ccnc3)C2CNC1	GTCACAGCT
1	73	CC1=C2CC(C(=O)N3CC=C(c4ccnn4C)C3)C2CNC1	TGGCAGACT
1	74	CC1=C2C(CNC1)C(c1ccco1)C2C(=O)N1CCCCC1	ACGCTACCT
1	75	CC1=C2CC(n3cnc4c3c(=O)n(C)c(=O)n4C)C2CNC1	CTGCTTGCT
1	76	CCN(C)CCc1c(C2CC3=C(C)CNCC32)[nH]c2ccccc12	ATAGCGGCT
1	77	CC1=C2C(CNC1)C1CCCC(=O)C21	ATTCCGCCT
1	78	CC1=C2C(CNC1)C1CC(=O)CC21	AGCACACCT
1	79	N1C(=O)C2CC1C1C3CNCC(C)=C3C21	TGACGTGCT
1	80	COC(=O)C12CCCC1C1=C(C)CNCC12	AGGAGTCCT
1	81	CCOC(=O)C1C2=C(C)CNCC2C1c1ccccc1	TGCGCTTCT
1	82	CC1=C2C(CNC1)C(C#N)(C#N)C2c1ccccc1	TTCAGCCCT
1	83	CC1=C2C(CNC1)C(c1ccccc1)C2C(=O)c1ccc(Cl)cc1	TCCAGGTCT
1	84	NC(=O)C1C2=C(C)CNCC2C1c1ccccc1	GCTACACCT
1	85	Nc1ccc2c(c1)S(=O)(=O)C1C3=C(C)CNCC3C21	TCACGGTCT
1	86	CC1=C2CC(C)(c3cncc(Br)c3)C2CNC1	GAAGTGCCT
1	87	COc1cc(C2C3=C(C)CNCC3C2CO)ccc1O	GCTCTACCT
1	88	CC1=C2CC(c3ccncc3)C2CNC1	CGTAGACCT
1	89	CCOC(=O)C1(c2ccccc2)CC2=C(C)CNCC21	TGATGCGCT

1	90	CC1=C2C(CNC1)C1c3cccc(Br)c3CC21	GTGCTACCT
1	91	CC1=C2C(CNC1)ON(C)C2c1c[nH]nc1C	AACGTGCCT
1	92	CC1=C2C(CNC1)ON(C)C2c1scnc1C	TATGCGGCT
1	93	CC1=C2C(CNC1)ON(C)C2c1ccccn1	TCGTGTGCT
1	94	CC1=C2C(CNC1)ON(C)C2c1cn(C)cn1	TCGTACCCT
1	95	CC1=C2C(CNC1)ON(C)C2c1nncn1C	GCAAGACCT
1	96	CC1=C2C(CNC1)ON(C)C2c1ncco1	TCAGGTGCT
1	97	CC1=C2C(CNC1)ON(C)C2c1ncccc1Cl	TACCGAGCT
1	98	CC1=C2C(CNC1)ON(C)C2c1cn[nH]c1	TCACCAGCT
1	99	CC1=C2C(CNC1)ON(C)C2c1cc(C)on1	ACTGCCACT
1	100	CC1=C2C(CNC1)ON(C)C2c1nc[nH]c1C	CGTTCGTCT
1	101	CC1=C2C(CNC1)ON(C)C2c1nccn1C	AACGAGGCT
1	102	CC1=C2C(CNC1)ON(C)C2c1cccnn1	GCGTAACCT
1	103	COc1cncc(C2C3=C(C)CNCC3ON2C)c1	GTGATGGCT
1	104	COc1ccnc(C2C3=C(C)CNCC3ON2C)n1	CGCTCTTCT
1	105	CC1=C2C(CNC1)ON(C)C2C1CN(C(=O)OC(C)(C)C)C1	AACAGCGCT
1	106	CC1=C2C(CNC1)ON(C)C2c1cncc2ccccc12	GCGAGTTCT
1	107	CC1=C2C(CNC1)ON(C)C2c1cnc(N2CCCC2)nc1	ACTCGGTCT
1	108	CC1=C2C(CNC1)ON(C)C2c1ccc(S(N)(=O)=O)cc1	CCATCTGCT
1	109	CC1=C2C(CNC1)ON(C)C2c1nccn1Cc1ccccc1	CTTCGTGCT
1	110	CC1=C2C(CNC1)ON(C)C2c1cc(C(F)(F)F)[nH]n1	CCAGCTACT
1	111	CC1=C2C(CNC1)ON(C)C2c1ccc2cn[nH]c2c1	ACAGCTCCT
1	112	CC1=C2C(CNC1)ON(C)C2c1ccnc(-c2ccccc2)n1	AGGTACGCT
1	113	CCCCC1C2=C(C)CNCC2ON1C	CCAAGCACT
1	114	CC1=C2C(CNC1)ON(C)C2C1CCOC1	CCATAGCCT
1	115	CC1=C2C(CNC1)ON(C)C2C[C@H](O)[C@H](O)CO	GCGAACACT
1	116	CC1=C2C(CNC1)ON(C)C2CCc1ccccc1	TGCGTAGCT
1	117	CC1=C2C(CNC1)ON(C)C2CC1CCCCC1	ACGGAGTCT
1	118	CC1=C2C(CNC1)ON(C)C2C(C)c1ccccc1	ACAGTGGCT
1	119	CC1=C2C(CNC1)ON(C)C2C1CC1	TCGTAGGCT
1	120	CC1=C2C(CNC1)ON(C)C2C1CCOCC1	TCCGAGACT
1	121	CC1=C2C(CNC1)ON(C)C2C1COC1	ACCGACACT
1	122	CCN1OC2CNCC(C)=C2C1c1c(C)noc1C	TTGCGACCT
1	123	CCN1OC2CNCC(C)=C2C1c1scnc1C	AGTCGAGCT
1	124	CCN1OC2CNCC(C)=C2C1c1ccccn1	TGGAGACCT
1	125	CCN1OC2CNCC(C)=C2C1c1cn(C)cn1	CCACATCCT
1	126	CCN1OC2CNCC(C)=C2C1c1ccn(C)n1	GCAGGTACT
1	127	CCN1OC2CNCC(C)=C2C1c1nncn1C	CTACGTCCT
1	128	CCN1OC2CNCC(C)=C2C1c1ncco1	TAGGTGGCT
1	129	CCN1OC2CNCC(C)=C2C1c1ncccc1Cl	GCAGACACT
1	130	CCN1OC2CNCC(C)=C2C1c1cn[nH]c1	GTGACTCCT
1	131	CCN1OC2CNCC(C)=C2C1c1cc(C)on1	CCACGTACT
1	132	CCN1OC2CNCC(C)=C2C1c1nc(C)oc1C	CTGGTCACT
1	133	CCN1OC2CNCC(C)=C2C1c1nccn1C	CTCCATCCT
1	134	CCN1OC2CNCC(C)=C2C1c1cccnn1	GAGCAACCT
1	135	CCN1OC2CNCC(C)=C2C1c1cncc(OC)c1	TCGCTCACT
1	136	CC1=C2C(CNC1)ON(C(C)(C)C)C2c1ccccn1	CCGATACCT

1	137	CC1=C2C(CNC1)ON(C(C)(C)C)C2c1cn(C)cn1	TCGGCTACT
1	138	CC1=C2C(CNC1)ON(C(C)(C)C)C2c1ccn(C)n1	GTGGTGACT
1	139	CC1=C2C(CNC1)ON(C(C)(C)C)C2c1ncco1	TTCACCGCT
1	140	CC1=C2C(CNC1)ON(C(C)(C)C)C2c1cc(C)on1	CTCGTGACT
1	141	CC1=C2C(CNC1)ON(C(C)(C)C)C2c1cccnn1	CGAGTTCCT
1	142	CC1=C2C(CNC1)ON(C(C)(C)C)C2c1ccc(S(N)(=O)=O)cc1	CCTACGACT
1	143	CC1=C2C(CNC1)ON(C(C)(C)C)C2c1ccnc(-c2ccccc2)n1	GCTGGTTCT
1	144	CC1=C2C(CNC1)ON(C1CCCC1)C2c1c(C)noc1C	CGTATCGCT
1	145	CC1=C2C(CNC1)ON(C1CCCC1)C2c1ccn(C)n1	GTCCTGACT
1	146	CC1=C2C(CNC1)ON(C1CCCC1)C2c1ncco1	CTGACCACT
1	147	CC1=C2C(CNC1)ON(C1CCCC1)C2c1nc(C)oc1C	GCGATAGCT
1	148	CC1=C2C(CNC1)ON(C1CCCC1)C2c1nccn1C	TAGTCGCCT
1	149	COc1cncc(C2C3=C(C)CNCC3ON2C2CCCC2)c1	CTGAGTGCT
1	150	CC1=C2C(CNC1)ON(C1CCCCC1)C2c1c(C)noc1C	CTGCCTACT
1	151	CC1=C2C(CNC1)ON(C1CCCCC1)C2c1ccccn1	CTGTCGTCT
1	152	CC1=C2C(CNC1)ON(C1CCCCC1)C2c1cn(C)cn1	GCATCTCCT
1	153	CC1=C2C(CNC1)ON(C1CCCCC1)C2c1ccn(C)n1	CTCGGATCT
1	154	CC1=C2C(CNC1)ON(C1CCCCC1)C2c1nncn1C	CCGTTCACT
1	155	CC1=C2C(CNC1)ON(C1CCCCC1)C2c1ncco1	ACGCACACT
1	156	CC1=C2C(CNC1)ON(C1CCCCC1)C2c1ncccc1Cl	CTACACCCT
1	157	CC1=C2C(CNC1)ON(C1CCCCC1)C2c1cc(C)on1	ATCGCGTCT
1	158	CC1=C2C(CNC1)ON(C1CCCCC1)C2c1nc(C)oc1C	TCATCCGCT
1	159	CC1=C2C(CNC1)ON(C1CCCCC1)C2c1nccn1C	GAACGACCT
1	160	CC1=C2C(CNC1)ON(C1CCCCC1)C2c1cccnn1	GTAGCTCCT
1	161	COc1cncc(C2C3=C(C)CNCC3ON2C2CCCCC2)c1	ACCTGGACT
1	162	CC1=C2C(CNC1)ON(C1CCCCC1)C2c1ccc(S(N)(=O)=O)cc1	CGTTCACCT
1	163	CC1=C2C(CNC1)ON(C1CCCCC1)C2c1nccn1Cc1ccccc1	ACCATCCCT
1	164	CC1=C2C(CNC1)ON(C1CCCCC1)C2c1cc(C(F)(F)F)[nH]n1	GCACAACCT
1	165	CC1=C2C(CNC1)ON(C1CCCCC1)C2c1ccnc(-c2ccccc2)n1	CCTTGGACT
1	166	CCCCC1C2=C(C)CNCC2ON1C1CCCCC1	TCCTAGCCT
1	167	CC1=C2C(CNC1)ON(C1CCCCC1)C2C1CCOC1	GTGGAACCT
1	168	CC1=C2C(CNC1)ON(C1CCCCC1)C2C[C@H](O)[C@H](O)CO	CGTGACACT
1	169	CC1=C2C(CNC1)ON(C1CCCCC1)C2CCc1ccccc1	GTTCGACCT
1	170	CC1=C2C(CNC1)ON(C1CCCCC1)C2CC1CCCCC1	CGTTGAGCT
1	171	CC1=C2C(CNC1)ON(C1CCCCC1)C2C(C)c1ccccc1	GACGTAGCT
1	172	CCC(CC)C1C2=C(C)CNCC2ON1C1CCCCC1	TCCACCACT
1	173	CC1=C2C(CNC1)ON(C1CCCCC1)C2C1CCOCC1	GTCTCTCCT
1	174	CC1=C2C(CNC1)ON(C1CCCCC1)C2C1COC1	AGTTGGCCT
1	175	CC1=C2C(CNC1)ON(Cc1ccccc1)C2c1c(C)noc1C	CCTGAGTCT
1	176	CC1=C2C(CNC1)ON(Cc1ccccc1)C2c1c[nH]nc1C	AGACCAGCT
1	177	CC1=C2C(CNC1)ON(Cc1ccccc1)C2c1scnc1C	CCACAAGCT
1	178	CC1=C2C(CNC1)ON(Cc1ccccc1)C2c1ccccn1	GCGACTACT
1	179	CC1=C2C(CNC1)ON(Cc1ccccc1)C2c1cn(C)cn1	AGTGGACCT
1	180	CC1=C2C(CNC1)ON(Cc1ccccc1)C2c1nncn1C	GCGTCTTCT
1	181	CC1=C2C(CNC1)ON(Cc1ccccc1)C2c1ncco1	TTGGAGCCT
1	182	CC1=C2C(CNC1)ON(Cc1ccccc1)C2c1ncccc1Cl	TGGTCTCCT
1	183	CC1=C2C(CNC1)ON(Cc1ccccc1)C2c1cn[nH]c1	TGCTTGGCT

1	184	CC1=C2C(CNC1)ON(Cc1ccccc1)C2c1cc(C)on1	ATCCGAGCT
1	185	CC1=C2C(CNC1)ON(Cc1ccccc1)C2c1nc(C)oc1C	AGGTGGTCT
1	186	CC1=C2C(CNC1)ON(Cc1ccccc1)C2c1nc[nH]c1C	TGTGCTGCT
1	187	CC1=C2C(CNC1)ON(Cc1ccccc1)C2c1nccn1C	CTCATGGCT
1	188	CC1=C2C(CNC1)ON(Cc1ccccc1)C2c1cccnn1	CGGTCATCT
1	189	COc1cncc(C2C3=C(C)CNCC3ON2Cc2ccccc2)c1	TCTGCTCCT
1	190	CC1=C2C(CNC1)ON(C1CCS(=O)(=O)C1)C2c1c(C)noc1C	GTGTGAGCT
1	191	CC1=C2C(CNC1)ON(C1CCS(=O)(=O)C1)C2c1c[nH]nc1C	CGTCCTTCT
1	192	CC1=C2C(CNC1)ON(C1CCS(=O)(=O)C1)C2c1scnc1C	GACATCGCT
1	193	CC1=C2C(CNC1)ON(C1CCS(=O)(=O)C1)C2c1ccccn1	CCTGTGACT
1	194	CC1=C2C(CNC1)ON(C1CCS(=O)(=O)C1)C2c1cn(C)cn1	CTCCGTTCT
1	195	CC1=C2C(CNC1)ON(C1CCS(=O)(=O)C1)C2c1ccn(C)n1	ATGGAGGCT
1	196	CC1=C2C(CNC1)ON(C1CCS(=O)(=O)C1)C2c1nncn1C	ATGCACCCT
1	197	CC1=C2C(CNC1)ON(C1CCS(=O)(=O)C1)C2c1ncco1	CGGAACACT
1	198	CC1=C2C(CNC1)ON(C1CCS(=O)(=O)C1)C2c1ncccc1Cl	GTCTCGTCT
1	199	CC1=C2C(CNC1)ON(C1CCS(=O)(=O)C1)C2c1cn[nH]c1	GATGTCCCT
1	200	CC1=C2C(CNC1)ON(C1CCS(=O)(=O)C1)C2c1cc(C)on1	CCTAGAGCT
1	201	CC1=C2C(CNC1)ON(C1CCS(=O)(=O)C1)C2c1nc(C)oc1C	CCGATTGCT
1	202	CC1=C2C(CNC1)ON(C1CCS(=O)(=O)C1)C2c1nc[nH]c1C	ACAGGACCT
1	203	CC1=C2C(CNC1)ON(C1CCS(=O)(=O)C1)C2c1nccn1C	GCGTTGTCT
1	204	CC1=C2C(CNC1)ON(C1CCS(=O)(=O)C1)C2c1cccnn1	CGGTATCCT
1	205	COc1cncc(C2C3=C(C)CNCC3ON2C2CCS(=O)(=O)C2)c1	TGTGTCGCT
1	206	COc1ccnc(C2C3=C(C)CNCC3ON2C2CCS(=O)(=O)C2)n1	GTTCACGCT
1	207	CC1=C2C(CNC1)ON(C1CCS(=O)(=O)C1)C2C1CN(C(=O)OC(C)(C)C)C1	TTCGTGCCT
1	208	CC1=C2C(CNC1)ON(C1CCS(=O)(=O)C1)C2c1cncc2ccccc12	ACACGAGCT
1	209	CC1=C2C(CNC1)ON(C1CCS(=O)(=O)C1)C2c1cnc(N2CCCC2)nc1	TTCCGTCCT
1	210	CC1=C2C(CNC1)ON(C1CCS(=O)(=O)C1)C2c1ccc(S(N)(=O)=O)cc1	GTGCAGTCT
1	211	CC1=C2C(CNC1)ON(C1CCS(=O)(=O)C1)C2c1ccc2nccnc2c1	AAGGCGACT
1	212	CC1=C2C(CNC1)ON(C1CCS(=O)(=O)C1)C2c1nccn1Cc1ccccc1	ATGACCGCT
1	213	CC1=C2C(CNC1)ON(C1CCS(=O)(=O)C1)C2c1cc(C(F)(F)F)[nH]n1	CCAATCCCT
1	214	CC1=C2C(CNC1)ON(C1CCS(=O)(=O)C1)C2c1ccc2cn[nH]c2c1	CGTGGTTCT
1	215	CC1=C2C(CNC1)ON(C1CCS(=O)(=O)C1)C2c1ccnc(-c2ccccc2)n1	TCATGCCCT
1	216	CCCCC1C2=C(C)CNCC2ON1C1CCS(=O)(=O)C1	CCAGAACCT
1	217	CC1=C2C(CNC1)ON(C1CCS(=O)(=O)C1)C2C1CCOC1	CTGATCGCT
1	218	CC1=C2C(CNC1)ON(C1CCS(=O)(=O)C1)C2C[C@H](O)[C@H](O)CO	CCTAGTCCT
1	219	CC1=C2C(CNC1)ON(C1CCS(=O)(=O)C1)C2CCc1ccccc1	GAGCGTTCT
1	220	CC1=C2C(CNC1)ON(C1CCS(=O)(=O)C1)C2CC1CCCCC1	TGTCAGCCT
1	221	CC1=C2C(CNC1)ON(C1CCS(=O)(=O)C1)C2C(C)c1ccccc1	TAGCAGCCT
1	222	CCC(CC)C1C2=C(C)CNCC2ON1C1CCS(=O)(=O)C1	GAGTAGGCT
1	223	CC1=C2C(CNC1)ON(C1CCS(=O)(=O)C1)C2C1CC1	TCACCTCCT
1	224	CC1=C2C(CNC1)ON(C1CCS(=O)(=O)C1)C2C1CCOCC1	TCTTGGCCT
1	225	CC1=C2C(CNC1)ON(C1CCS(=O)(=O)C1)C2C1COC1	AAGGAGCCT
1	226	CC1=C2C(CNC1)ON(Cc1ccccn1)C2c1cc(C)on1	CCAGGATCT
1	227	COc1cncc(C2C3=C(C)CNCC3ON2Cc2ccccn2)c1	ACGAGAGCT
1	228	CC1=C2C(CNC1)ON(Cc1ccccn1)C2C1CN(C(=O)OC(C)(C)C)C1	CCGTAAGCT
1	229	CC1=C2C(CNC1)ON(Cc1ccccn1)C2c1cncc2ccccc12	AGAGAGCCT
1	230	CC1=C2C(CNC1)ON(Cc1ccccn1)C2c1ccc(S(N)(=O)=O)cc1	TGCTCACCT

1	231	CC1=C2C(CNC1)ON(Cc1ccccn1)C2c1ccc2cn[nH]c2c1	TCTACGGCT
1	232	CC1=C2C(CNC1)ON(Cc1ccccn1)C2c1ccnc(-c2ccccc2)n1	GTTCTGGCT
1	233	CC1=C2C(CNC1)ON(Cc1ccccc1Cl)C2c1cnccc1N	CTGCCATCT
1	234	CC1=C2C(CNC1)ON(Cc1ccccc1Cl)C2c1c(C)noc1C	CTCGCTACT
1	235	CC1=C2C(CNC1)ON(Cc1ccccc1Cl)C2c1cn(C)cn1	TGAGAGGCT
1	236	CC1=C2C(CNC1)ON(Cc1ccccc1Cl)C2c1ccn(C)n1	ACCAGCACT
1	237	CC1=C2C(CNC1)ON(Cc1ccccc1Cl)C2c1nncn1C	TTCGCTCCT
1	238	CC1=C2C(CNC1)ON(Cc1ccccc1Cl)C2c1ncco1	CTTGCTCCT
1	239	CC1=C2C(CNC1)ON(Cc1ccccc1Cl)C2c1ncccc1Cl	ATCCTGGCT
1	240	CC1=C2C(CNC1)ON(Cc1ccccc1Cl)C2c1cn[nH]c1	TGAACCGCT
1	241	CC1=C2C(CNC1)ON(Cc1ccccc1Cl)C2c1cc(C)on1	ACAGTCCCT
1	242	CC1=C2C(CNC1)ON(Cc1ccccc1Cl)C2c1nc(C)oc1C	TTCGGTGCT
1	243	CC1=C2C(CNC1)ON(Cc1ccccc1Cl)C2c1nccn1C	GCTACCACT
1	244	CC1=C2C(CNC1)ON(Cc1ccccc1Cl)C2c1cccnn1	AACGGAGCT
1	245	COc1cncc(C2C3=C(C)CNCC3ON2Cc2ccccc2Cl)c1	CGAACCACT
1	246	CC1=C2C(CNC1)ON(Cc1ccc(Cl)cc1)C2c1ccccn1	TTACGGCCT
1	247	CC1=C2C(CNC1)ON(Cc1ccc(Cl)cc1)C2c1nncn1C	GTGTCTGCT
1	248	CC1=C2C(CNC1)ON(Cc1ccc(Cl)cc1)C2c1ncco1	TTGCTGGCT
1	249	CC1=C2C(CNC1)ON(Cc1ccc(Cl)cc1)C2c1ncccc1Cl	GCAGTGACT
1	250	CC1=C2C(CNC1)ON(Cc1ccc(Cl)cc1)C2c1nccn1C	ACGCTGACT
1	251	CC1=C2C(CNC1)ON(Cc1ccc(Cl)cc1)C2c1cncnc1	GTCAGTCCT
1	252	CC1=C2C(CNC1)ON(Cc1ccc(CO)cc1)C2c1c[nH]nc1C	AGCAGTGCT
1	253	CC1=C2C(CNC1)ON(Cc1ccc(CO)cc1)C2c1ccccn1	CGTTCCACT
1	254	CC1=C2C(CNC1)ON(Cc1ccc(CO)cc1)C2c1nncn1C	GCAGTTCCT
1	255	CC1=C2C(CNC1)ON(Cc1ccc(CO)cc1)C2c1ncco1	TCAGACGCT
1	256	CC1=C2C(CNC1)ON(Cc1ccc(CO)cc1)C2c1ncccc1Cl	TGAGGTCCT
1	257	COc1cncc(C2C3=C(C)CNCC3ON2Cc2ccc(CO)cc2)c1	GACCTTCCT
1	258	CC1=C2C(c3ccccn3)N3CCC(=O)N3C2CNC1	GCCTATGCT
1	259	CC1=C2C(c3ccn(C)n3)N3CCC(=O)N3C2CNC1	GGTATGGCT
1	260	CC1=C2C(c3nncn3C)N3CCC(=O)N3C2CNC1	TGCGTCACT
1	261	CC1=C2C(c3ncco3)N3CCC(=O)N3C2CNC1	CTACTGGCT
1	262	CC1=C2C(c3ncccc3Cl)N3CCC(=O)N3C2CNC1	CACGAAGCT
1	263	CC1=C2C(c3cc(C)on3)N3CCC(=O)N3C2CNC1	CGGTTGACT
1	264	CC1=C2C(c3nccn3C)N3CCC(=O)N3C2CNC1	TAGTGGCCT
1	265	CC1=C2C(c3cccnn3)N3CCC(=O)N3C2CNC1	AGTGAGCCT
1	266	COc1cncc(C2C3=C(C)CNCC3N3C(=O)CCN23)c1	CCTTGAGCT
1	267	COc1ccnc(C2C3=C(C)CNCC3N3C(=O)CCN23)n1	GTATCCGCT
1	268	CC1=C2C(c3cnc(N4CCCC4)nc3)N3CCC(=O)N3C2CNC1	CTCAGACCT
1	269	CC1=C2C(c3ccnc(-c4ccccc4)n3)N3CCC(=O)N3C2CNC1	GCTACTGCT
1	270	CC1=C2C(CCc3ccccc3)N3CCC(=O)N3C2CNC1	TCAGTCGCT
1	271	CC1=C2C(CC3CCCCC3)N3CCC(=O)N3C2CNC1	TACGGAGCT
1	272	CC1=C2C(C(C)c3ccccc3)N3CCC(=O)N3C2CNC1	GCGGTTACT
1	273	CCC(CC)C1C2=C(C)CNCC2N2C(=O)CCN12	ACTGTGCCT
1	274	CC1=C2C(C3CC3)N3CCC(=O)N3C2CNC1	CTCTGTGCT
1	275	CC1=C2C(C3CCOCC3)N3CCC(=O)N3C2CNC1	CATACCGCT
1	276	CC1=C2C(C3COC3)N3CCC(=O)N3C2CNC1	ACTGCTGCT
1	277	CC1=C2C(c3ccc(S(N)(=O)=O)cc3)N3CCC(=O)N3C2CNC1	TCAGAGGCT

1	278	CC1=C2C(c3nccn3Cc3ccccc3)N3CCC(=O)N3C2CNC1	CAAGCGACT
1	279	CC1=C2C(c3cc(C(F)(F)F)[nH]n3)N3CCC(=O)N3C2CNC1	GAGTTCCCT
1	280	CC1=C2C(CNC1)ON(Cc1ccc(Cl)cc1Cl)C2c1c(C)noc1C	TAGCGTGCT
1	281	CC1=C2C(CNC1)ON(Cc1ccc(Cl)cc1Cl)C2c1c[nH]nc1C	ATGACGCCT
1	282	CC1=C2C(CNC1)ON(Cc1ccc(Cl)cc1Cl)C2c1scnc1C	CCTTGTCCT
1	283	CC1=C2C(CNC1)ON(Cc1ccc(Cl)cc1Cl)C2c1ccccn1	CTCATCGCT
1	284	CC1=C2C(CNC1)ON(Cc1ccc(Cl)cc1Cl)C2c1cn(C)cn1	TCCTCTCCT
1	285	CC1=C2C(CNC1)ON(Cc1ccc(Cl)cc1Cl)C2c1ccn(C)n1	TCTCCTCCT
1	286	CC1=C2C(CNC1)ON(Cc1ccc(Cl)cc1Cl)C2c1nncn1C	CACCTACCT
1	287	CC1=C2C(CNC1)ON(Cc1ccc(Cl)cc1Cl)C2c1ncco1	GATGACGCT
1	288	CC1=C2C(CNC1)ON(Cc1ccc(Cl)cc1Cl)C2c1ncccc1Cl	GTGACGACT
1	289	CC1=C2C(CNC1)ON(Cc1ccc(Cl)cc1Cl)C2c1cn[nH]c1	GTCCACACT
1	290	CC1=C2C(CNC1)ON(Cc1ccc(Cl)cc1Cl)C2c1cc(C)on1	GAGCAGTCT
1	291	CC1=C2C(CNC1)ON(Cc1ccc(Cl)cc1Cl)C2c1nc(C)oc1C	CCAGAAGCT
1	292	CC1=C2C(CNC1)ON(Cc1ccc(Cl)cc1Cl)C2c1nc[nH]c1C	TTCGCTGCT
1	293	CC1=C2C(CNC1)ON(Cc1ccc(Cl)cc1Cl)C2c1nccn1C	CGGTTCACT
1	294	CC1=C2C(CNC1)ON(Cc1ccc(Cl)cc1Cl)C2c1cccnn1	TACGCACCT
1	295	COc1cncc(C2C3=C(C)CNCC3ON2Cc2ccc(Cl)cc2Cl)c1	GATGAGCCT
1	296	COc1ccnc(C2C3=C(C)CNCC3ON2Cc2ccc(Cl)cc2Cl)n1	TTGAGCGCT
1	297	CC1=C2C(CNC1)ON(Cc1ccc(Cl)cc1Cl)C2C1CN(C(=O)OC(C)(C)C)C1	CCGTTGTCT
1	298	CC1=C2C(CNC1)ON(Cc1ccc(Cl)cc1Cl)C2c1cncc2ccccc12	CTCGGTTCT
1	299	CC1=C2C(CNC1)ON(Cc1ccc(Cl)cc1Cl)C2c1cnc(N2CCCC2)nc1	CCTTGTGCT
1	300	CC1=C2C(CNC1)ON(Cc1ccc(Cl)cc1Cl)C2c1ccc(S(N)(=O)=O)cc1	TCTGCTGCT
1	301	CC1=C2C(CNC1)ON(Cc1ccc(Cl)cc1Cl)C2c1nccn1Cc1ccccc1	GACAGCACT
1	302	CC1=C2C(CNC1)ON(Cc1ccc(Cl)cc1Cl)C2c1cc(C(F)(F)F)[nH]n1	TGAGACCCT
1	303	CC1=C2C(CNC1)ON(Cc1ccc(Cl)cc1Cl)C2c1ccc2cn[nH]c2c1	CGTGAACCT
1	304	CC1=C2C(CNC1)ON(Cc1ccc(Cl)cc1Cl)C2c1ccnc(-c2ccccc2)n1	GTCTACGCT
1	305	CCCCC1C2=C(C)CNCC2ON1Cc1ccc(Cl)cc1Cl	CGATCACCT
1	306	CC1=C2C(CNC1)ON(Cc1ccc(Cl)cc1Cl)C2C1CCOC1	CCAGTTCCT
1	307	CC1=C2C(CNC1)ON(Cc1ccc(Cl)cc1Cl)C2C[C@H](O)[C@H](O)CO	CCGACTACT
1	308	CC1=C2C(CNC1)ON(Cc1ccc(Cl)cc1Cl)C2CCc1ccccc1	CGTACCACT
1	309	CC1=C2C(CNC1)ON(Cc1ccc(Cl)cc1Cl)C2CC1CCCCC1	CAGTGGACT
1	310	CC1=C2C(CNC1)ON(Cc1ccc(Cl)cc1Cl)C2C(C)c1ccccc1	AAGGACGCT
1	311	CCC(CC)C1C2=C(C)CNCC2ON1Cc1ccc(Cl)cc1Cl	AACCACCCT
1	312	CC1=C2C(CNC1)ON(Cc1ccc(Cl)cc1Cl)C2C1CC1	GCATCCACT
1	313	CC1=C2C(CNC1)ON(Cc1ccc(Cl)cc1Cl)C2C1CCOCC1	ACCAGGACT
1	314	CC1=C2C(CNC1)ON(Cc1ccc(Cl)cc1Cl)C2C1COC1	CGGTTGTCT
1	315	COc1cncc(C2C3=C(C)CNCC3ON2Cc2ccccc2OC)c1	AGGTAGCCT
1	316	CC1=C2C(CNC1)ON(C1CCCCC1)C2c1coc(S(N)(=O)=O)c1	AGGTAGGCT
1	317	CC1=C2C(CNC1)ON(C1CCS(=O)(=O)C1)C2c1coc(S(N)(=O)=O)c1	CACAGGACT
1	318	CC1=C2C(c3coc(S(N)(=O)=O)c3)N3CCC(=O)N3C2CNC1	CTCTCCACT
1	319	CC1=C2C(c3cc(-c4ccccn4)n[nH]3)N3CCC(=O)N3C2CNC1	GGAGATGCT
1	320	CC1=C2C(CNC1)ON(C1CCS(=O)(=O)C1)C2c1ccc(S(C)(=O)=O)o1	TCTTGCCCT
1	321	CC1=C2C(CNC1)ON(Cc1ccccn1)C2c1ccc(S(C)(=O)=O)o1	ATCCGTCCT
1	322	CC1=C2C(c3ccc(S(C)(=O)=O)o3)N3CCC(=O)N3C2CNC1	TTCGTGGCT
1	323	CC1=C2C(CNC1)ON(Cc1ccc(Cl)cc1Cl)C2c1ccc(S(C)(=O)=O)o1	CCAACCACT
1	324	CC1=C2C(CNC1)ON(C1CCS(=O)(=O)C1)C2c1cnc(-c2cnn(C)c2)nc1	GATGCGACT

1	325	CC1=C2C(CNC1)ON(Cc1ccccn1)C2c1cnc(-c2cnn(C)c2)nc1	ACTACGCCT
1	326	CC1=C2C(c3cnc(-c4cnn(C)c4)nc3)N3CCC(=O)N3C2CNC1	ATCTGCGCT
1	327	CC1=C2C(CNC1)ON(Cc1ccc(Cl)cc1Cl)C2c1cnc(-c2cnn(C)c2)nc1	GGAAGAGCT
1	328	CC1=C2C(c3nc(-c4ccccc4)c[nH]3)N3CCC(=O)N3C2CNC1	GTTGGACCT
1	329	CC1=C2C(CNC1)ON(C1CCS(=O)(=O)C1)C2c1ccnn1-c1ccccn1	ACACCAGCT
1	330	CC1=C2C(CNC1)ON(Cc1ccccn1)C2c1ccnn1-c1ccccn1	CGGTCTTCT
1	331	CC1=C2C(c3ccnn3-c3ccccn3)N3CCC(=O)N3C2CNC1	GGCAATCCT
1	332	CC1=C2C(CNC1)ON(Cc1ccc(Cl)cc1Cl)C2c1ccnn1-c1ccccn1	CTCGAACCT
1	333	CC1=C2C(CNC1)ON(C)C2c1ccc(-n2ccnc2)nc1	CTGAGGTCT
1	334	CC1=C2C(CNC1)ON(C1CCCCC1)C2c1ccc(-n2ccnc2)nc1	TCGCACACT
1	335	CC1=C2C(CNC1)ON(C1CCS(=O)(=O)C1)C2c1ccc(-n2ccnc2)nc1	CGTGTCACT
1	336	CC1=C2C(CNC1)ON(Cc1ccccn1)C2c1ccc(-n2ccnc2)nc1	CCTGTGTCT
1	337	CC1=C2C(c3ccc(-n4ccnc4)nc3)N3CCC(=O)N3C2CNC1	TTGGAGGCT
1	338	CC1=C2C(CNC1)ON(Cc1ccc(Cl)cc1Cl)C2c1ccc(-n2ccnc2)nc1	GTCTTGCCT
1	339	CC1=C2C(CNC1)ON(C)C2c1cccc(-c2cnn(C)c2)c1	CTGTACCCT
1	340	CC1=C2C(CNC1)ON(C1CCCCC1)C2c1cccc(-c2cnn(C)c2)c1	CACCGATCT
1	341	CC1=C2C(CNC1)ON(C1CCS(=O)(=O)C1)C2c1cccc(-c2cnn(C)c2)c1	CGACACACT
1	342	CC1=C2C(CNC1)ON(Cc1ccccn1)C2c1cccc(-c2cnn(C)c2)c1	AAGAGCGCT
1	343	CC1=C2C(CNC1)ON(Cc1ccc(Cl)cc1Cl)C2c1cccc(-c2cnn(C)c2)c1	CGGTAAGCT
1	344	CC1=C2C(CNC1)C1C=CC2N1c1cccc(C=O)c1	TGGAGTGCT
1	345	CC1=C2C(CNC1)C1C=CC2N1c1ccc([N+](=O)[O-])cc1	GAACCGTCT
1	346	CC1=C2C(CNC1)C1C=CC2N1c1ccc(O)cc1	GCACGTTCT
1	347	CC1=C2C(CNC1)C1C=CC23CNC(=O)c2ccccc2N13	AAGCCGTCT
1	348	CC1=C2C(CNC1)C1C=CC23C2c4ccccc4C(=O)N2CCN13	ACGACCACT
1	349	CC1=C2C(CNC1)C1C=CC23N1CCN1C(=O)CC2CCCC13C2	TAGTGCCCT
1	350	CC1=C2C(CNC1)C1C=CC23N1CCN1C(=O)C[C@@H]2CCCC[C@@]213	ACGCGATCT
1	351	CC1=C2C(CNC1)C1C=CC2N1c1cnccn1	TCTCCACCT
1	352	CC1=C2C(CNC1)C1C=CC2N1c1n[nH]c2ccccc12	TCCAGACCT
1	353	CC1=C2C(CNC1)C1C=CC2N1c1ccc2ncoc2c1	CCTCTGTCT
1	354	CC1=C2C(CNC1)C1C=CC2N1c1cc(C2CC2)ncn1	TCCATCGCT
1	355	CC(=O)c1ccc(N2C3C=CC2C2CNCC(C)=C23)cc1	CCATCACCT
1	356	CC1=C2C(CNC1)C1C=CC2N1c1ccc(C(C)O)cc1	GCAGCTTCT
1	357	CC1=C2C(CNC1)C1C=CC2N1c1ccc([N+](=O)[O-])cc1	AGTGTCCCT
1	358	CC1=C2C(CNC1)C1C=CC2N1c1ccc2c(c1)CCC2O	CGTCTGACT
1	359	CC1=C2C(CNC1)C1C=CC2N1c1nc2ccccc2s1	ACAGCGTCT
1	360	CC1=C2C(CNC1)C1C=CC2N1c1nc2cccnc2s1	ACTGGTGCT
1	361	CC1=C2C(CNC1)C1C=CC2N1CC#CCN1CCCC1=O	CTCAGTCCT
1	362	CC1=C2C(CNC1)C1C=CC2N1c1ccc(-c2cc[nH]n2)cc1	GTGATCCCT
1	363	COc1ccc(C#N)cc1C(=O)N1C2C=CC1C1CNCC(C)=C12	GATTGGCCT
1	364	CC1=C2C(CNC1)C1C=CC2N1c1ccc(C(=O)NC2CC2)cn1	ATCGAGCCT
1	365	COc1nc(NC(C)C)nc(N2C3C=CC2C2CNCC(C)=C23)n1	GACTCGTCT
1	366	CCOC(=O)c1sc(N2C3C=CC2C2CNCC(C)=C23)nc1C	CGGATGACT
1	367	CC1=C2C(CNC1)C1C=CC2N1c1ccc(Oc2ncccn2)cc1	GACTGTGCT
1	368	COc1ccc(N2C3C=C(CC(O)CO)C2C2CNCC(C)=C23)cc1	GTAGGACCT
1	369	CC1=C2C(CNC1)C1C(c3cc(C(=O)Nc4cccc(F)c4)n[nH]3)=CC2N1C	CCGACTTCT
1	370	COc1ccc(N2C3C=C(CCNC(C)=O)C2C2CNCC(C)=C23)cc1OC	AAGCCAGCT
1	371	CC1=C2C3C=CC(CC3)C2CNC1	GACAGACCT

1	372	CCOC(=O)C1C=CC(C)C2=C(C)CNCC21	CCGTGTACT
1	373	CC1=C2C(CNC1)ON1C2CCC1(C)C	GATGGCACT
2	1	NCC1(CC(=O)O)CCC1	GATAGGCAC
2	2	NCCCCC(=O)O	CACTTCGAC
2	3	NC(C(=O)O)C1CCC1	GCATGAGAC
2	4	O=C(O)c1cc(C2CNC2)n[nH]1	GCACCAAAC
2	5	NC(CC(=O)O)c1cccs1	GAGAGTGAC
2	6	O=C(O)c1cccc2c3c([nH]c12)CCNC3	ATGGACGAC
2	7	NCCc1ccc(OCC(=O)O)cc1	AGGTGCTAC
2	8	COc1ccc(C(N)C(=O)O)cc1	CCTGTTGAC
2	9	NCCc1ccc(CC(=O)O)cc1	CACCTGAAC
2	10	NC1(CCC(=O)O)CCOC1	CGGTCTAAC
2	11	Nc1ccc(Cc2nc(C(=O)O)cs2)cc1	GTTAGCCAC
2	12	NCCCCn1cc(C(=O)O)nn1	GAAGCCAAC
2	13	CC(N)c1ncc(C(=O)O)s1	CGATCTCAC
2	14	NCCOCC(=O)O	GTGAGTCAC
2	15	O=C(O)C1CCCNCC1	TGAGCTGAC
2	16	O=C(O)C1CC2C=CC1NC2	CGCATAGAC
2	17	Cn1cc(C(=O)O)c(CN)n1	CAGACAGAC
2	18	CNCc1cc(C(=O)O)c(C)o1	CGGATCAAC
2	19	O=C(O)C1CCC2CCNC2C1	GAACGTGAC
2	20	CC(C)(N)c1nocc1C(=O)O	CGACCTAAC
2	21	CNCCc1ccccc1C(=O)O	CAGGCAAAC
2	22	COc1ccc(CN)cc1C(=O)O	CAAGCCTAC
2	23	NC1CCCCN(CC(=O)O)C1=O	ACGCCAAAC
2	24	O=C(O)c1ccc2c(c1)CNCCO2	GAACCTCAC
2	25	O=C(O)c1cn(C2CCCNCC2)nn1	AGGTCAGAC
2	26	NCCCCCC(=O)O	CAATGCCAC
2	27	NCCCn1ccc2cc(C(=O)O)ccc21	GACGATGAC
2	28	O=C(O)c1cnc(CC2CCNCC2)s1	AAGGTCCAC
2	29	NCc1cccc(-c2nc(C(=O)O)cs2)c1	CAAGCTCAC
2	30	O=C(O)Cc1csc(C2CCNCC2)n1	GTCGTGAAC
2	31	CC1(C(N)C(=O)O)CC1	AGGATCGAC
2	32	O=C(O)CC(c1ccc(F)cc1)C1CCNCC1	GTTGCTCAC
2	33	O=C(O)C1CC12CCNC2	AGCGAGAAC
2	34	O=C(O)c1ccc(N2CCNCC2)nn1	CCGAGATAC
2	35	NCc1c(C(=O)O)sc2ccccc12	GTAGCTGAC
2	36	O=C(O)c1cccc(S(=O)(=O)N2CCNCC2)c1	AACTCGCAC
2	37	O=C(O)C1CCNC1	ATTCGGCAC
2	38	O=C(O)C1COC2(CCNCC2)C1	GTGTACGAC
2	39	O=C(O)COCC1CCNCC1	CGATGCTAC
2	40	Cn1ncc2c1CNC2C(=O)O	GAATCGCAC
2	41	O=C(O)c1ccc(C2CCNCC2)cc1	CCAAGGTAC
2	42	NCC1(O)CC(C(=O)O)C1	AGGACTCAC
2	43	Cc1sc(CN)nc1C(=O)O	CATCTCGAC
2	44	Cn1cc(N)cc1C(=O)O	CGAGATGAC
2	45	O=C(O)C1CC2(CCOCC2)CN1	GATGACCAC

2	46	O=C(O)c1ccn(C2CCNCC2)n1	CAGTCCTAC
2	47	CNCCOc1ccc(C(=O)O)nc1	GTTGTCGAC
2	48	NCC1(CC(=O)O)CC1	AGACTCGAC
2	49	NCCc1nc2cc(C(=O)O)ccc2[nH]1	ACGACCTAC
2	50	NC1(CC(=O)O)CCC1	CAGAGGAAC
2	51	NCCSCC(=O)O	AGCATCCAC
2	52	NC1CCN(c2ccc(C(=O)O)cc2)CC1	TCCTCAGAC
2	53	NC1CCN(c2ccc(C(=O)O)cn2)CC1	GCCAGTAAC
2	54	Cc1scc(C(=O)O)c1N	TCTCCAGAC
2	55	O=C(O)c1cnc(N2CCNCC2)o1	CAGTGGTAC
2	56	O=C(O)C1COC2(CNC2)C1	ATGTCGCAC
2	57	NC(CC(=O)O)c1ccncc1	TGGACAGAC
2	58	NCCNC(=O)NCc1cccc(C(=O)O)c1	TGGAAGCAC
2	59	NC(C(=O)O)C1CCOC1	CGTCACAAC
2	60	Nc1cc2c(c(C(=O)O)c1)OCCO2	CCGTGAAAC
2	61	O=C(O)C1CCCCN1	GACTGCTAC
2	62	CC(C)NCC(=O)O	TTCGGTCAC
2	63	NC1C2CCC(C2)C1C(=O)O	GCCATTGAC
2	64	O=C(O)c1ccc2c(c1)OCCNC2	CAAGGCAAC
2	65	NC1CCC1C(=O)O	CCGTTGAAC
2	66	O=C(O)C[C@@H]1Cc2ccccc2CN1	TGCCAGTAC
2	67	Nc1ccc2oc(C(=O)O)cc2c1	CGTGTGTAC
2	68	NCCc1cccc(C(=O)O)c1	ATCGCTGAC
2	69	N[C@@H](Cc1c[nH]c2ccc(O)cc12)C(=O)O	ACCTTCCAC
2	70	NCCOCCOCCC(=O)O	GCACTCTAC
2	71	O=C(O)[C@H]1CCCN1	TGGAGTCAC
2	72	O=C(O)C[C@H]1CCCNC1	TGGCAAGAC
2	73	O=C(O)[C@H]1CCCNC1	AGCGGAAAC
2	74	NC1CCC(CC(=O)O)C1	CATTCCGAC
2	75	C[C@H](OC(C)(C)C)[C@@H](N)C(=O)O	TACAGCCAC
2	76	Nc1c[nH]nc1C(=O)O	CAGGTTCAC
2	77	NCC(=O)O	ACTCCTGAC
2	78	NC[C@@H](O)CC(=O)O	TACCTGCAC
2	79	O=C(O)CCNOCc1ccccc1	CCGTCATAC
2	80	O=C(O)C=C1CNC1	TCCTCCAAC
2	81	O=C(O)CCNC1CC1	GTCTGTCAC
2	82	O=C(O)CCOC1CNC1	CGTGTAGAC
2	83	O=C(O)CNC1CC1	CCAAGTGAC
2	84	NCc1ccc(C(=O)O)cn1	AACTCCGAC
2	85	O=C(O)[C@@H]1CCCN1	GTCGGAAAC
2	86	CC(C)(N)CCC(=O)O	ATGCCGAAC
2	87	O=C(O)C1CCCCCCN1	GCTTCCAAC
2	88	O=C(O)c1ccc2c(c1)CCN2	AGACGCTAC
2	89	NCc1cccc(C(=O)O)c1	GCAACAGAC
2	90	O=C(O)CC1CC2CCC(C1)N2	CGAACGAAC
2	91	NC(C(=O)O)C1CC1	CGATGGAAC
2	92	O=C(O)C[C@@H]1CCCN1	GTGTGGAAC

2	93	NCCc1nc(C(=O)O)cs1	ATTGGCGAC
2	94	O=C(O)C[C@H]1CCCN1	AGGCAGAAC
2	95	NC[C@H]1CC[C@H](C(=O)O)CC1	TAGCCTCAC
2	96	O=C(O)CCNC(=O)C1CCCN1	ACCTCTGAC
2	97	CC(C)(N)CC(=O)O	GCAACCTAC
2	98	NCCCCCCCCCCCC(=O)O	ATGTCCGAC
2	99	O=C(O)c1csc(C2CCNCC2)n1	CGGAGTAAC
2	100	Nc1cccc(C(=O)O)c1	TGGCTTGAC
2	101	NCC(=O)NCC(=O)O	CGAGACAAC
2	102	C=CC[C@@H](N)C(=O)O	TTGTGCGAC
2	103	O=C(O)c1cnn(C2CCCNC2)c1	GAGGAGTAC
2	104	NCC1(C(=O)O)CC1	ATGCTCGAC
2	105	O=C(O)c1[nH]nc2c1CNCC2	GTGGTTCAC
2	106	NCCCC(=O)O	CCAGCAAAC
2	107	O=C(O)C1C=CCN1	TCTGTGCAC
2	108	O=C(O)CC1CNC1	ACTGTCGAC
2	109	O=C(O)C1CCNCC1	TTGCGGTAC
2	110	O=C(O)C1NCC2CCCC21	GCATCTGAC
2	111	O=C(O)C=C1CCNCC1	CACGACAAC
2	112	NCCC(=O)O	TTGCTCCAC
2	113	[N-]=[N+]=N[C@@H]1CN[C@@H](C(=O)O)C1	ACCTAGCAC
2	114	NOCc1ccc(C(=O)O)cc1	GTGGTCAAC
2	115	NCC(=O)N1[C@H](C(=O)O)C[C@@H]2CCC[C@@H]21	TCGTCTGAC
2	116	NCCCSCCC(=O)O	ACGCATCAC
2	117	NC1CCOCC1CC(=O)O	TGCCGTAAC
2	118	O=C(O)CC1COCCN1	TGCTCGTAC
2	119	NCCOCCOCC(=O)O	TGAGCGAAC
2	120	NCc1ccccc1C(=O)O	TGTCGTGAC
2	121	NCc1ccc(CC(=O)O)cc1	GCGTGTAAC
2	122	NCCCCCCCCC(=O)O	TGGCCTTAC
2	123	NCc1ccc(-c2cccc(C(=O)O)c2)cc1	GTGGACTAC
2	124	NCCOCCC(=O)O	GCAGTCAAC
2	125	NC1CC(CC(=O)O)C1	CGGTATGAC
2	126	NC1CCN(c2cc(C(=O)O)ccn2)CC1	AAGCGGTAC
2	127	O=C(O)C[C@@H]1CCCNC1	ATCCTCCAC
2	128	O=C(O)c1csc(C2CCNC2)n1	CCAGCTTAC
2	129	O=C(O)CC1CNCCO1	CGAAGTCAC
2	130	O=C(O)C1CNC1	TGGTTCGAC
2	131	NC1(C(=O)O)CC1	TTGGACCAC
2	132	NC1(CC(=O)O)CCOCC1	GCTGTGTAC
2	133	NCc1ccc(C(=O)O)cc1	CGCAGATAC
2	134	O=C(O)[C@@H]1CCCNC1	CCGATTCAC
2	135	NCC1CC(C(=O)O)CO1	ACCTCGTAC
2	136	NC[C@H](O)CC(=O)O	AGTCGTCAC
2	137	O=C(O)CCN1CCNCC1	AAGGCTGAC
2	138	Blank	ACACCTCAC
3	1	O=C1N(C(CCC(N2)=O)C2=O)C(c3c1c(OCC(O)=O)ccc3)=O	GCCAATGTT

(CRBN CIP-DEL)	2	O=C1N(C(CCC(N2)=O)C2=O)C(c3c1c(C(O)=O)ccc3)=O	CATTCGGTT
	3	O=C1N(C(CCC(N2)=O)C2=O)C(c3c1cc(C(O)=O)cc3)=O	GCTGTTGTT
	4	O=C1N(C(CCC(N2)=O)C2=O)Cc3c1cc(OCC(O)=O)cc3	TGGCTTCTT
	5	O=C1N(c2c(OC)ccc(C(O)=O)c2)CCC(N1)=O	TGTCCGATT
	6	O=C1N(c2cccc(C(O)=O)c2)CCC(N1)=O	CTCCACATT
	7	O=C1N(c2ccc(OCC(O)=O)cc2)CCC(N1)=O	CTTGGTGTT
	8	O=C1N(c2c(C)ccc(OCC(O)=O)c2)CCC(N1)=O	CTGACTGTT
3 (VHL CIP-DEL)	1	O=C([C@H](C(C)(C)C)NC(CCC(O)=O)=O)N1C[C@H](O)C[C@H]1C(NCc2ccc(c3c(C)ncs3)cc2)=O	CGATTCCTT
	2	O=C([C@H](C(C)(C)C)NC(CCC(O)=O)=O)N1C[C@H](O)C[C@H]1C(N[C@@H](C)c2ccc(c3c(C)ncs3)cc2)=O	GTGGTTGTT
	3	O=C([C@H](C(C)(C)C)NC(C1(F)CC1)=O)N2C[C@H](O)C[C@H]2C(NCc3ccc(c4c(C)ncs4)cc3OCC(O)=O)=O	GGAACGATT
3 (FKBP CIP-DEL)	1	O=C(C(c1cccc(OCC(O)=O)c1)=O)N2CCCC[C@H]2C(OCCCc3ccc(OC)c(OC)c3)=O	CTCCACATT
	2	O=C(C(c1cccc(OCC(O)=O)c1)=O)N2CCCC[C@H]2C(O[C@@H](c3ccccc3)CCc4ccc(OC)c(OC)c4)=O	CTTGGTGTT
	3	O=C(C(C(CC)(C)C)=O)N1CCCC[C@H]1C(OCCCc2ccc(OCC(O)=O)cc2)=O	CTGACTGTT
	4	O=C(C(C(CC)(C)C)=O)N1CCCC[C@H]1C(O[C@@H](c2ccccc2)CCc3ccc(OCC(O)=O)cc3)=O	TACGTGGTT
	5	OC([C@H](NC([C@H]1N(C(C(C(CC)(C)C)=O)=O)CCCC1)=O)CCc2ccc(OC)cc2)=O	CGCTTAGTT
	6	O=C(C(C(CC)(C)C)=O)N1CCCC[C@H]1C(O[C@@H](c2cc(OCC(O)=O)ccc2)CCc3ccc(OC)c(OC)c3)=O	GTCGAGATT

Appendix III. NMR Spectra

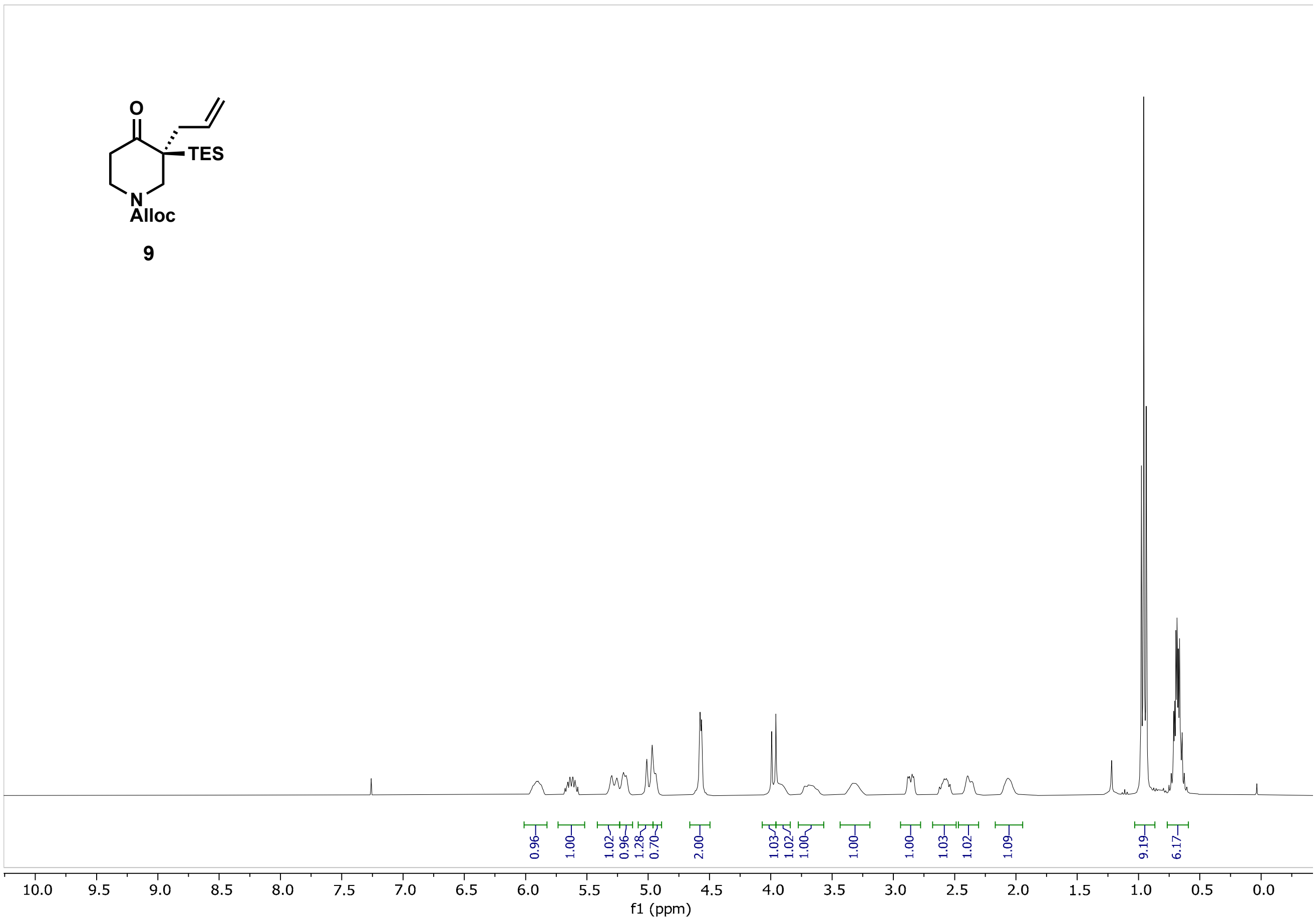

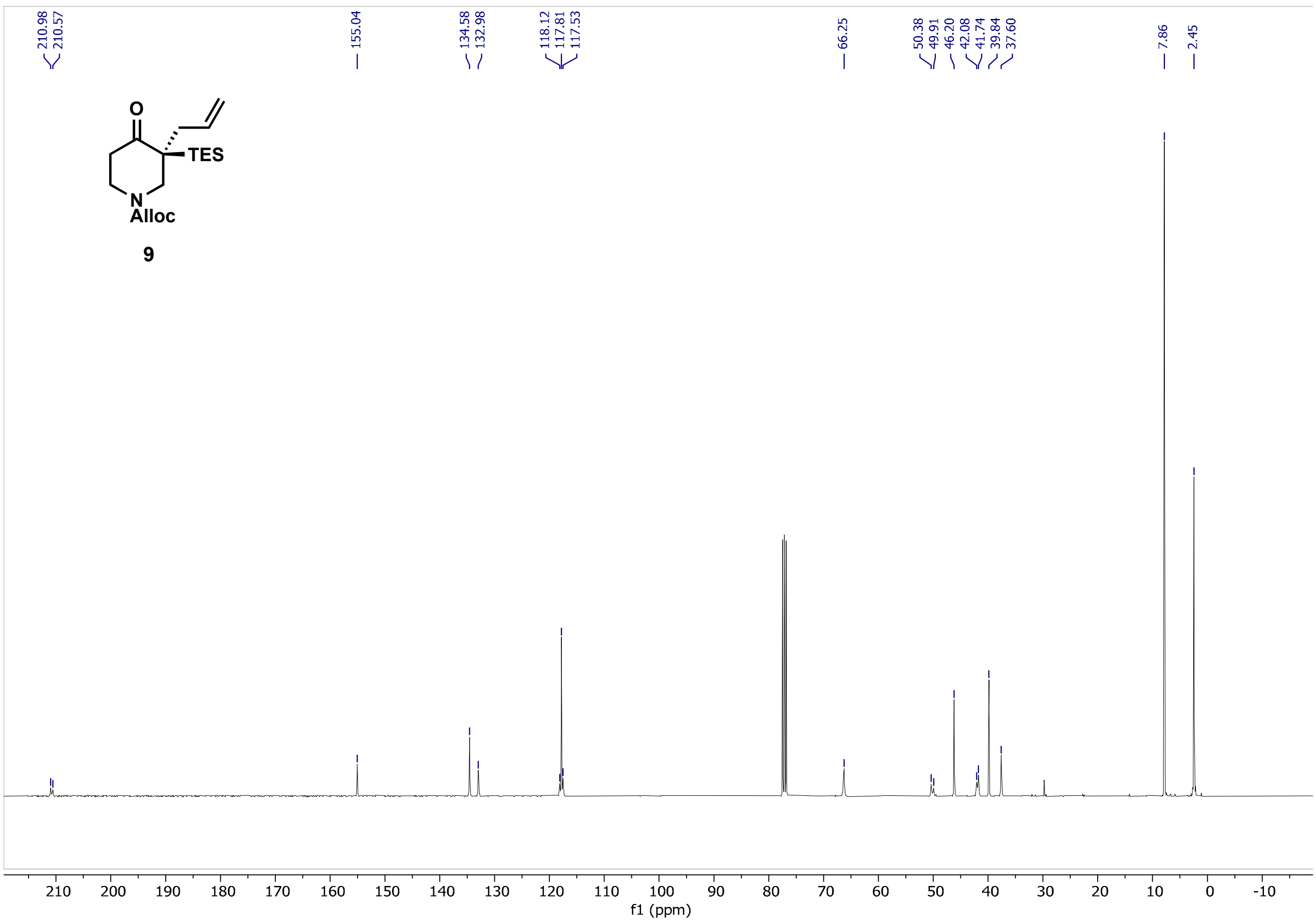

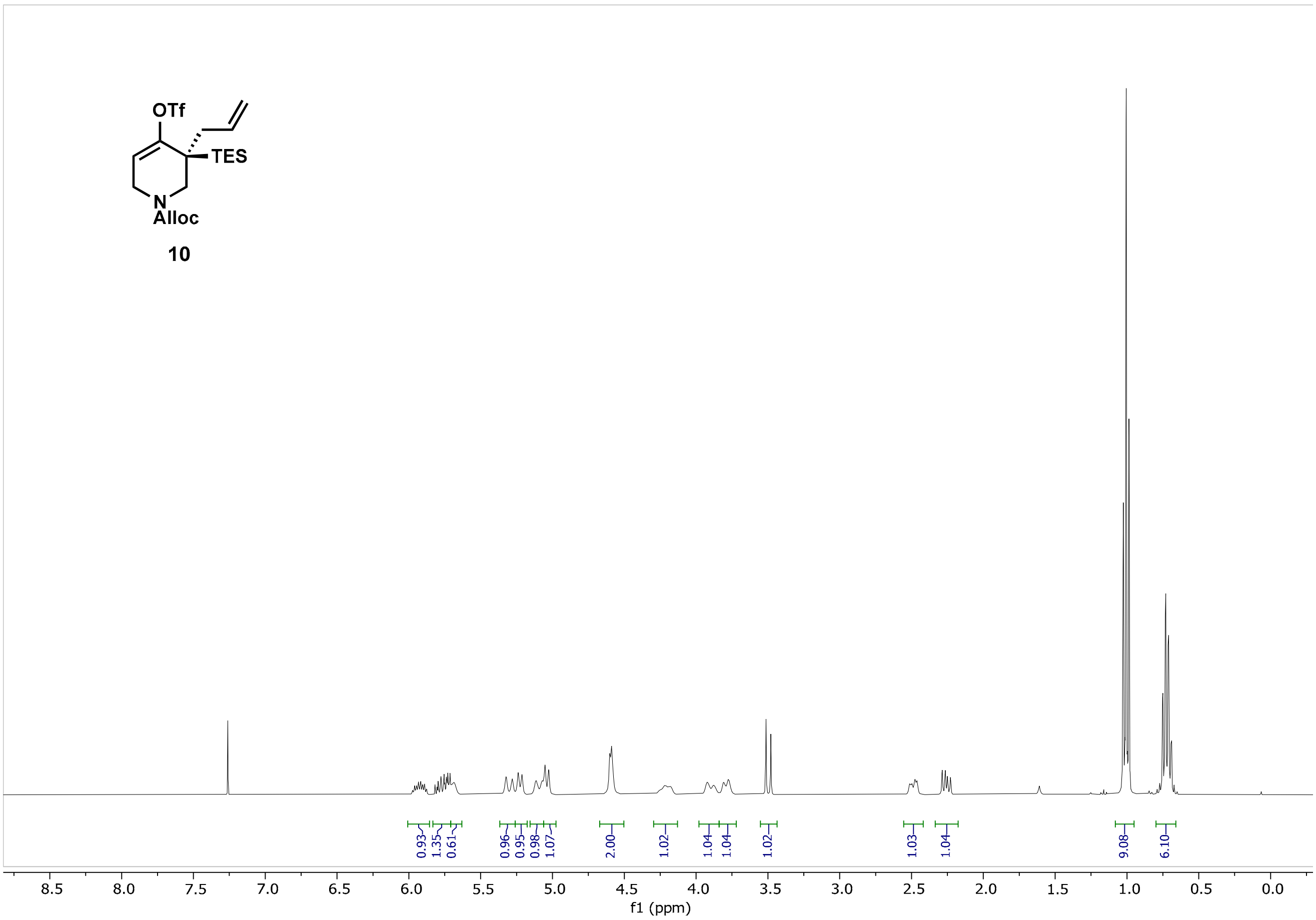
OTf
TES
N
Alloc
10
0.93
1.35
0.61
0.96
0.95
0.98
1.07
2.00
1.02
1.04
1.04
1.02
1.03
1.04
9.08
6.10
8.5
8.0
7.5
7.0
6.5
6.0
5.5
5.0
4.5
4.0
3.5
3.0
2.5
2.0
1.5
1.0
0.5
0.0
f1 (ppm)

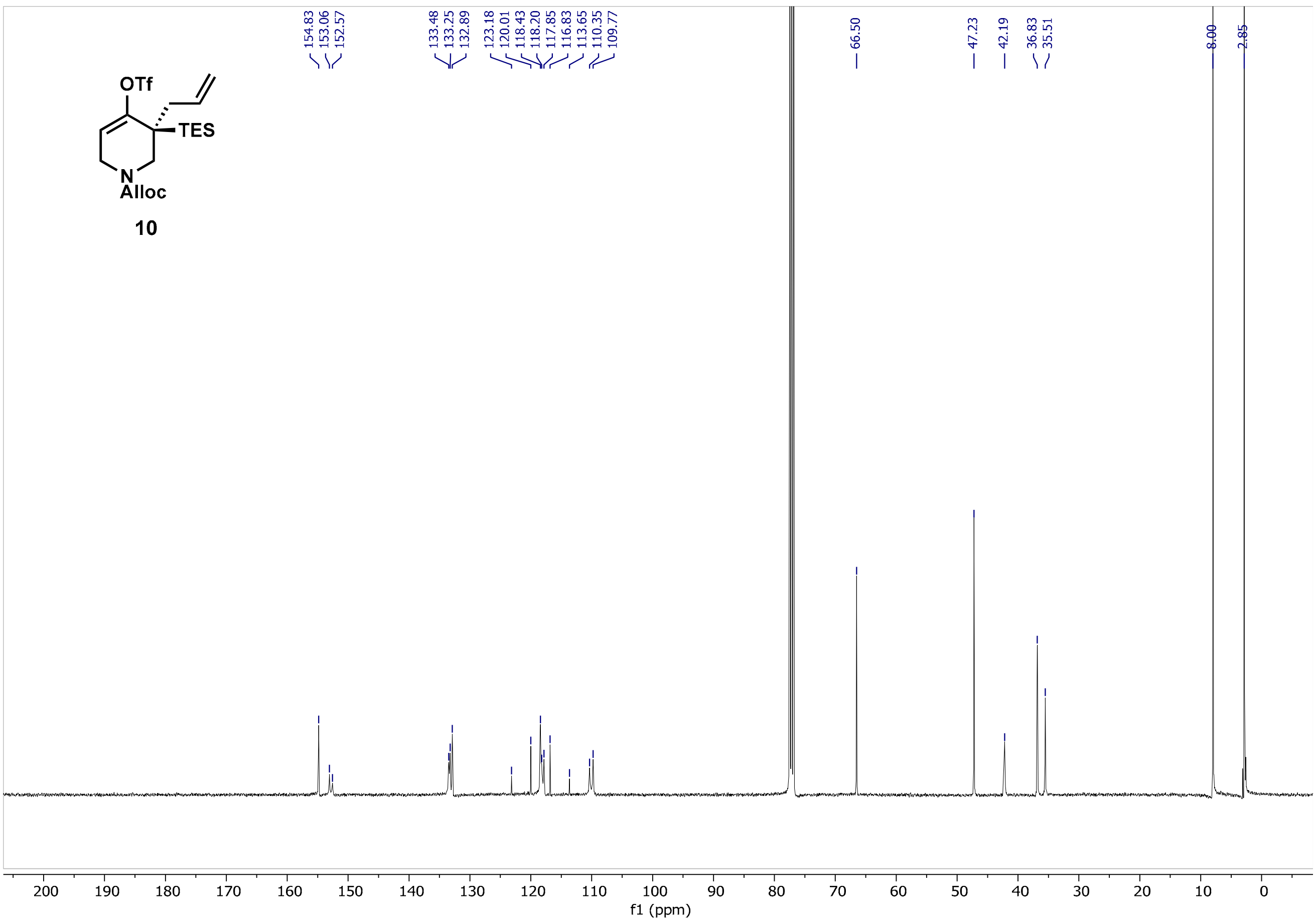
154.83
153.06
152.57
133.48
133.25
132.89
123.18
120.01
118.43
118.20
117.85
116.83
113.65
110.35
109.77
66.50
47.23
42.19
36.83
35.51
8.00
2.85
OTf
TES
N
Alloc
10
200
190
180
170
160
150
140
130
120
110
100
90
80
70
60
50
40
30
20
10
0
f1 (ppm)

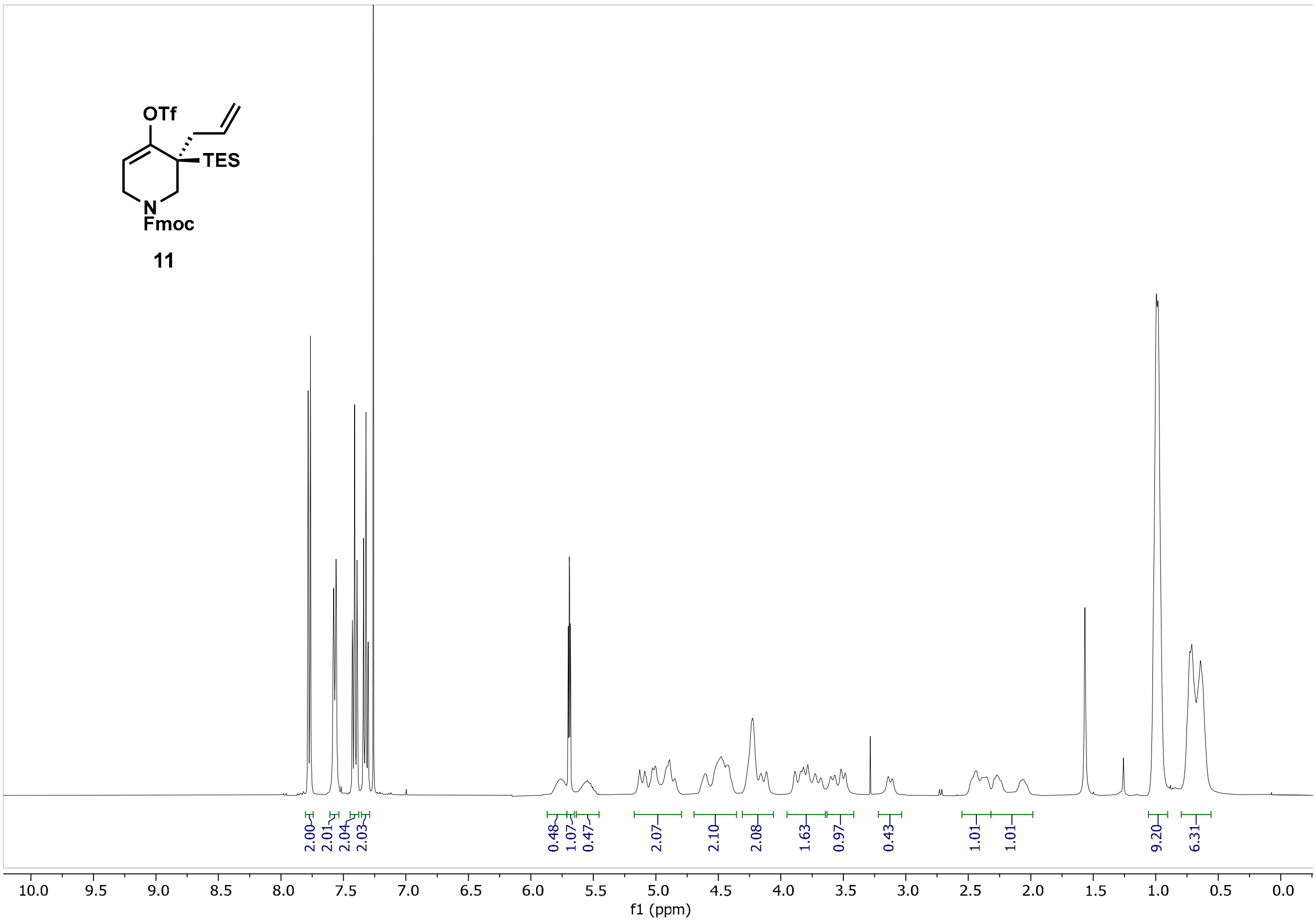
OTf
TES
N
Fmoc
11
2.00
2.01
2.04
2.03
0.48
1.07
0.47
2.07
2.10
2.08
1.63
0.97
0.43
1.01
1.01
9.20
6.31
f1 (ppm)

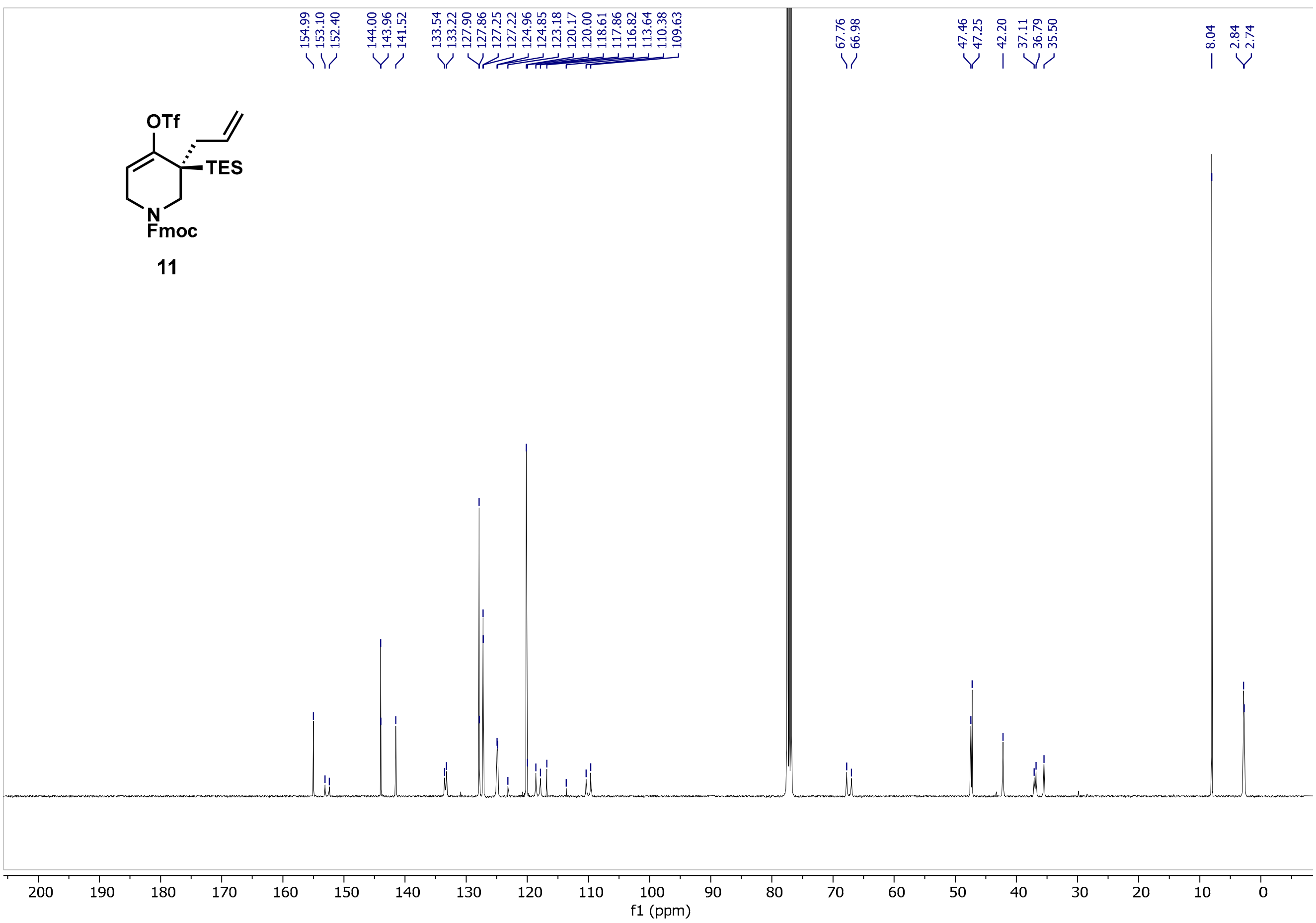
154.99
153.10
152.40
144.00
143.96
141.52
133.54
133.22
127.90
127.86
127.25
127.22
124.96
124.85
123.18
120.17
120.00
118.61
117.86
116.82
113.64
110.38
109.63
67.76
66.98
47.46
47.25
42.20
37.11
36.79
35.50
8.04
2.84
2.74
OTf
TES
N
Fmoc
11
f1 (ppm)

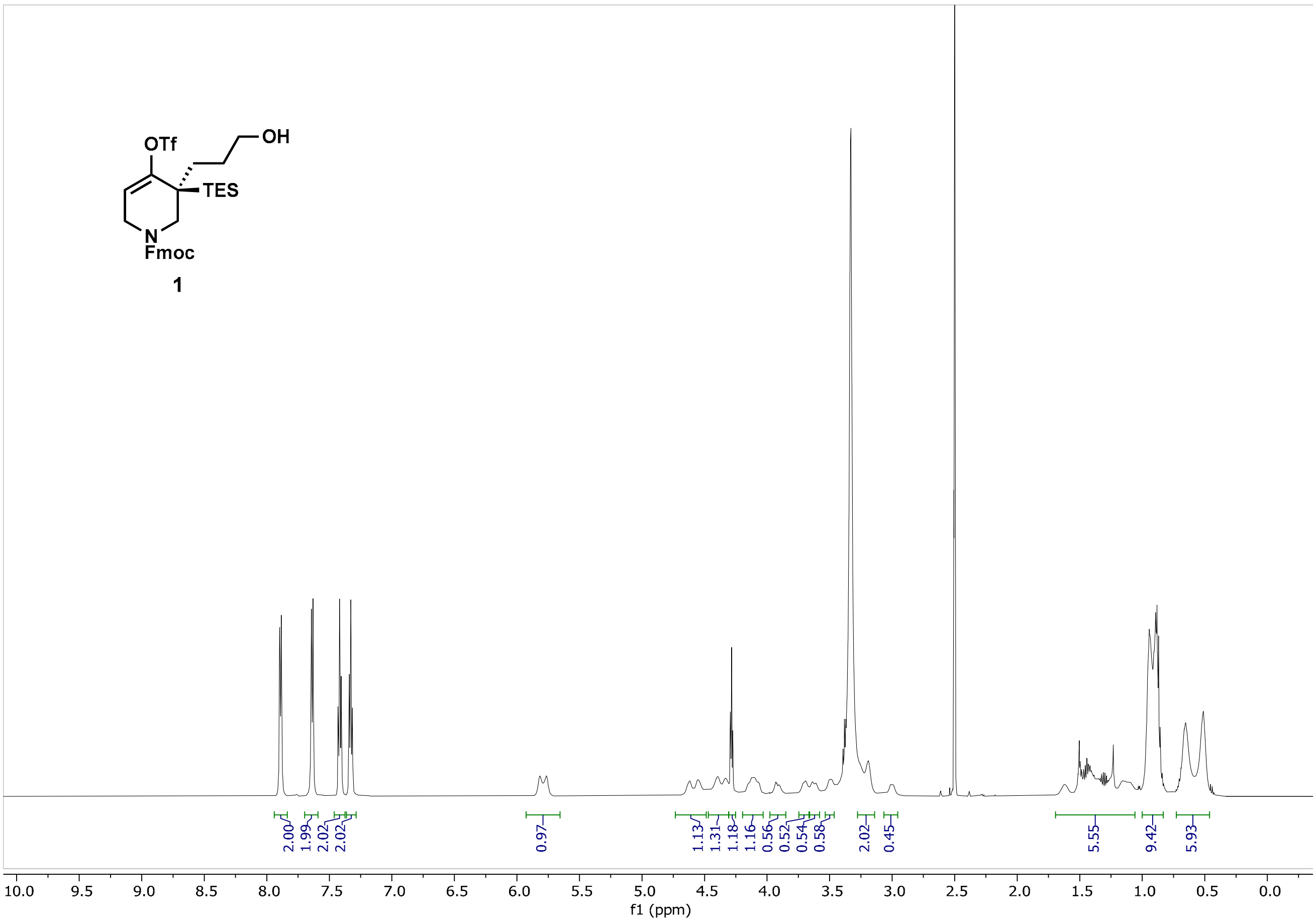
OTf
OH
TES
N
Fmoc
1
2.00
1.99
2.02
2.02
0.97
1.13
1.31
1.18
1.16
0.56
0.52
0.54
0.58
2.02
0.45
5.55
9.42
5.93
10.0 9.5 9.0 8.5 8.0 7.5 7.0 6.5 6.0 5.5 5.0 4.5 4.0 3.5 3.0 2.5 2.0 1.5 1.0 0.5 0.0
f1 (ppm)

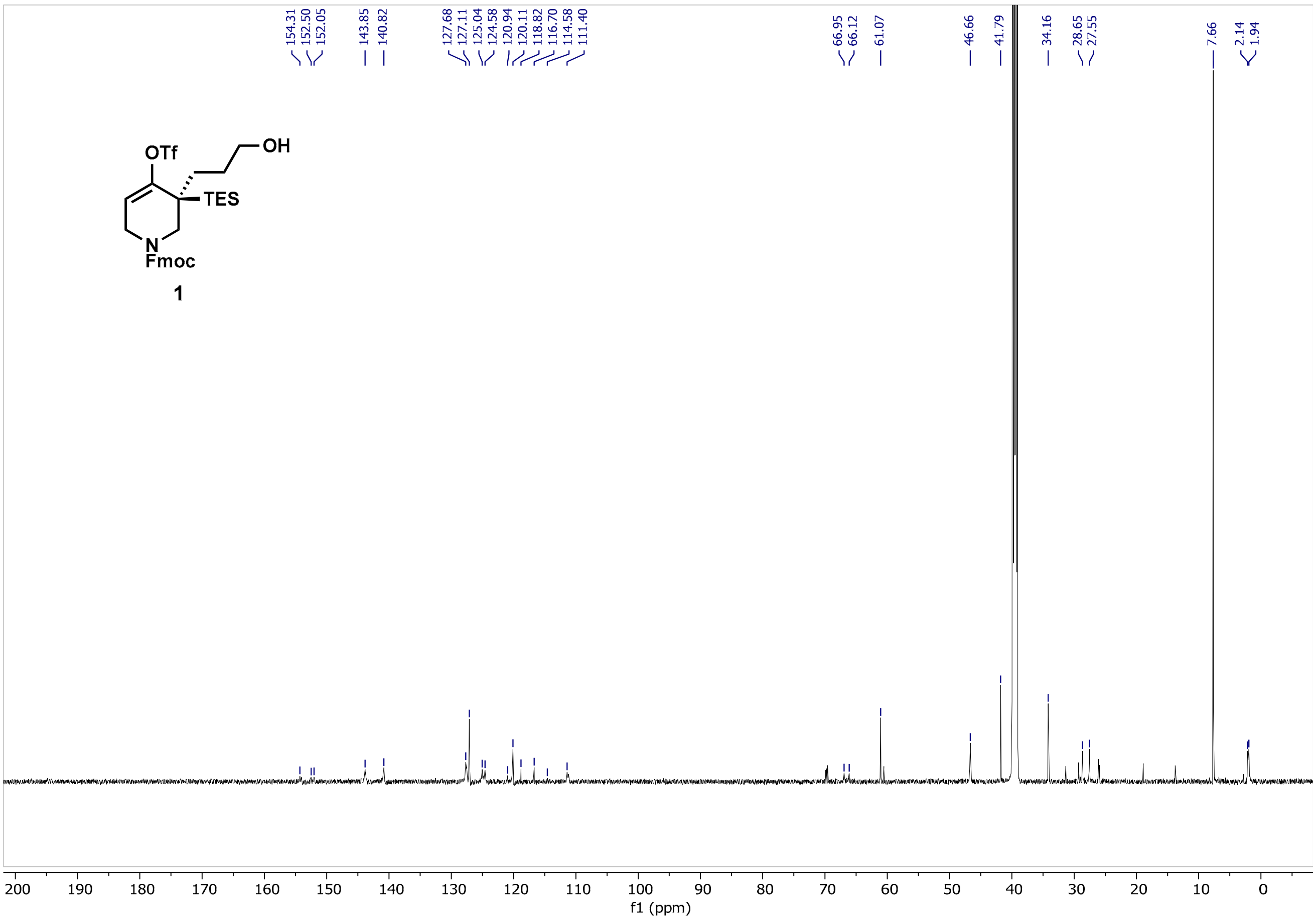
154.31
152.50
152.05
143.85
140.82
127.68
127.11
125.04
124.58
120.94
120.11
118.82
116.70
114.58
111.40
66.95
66.12
61.07
46.66
41.79
34.16
28.65
27.55
7.66
2.14
1.94
OTf
OH
TES
N
Fmoc
1
200 190 180 170 160 150 140 130 120 110 100 90 80 70 60 50 40 30 20 10 0
f1 (ppm)

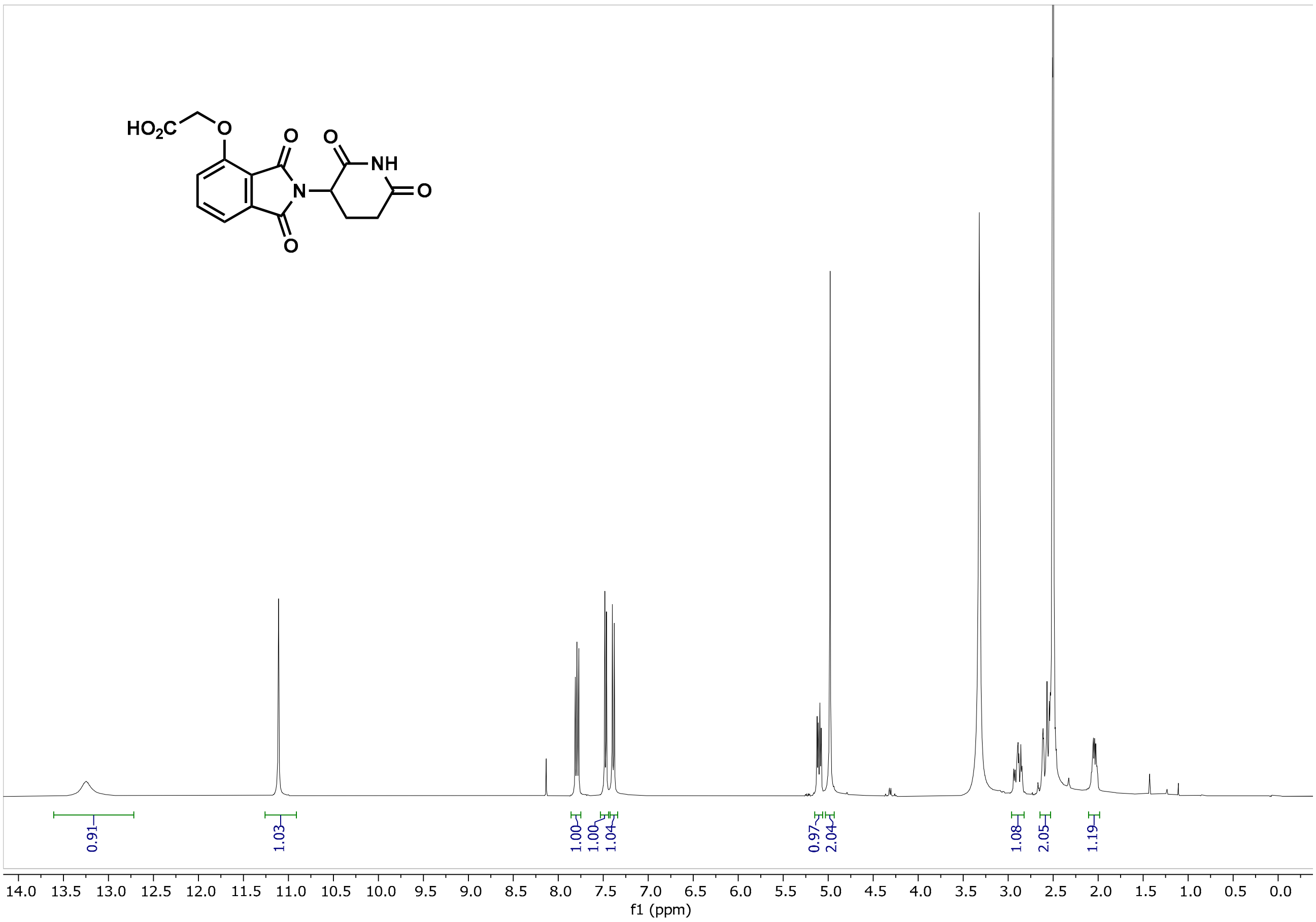
HO2C
O
O
O
NH
N
O
O
0.91
1.03
1.00
1.00
1.04
0.97
2.04
1.08
2.05
1.19
14.0 13.5 13.0 12.5 12.0 11.5 11.0 10.5 10.0 9.5 9.0 8.5 8.0 7.5 7.0 6.5 6.0 5.5 5.0 4.5 4.0 3.5 3.0 2.5 2.0 1.5 1.0 0.5 0.0
f1 (ppm)

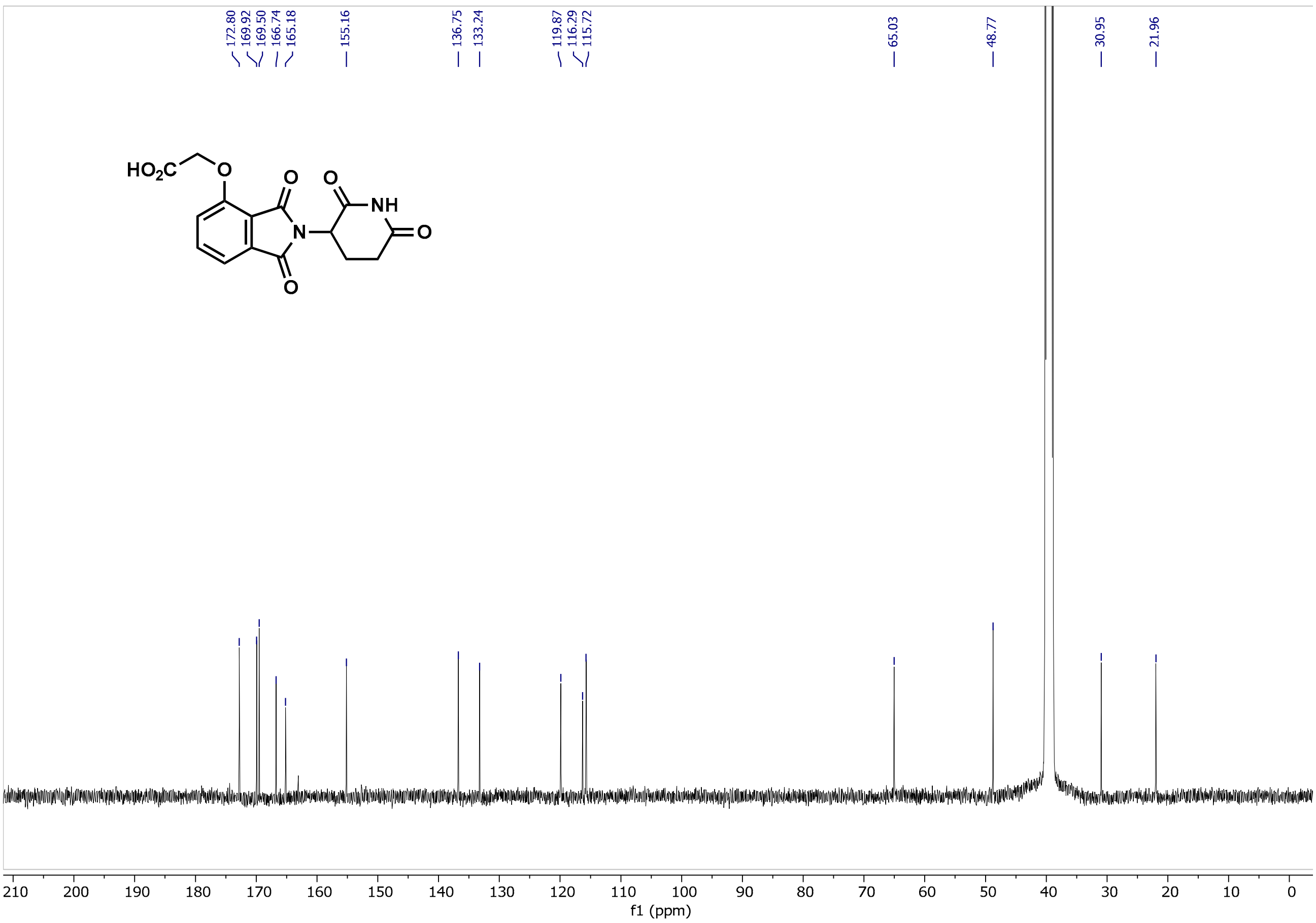
172.80
169.92
169.50
166.74
165.18
155.16
136.75
133.24
119.87
116.29
115.72
65.03
48.77
30.95
21.96
HO2C
O
O
O
NH
N
O
O
210 200 190 180 170 160 150 140 130 120 110 100 90 80 70 60 50 40 30 20 10 0
f1 (ppm)

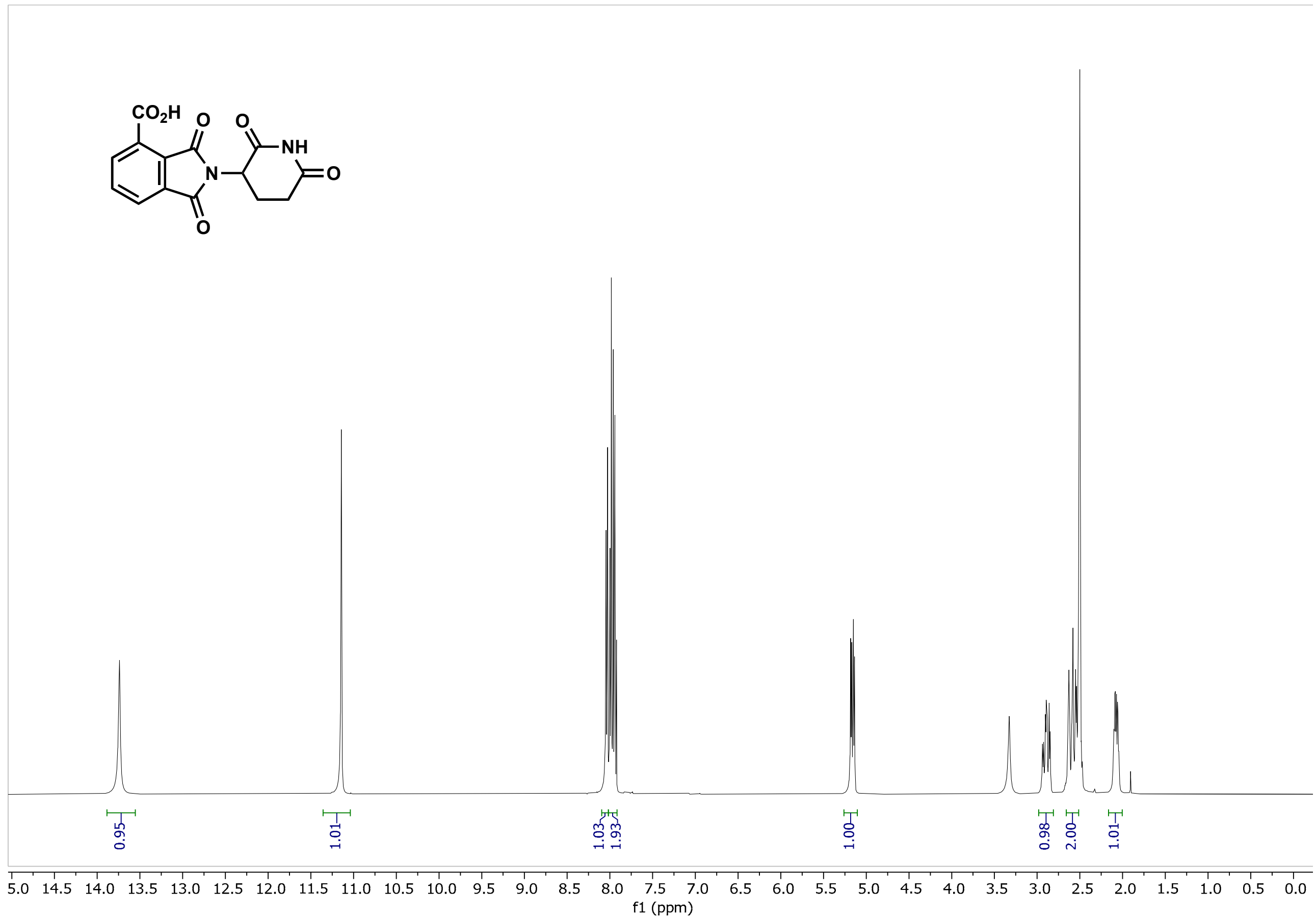

CO_2H
O
O
NH
N
O
O
0.95
1.01
1.03
1.93
1.00
0.98
2.00
1.01
5.0 14.5 14.0 13.5 13.0 12.5 12.0 11.5 11.0 10.5 10.0 9.5 9.0 8.5 8.0 7.5 7.0 6.5 6.0 5.5 5.0 4.5 4.0 3.5 3.0 2.5 2.0 1.5 1.0 0.5 0.0
f1 (ppm)

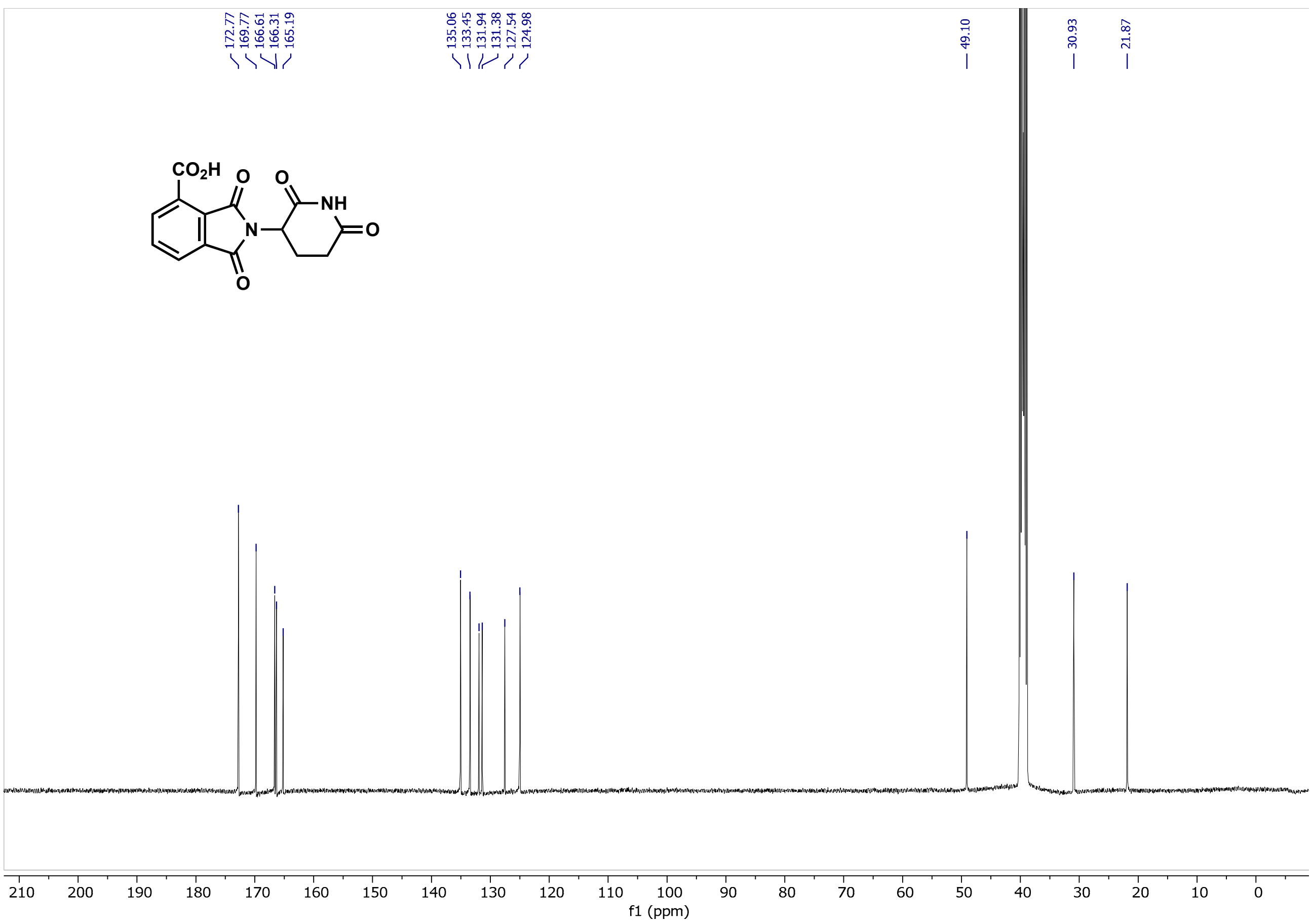

172.77
169.77
166.61
166.31
165.19
135.06
133.45
131.94
131.38
127.54
124.98
49.10
30.93
21.87
CO_2H
O
O
NH
N
O
O
210 200 190 180 170 160 150 140 130 120 110 100 90 80 70 60 50 40 30 20 10 0
f1 (ppm)

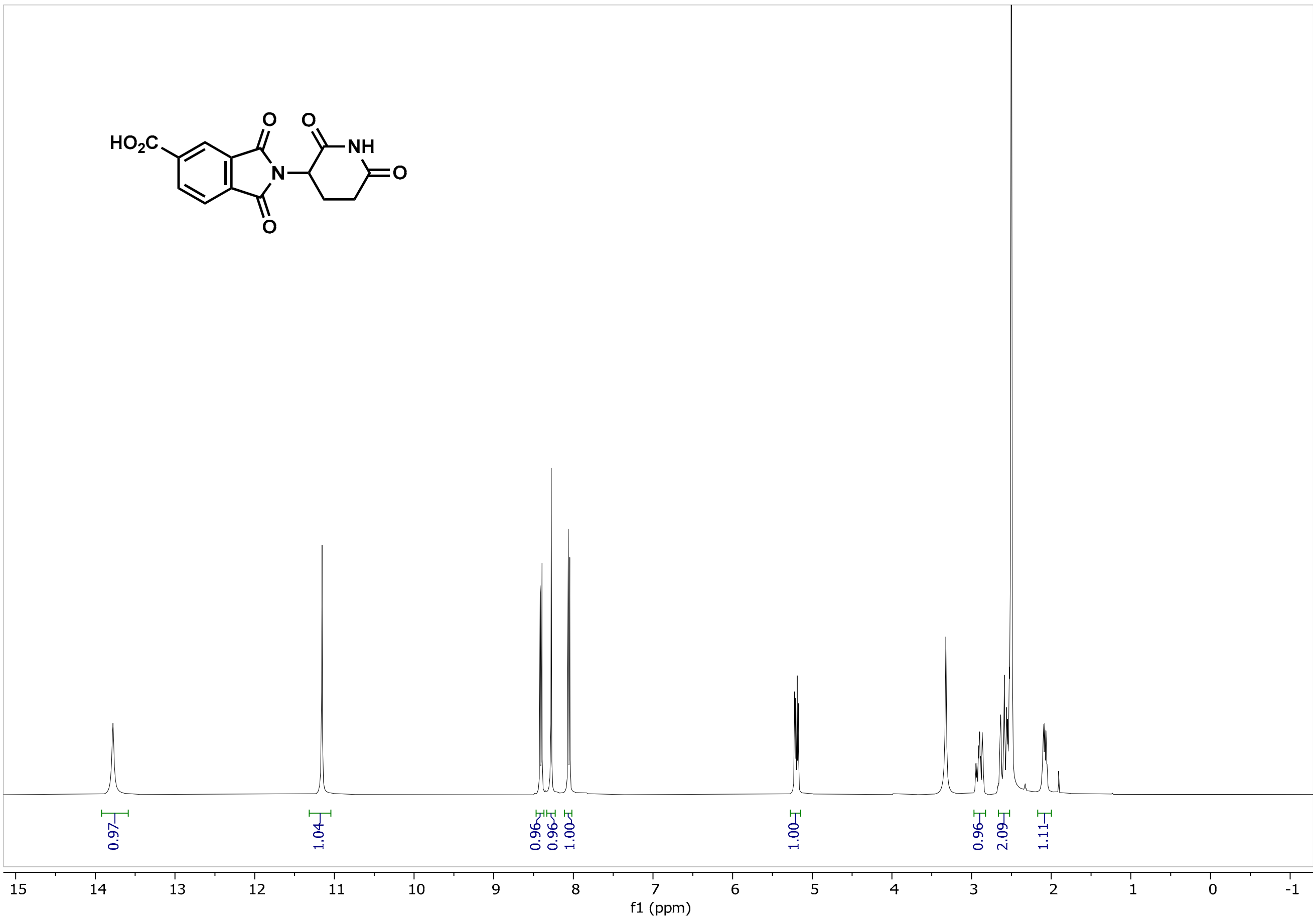
HO2C
O
O
N
O
NH
O
0.97
1.04
0.96
0.96
1.00
1.00
0.96
2.09
1.11
15
14
13
12
11
10
9
8
7
6
5
4
3
2
1
0
-1
f1 (ppm)

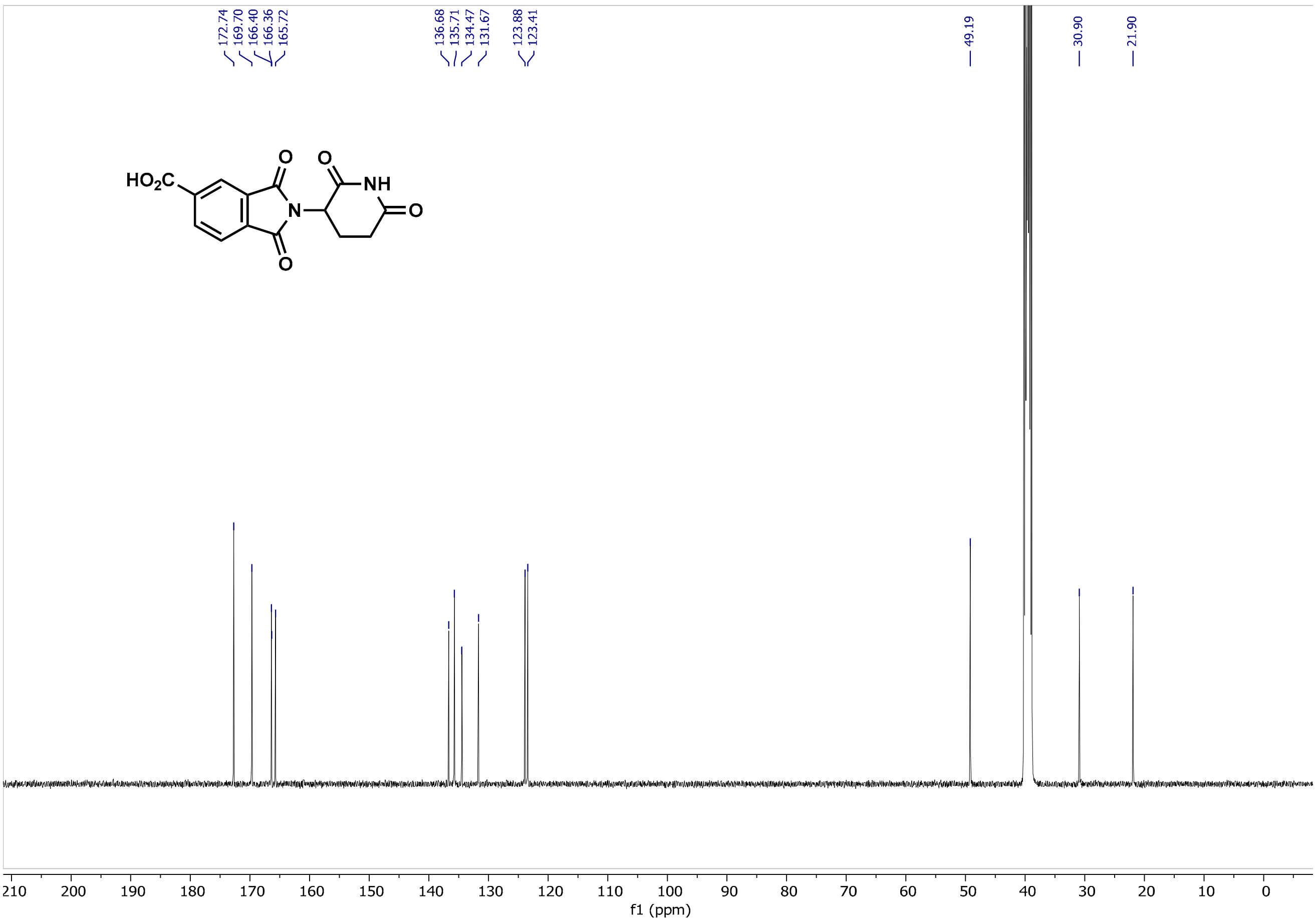
172.74
169.70
166.40
166.36
165.72
136.68
135.71
134.47
131.67
123.88
123.41
49.19
30.90
21.90
HO2C
O
O
N
O
NH
O
210
200
190
180
170
160
150
140
130
120
110
100
90
80
70
60
50
40
30
20
10
0
f1 (ppm)

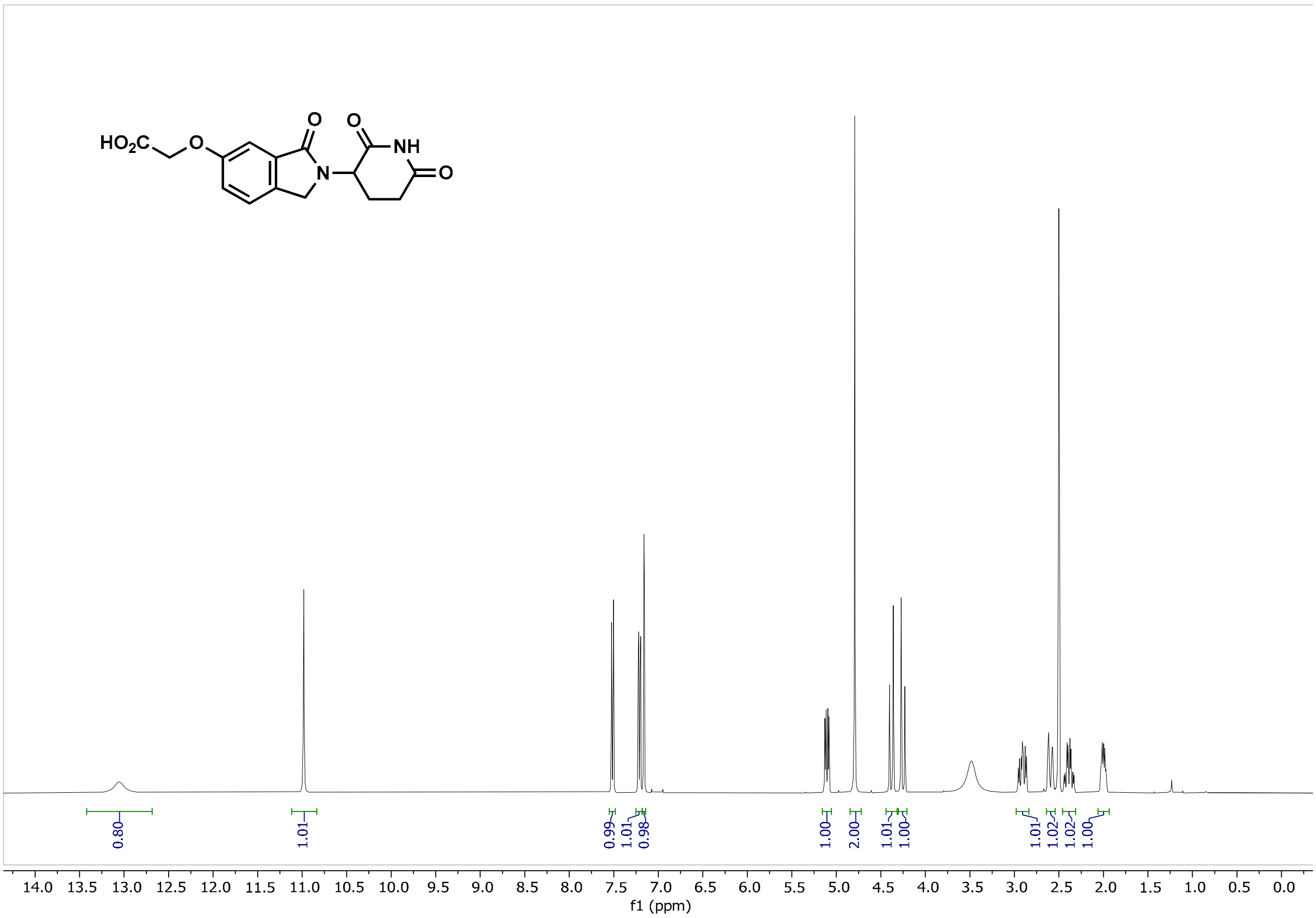
HO2C
O
O
N
O
NH
O
0.80
1.01
0.99
1.01
0.98
1.00
2.00
1.01
1.00
1.01
1.02
1.02
1.00
14.0 13.5 13.0 12.5 12.0 11.5 11.0 10.5 10.0 9.5 9.0 8.5 8.0 7.5 7.0 6.5 6.0 5.5 5.0 4.5 4.0 3.5 3.0 2.5 2.0 1.5 1.0 0.5 0.0
f1 (ppm)

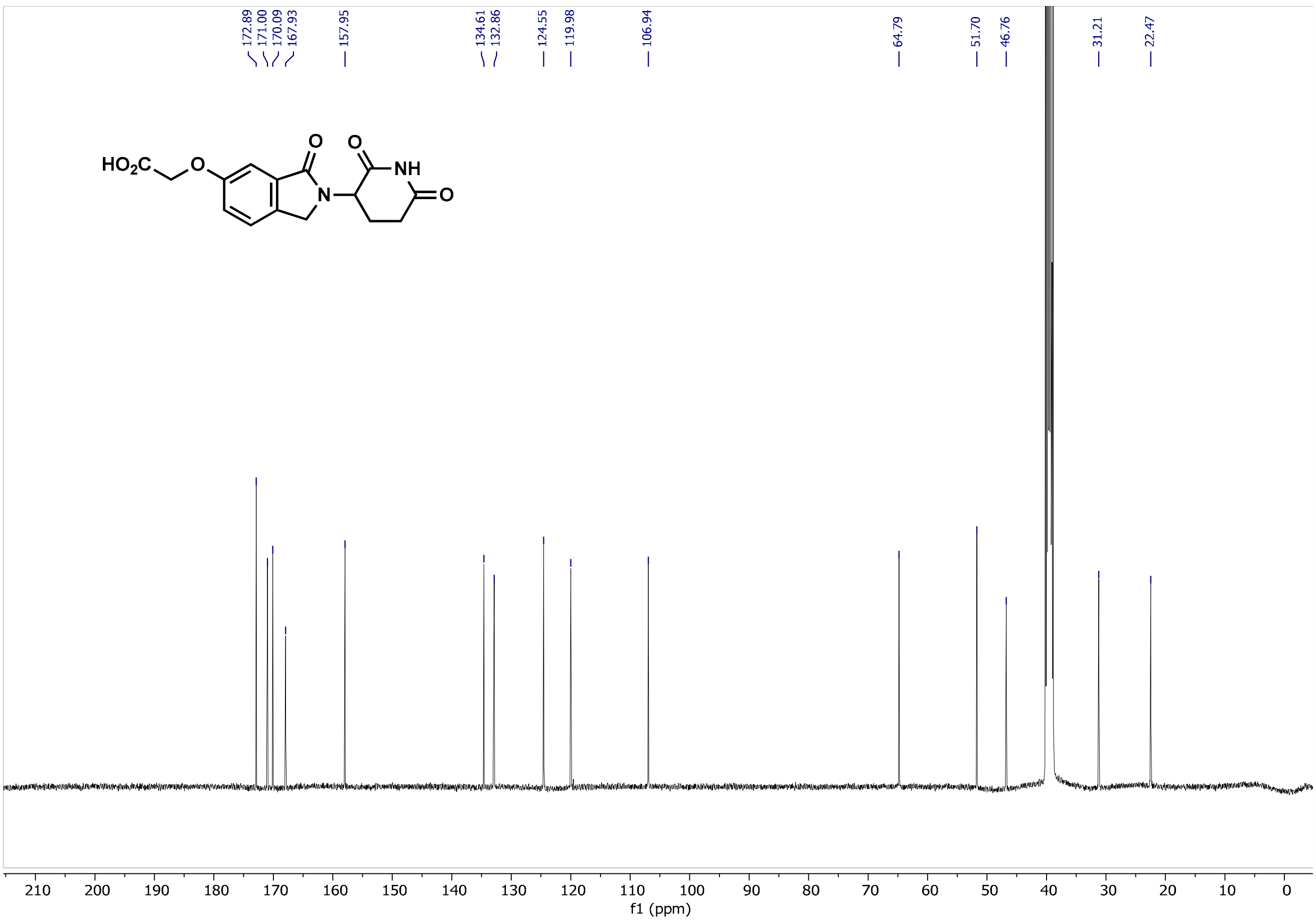
172.89
171.00
170.09
167.93
157.95
134.61
132.86
124.55
119.98
106.94
64.79
51.70
46.76
31.21
22.47
HO2C
O
O
N
O
NH
O
210 200 190 180 170 160 150 140 130 120 110 100 90 80 70 60 50 40 30 20 10 0
f1 (ppm)

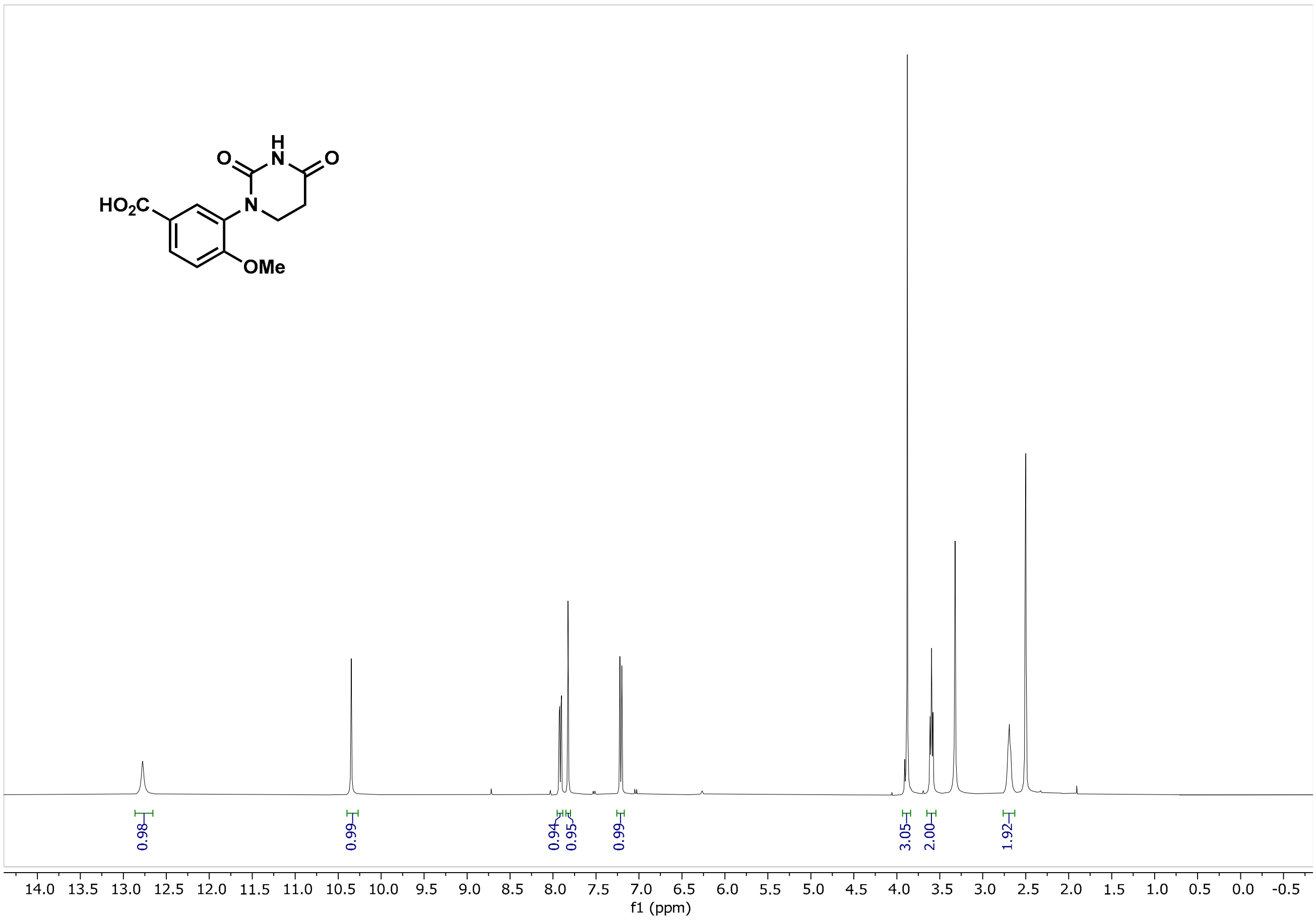
HO2C
OMe
0.98
0.99
0.94
0.95
0.99
3.05
2.00
1.92
14.0 13.5 13.0 12.5 12.0 11.5 11.0 10.5 10.0 9.5 9.0 8.5 8.0 7.5 7.0 6.5 6.0 5.5 5.0 4.5 4.0 3.5 3.0 2.5 2.0 1.5 1.0 0.5 0.0 -0.5
f1 (ppm)

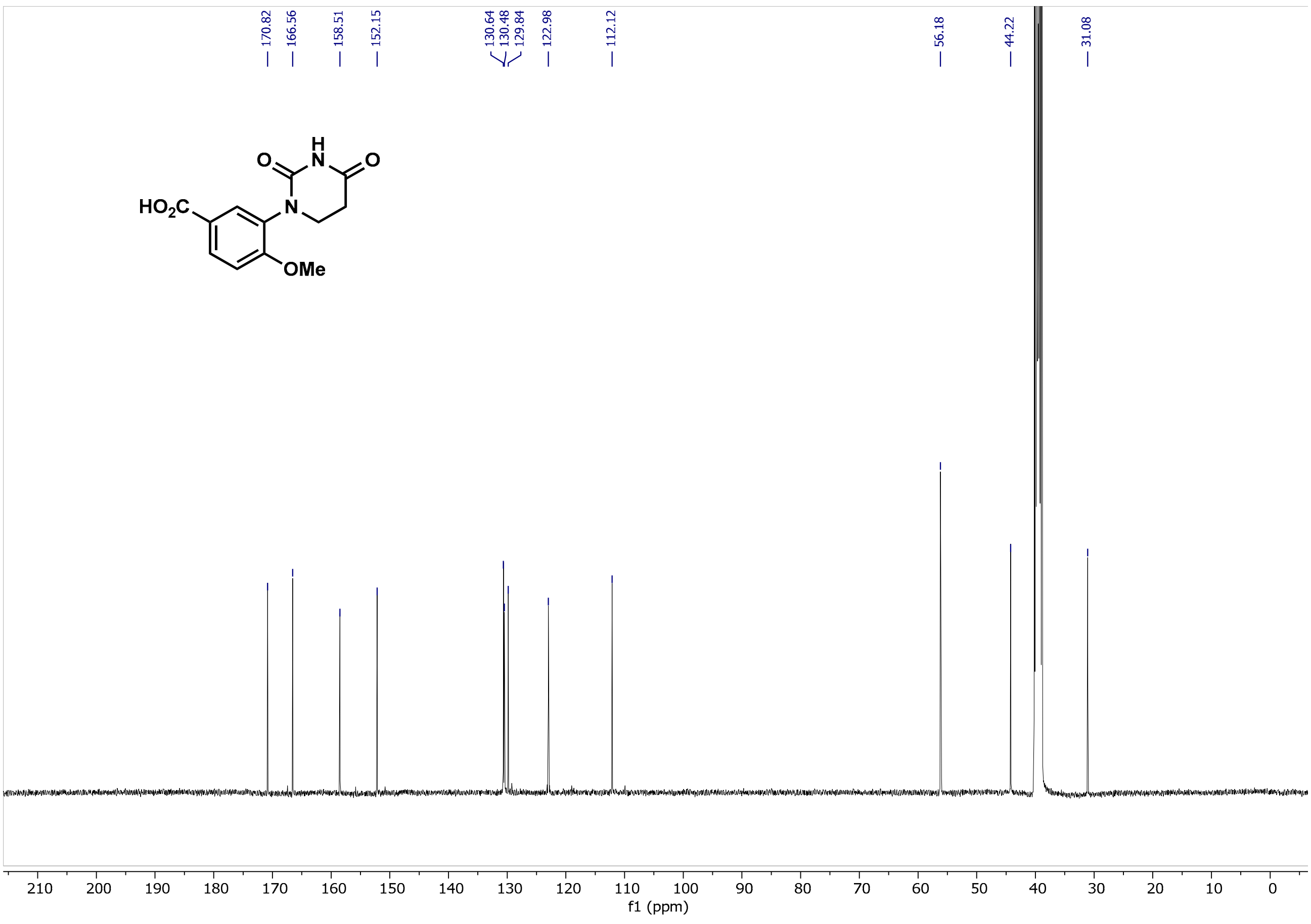
170.82
166.56
158.51
152.15
130.64
130.48
129.84
122.98
112.12
56.18
44.22
31.08
HO2C
OMe
210 200 190 180 170 160 150 140 130 120 110 100 90 80 70 60 50 40 30 20 10 0
f1 (ppm)

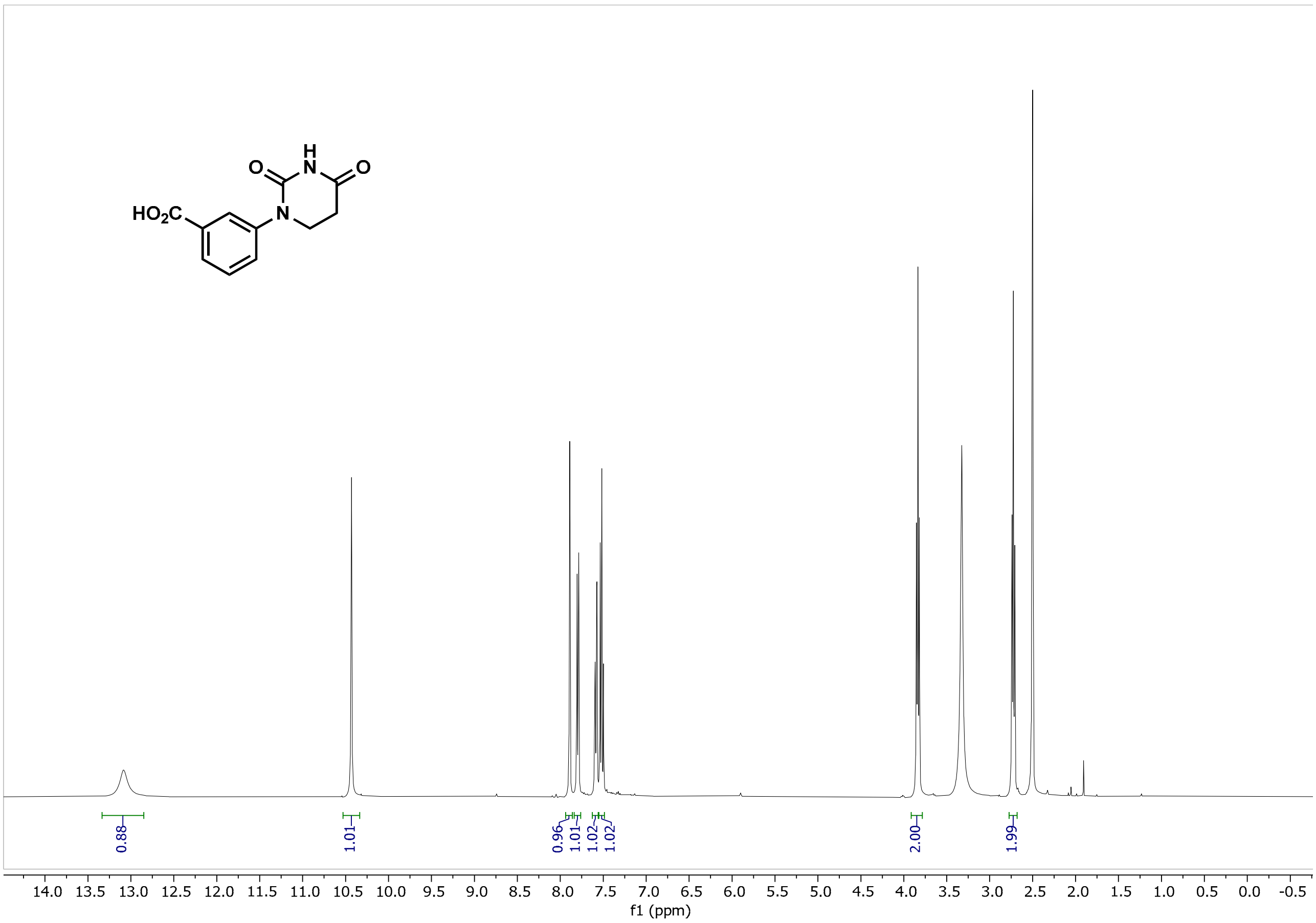

HO2C
N
H
O
O
0.88
1.01
0.96
1.01
1.02
1.02
2.00
1.99
14.0 13.5 13.0 12.5 12.0 11.5 11.0 10.5 10.0 9.5 9.0 8.5 8.0 7.5 7.0 6.5 6.0 5.5 5.0 4.5 4.0 3.5 3.0 2.5 2.0 1.5 1.0 0.5 0.0 -0.5
f1 (ppm)

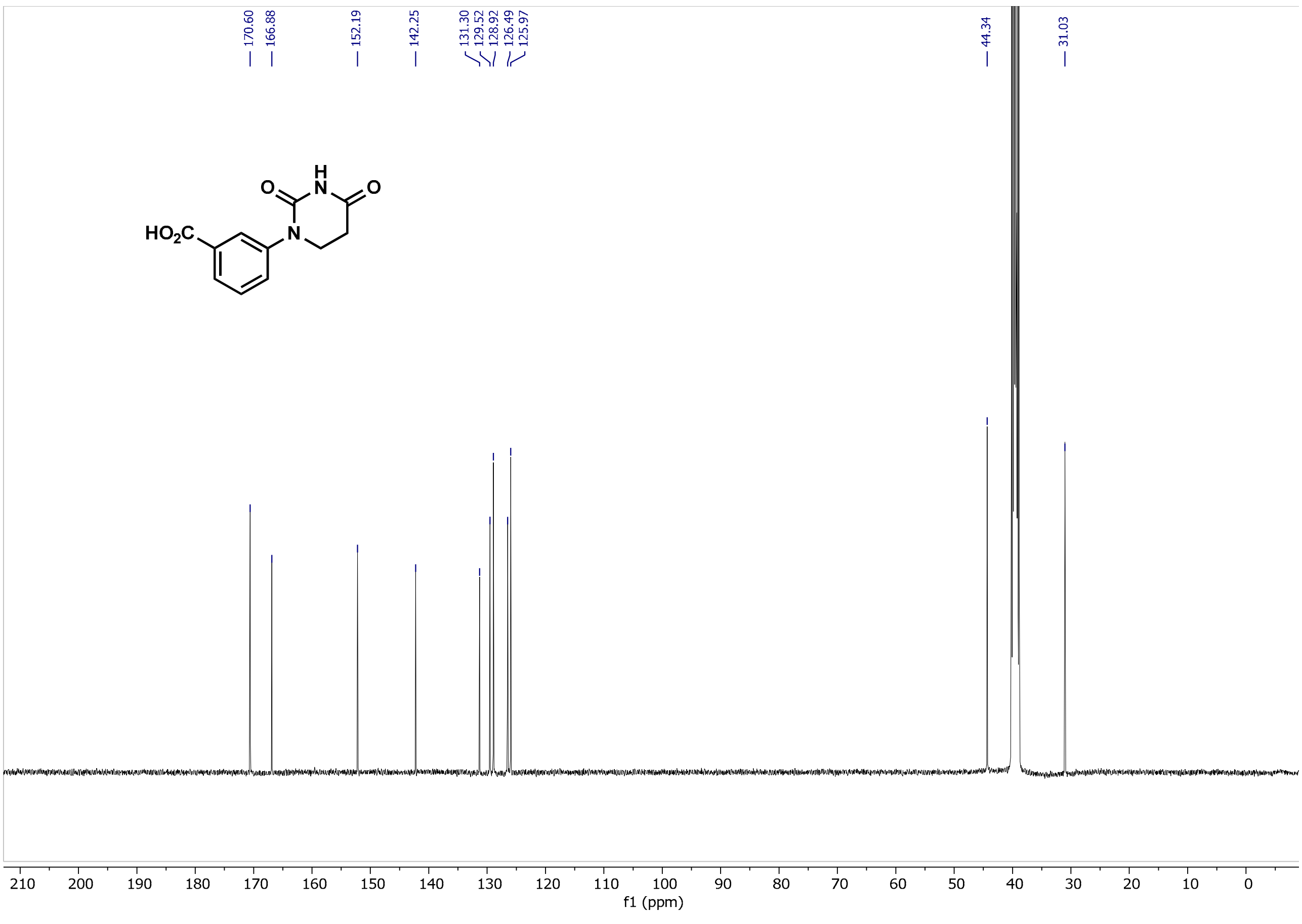

170.60
166.88
152.19
142.25
131.30
129.52
128.92
126.49
125.97
44.34
31.03
HO2C
N
H
O
O
210 200 190 180 170 160 150 140 130 120 110 100 90 80 70 60 50 40 30 20 10 0
f1 (ppm)

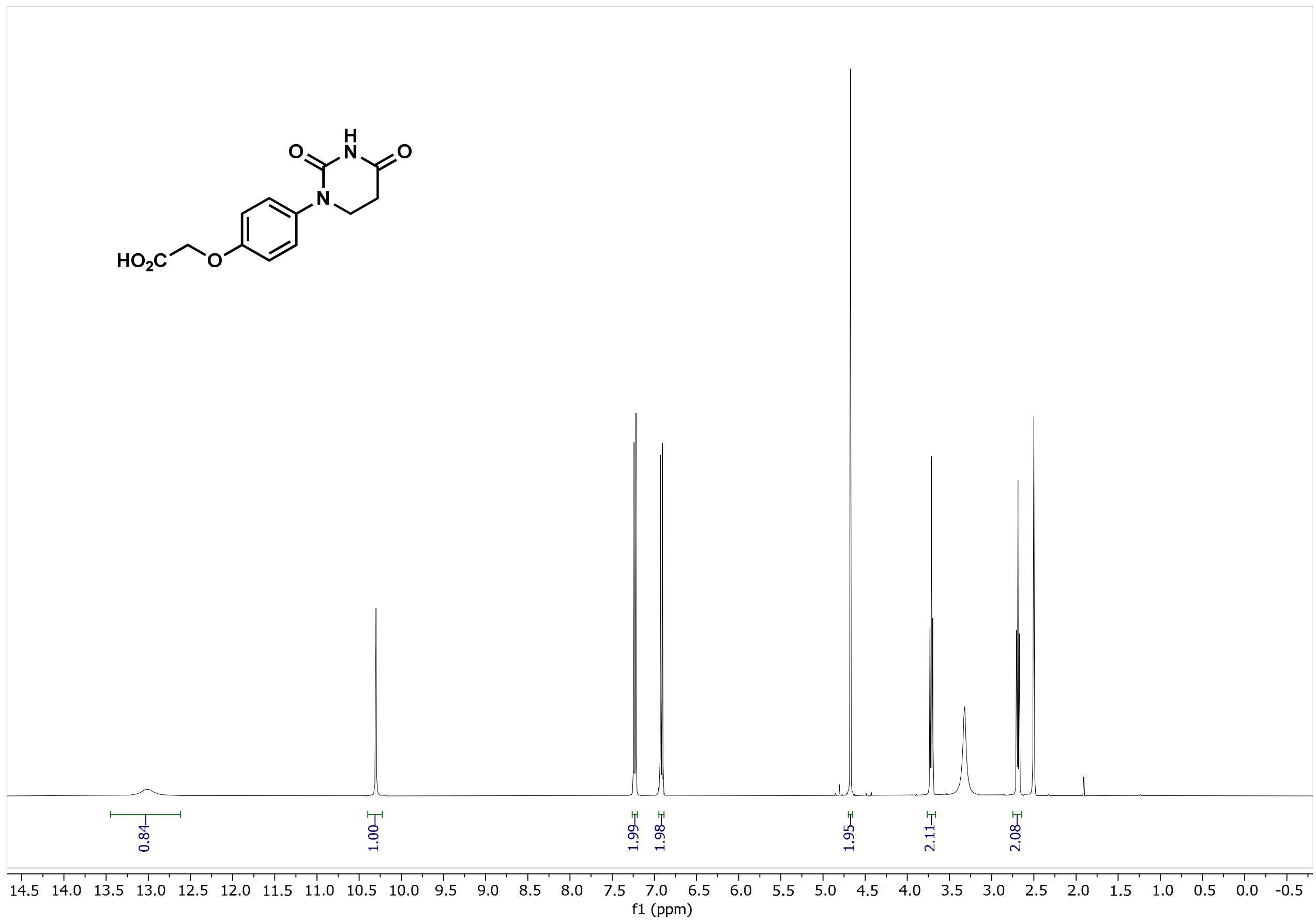
HO2C
O
N
H
0.84
1.00
1.99
1.98
1.95
2.11
2.08
14.5 14.0 13.5 13.0 12.5 12.0 11.5 11.0 10.5 10.0 9.5 9.0 8.5 8.0 7.5 7.0 6.5 6.0 5.5 5.0 4.5 4.0 3.5 3.0 2.5 2.0 1.5 1.0 0.5 0.0 -0.5
f1 (ppm)

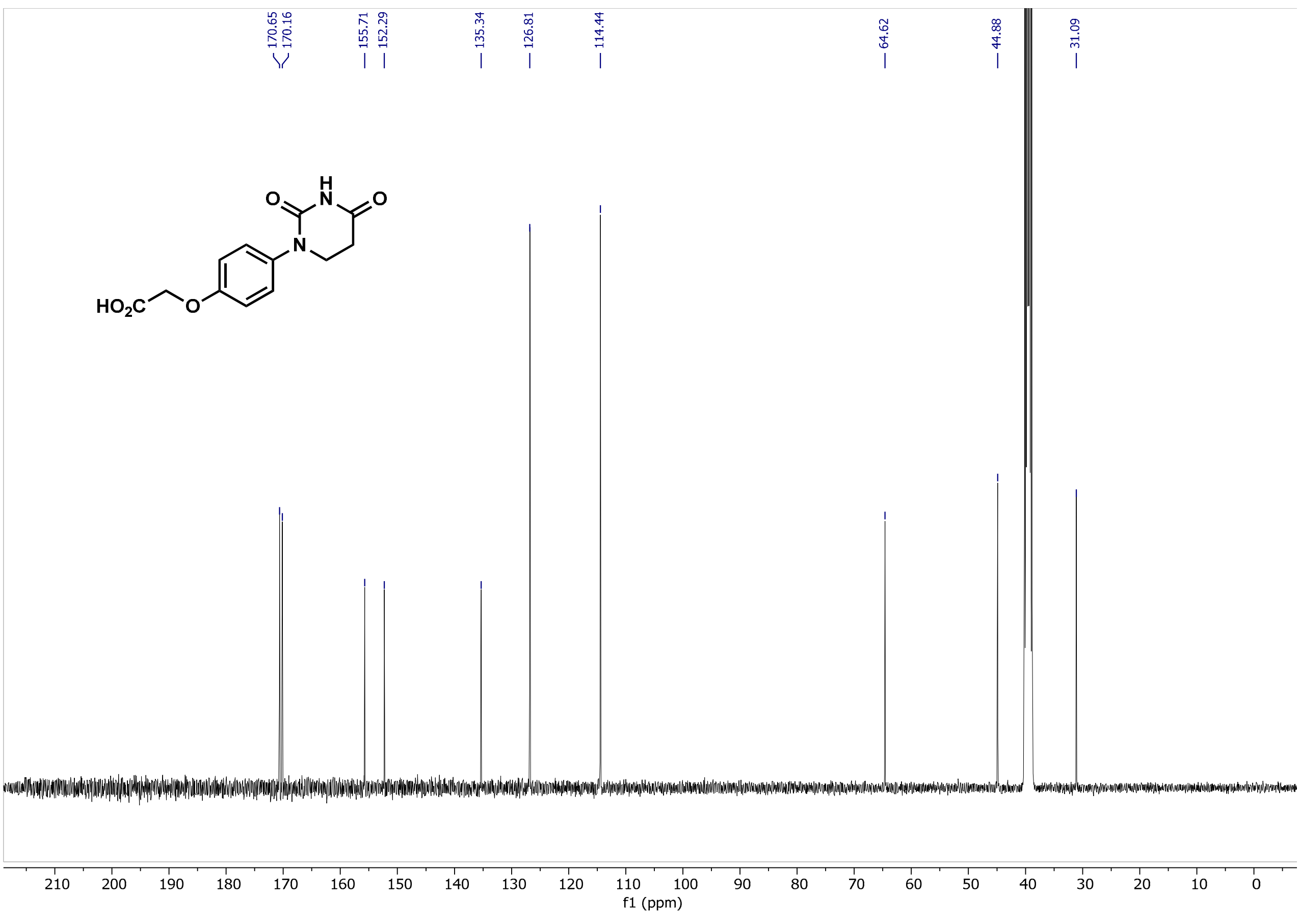
170.65
170.16
155.71
152.29
135.34
126.81
114.44
64.62
44.88
31.09
HO2C
O
N
H
210 200 190 180 170 160 150 140 130 120 110 100 90 80 70 60 50 40 30 20 10 0
f1 (ppm)

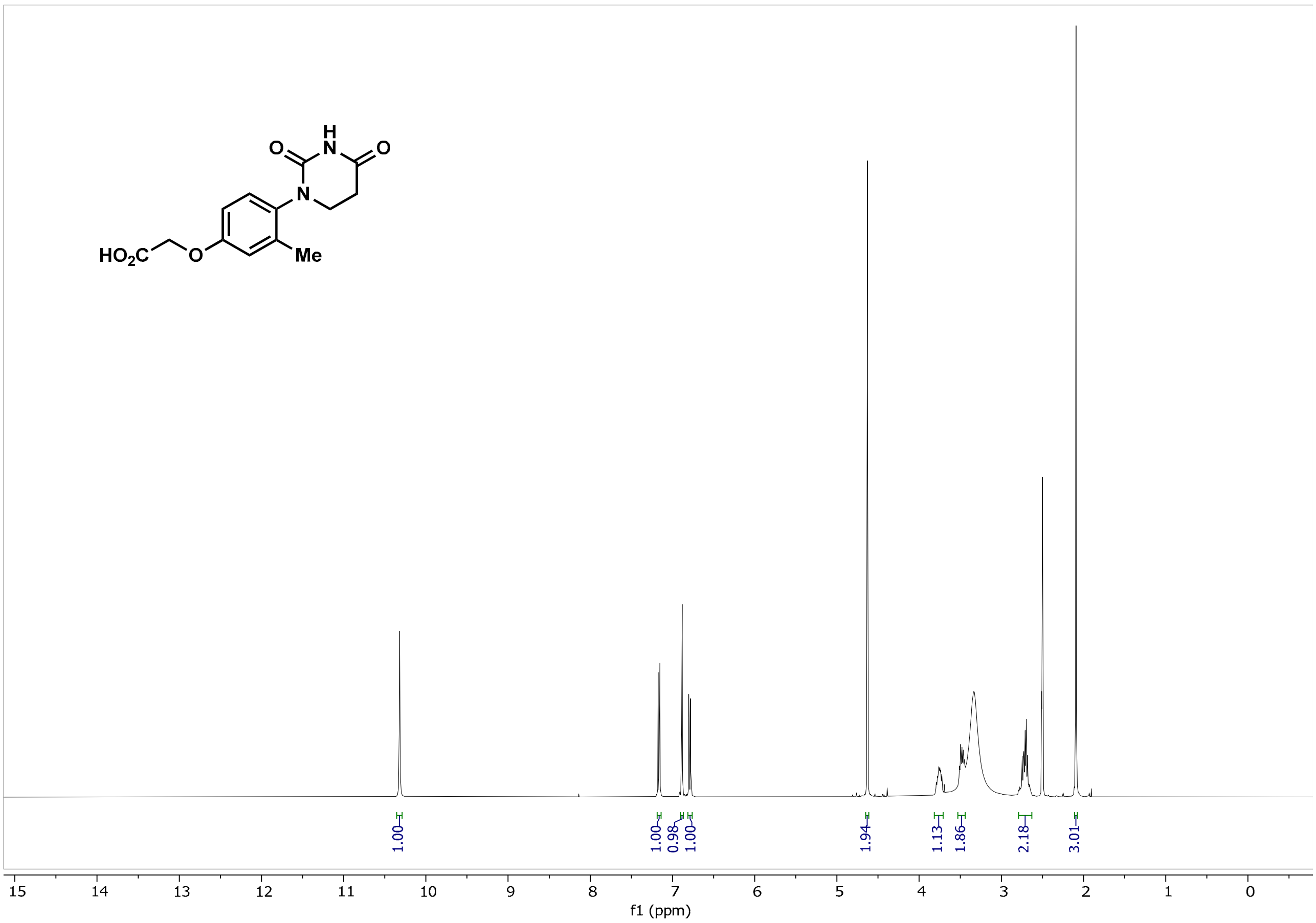
HO2C O Me N NH O O
1.00
1.00
0.98
1.00
1.94
1.13
1.86
2.18
3.01
15 14 13 12 11 10 9 8 7 6 5 4 3 2 1 0
f1 (ppm)

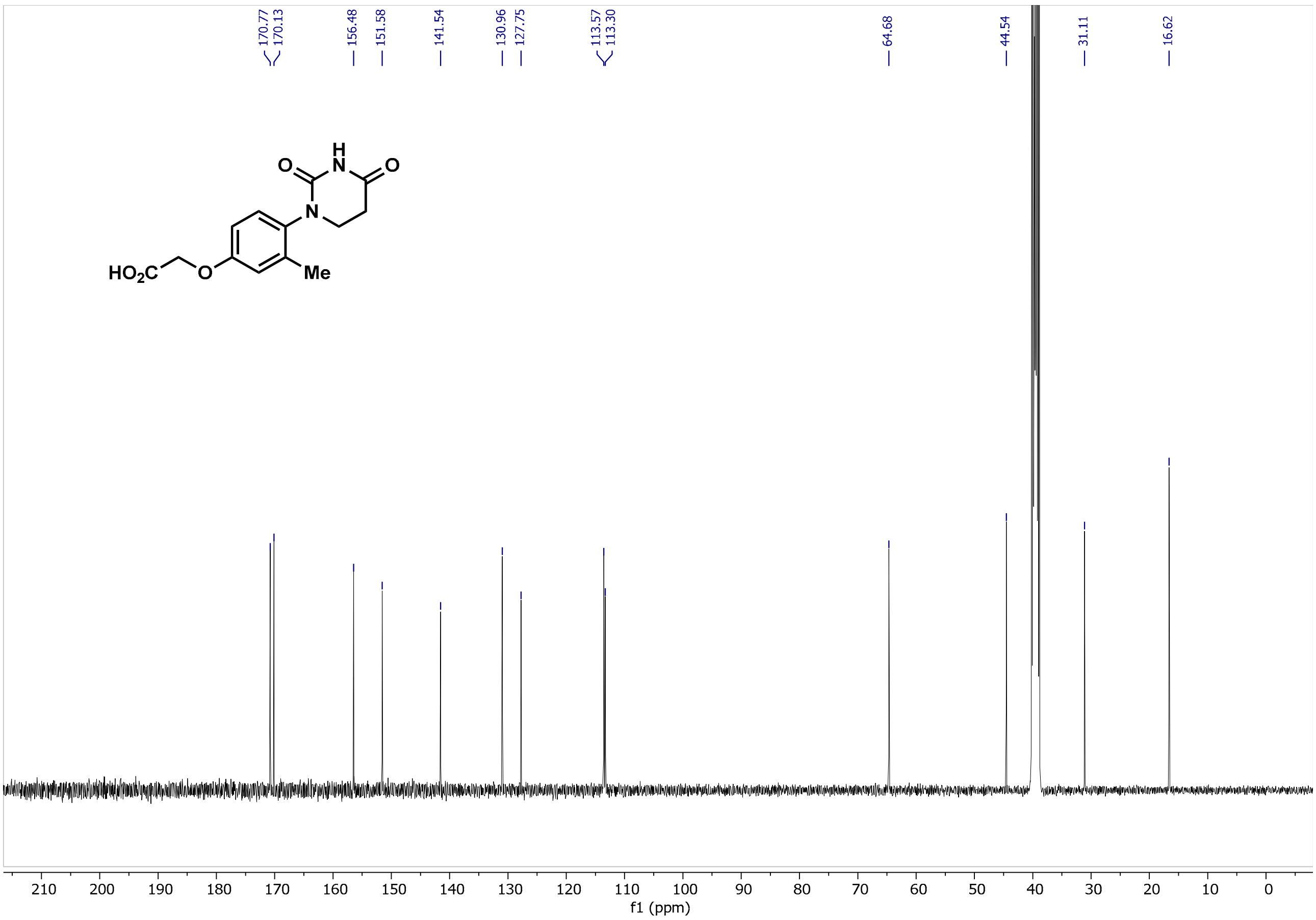
170.77
170.13
156.48
151.58
141.54
130.96
127.75
113.57
113.30
64.68
44.54
31.11
16.62
HO2C O Me N NH O O
210 200 190 180 170 160 150 140 130 120 110 100 90 80 70 60 50 40 30 20 10 0
f1 (ppm)

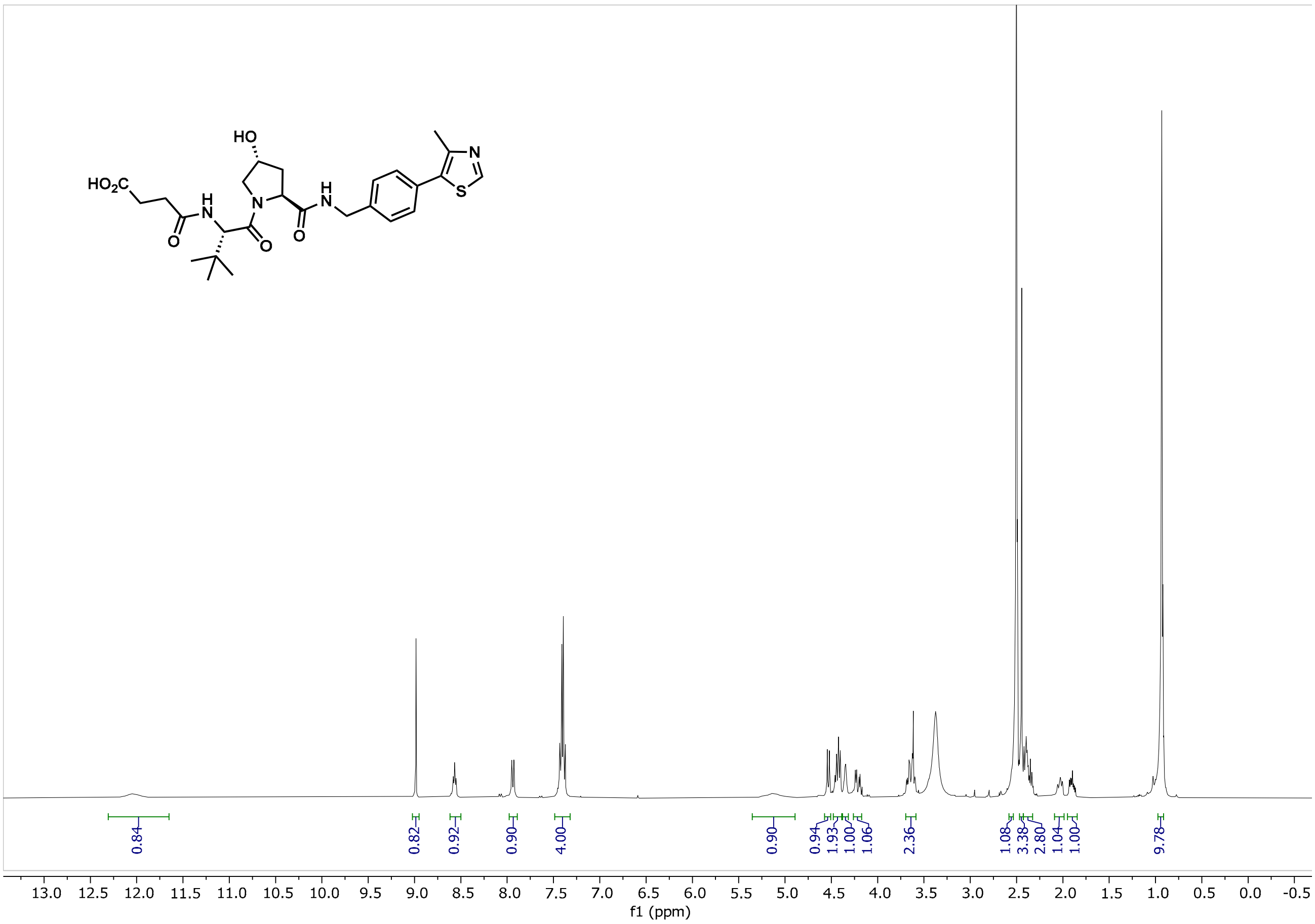
HO
HO2C
H
N
N
H
N
O
O
O
N
S
0.84
0.82
0.92
0.90
4.00
0.90
0.94
1.93
1.00
1.06
2.36
1.08
3.38
2.80
1.04
1.00
9.78
13.0 12.5 12.0 11.5 11.0 10.5 10.0 9.5 9.0 8.5 8.0 7.5 7.0 6.5 6.0 5.5 5.0 4.5 4.0 3.5 3.0 2.5 2.0 1.5 1.0 0.5 0.0 -0.5
f1 (ppm)

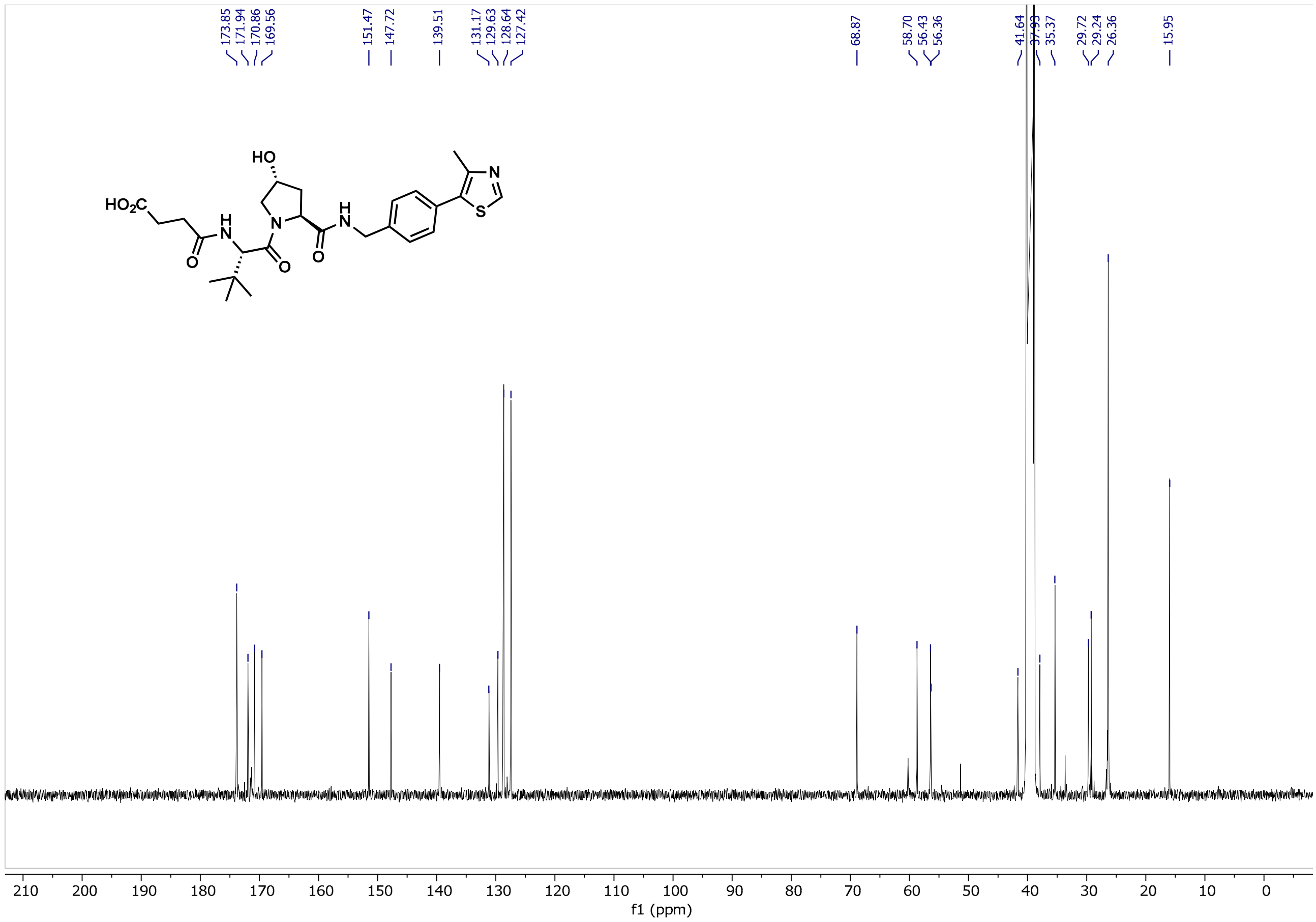
173.85
171.94
170.86
169.56
151.47
147.72
139.51
131.17
129.63
128.64
127.42
68.87
58.70
56.43
56.36
41.64
37.93
35.37
29.72
29.24
26.36
15.95
HO
HO2C
H
N
N
H
N
O
O
O
N
S
210 200 190 180 170 160 150 140 130 120 110 100 90 80 70 60 50 40 30 20 10 0
f1 (ppm)

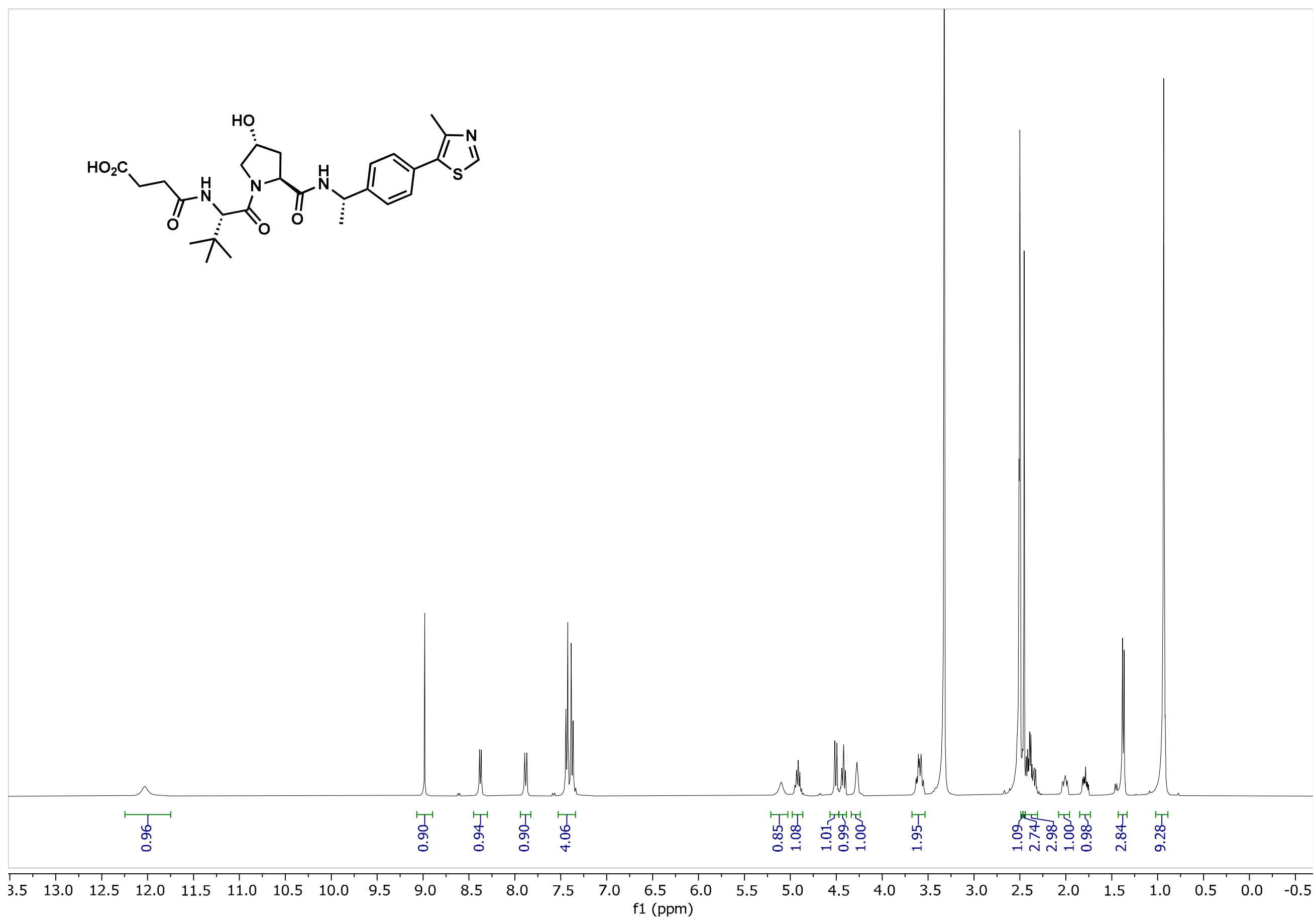
HO
HO2C
H
N
N
O
O
H
N
O
N
S
0.96
0.90
0.94
0.90
4.06
0.85
1.08
1.01
0.99
1.00
1.95
1.09
2.74
2.98
1.00
0.98
2.84
9.28
13.0 12.5 12.0 11.5 11.0 10.5 10.0 9.5 9.0 8.5 8.0 7.5 7.0 6.5 6.0 5.5 5.0 4.5 4.0 3.5 3.0 2.5 2.0 1.5 1.0 0.5 0.0 -0.5
f1 (ppm)

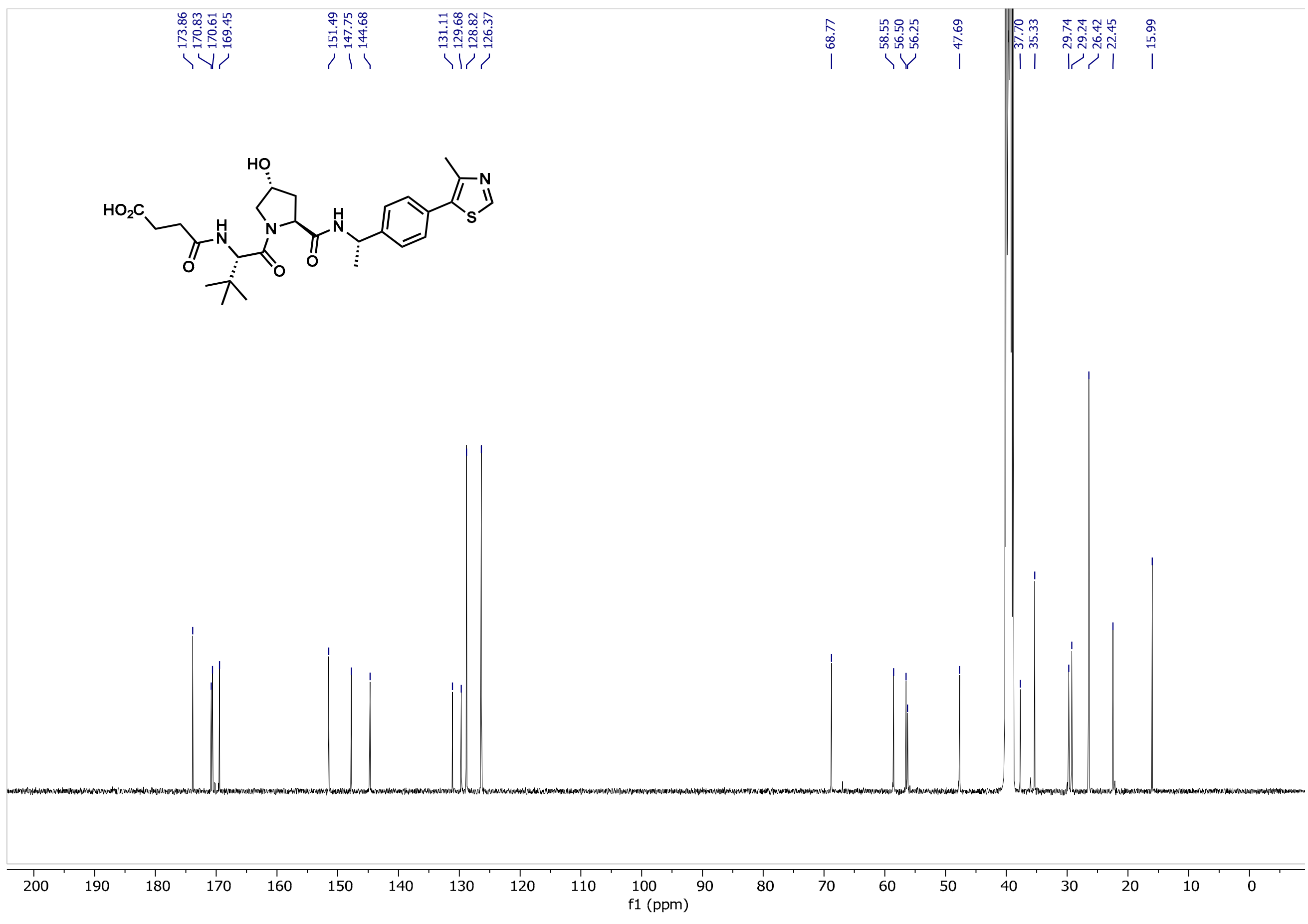
173.86
170.83
170.61
169.45
151.49
147.75
144.68
131.11
129.68
128.82
126.37
68.77
58.55
56.50
56.25
47.69
37.70
35.33
29.74
29.24
26.42
22.45
15.99
HO
HO2C
H
N
N
O
O
H
N
O
N
S
200 190 180 170 160 150 140 130 120 110 100 90 80 70 60 50 40 30 20 10 0
f1 (ppm)

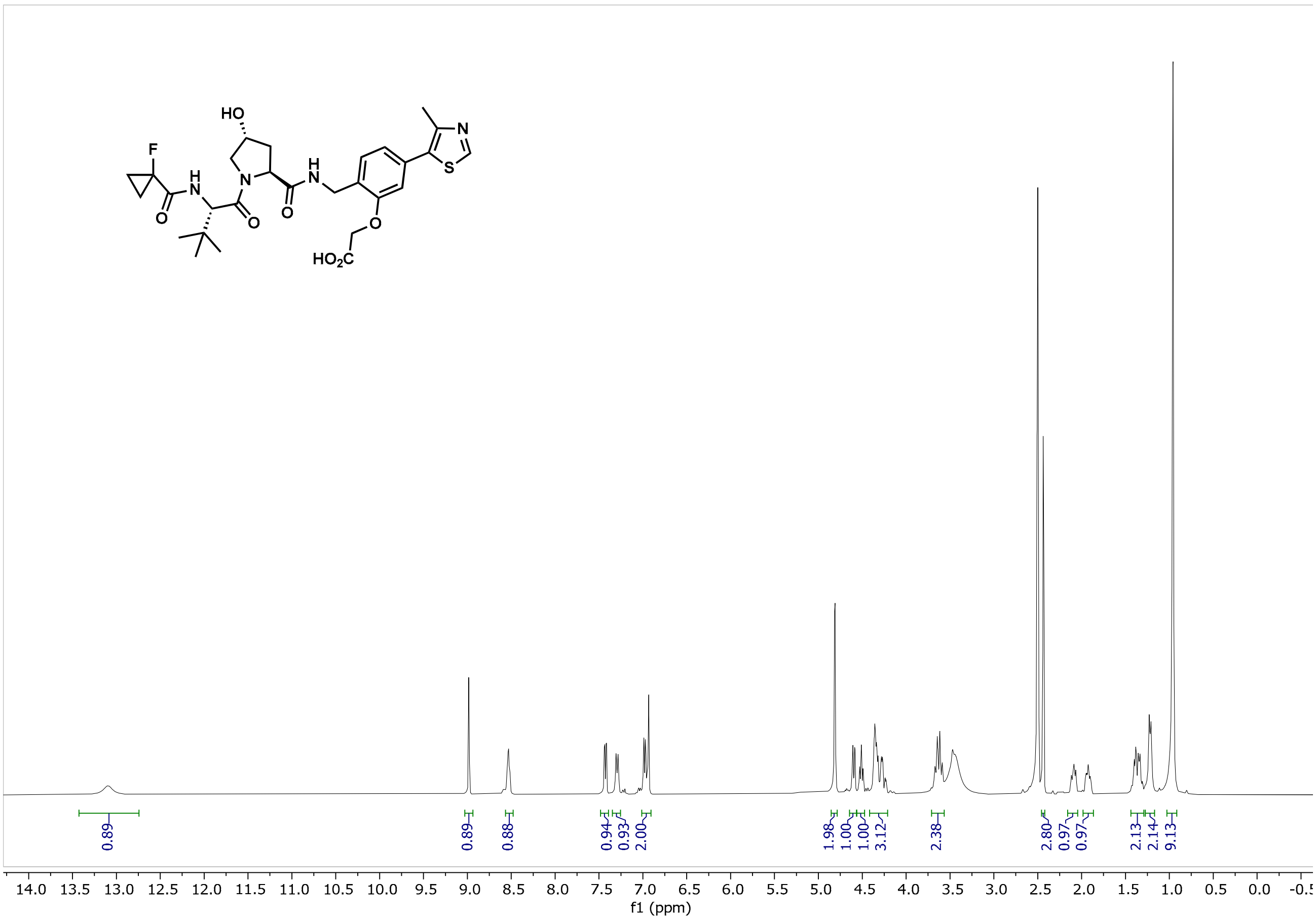

HO
F
H
N
N
O
O
H
N
O
O
HO2C
N
S
0.89
0.89
0.88
0.94
0.93
2.00
1.98
1.00
1.00
3.12
2.38
2.80
0.97
0.97
2.13
2.14
9.13
14.0 13.5 13.0 12.5 12.0 11.5 11.0 10.5 10.0 9.5 9.0 8.5 8.0 7.5 7.0 6.5 6.0 5.5 5.0 4.5 4.0 3.5 3.0 2.5 2.0 1.5 1.0 0.5 0.0 -0.5
f1 (ppm)

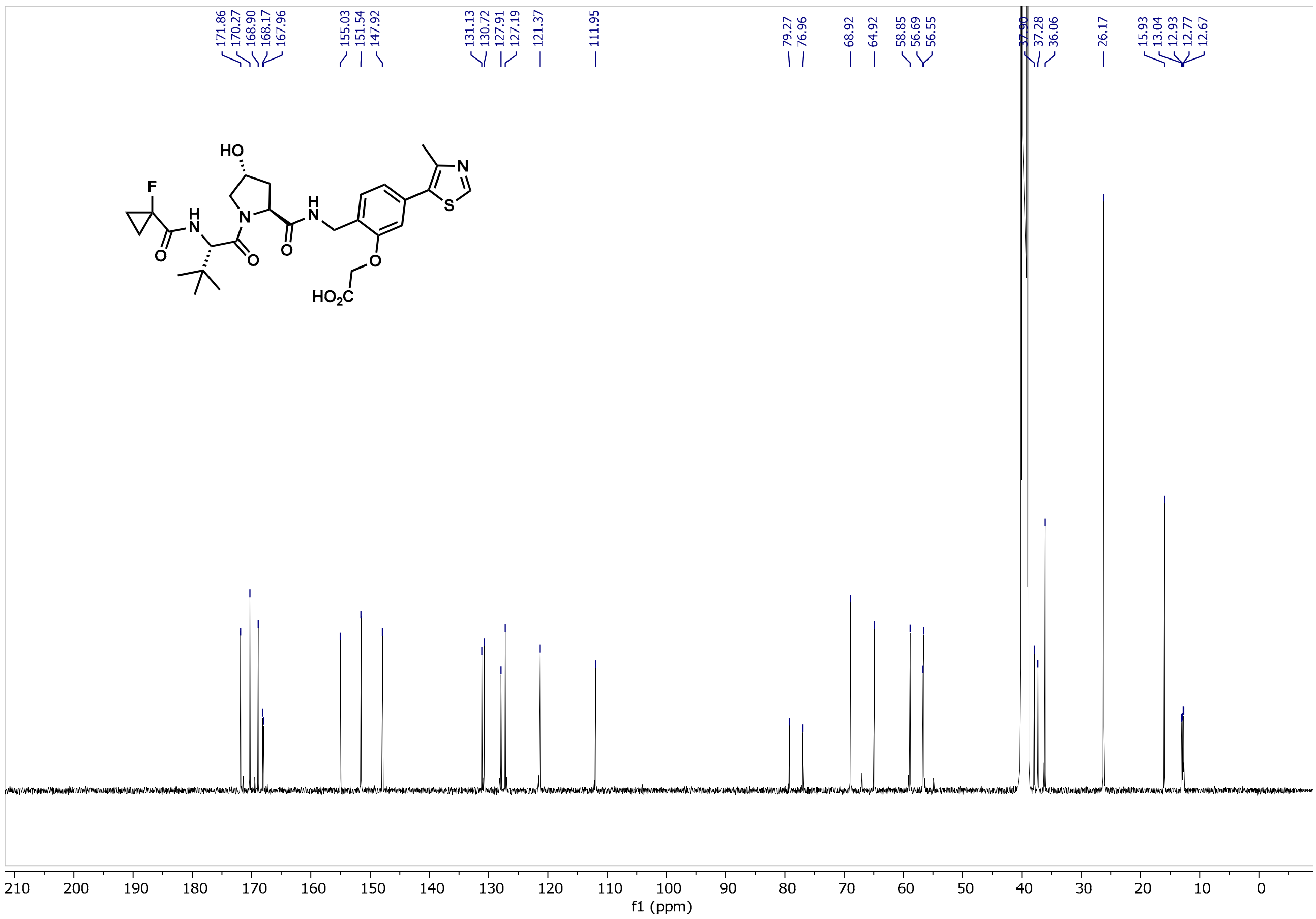

171.86
170.27
168.90
168.17
167.96
155.03
151.54
147.92
131.13
130.72
127.91
127.19
121.37
111.95
79.27
76.96
68.92
64.92
58.85
56.69
56.55
37.50
37.28
36.06
26.17
15.93
13.04
12.93
12.77
12.67
HO
F
H
N
N
O
O
H
N
O
O
HO2C
N
S
210 200 190 180 170 160 150 140 130 120 110 100 90 80 70 60 50 40 30 20 10 0
f1 (ppm)

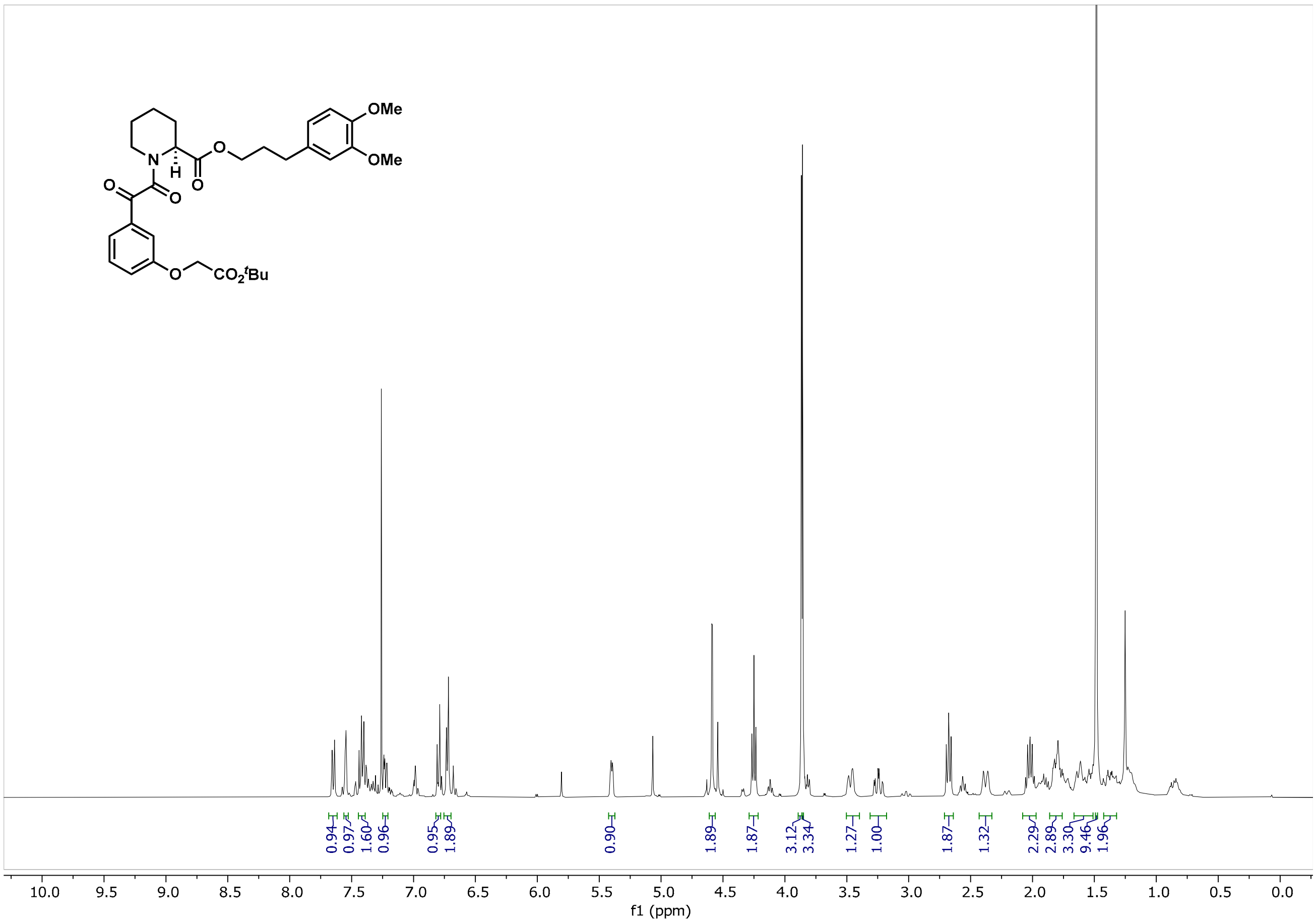
OMe
OMe
N
H
O
O
O
O
O
O
CO2tBu
0.94
0.97
1.60
0.96
0.95
1.89
0.90
1.89
1.87
3.12
3.34
1.27
1.00
1.87
1.32
2.29
2.89
3.30
9.46
1.96
10.0
9.5
9.0
8.5
8.0
7.5
7.0
6.5
6.0
5.5
5.0
4.5
4.0
3.5
3.0
2.5
2.0
1.5
1.0
0.5
0.0
f1 (ppm)

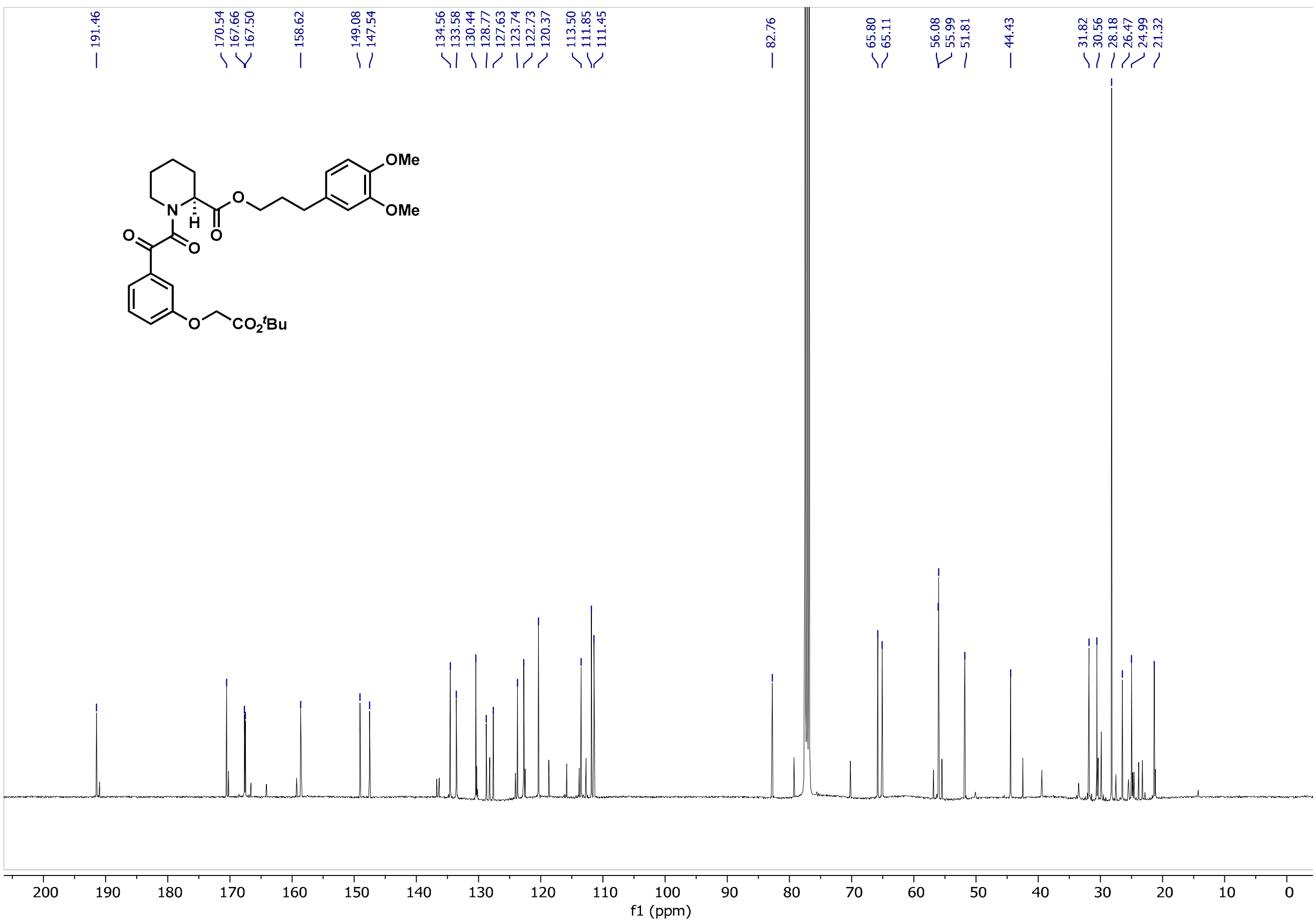
191.46
170.54
167.66
167.50
158.62
149.08
147.54
134.56
133.58
130.44
128.77
127.63
123.74
122.73
120.37
113.50
111.85
111.45
82.76
65.80
65.11
56.08
55.99
51.81
44.43
31.82
30.56
28.18
26.47
24.99
21.32
OMe
OMe
N
H
O
O
O
O
O
O
CO2tBu
200
190
180
170
160
150
140
130
120
110
100
90
80
70
60
50
40
30
20
10
0
f1 (ppm)

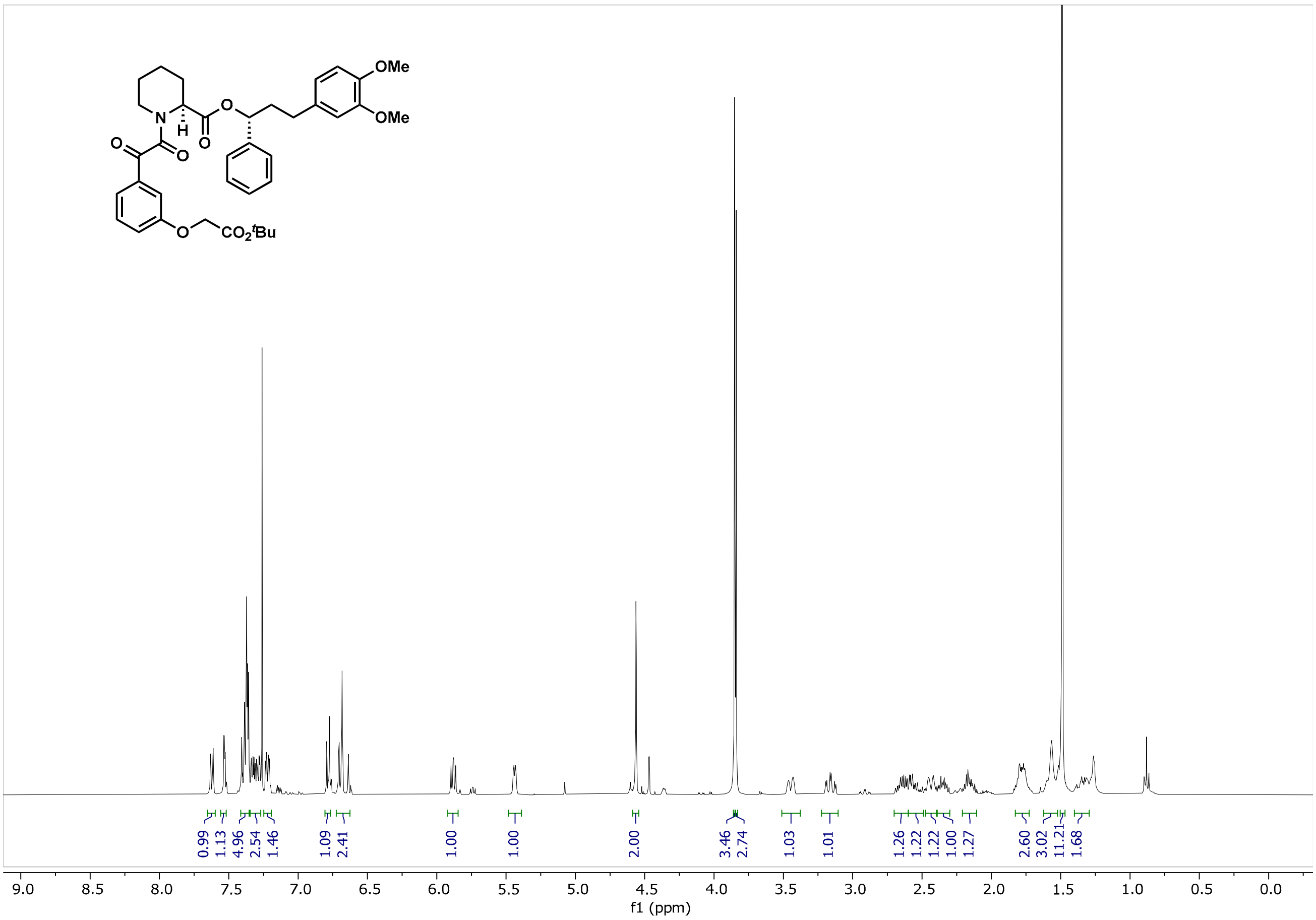
OMe
OMe
N
H
O
O
O
O
O
CO2tBu
0.99
1.13
4.96
2.54
1.46
1.09
2.41
1.00
1.00
2.00
3.46
2.74
1.03
1.01
1.26
1.22
1.22
1.00
1.27
2.60
3.02
11.21
1.68
9.0 8.5 8.0 7.5 7.0 6.5 6.0 5.5 5.0 4.5 4.0 3.5 3.0 2.5 2.0 1.5 1.0 0.5 0.0
f1 (ppm)

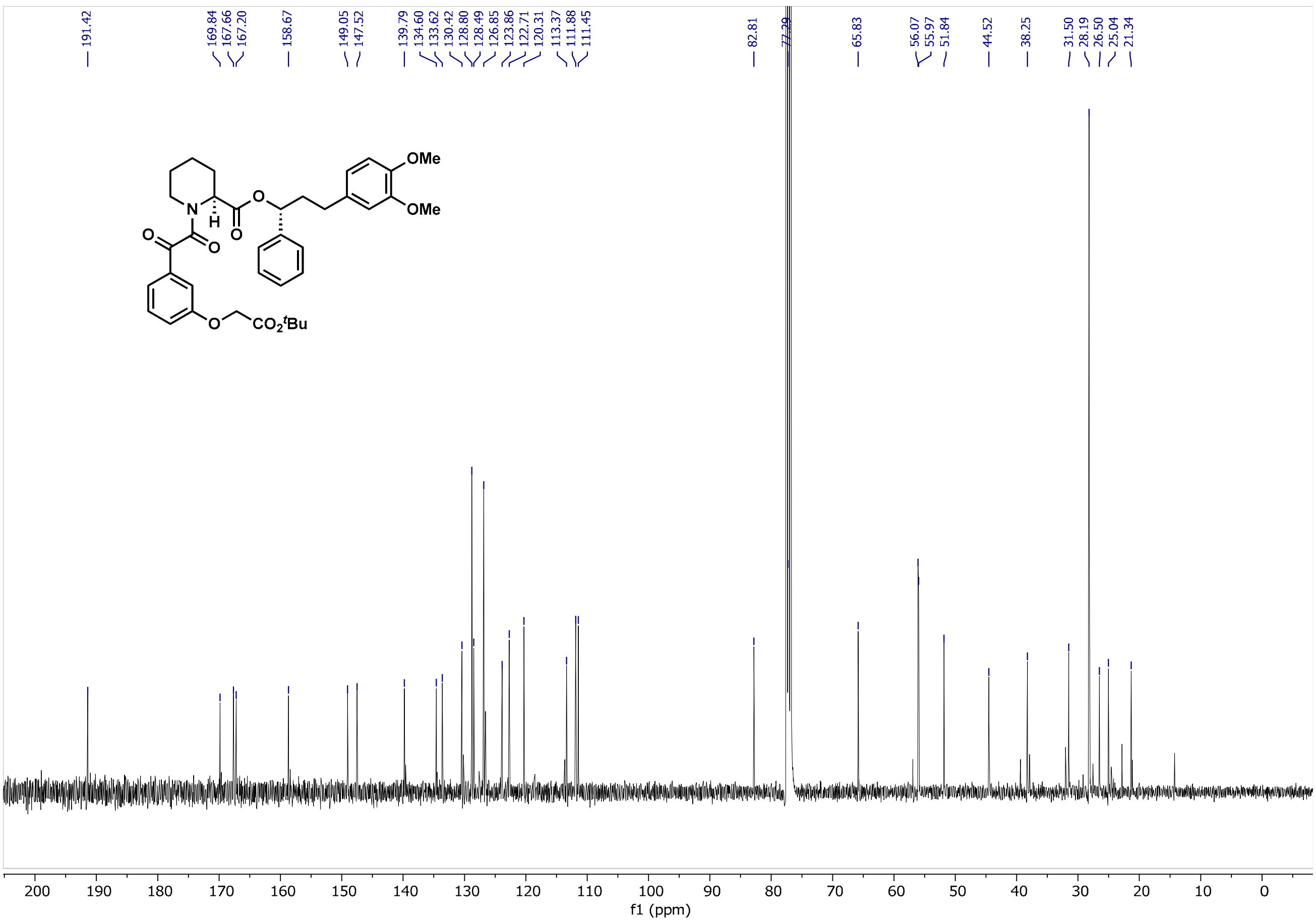
191.42
169.84
167.66
167.20
158.67
149.05
147.52
139.79
134.60
133.62
130.42
128.80
128.49
126.85
123.86
122.71
120.31
113.37
111.88
111.45
82.81
77.29
65.83
56.07
55.97
51.84
44.52
38.25
31.50
28.19
26.50
25.04
21.34
OMe
OMe
N
H
O
O
O
O
O
CO2tBu
200 190 180 170 160 150 140 130 120 110 100 90 80 70 60 50 40 30 20 10 0
f1 (ppm)

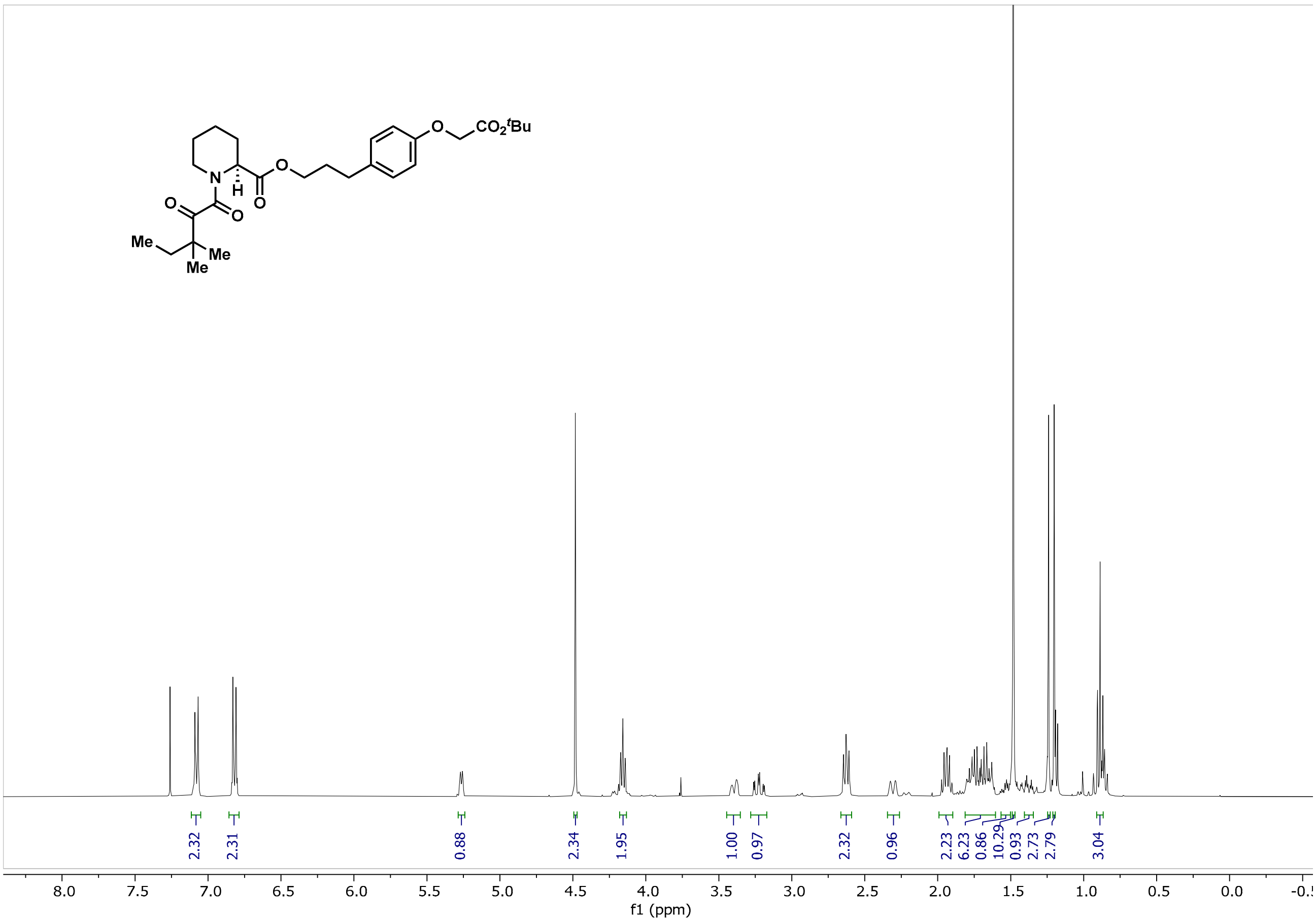
CO2tBu
Me
Me
Me
2.32
2.31
0.88
2.34
1.95
1.00
0.97
2.32
0.96
2.23
6.23
0.86
10.29
0.93
2.73
2.79
3.04
f1 (ppm)

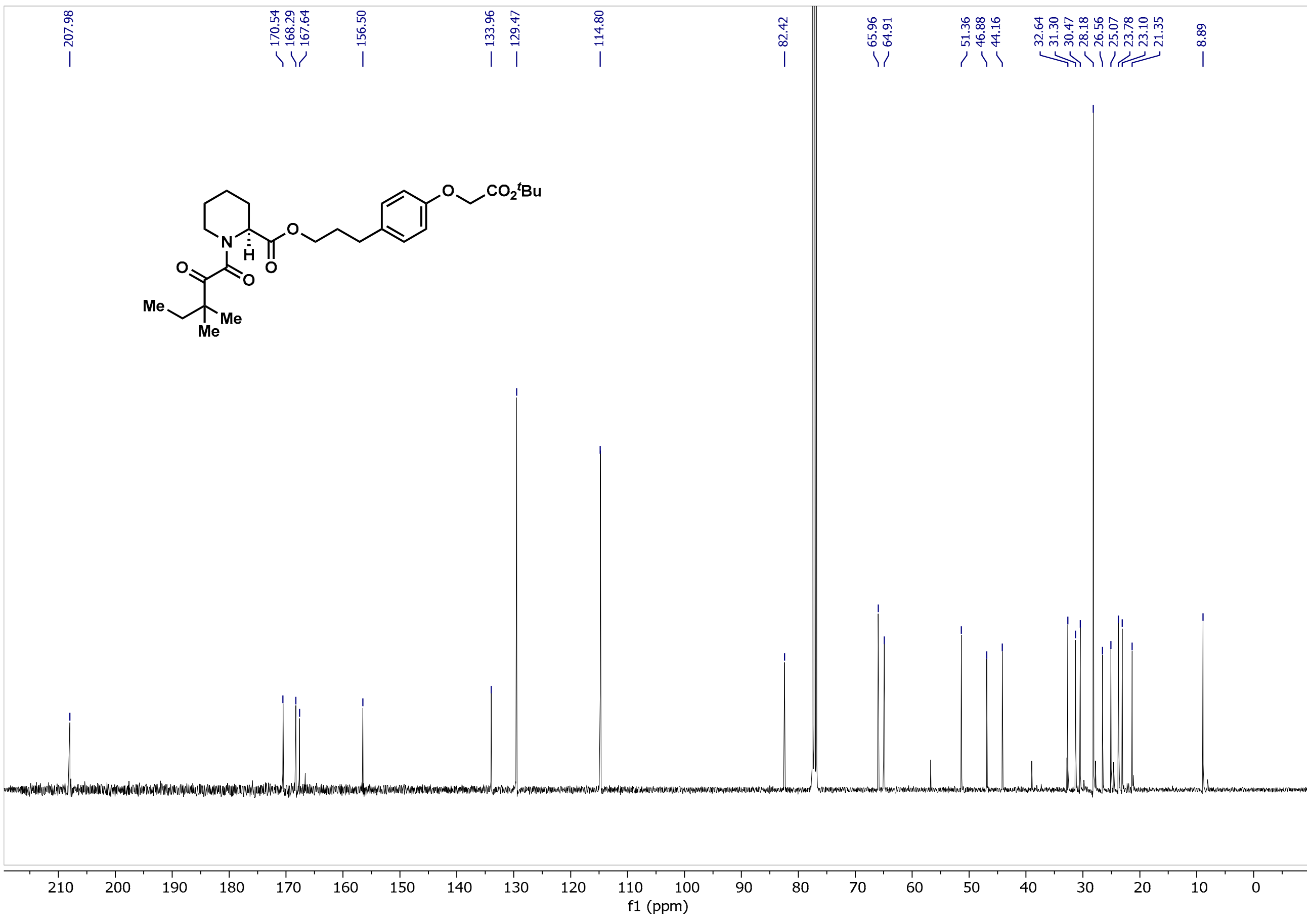
207.98
170.54
168.29
167.64
156.50
133.96
129.47
114.80
82.42
65.96
64.91
51.36
46.88
44.16
32.64
31.30
30.47
28.18
26.56
25.07
23.78
23.10
21.35
8.89
CO2tBu
Me
Me
Me
f1 (ppm)

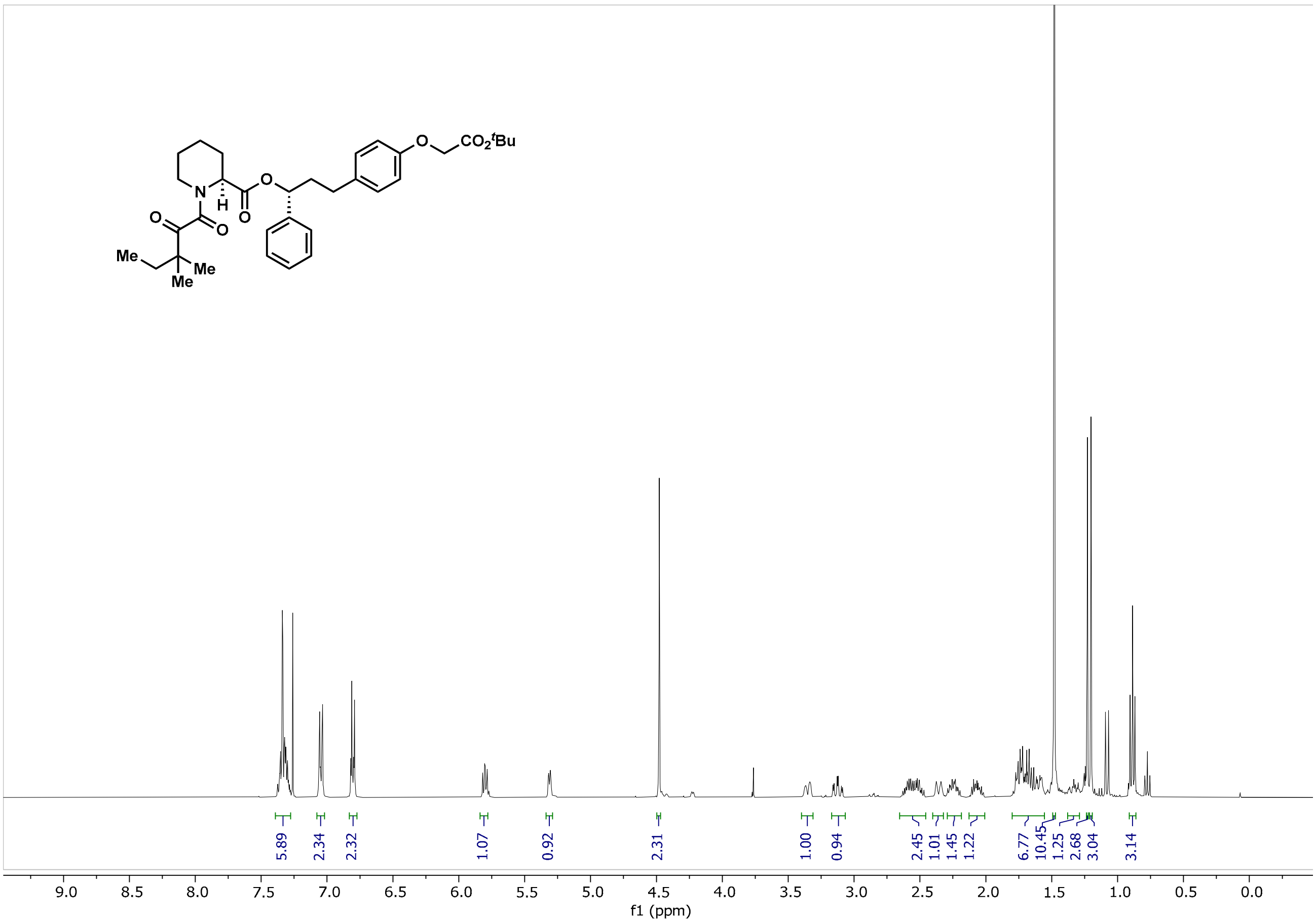

$CO_2{}^tBu$
Me
Me
Me
5.89
2.34
2.32
1.07
0.92
2.31
1.00
0.94
2.45
1.01
1.45
1.22
6.77
10.45
1.25
2.68
3.04
3.14
f1 (ppm)

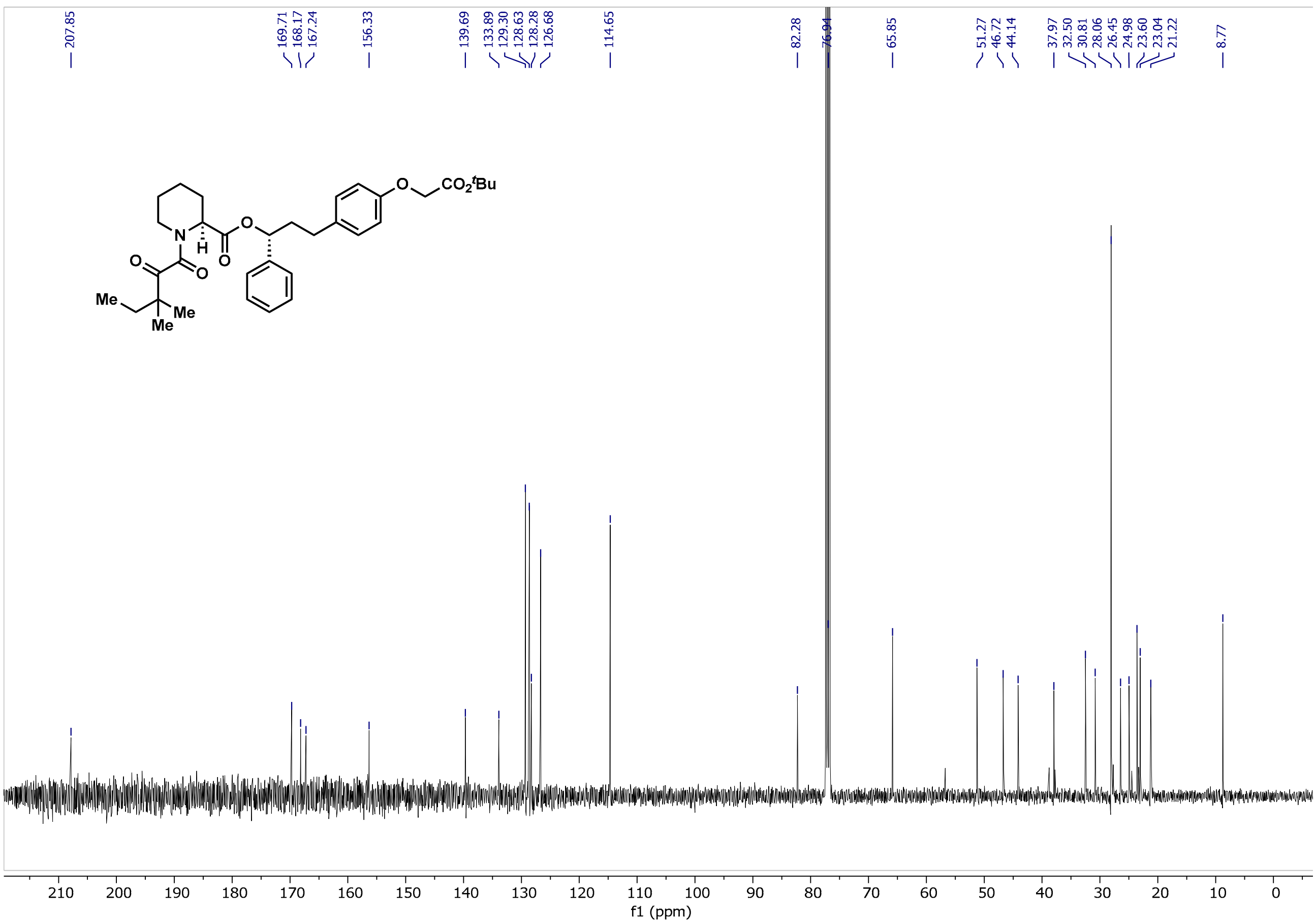

207.85
169.71
168.17
167.24
156.33
139.69
133.89
129.30
128.63
128.28
126.68
114.65
82.28
65.85
51.27
46.72
44.14
37.97
32.50
30.81
28.06
26.45
24.98
23.60
23.04
21.22
8.77
$CO_2{}^tBu$
Me
Me
Me
f1 (ppm)

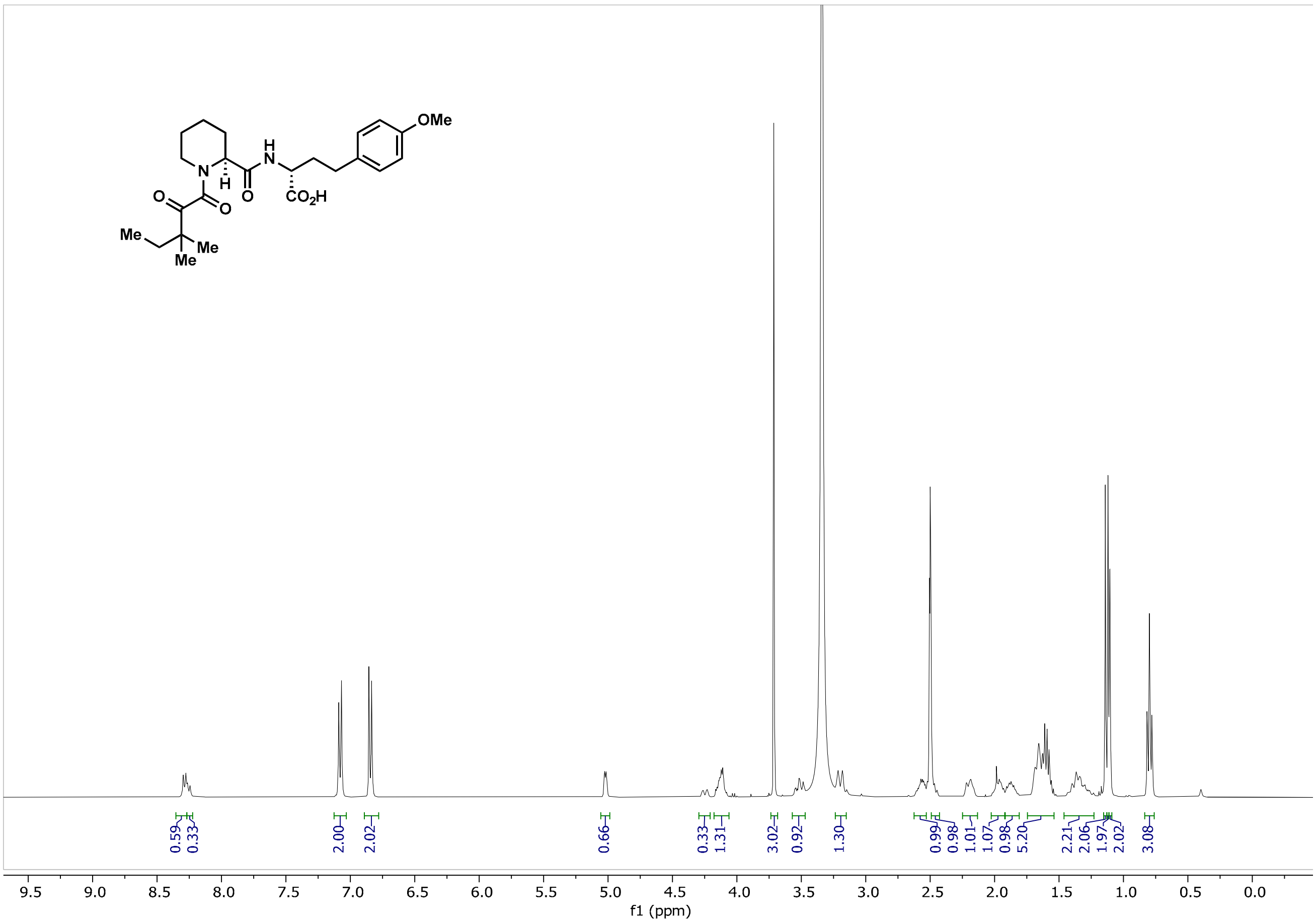
OMe
H
N
N
H
O
O
O
CO2H
Me
Me
Me
0.59
0.33
2.00
2.02
0.66
0.33
1.31
3.02
0.92
1.30
0.99
0.98
1.01
1.07
0.98
5.20
2.21
2.06
1.97
2.02
3.08
9.5 9.0 8.5 8.0 7.5 7.0 6.5 6.0 5.5 5.0 4.5 4.0 3.5 3.0 2.5 2.0 1.5 1.0 0.5 0.0
f1 (ppm)

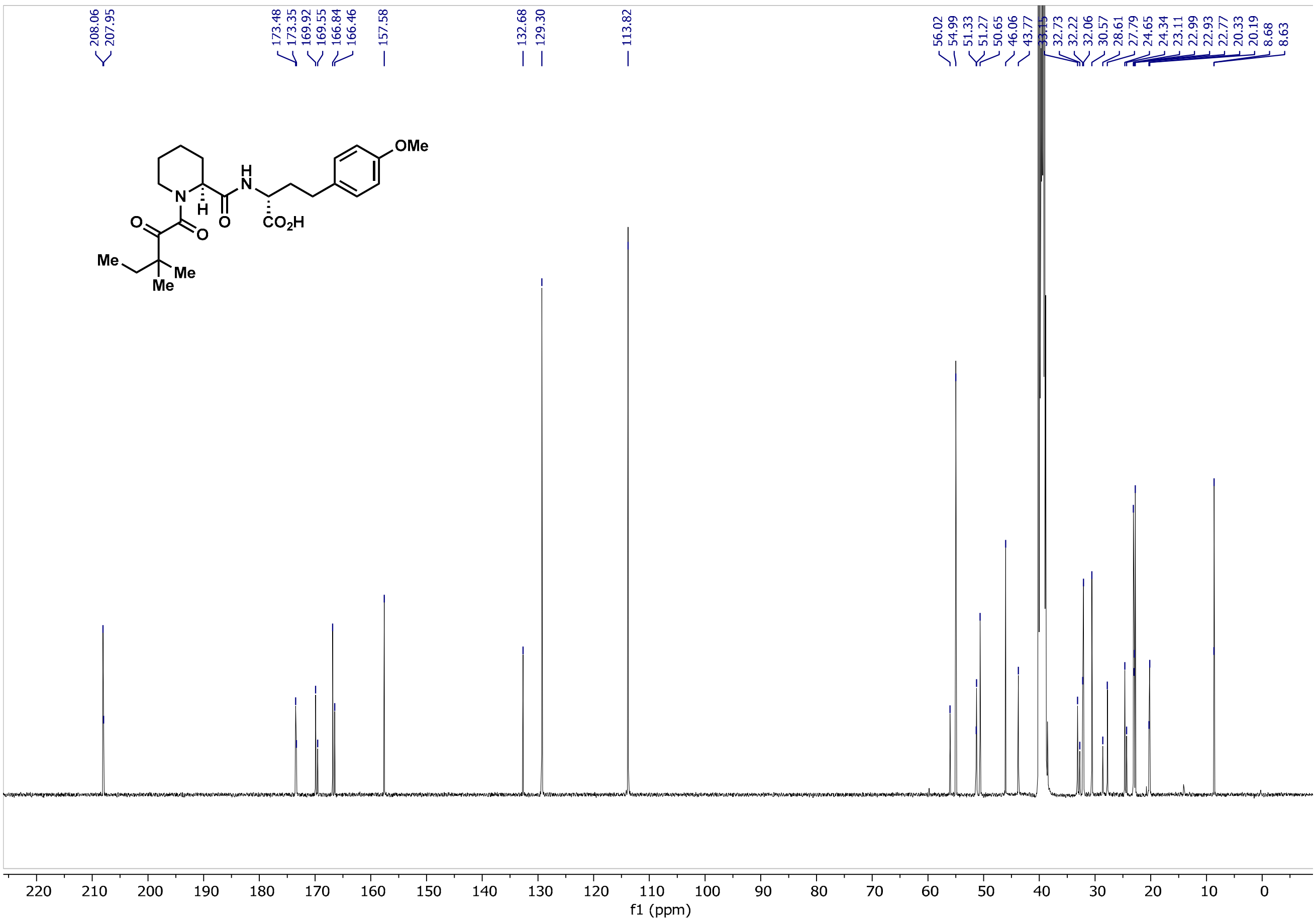
208.06
207.95
173.48
173.35
169.92
169.55
166.84
166.46
157.58
132.68
129.30
113.82
56.02
54.99
51.33
51.27
50.65
46.06
43.77
32.73
32.22
32.06
30.57
28.61
27.79
24.65
24.34
23.11
22.99
22.93
22.77
20.33
20.19
8.68
8.63
OMe
H
N
N
H
O
O
O
CO2H
Me
Me
Me
220 210 200 190 180 170 160 150 140 130 120 110 100 90 80 70 60 50 40 30 20 10 0
f1 (ppm)

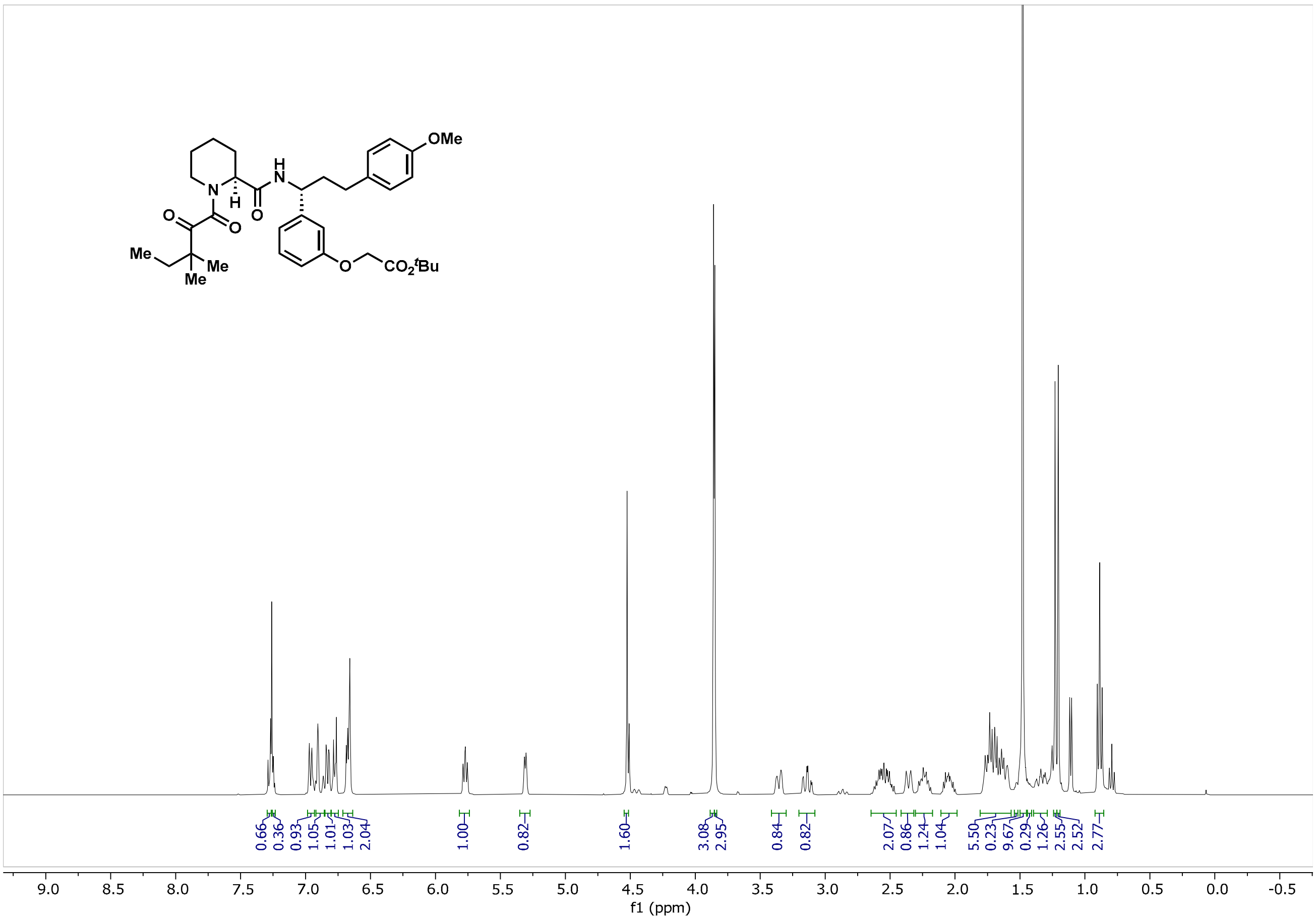
OMe
H
N
N
H
O
O
O
Me
Me
Me
O
CO2tBu
0.66
0.36
0.93
1.05
1.01
1.03
2.04
1.00
0.82
1.60
3.08
2.95
0.84
0.82
2.07
0.86
1.24
1.04
5.50
0.23
9.67
0.29
1.26
2.55
2.52
2.77
9.0
8.5
8.0
7.5
7.0
6.5
6.0
5.5
5.0
4.5
4.0
3.5
3.0
2.5
2.0
1.5
1.0
0.5
0.0
-0.5
f1 (ppm)

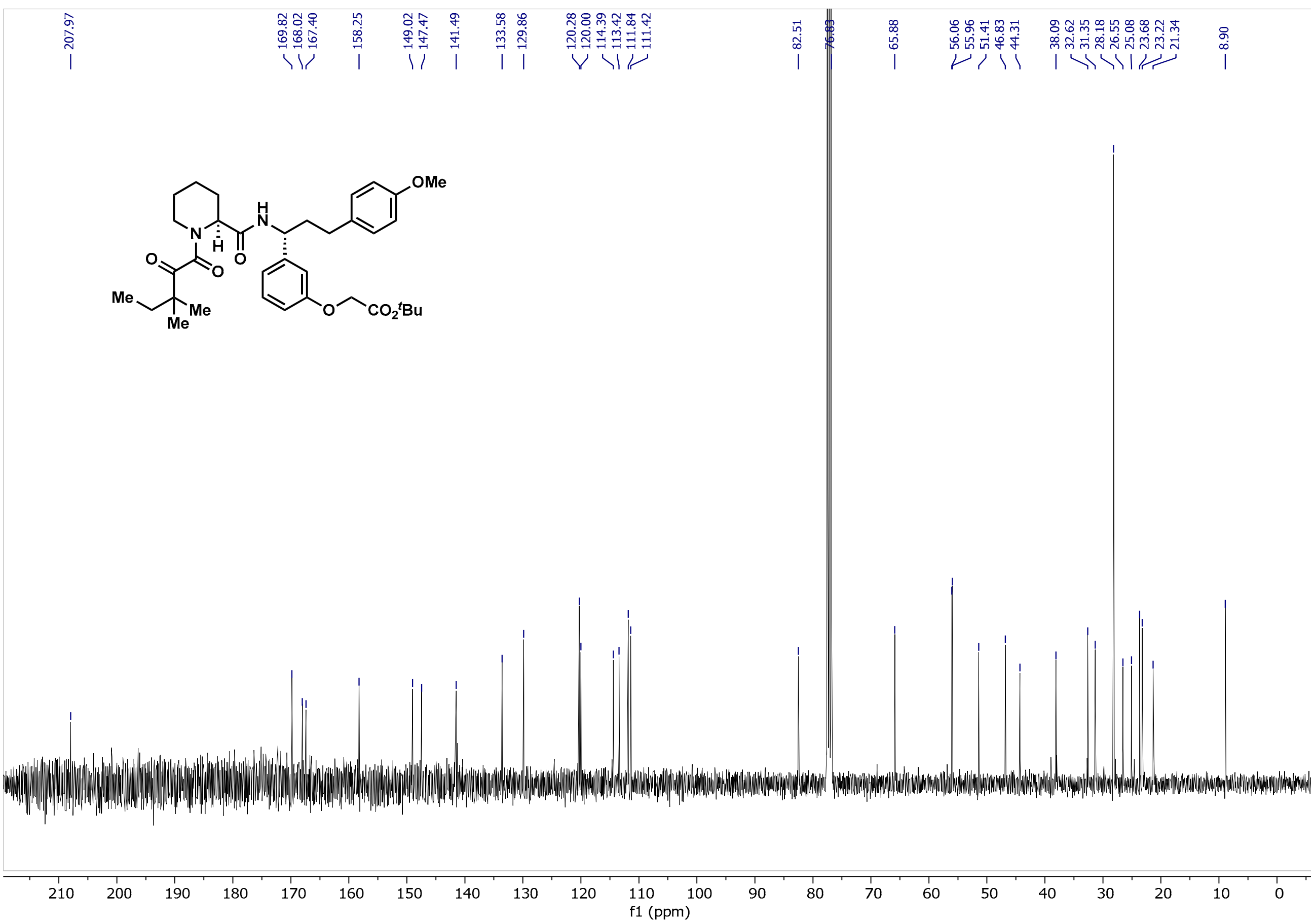
207.97
169.82
168.02
167.40
158.25
149.02
147.47
141.49
133.58
129.86
120.28
120.00
114.39
113.42
111.84
111.42
82.51
76.83
65.88
56.06
55.96
51.41
46.83
44.31
38.09
32.62
31.35
28.18
26.55
25.08
23.68
23.22
21.34
8.90
OMe
H
N
N
H
O
O
O
Me
Me
Me
O
CO2tBu
210
200
190
180
170
160
150
140
130
120
110
100
90
80
70
60
50
40
30
20
10
0
f1 (ppm)

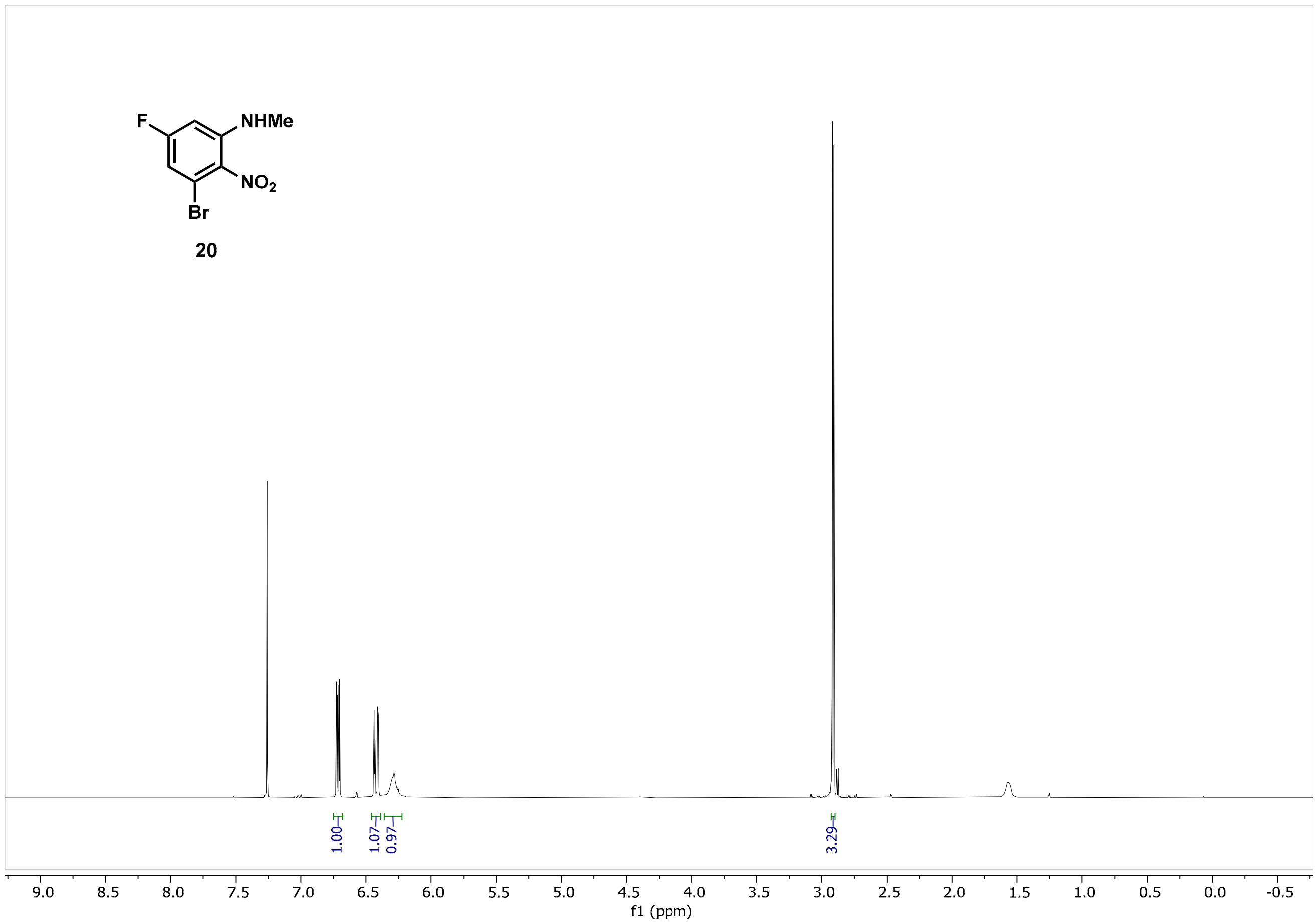

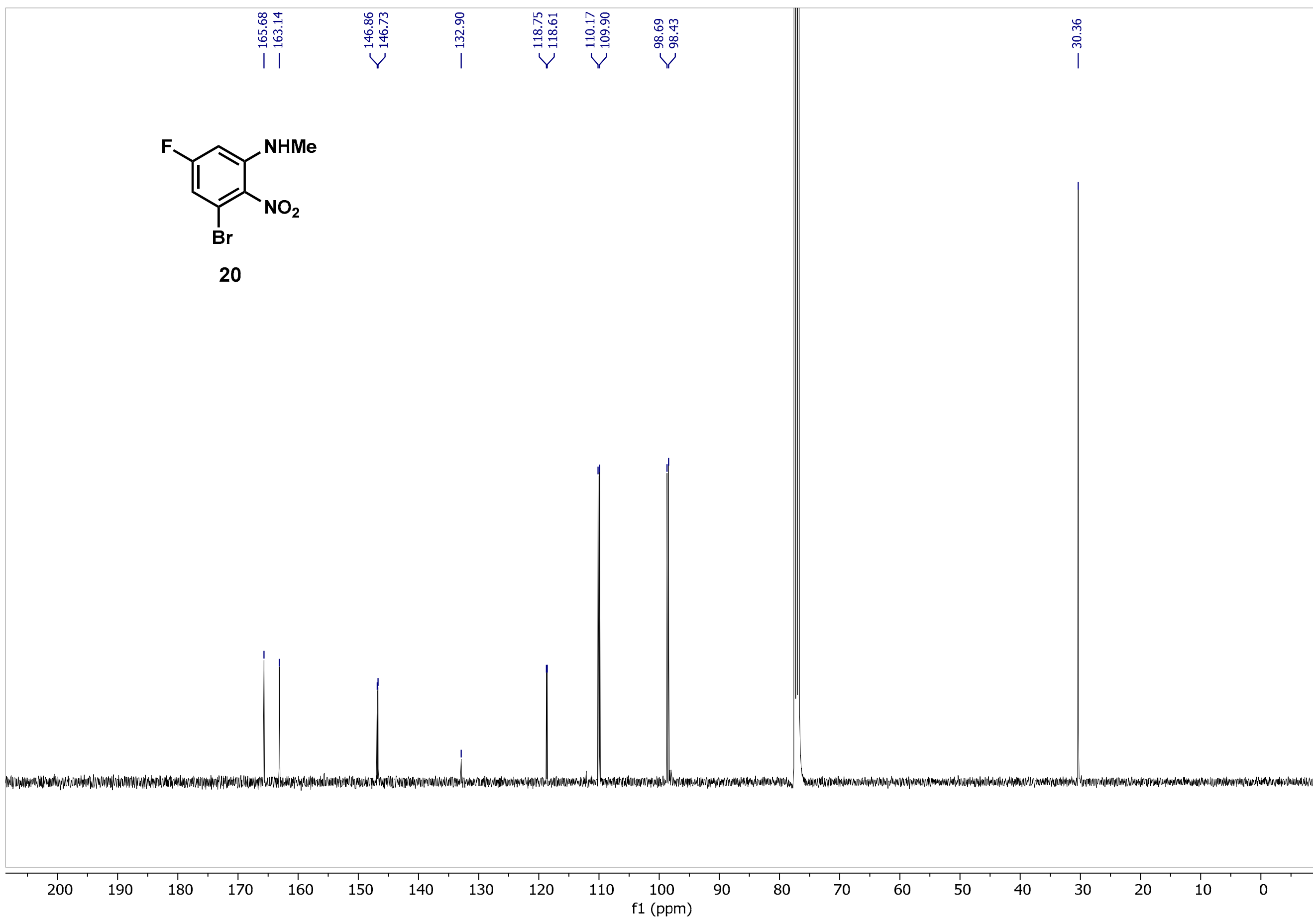

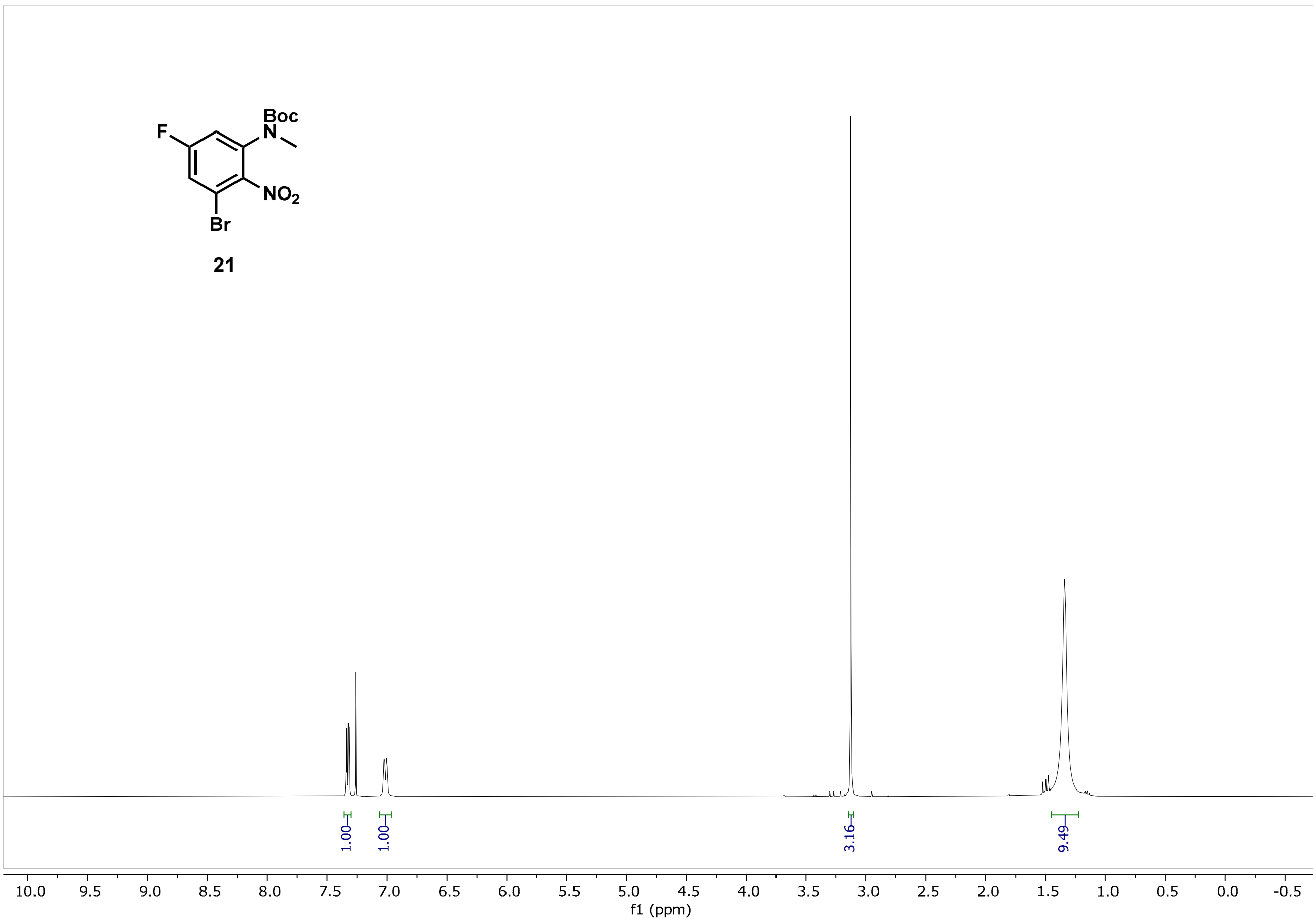

Boc
F
N
NO2
Br
21
1.00
1.00
3.16
9.49
10.0 9.5 9.0 8.5 8.0 7.5 7.0 6.5 6.0 5.5 5.0 4.5 4.0 3.5 3.0 2.5 2.0 1.5 1.0 0.5 0.0 -0.5
f1 (ppm)

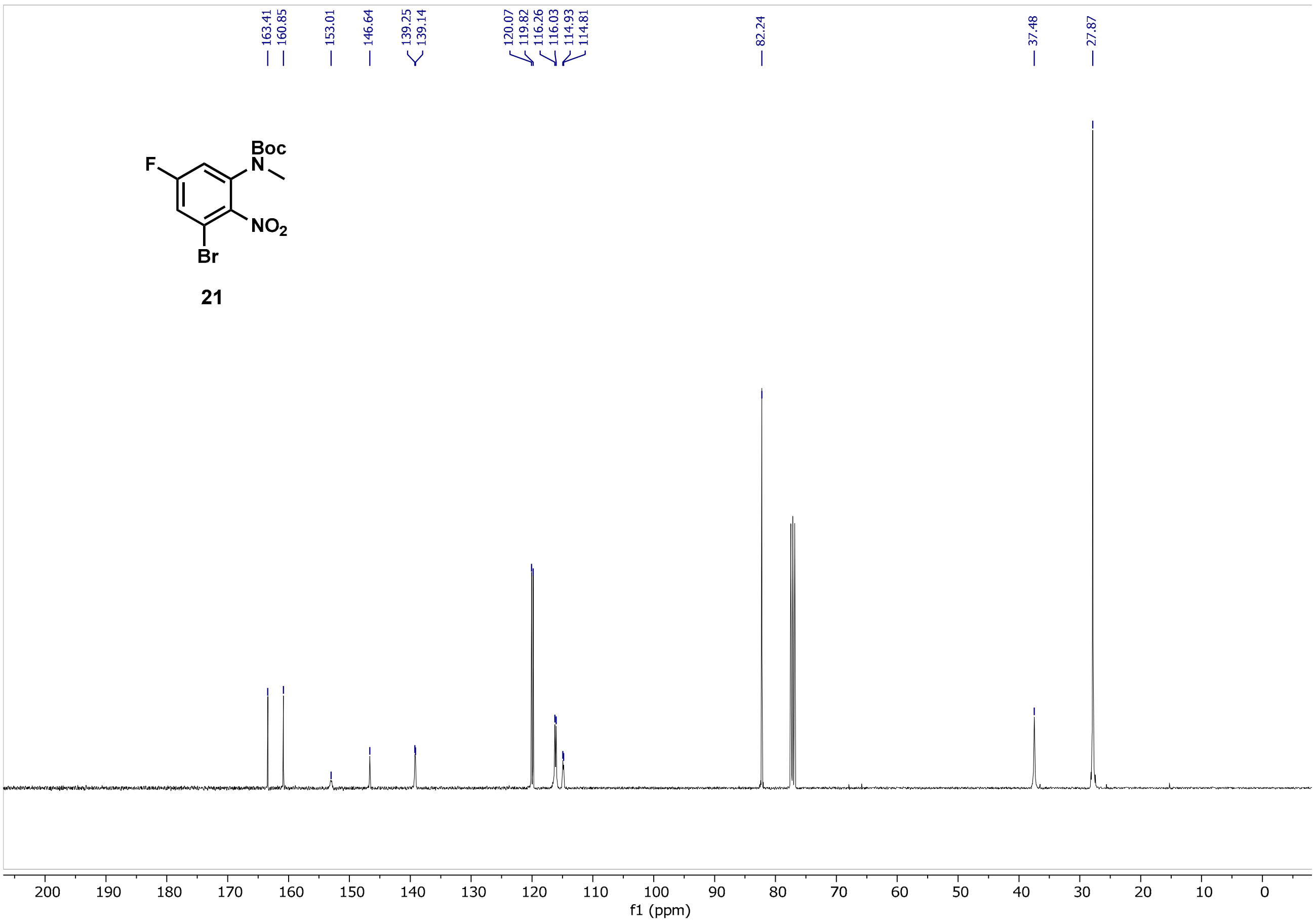

163.41
160.85
153.01
146.64
139.25
139.14
120.07
119.82
116.26
116.03
114.93
114.81
82.24
37.48
27.87
Boc
F
N
NO2
Br
21
200 190 180 170 160 150 140 130 120 110 100 90 80 70 60 50 40 30 20 10 0
f1 (ppm)

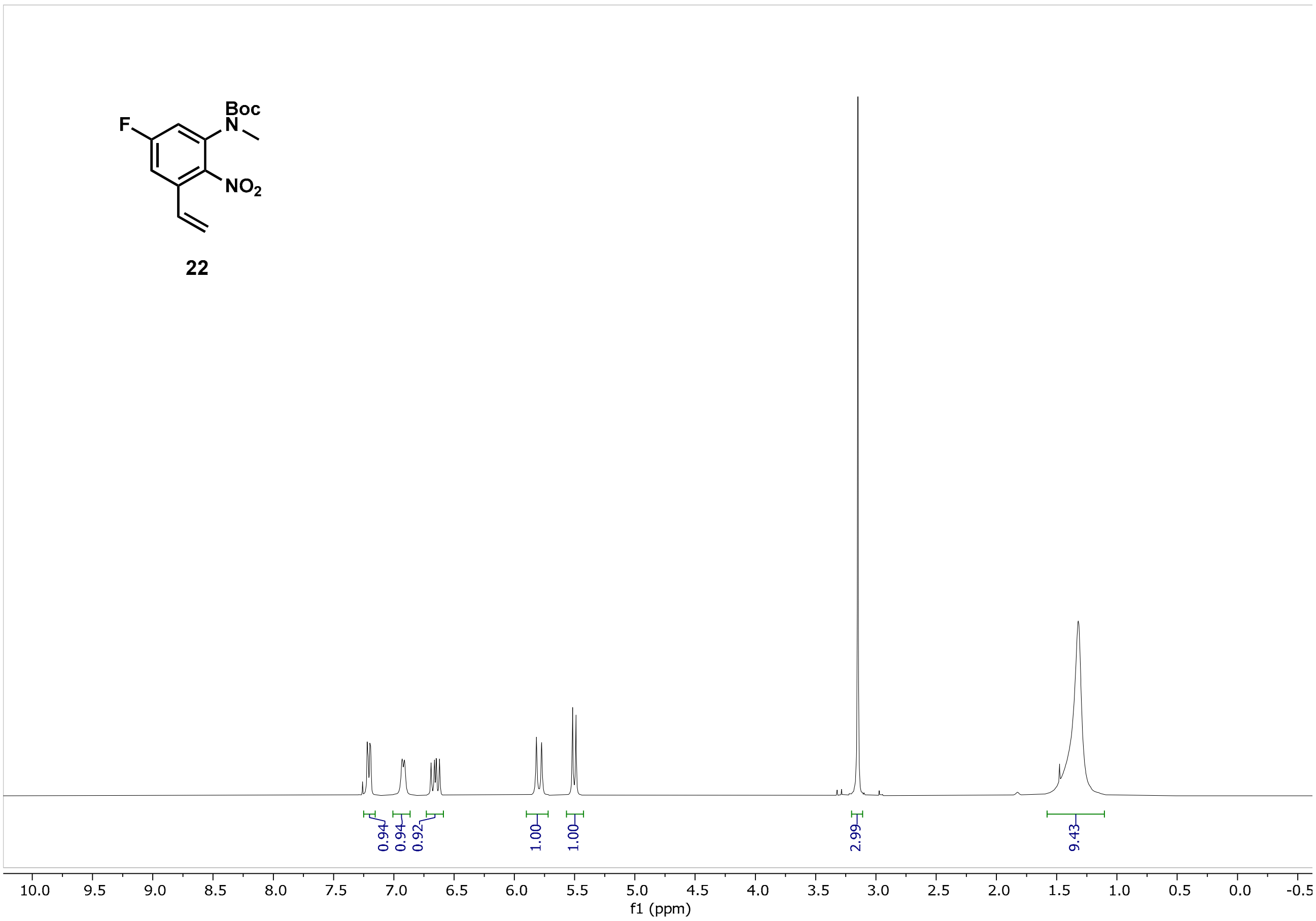
Boc
N
F
NO2
22
0.94
0.94
0.92
1.00
1.00
2.99
9.43
10.0 9.5 9.0 8.5 8.0 7.5 7.0 6.5 6.0 5.5 5.0 4.5 4.0 3.5 3.0 2.5 2.0 1.5 1.0 0.5 0.0 -0.5
f1 (ppm)

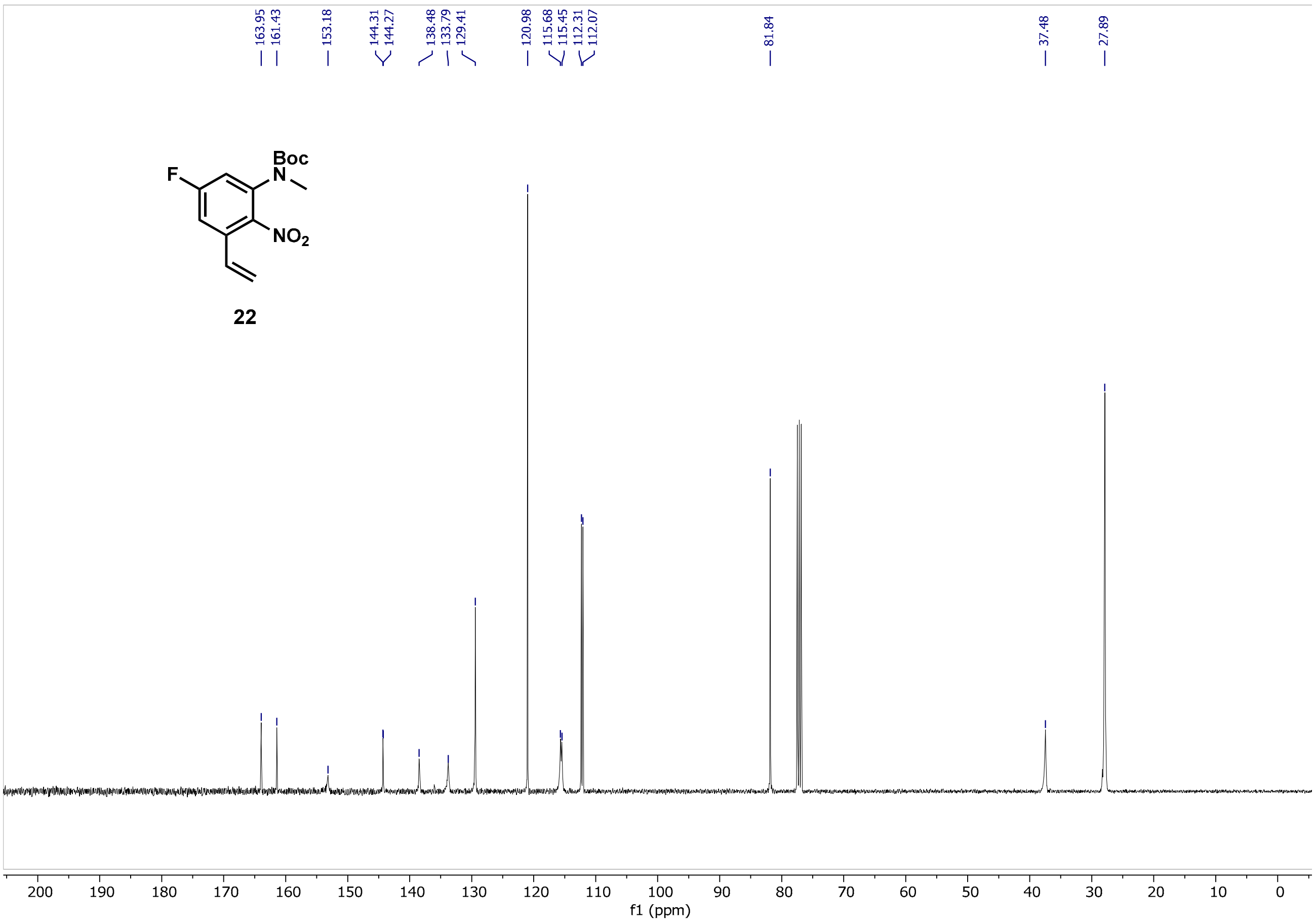
163.95
161.43
153.18
144.31
144.27
138.48
133.79
129.41
120.98
115.68
115.45
112.31
112.07
81.84
37.48
27.89
Boc
N
F
NO2
22
200 190 180 170 160 150 140 130 120 110 100 90 80 70 60 50 40 30 20 10 0
f1 (ppm)

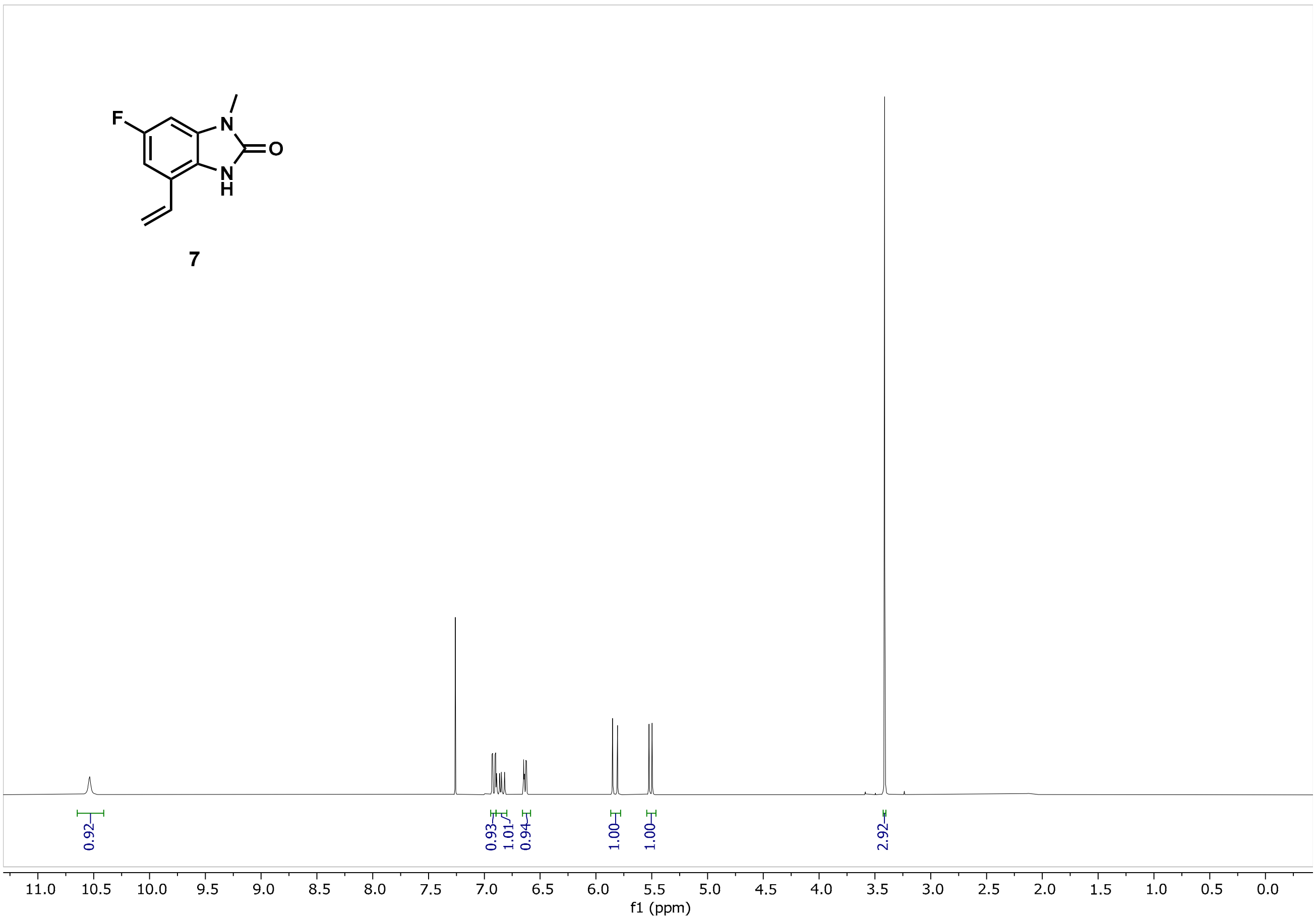
F
N
O
N
H
7
0.92
0.93
1.01
0.94
1.00
1.00
2.92
11.0 10.5 10.0 9.5 9.0 8.5 8.0 7.5 7.0 6.5 6.0 5.5 5.0 4.5 4.0 3.5 3.0 2.5 2.0 1.5 1.0 0.5 0.0
f1 (ppm)

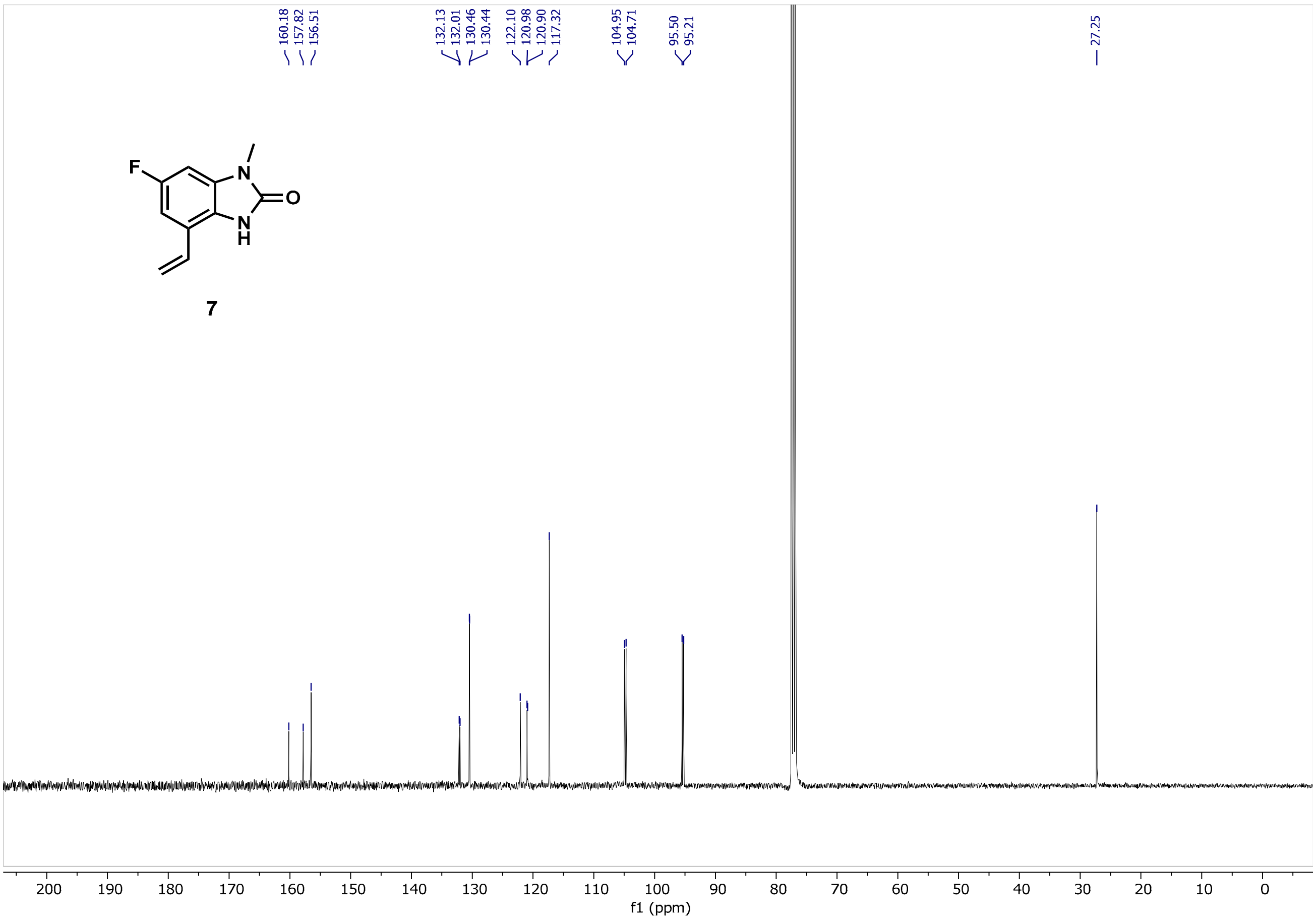
160.18
157.82
156.51
132.13
132.01
130.46
130.44
122.10
120.98
120.90
117.32
104.95
104.71
95.50
95.21
27.25
F
N
O
N
H
7
200 190 180 170 160 150 140 130 120 110 100 90 80 70 60 50 40 30 20 10 0
f1 (ppm)

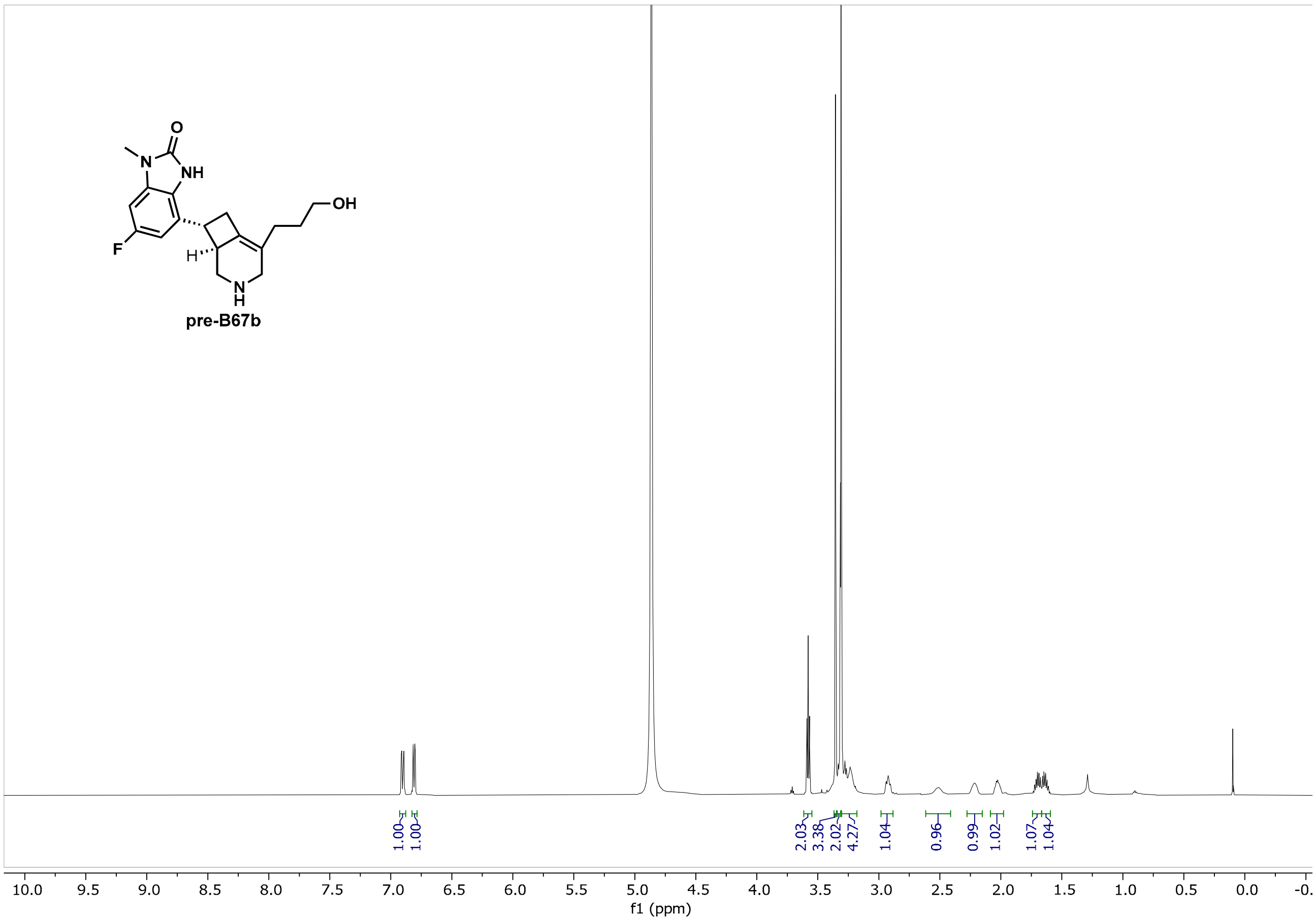
O
N
NH
OH
F
H
N
H
pre-B67b
1.00
1.00
2.03
3.38
2.02
4.27
1.04
0.96
0.99
1.02
1.07
1.04
10.0 9.5 9.0 8.5 8.0 7.5 7.0 6.5 6.0 5.5 5.0 4.5 4.0 3.5 3.0 2.5 2.0 1.5 1.0 0.5 0.0
f1 (ppm)

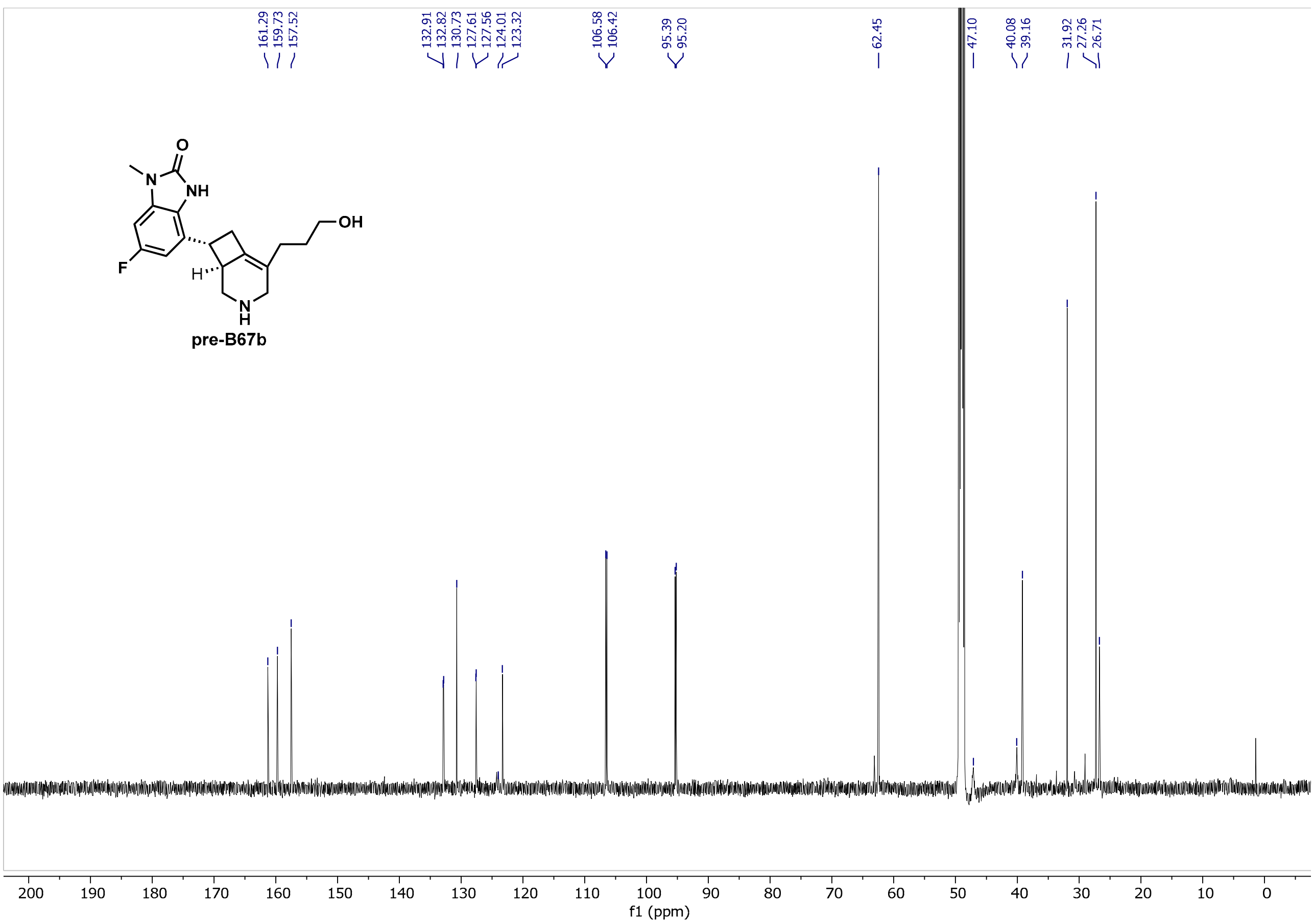
161.29
159.73
157.52
132.91
132.82
130.73
127.61
127.56
124.01
123.32
106.58
106.42
95.39
95.20
62.45
47.10
40.08
39.16
31.92
27.26
26.71
O
N
NH
OH
F
H
N
H
pre-B67b
200 190 180 170 160 150 140 130 120 110 100 90 80 70 60 50 40 30 20 10 0
f1 (ppm)

Atom	δ (ppm)	Min..Max (ppm)	HSQC
1 N	115.83	115.83..115.83	
2 C	157.52	157.52..157.52	
3 N	114.19	114.19..114.19	
H			
4 C	123.33	123.33..123.33	
5 C	132.87	132.87..132.87	
6 C	95.28	95.28..95.28	6
H	6.80	6.80..6.80	6
7 C	159.70, 161.30	159.70..159.70, 161.30..161.30	
8 C	106.49	106.49..106.49	8
H	6.89	6.89..6.90	8
9 C	127.59	127.59..127.59	
10 C	27.24	27.24..27.25	10
H3	3.34	3.34..3.35	10
11 O			
12 F	-122.23	-122.24..-122.23	
13 C	40.07	40.06..40.07	13
H	3.31	3.31..3.31	13
14 C	48.44	48.44..48.45	14
14 H	3.22	3.22..3.22	14
15 C	47.19	47.19..47.20	15', 15"
H'	2.50	2.50..2.51	15
H"	3.26	3.25..3.26	15
16 N			
H			
17 C	47.14	47.14..47.15	17', 17"
H'	3.23	3.23..3.24	17
H"	3.35	3.35..3.35	17
18 C	124.23	124.23..124.23	
19 C	130.71	130.71..130.71	
20 C	39.16	39.16..39.16	20', 20"
H'	3.27	3.27..3.27	20
H"	2.92	2.91..2.92	20
21 C	26.70	26.70..26.70	21', 21"
H'	2.21	2.20..2.21	21
H"	2.02	2.01..2.02	21
22 C	31.91	31.91..31.91	22', 22"
H'	1.63	1.63..1.63	22
H"	1.68	1.68..1.68	22
23 C	62.45	62.45..62.46	23"
H'			
H"	3.57	3.57..3.57	23
24 O			
H			

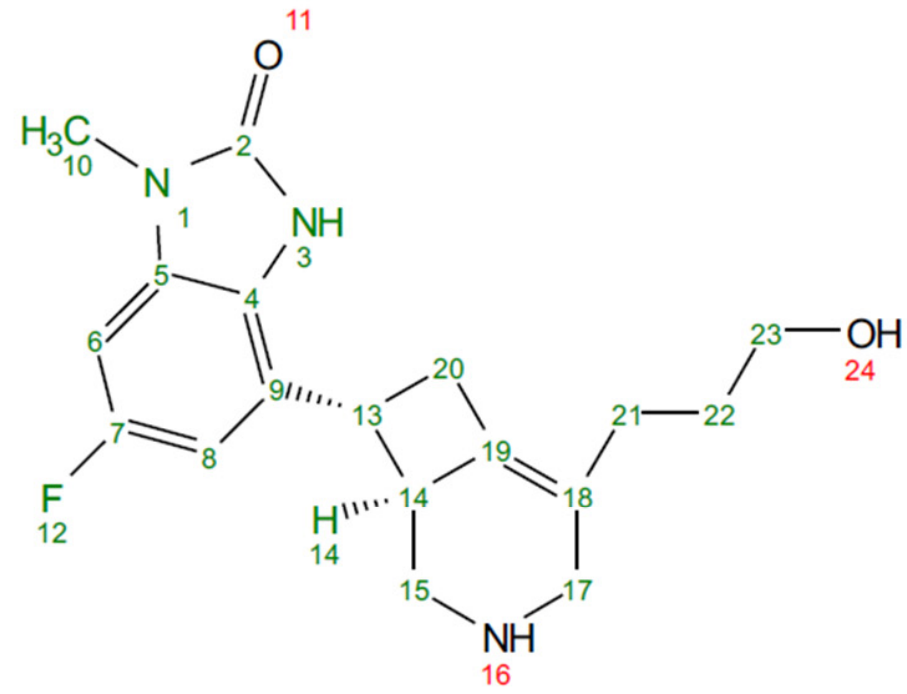

pre-B67b

HSQC

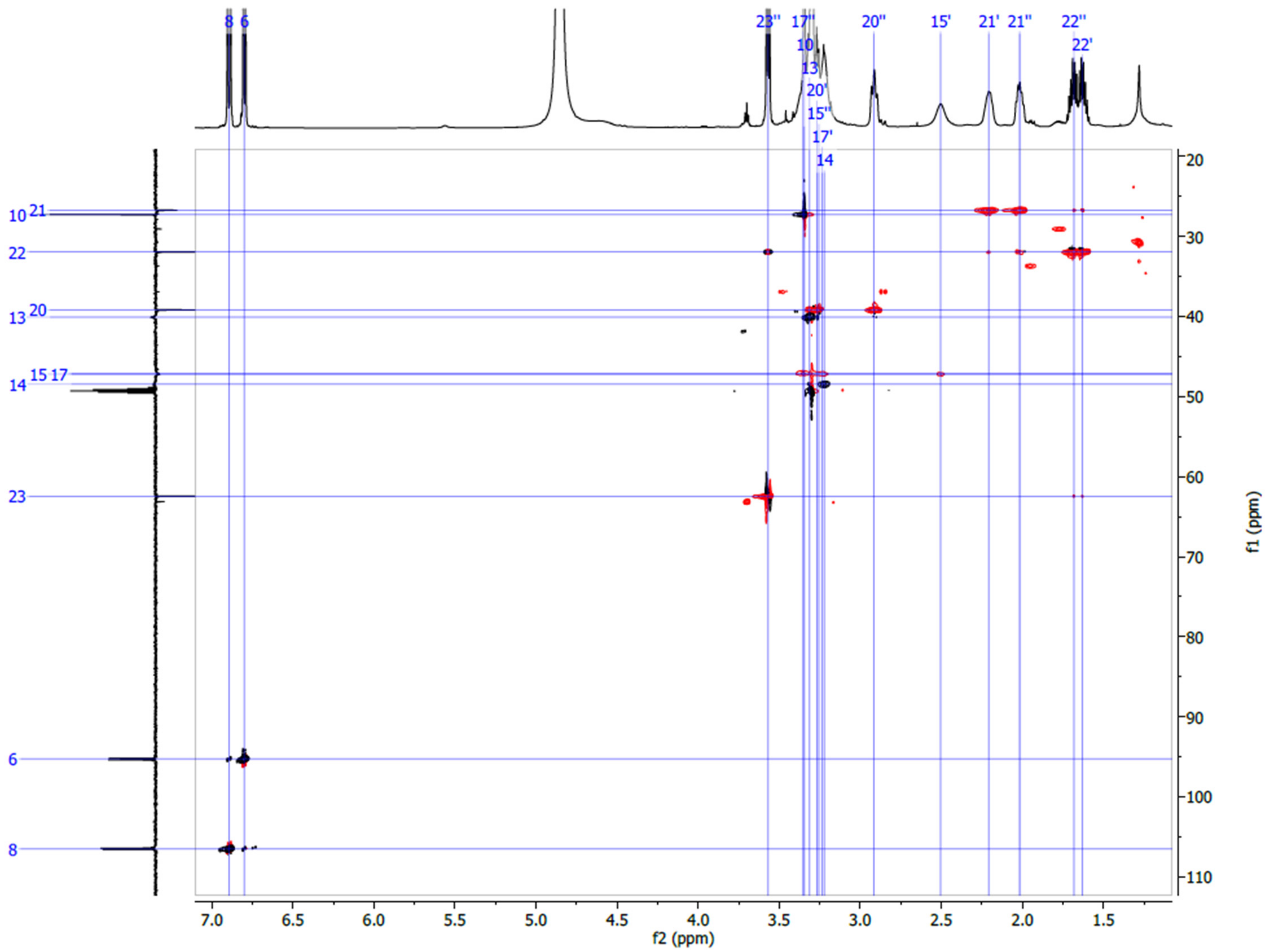

HMBC

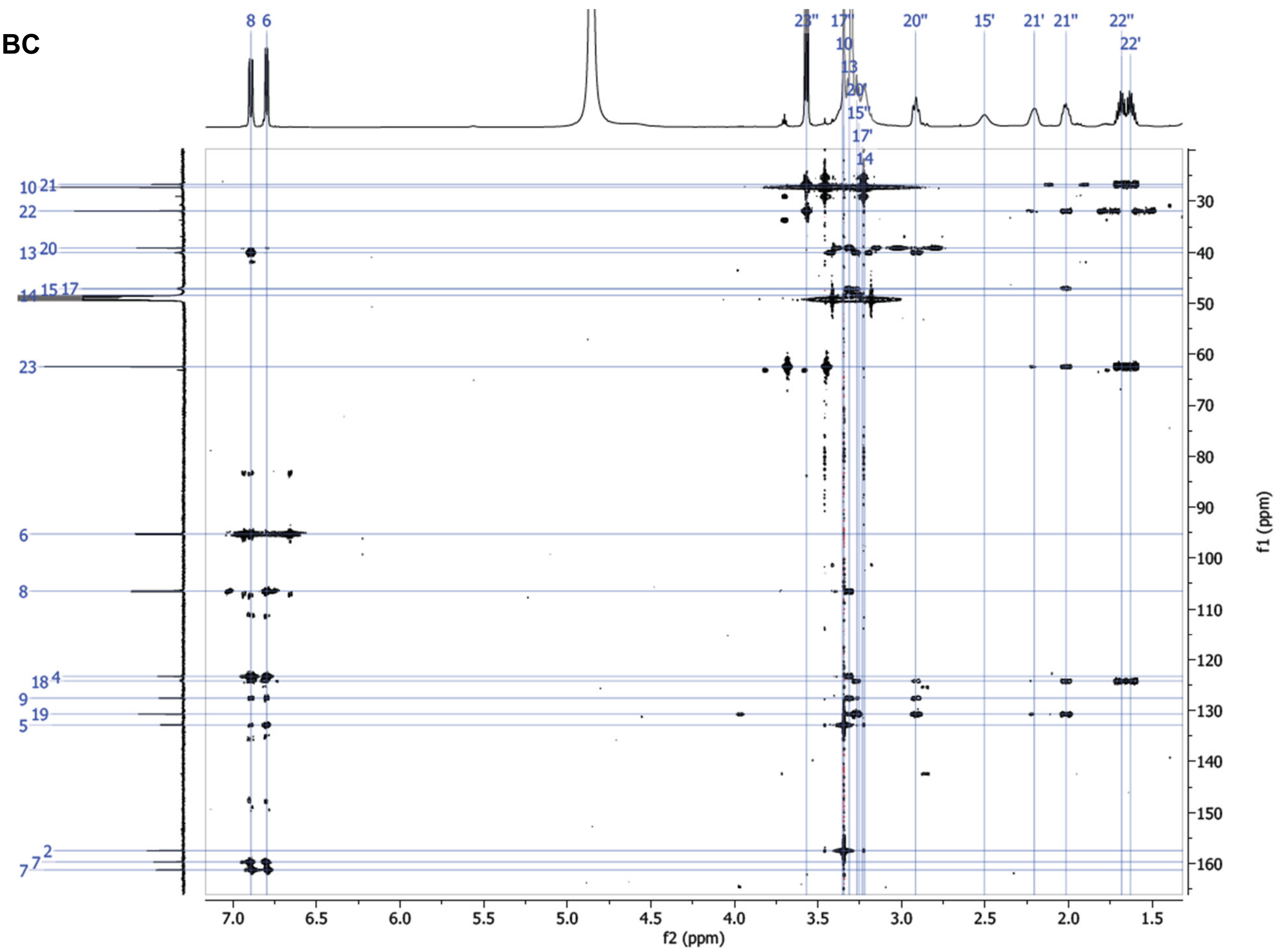

COSY

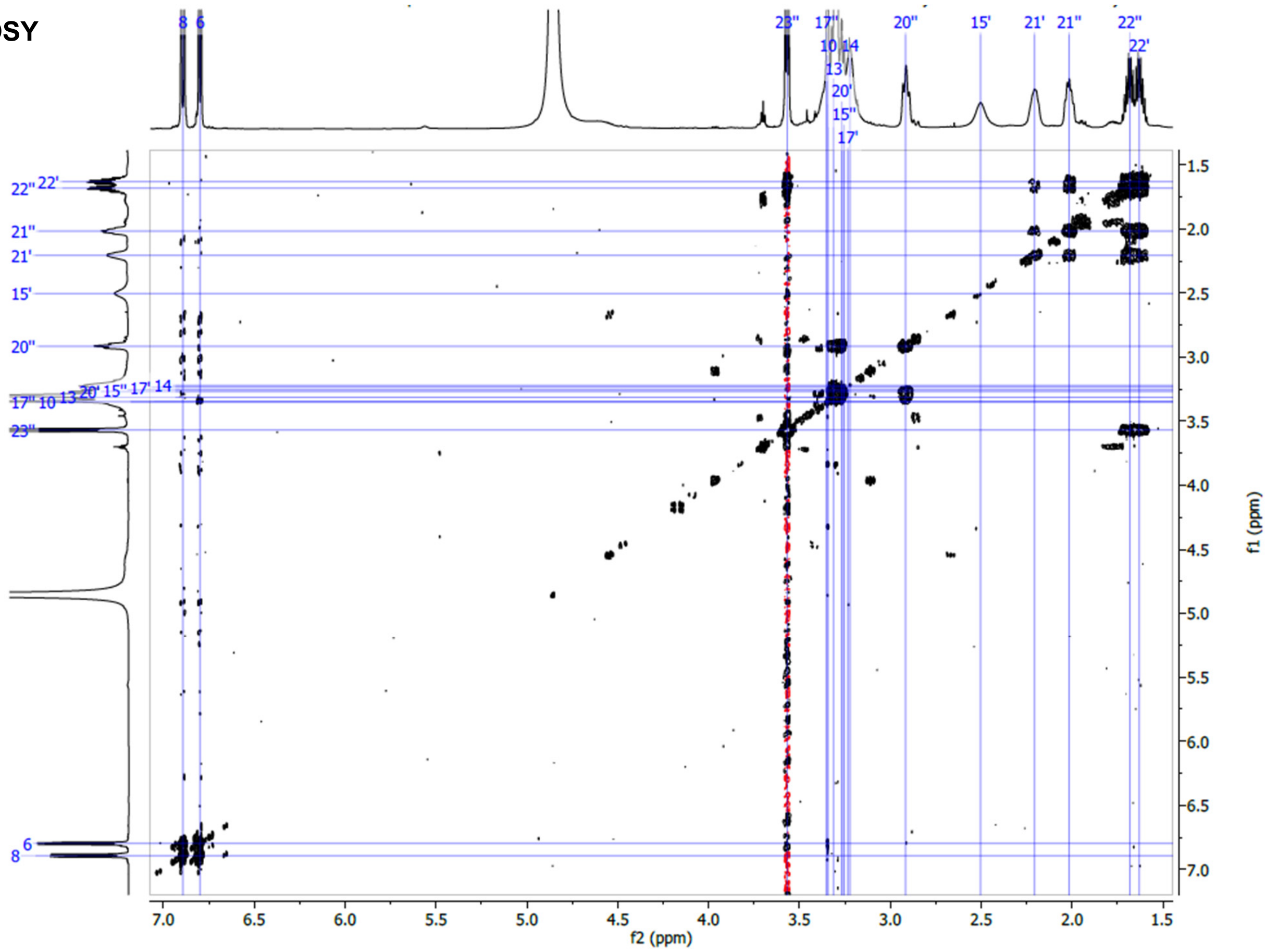

NOESY

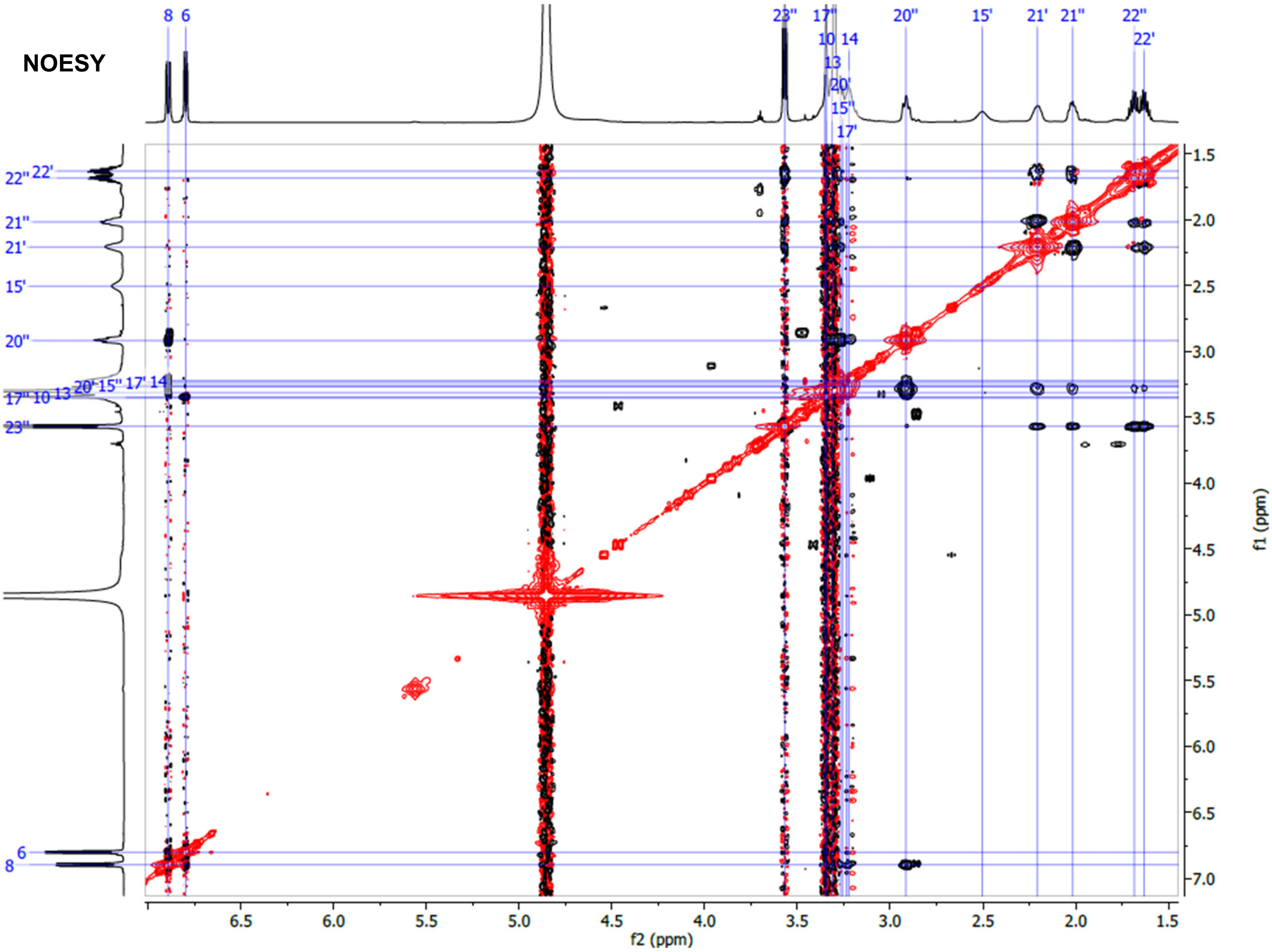

NOESY (expanded)

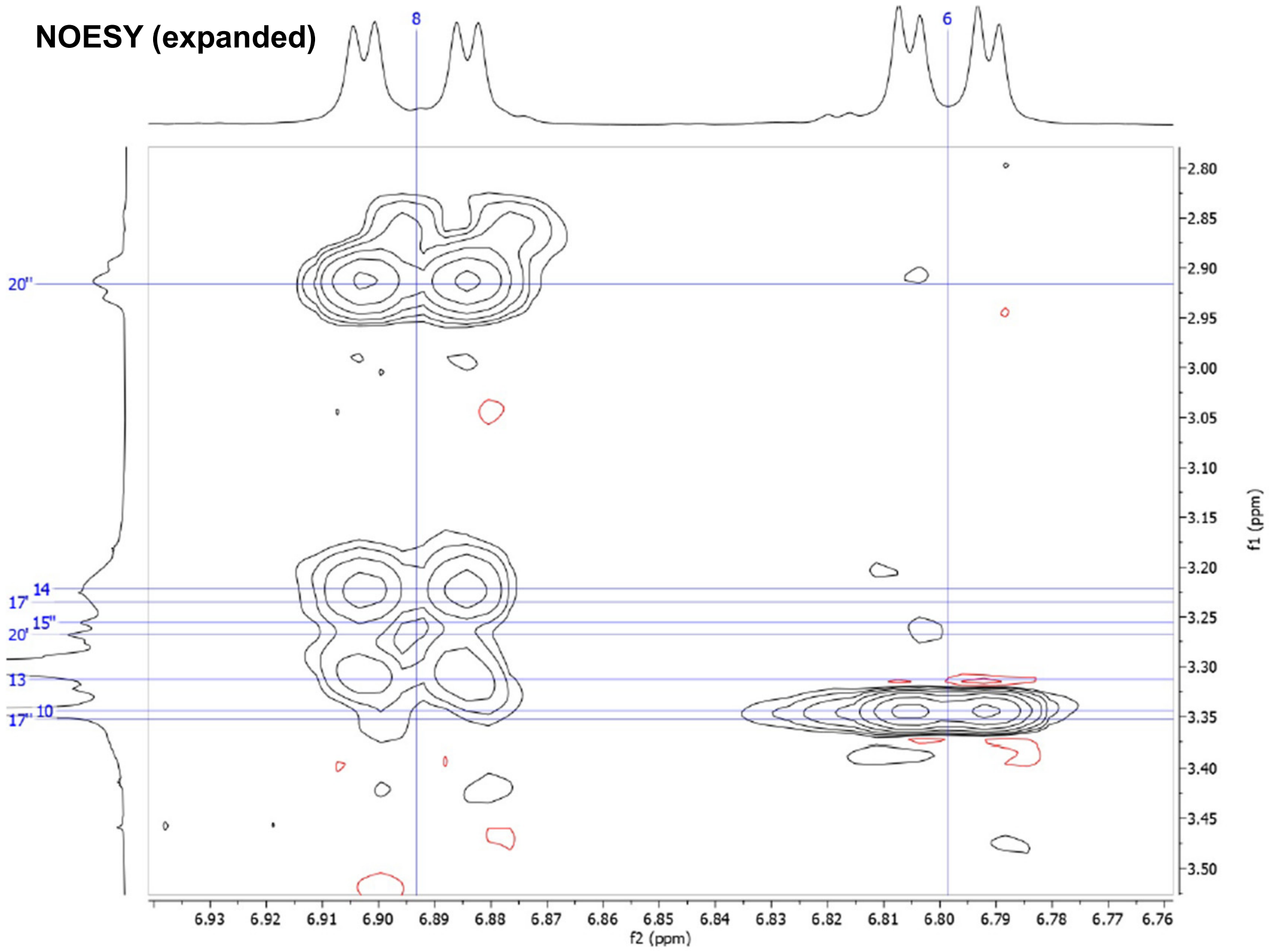

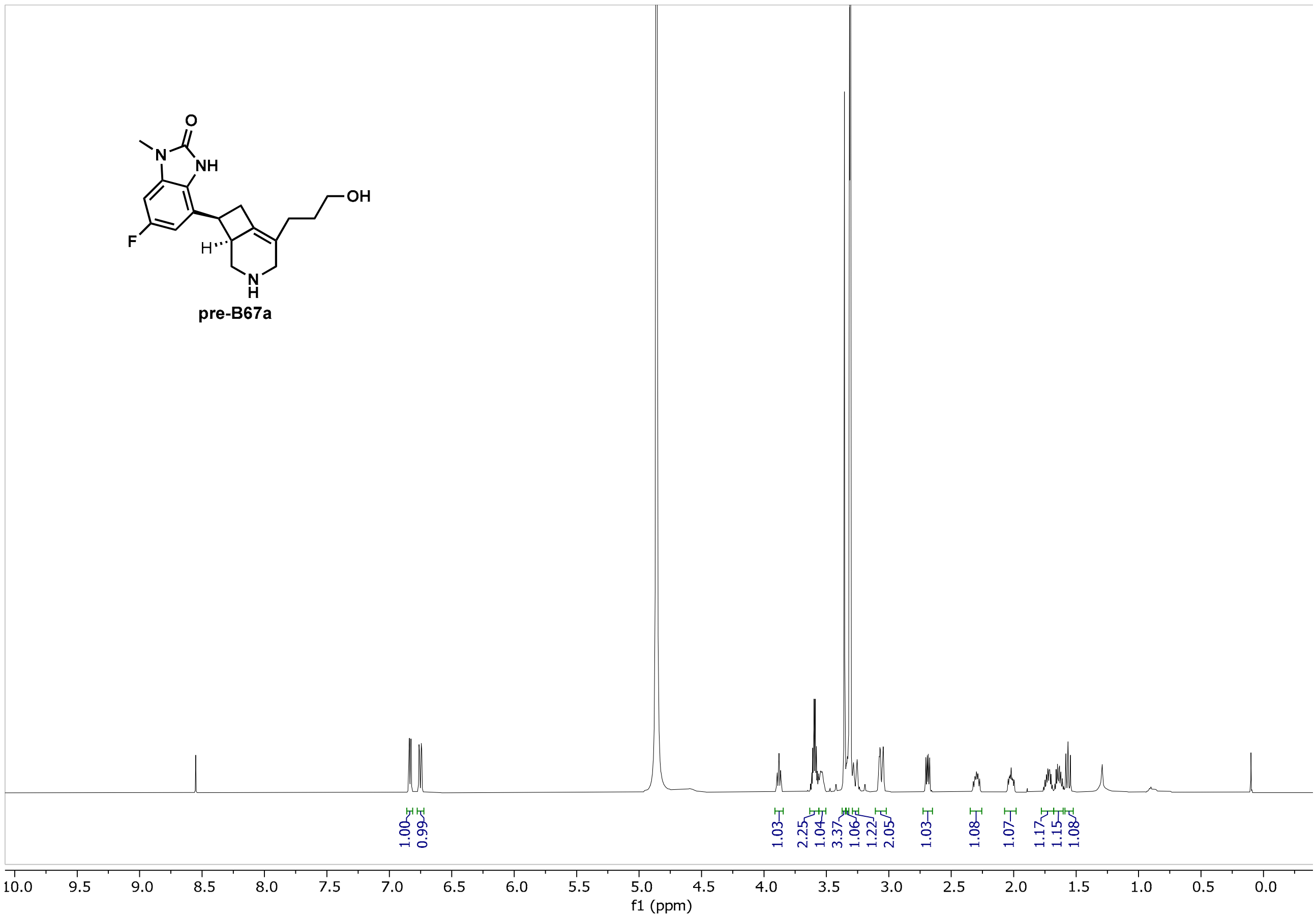

pre-B67a
1.00
0.99
1.03
2.25
1.04
3.37
1.06
1.22
2.05
1.03
1.08
1.07
1.17
1.15
1.08
10.0 9.5 9.0 8.5 8.0 7.5 7.0 6.5 6.0 5.5 5.0 4.5 4.0 3.5 3.0 2.5 2.0 1.5 1.0 0.5 0.0
f1 (ppm)

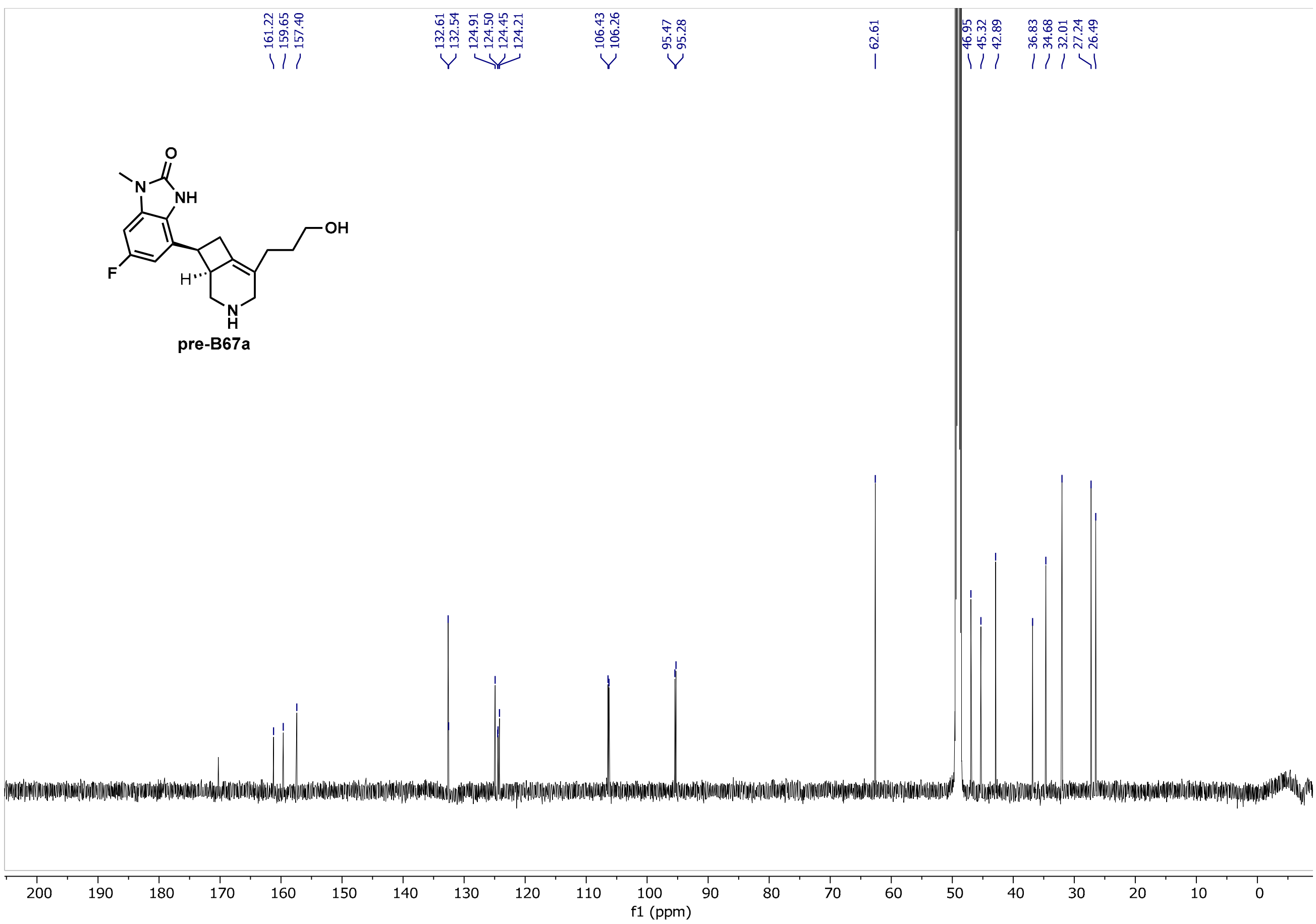

161.22
159.65
157.40
132.61
132.54
124.91
124.50
124.45
124.21
106.43
106.26
95.47
95.28
62.61
46.95
45.32
42.89
36.83
34.68
32.01
27.24
26.49
pre-B67a
200 190 180 170 160 150 140 130 120 110 100 90 80 70 60 50 40 30 20 10 0
f1 (ppm)

Atom	δ (ppm)	Min..Max (ppm)	HSQC	Atom	δ (ppm)	Min..Max (ppm)	HSQC
1 N	116.29	116.29..116.29		16 N	27.25	27.25..27.25	
2 C	157.39	157.38..157.39		H			
3 N	115.24	115.24..115.24		17 C	46.95	46.95..46.96	17', 17"
H				H'	3.25	3.25..3.26	17
4 C	124.22	124.22..124.22		H"	3.05	3.05..3.05	17
5 C	132.54	132.54..132.54		18 C	124.91	124.91..124.91	
6 C	95.37	95.37..95.38	6	19 C	132.61	132.61..132.62	
H	6.82	6.82..6.83	6	20 C	34.69	34.69..34.69	20', 20"
7 C	159.66, 161.22	159.66..159.66, 161.22..161.22		H'	3.05	3.05..3.05	20
8 C	106.32	106.32..106.32	8	H"	3.33	3.33..3.33	20
H	6.74	6.74..6.74	8	21 C	26.49	26.49..26.50	21', 21"
9 C	124.47	124.47..124.47		H'	2.28	2.28..2.29	21
10 C	27.24	27.24..27.24	10	H"	2.01	2.01..2.01	21
H3	3.35	3.34..3.35	10	22 C	32.03	32.02..32.03	22', 22"
11 O				H'	1.63	1.63..1.63	22
12 F	-122.46	-122.47..-122.45		H"	1.71	1.71..1.71	22
13 C	36.83	36.82..36.83	13	23 C	62.60	62.60..62.60	23"
H	3.87	3.87..3.87	13	H'			
14 C	45.32	45.32..45.33	14	H"	3.58	3.58..3.58	23
14 H	3.53	3.53..3.53	14	24 O			
15 C	42.88	42.88..42.89	15', 15"	H			
H'	2.68	2.67..2.68	15				
H"	1.55	1.55..1.56	15				

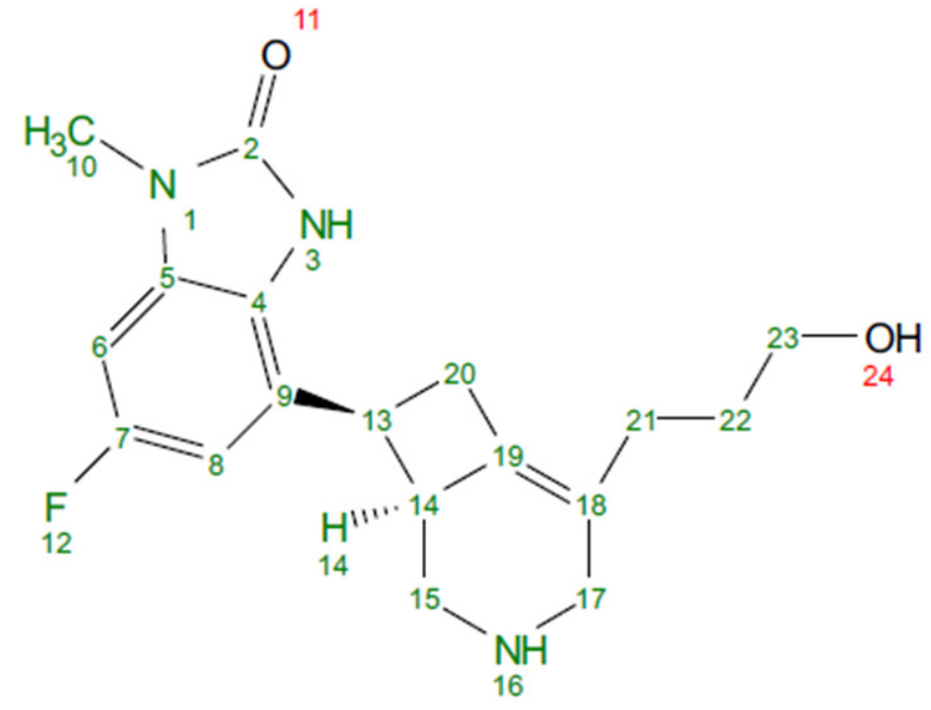

pre-B67a

HSQC

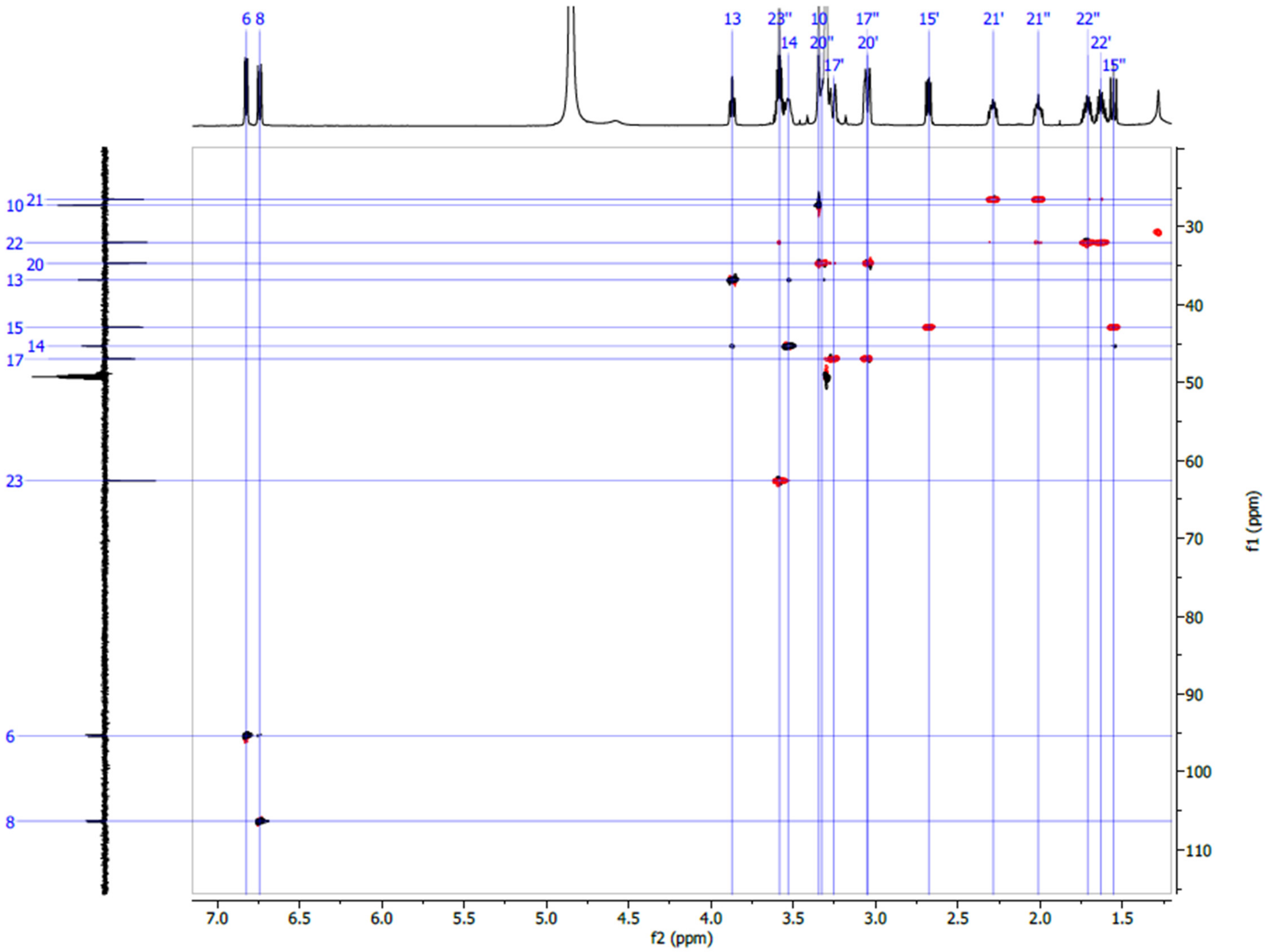

HMBC

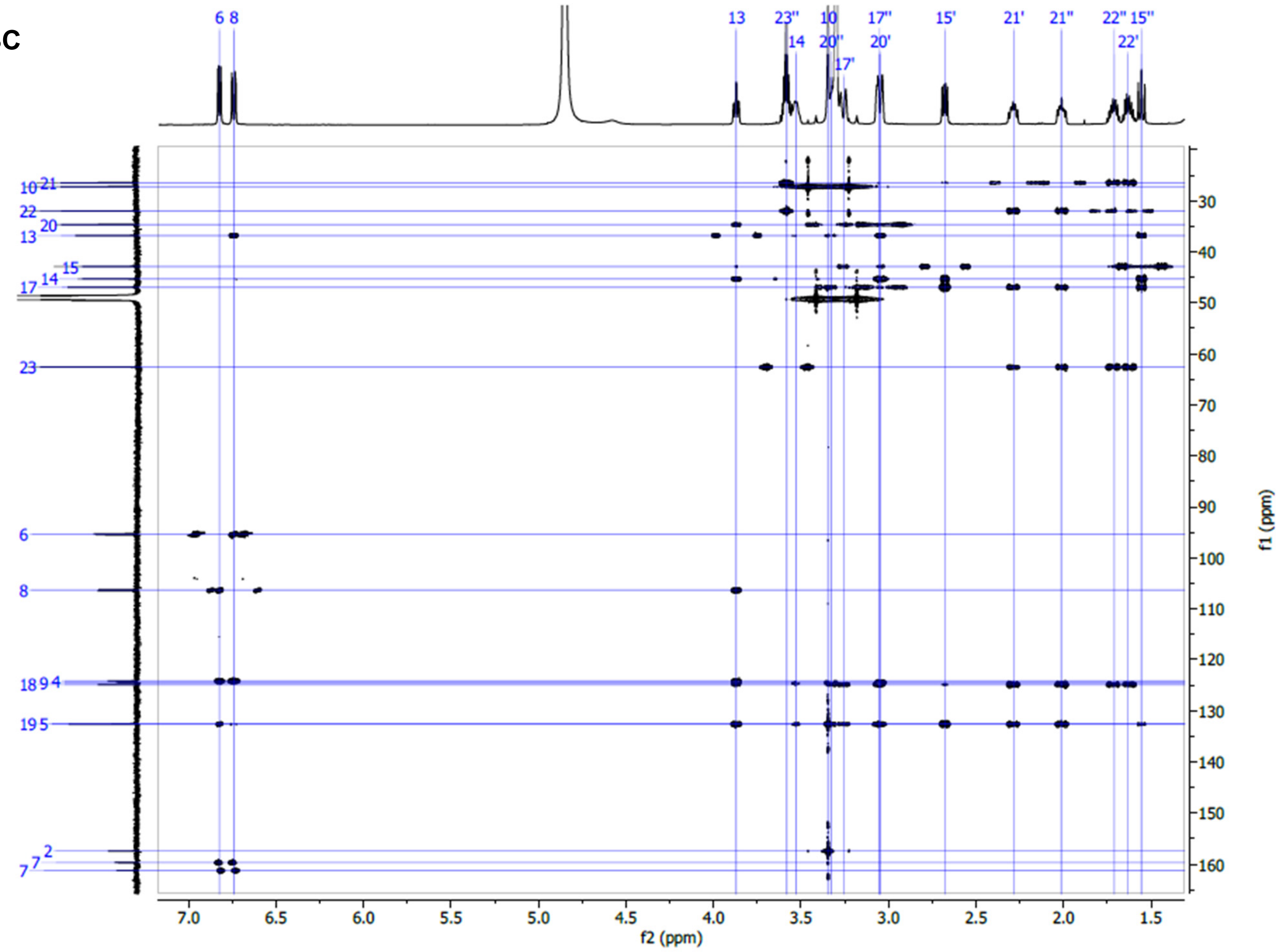
6 8
13
23''
14
10
20''
17'
17''
20'
15'
21'
21''
22''
15''
22'
10 21
22
20
13
15
14
17
23
6
8
18 9 4
19 5
2
7
7
f2 (ppm)
f1 (ppm)

COSY

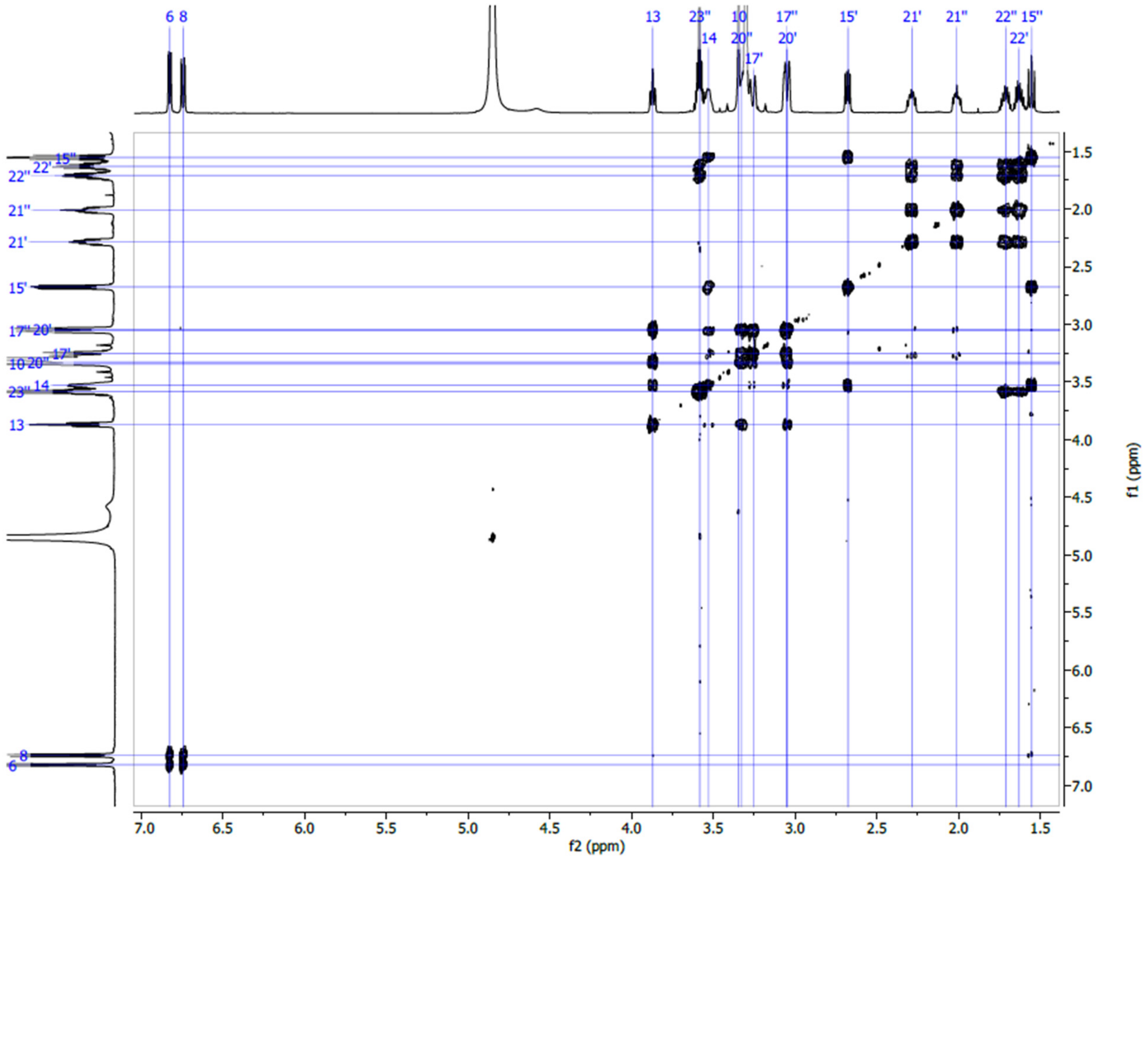
6 8
13
23''
14
10
20''
17'
17''
20'
15'
21'
21''
22''
15''
22'
15''
22'
22''
21''
21'
15'
17'' 20'
17'
10 20''
23'' 14
13
8
6
f2 (ppm)
f1 (ppm)

NOESY

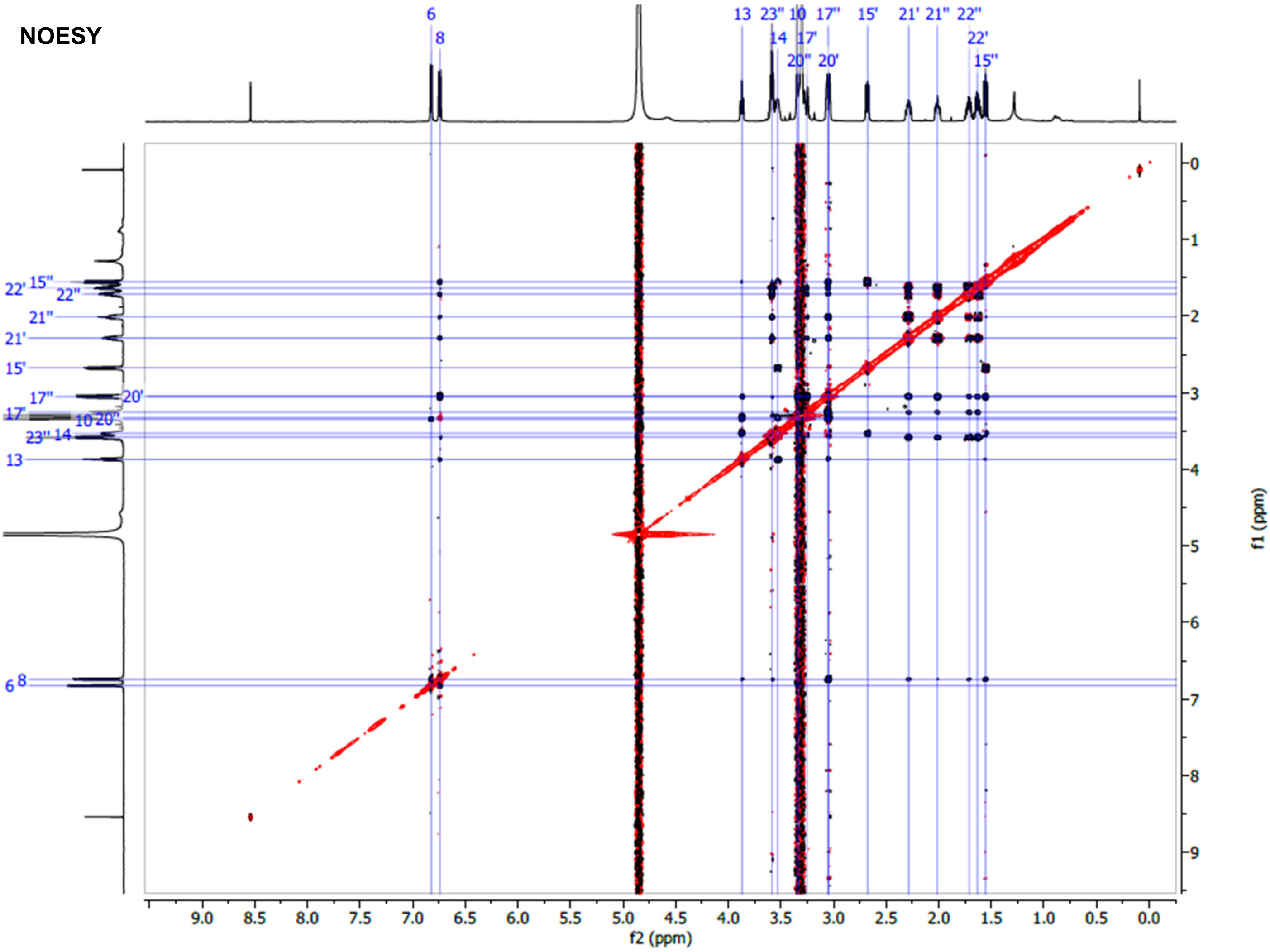

NOESY (expanded)

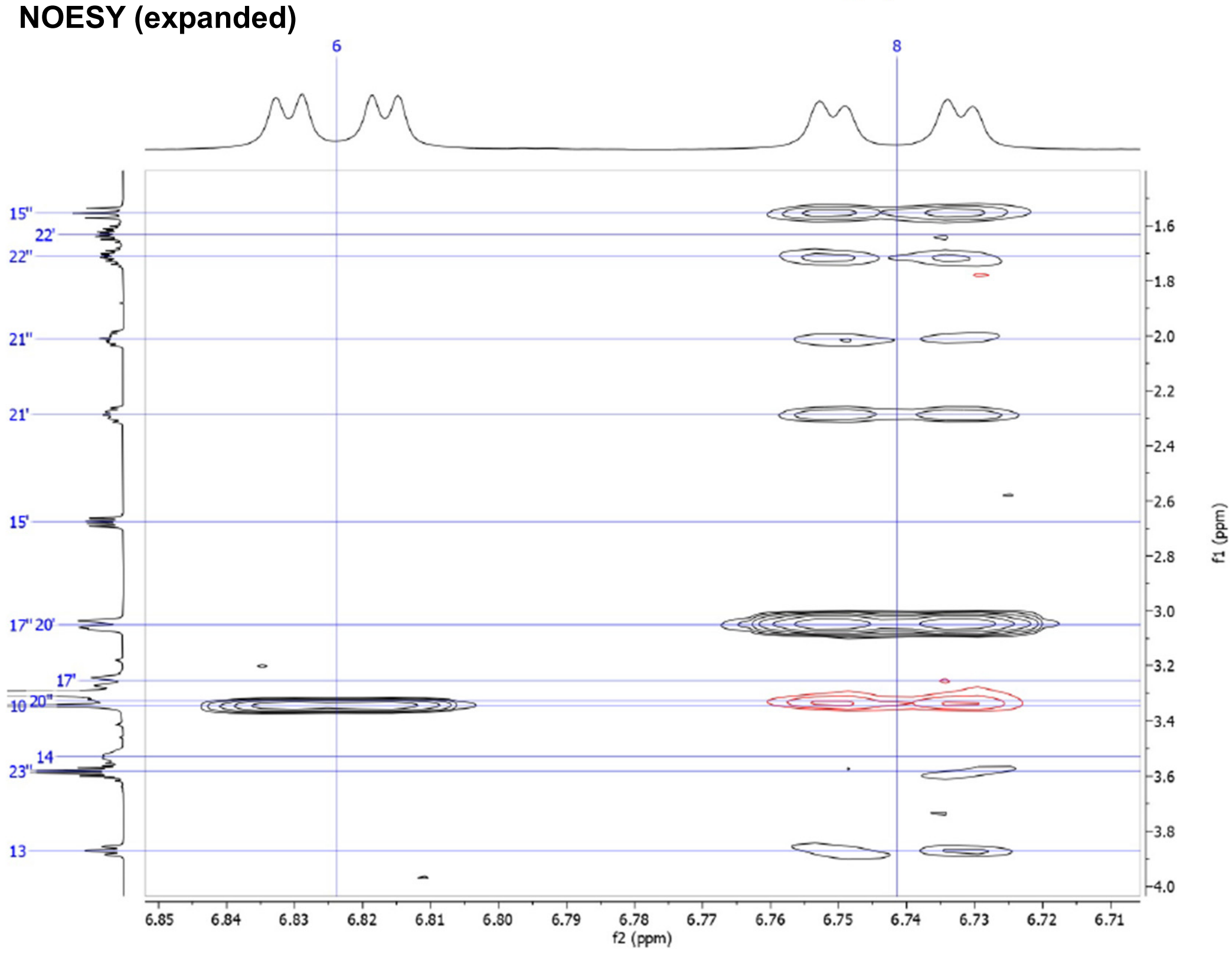

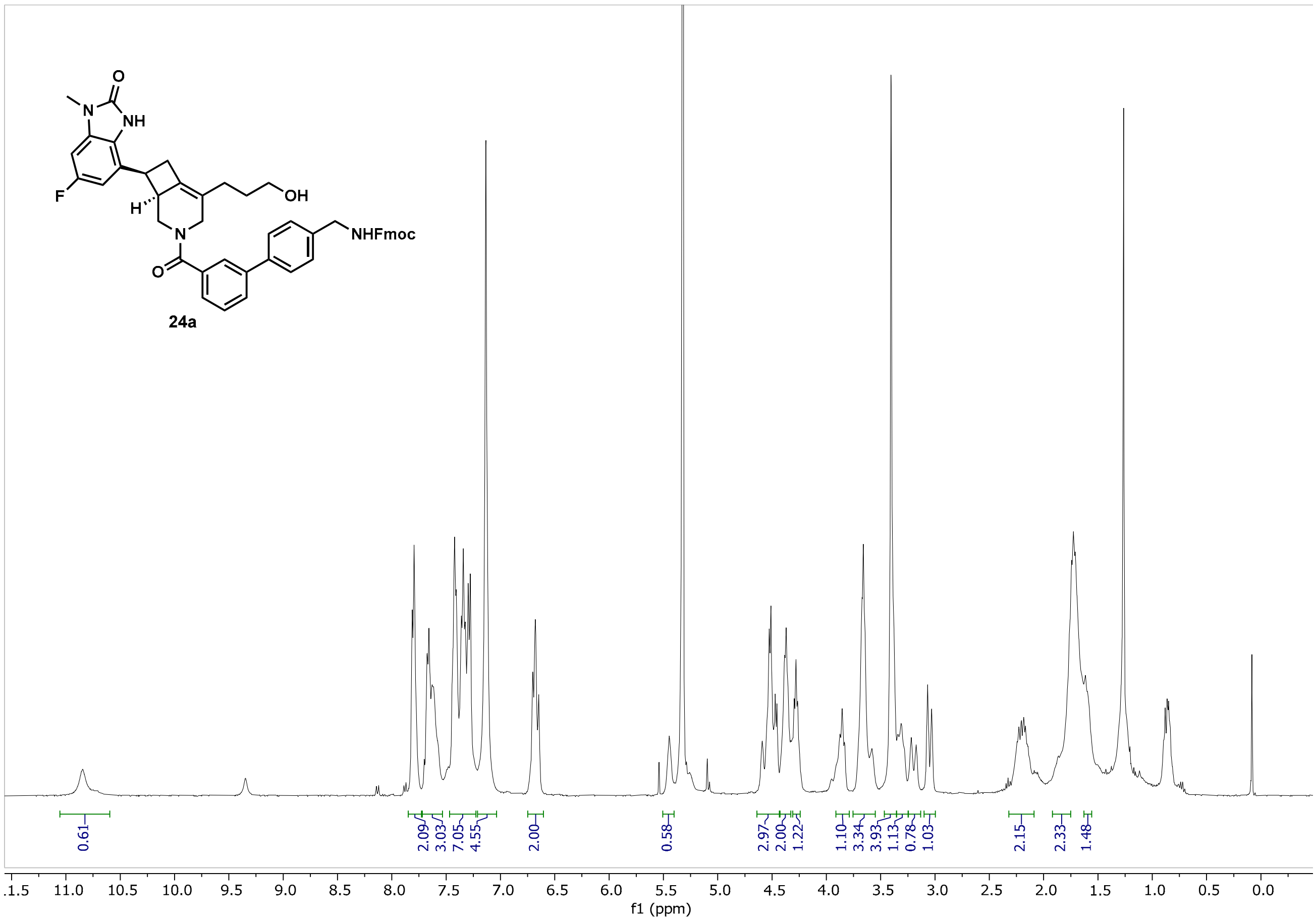
24a
0.61
2.09
3.03
7.05
4.55
2.00
0.58
2.97
2.00
1.22
1.10
3.34
3.93
1.13
0.78
1.03
2.15
2.33
1.48
f1 (ppm)

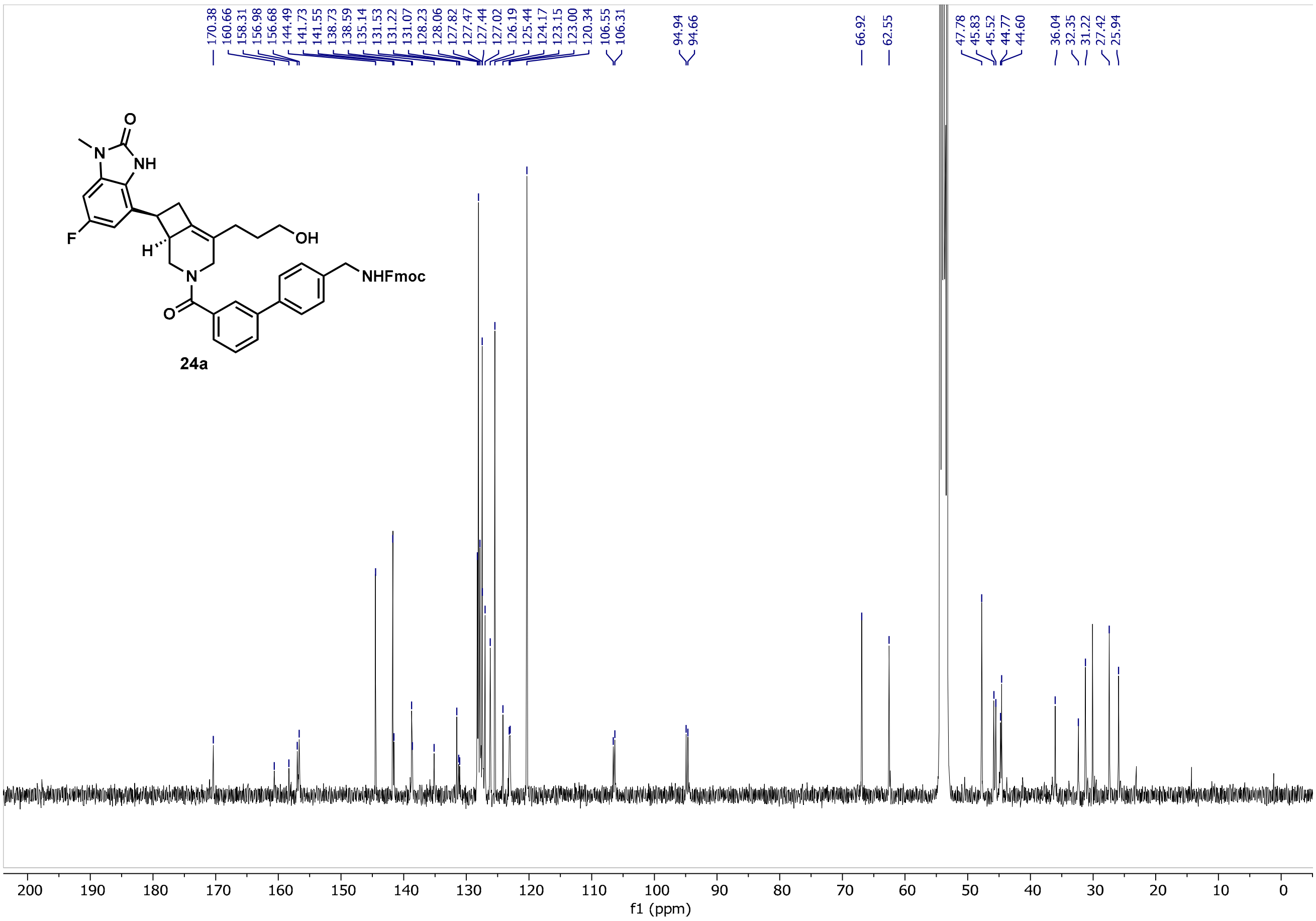
170.38
160.66
158.31
156.98
156.68
144.49
141.73
141.55
138.73
138.59
135.14
131.53
131.22
131.07
128.23
128.06
127.82
127.47
127.44
127.02
126.19
125.44
124.17
123.15
123.00
120.34
106.55
106.31
94.94
94.66
66.92
62.55
47.78
45.83
45.52
44.77
44.60
36.04
32.35
31.22
27.42
25.94
24a
f1 (ppm)

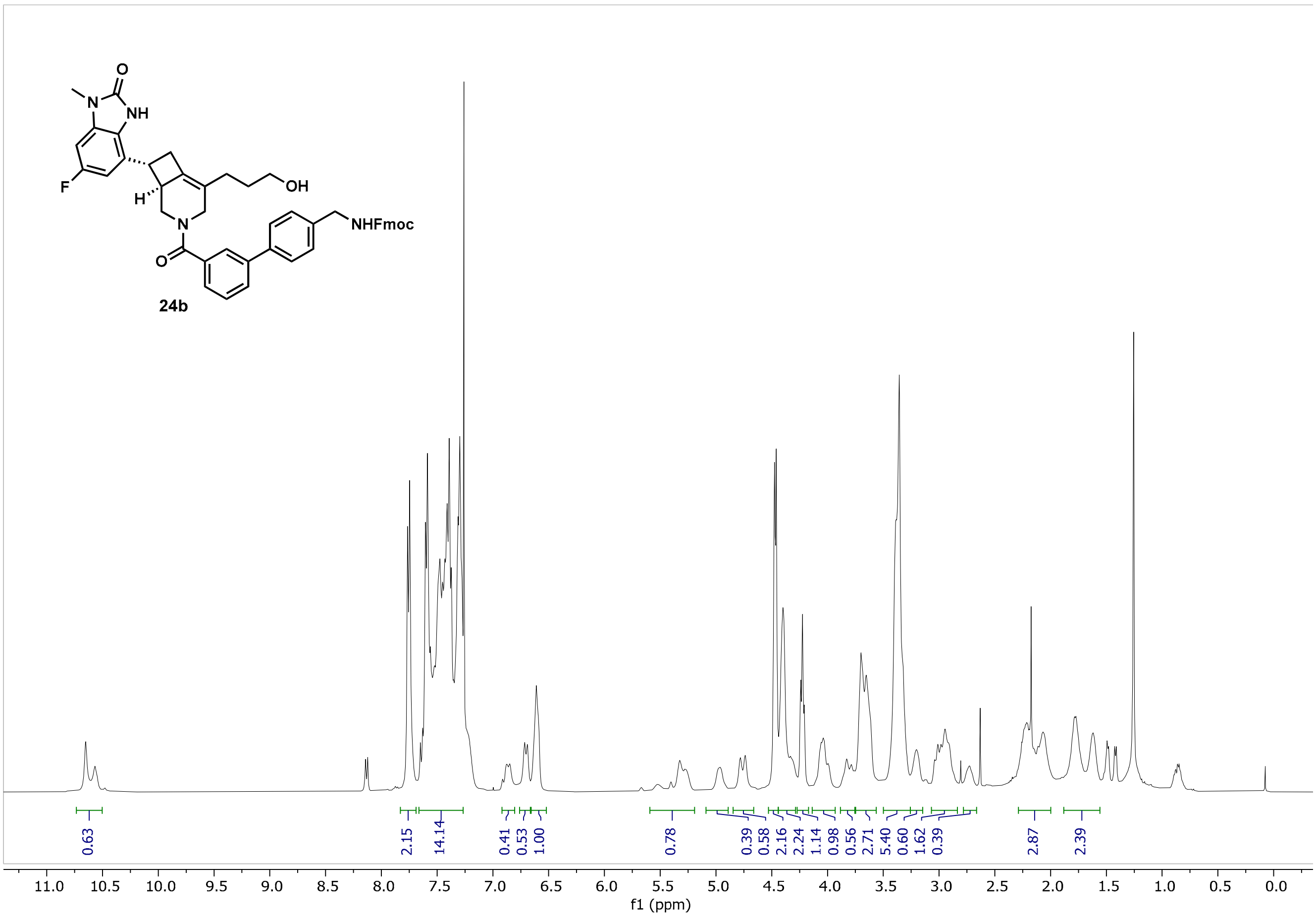

O
N
NH
F
H
OH
N
NHFmoc
O
24b
0.63
2.15
14.14
0.41
0.53
1.00
0.78
0.39
0.58
2.16
2.24
1.14
0.98
0.56
2.71
5.40
0.60
1.62
0.39
2.87
2.39
11.0 10.5 10.0 9.5 9.0 8.5 8.0 7.5 7.0 6.5 6.0 5.5 5.0 4.5 4.0 3.5 3.0 2.5 2.0 1.5 1.0 0.5 0.0
f1 (ppm)

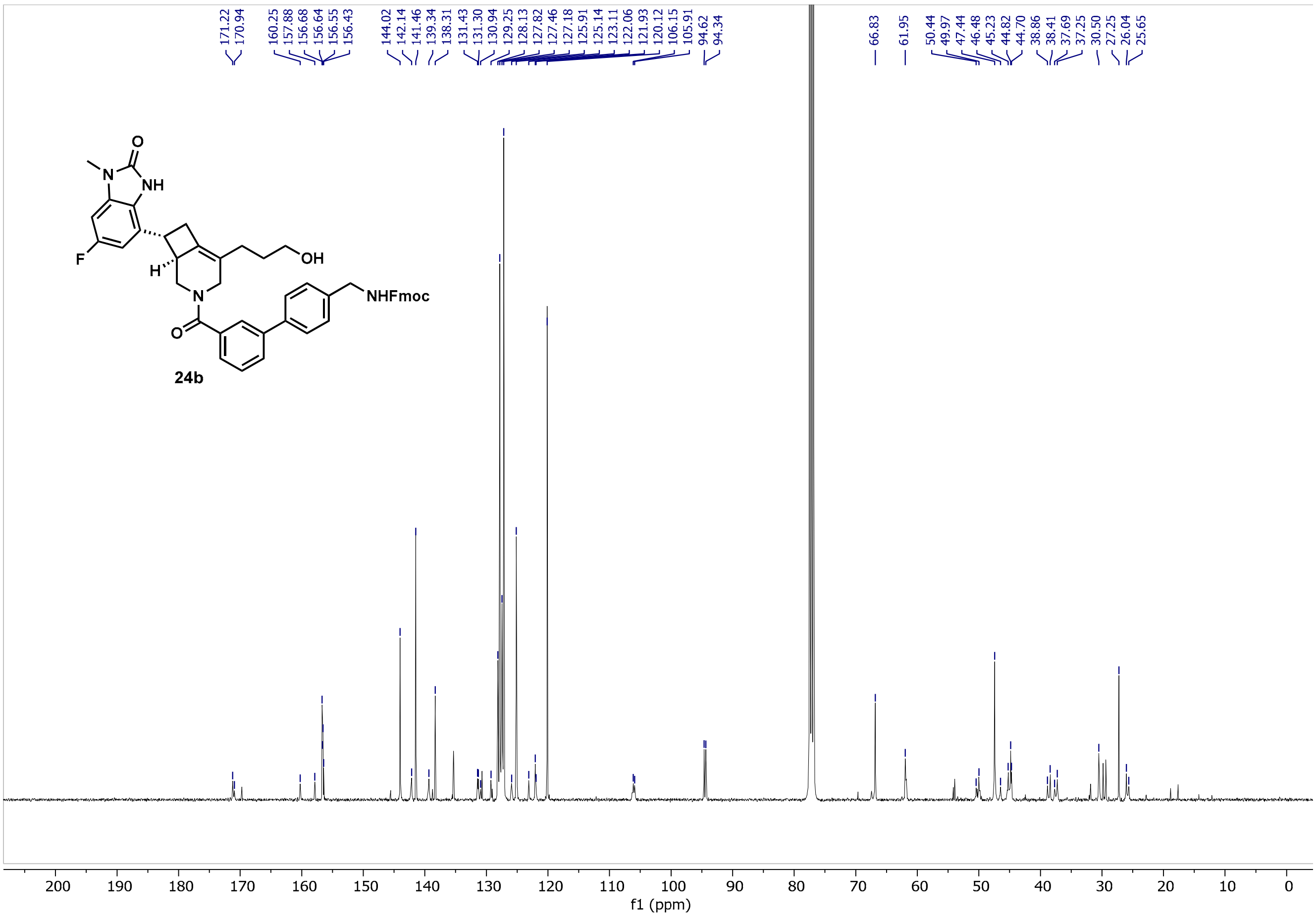

171.22
170.94
160.25
157.88
156.68
156.64
156.55
156.43
144.02
142.14
141.46
139.34
138.31
131.43
131.30
130.94
129.25
128.13
127.82
127.46
127.18
125.91
125.14
123.11
122.06
121.93
120.12
106.15
105.91
94.62
94.34
66.83
61.95
50.44
49.97
47.44
46.48
45.23
44.82
44.70
38.86
38.41
37.69
37.25
30.50
27.25
26.04
25.65
O
N
NH
F
H
OH
N
NHFmoc
O
24b
200 190 180 170 160 150 140 130 120 110 100 90 80 70 60 50 40 30 20 10 0
f1 (ppm)

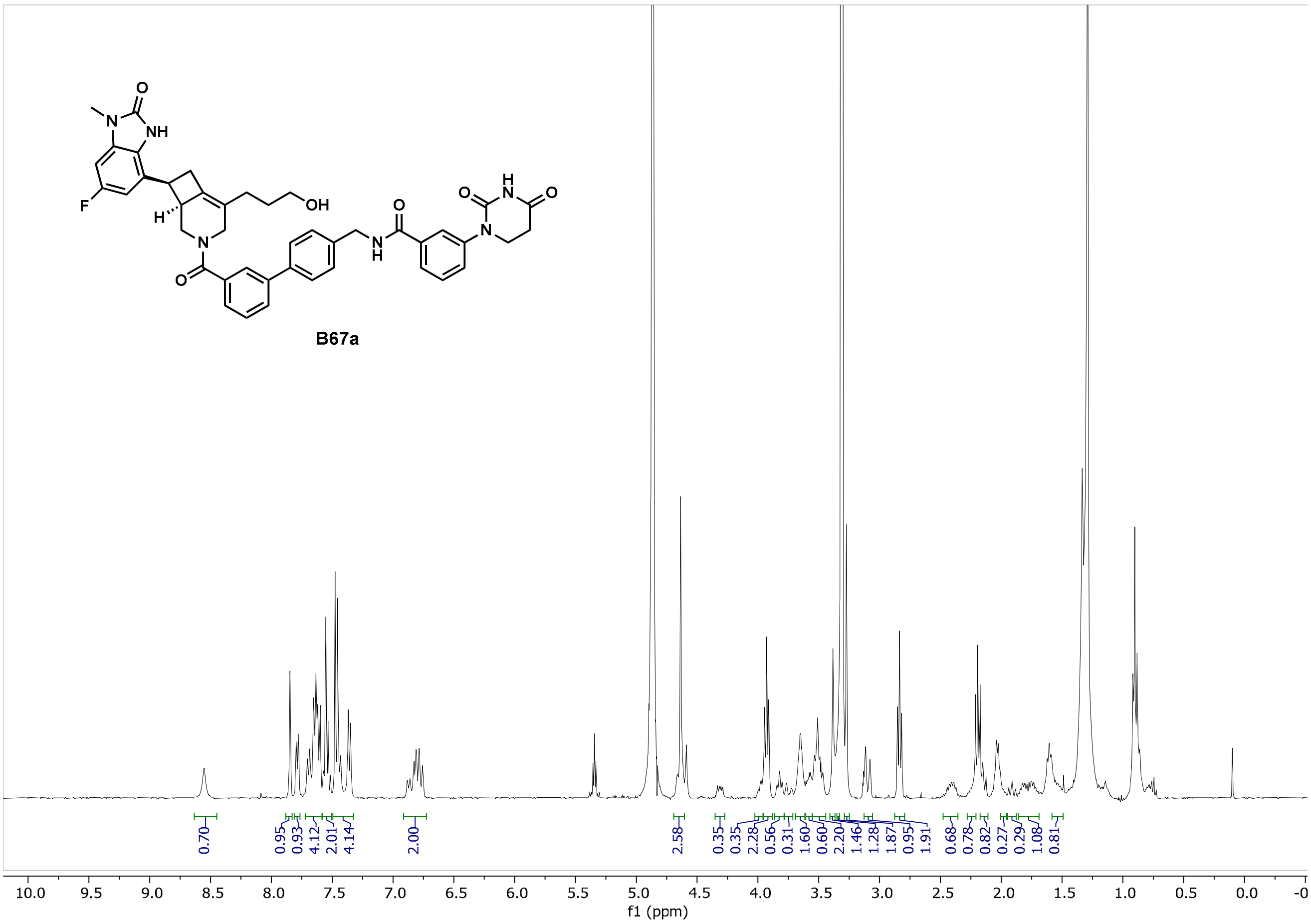
B67a
0.70
0.95
0.93
4.12
2.01
4.14
2.00
2.58
0.35
0.35
2.28
0.56
0.31
1.60
0.60
2.20
1.46
1.28
1.87
0.95
1.91
0.68
0.78
0.82
0.27
0.29
1.08
0.81
f1 (ppm)

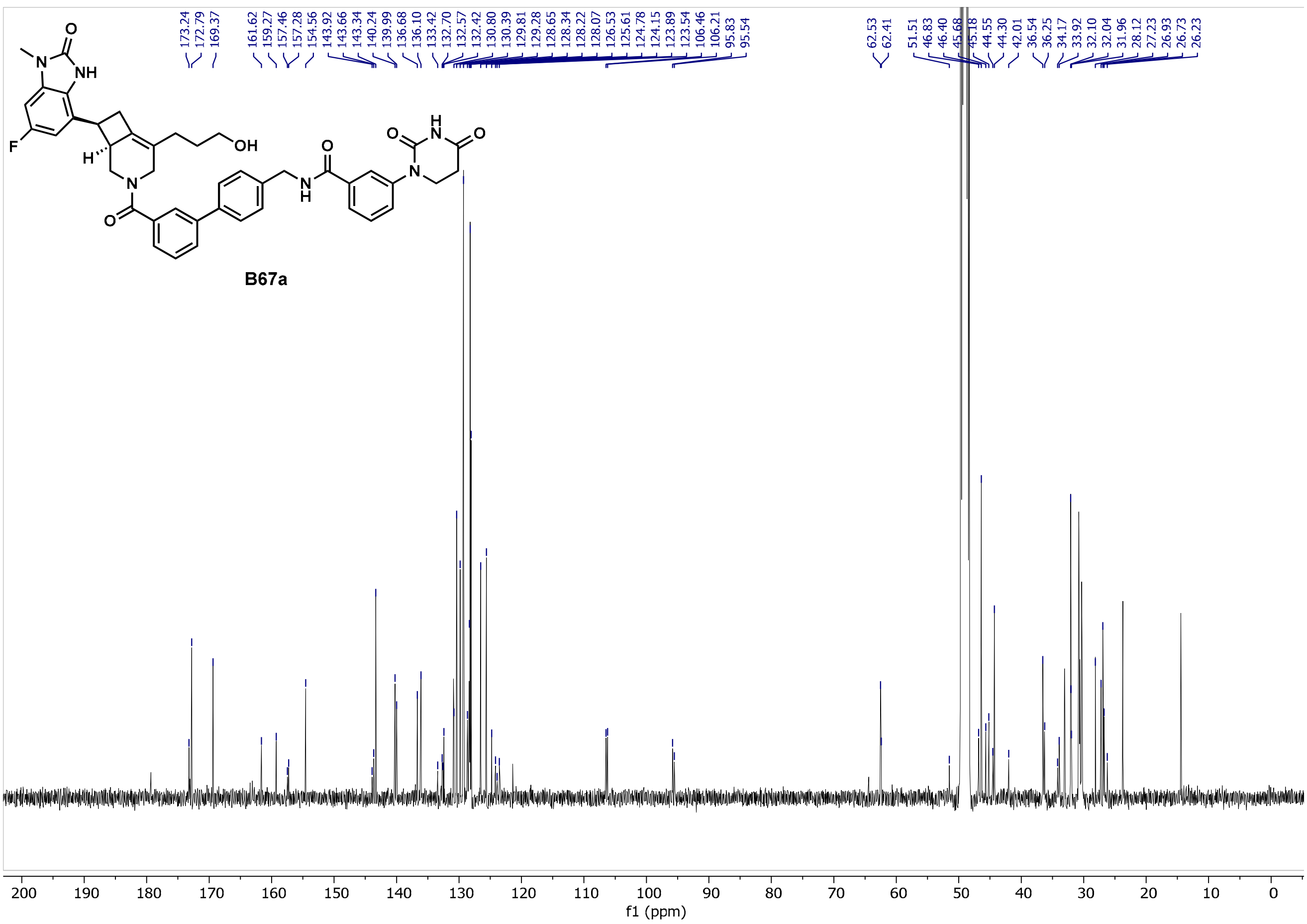
173.24
172.79
169.37
161.62
159.27
157.46
157.28
154.56
143.92
143.66
143.34
140.24
139.99
136.68
136.10
133.42
132.70
132.57
132.42
130.80
130.39
129.81
129.28
128.65
128.34
128.22
128.07
126.53
125.61
124.78
124.15
123.89
123.54
106.46
106.21
95.83
95.54
62.53
62.41
51.51
46.83
46.40
45.68
45.18
44.55
44.30
42.01
36.54
36.25
34.17
33.92
32.10
32.04
31.96
28.12
27.23
26.93
26.73
26.23
B67a
f1 (ppm)

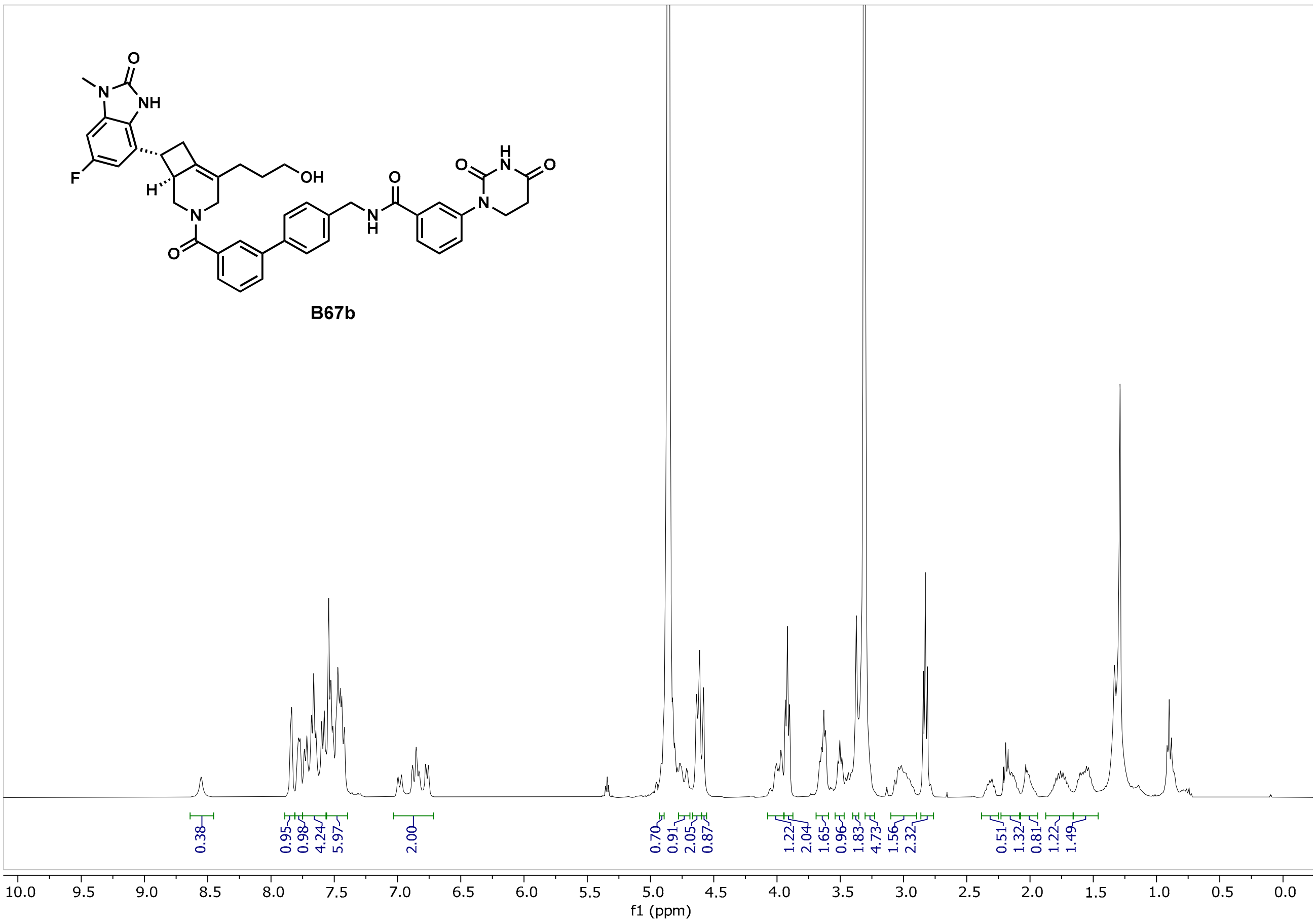

B67b
0.38
0.95
0.98
4.24
5.97
2.00
0.70
0.91
2.05
0.87
1.22
2.04
1.65
0.96
1.83
4.73
1.56
2.32
0.51
1.32
0.81
1.22
1.49
f1 (ppm)

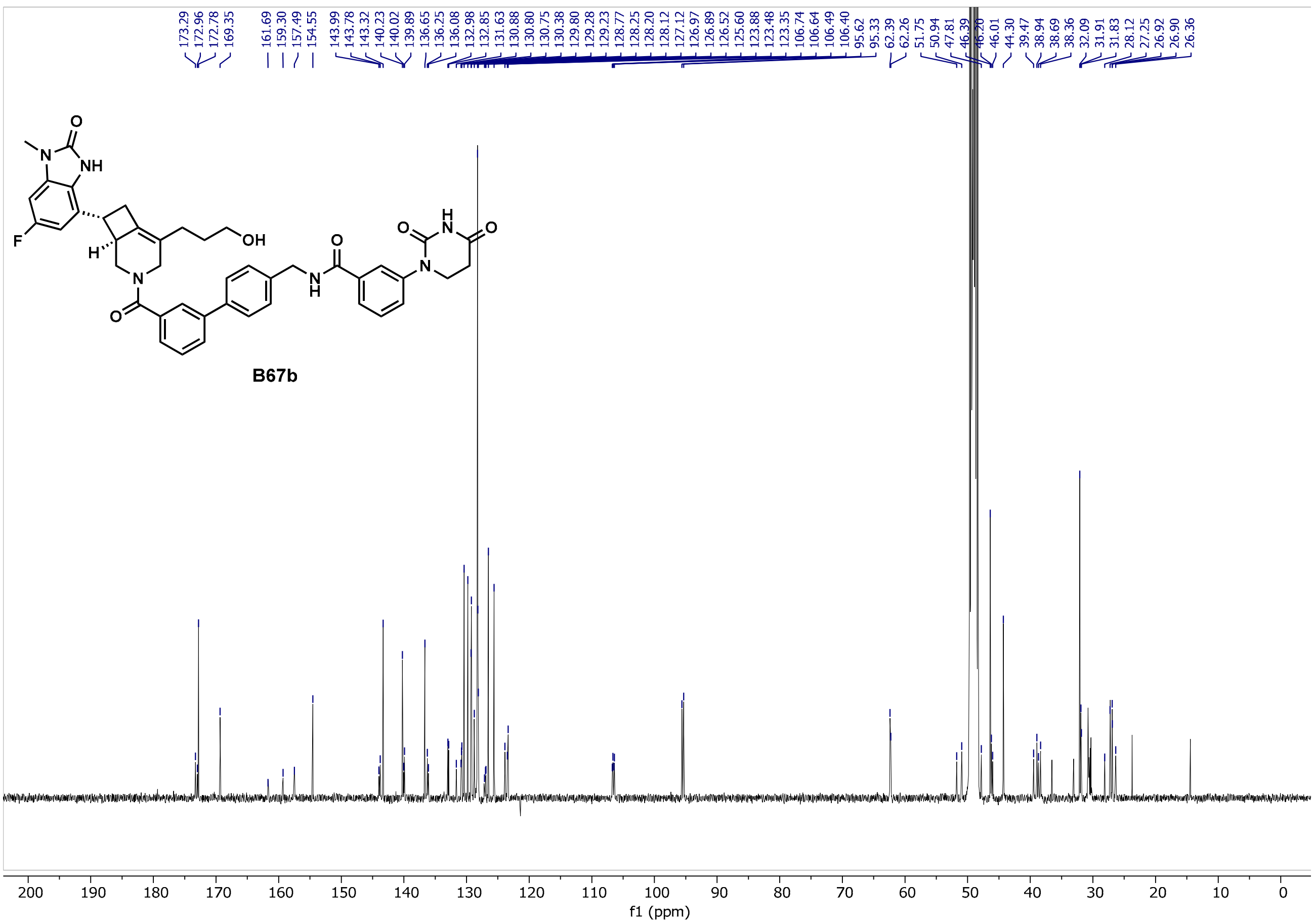

173.29
172.96
172.78
169.35
161.69
159.30
157.49
154.55
143.99
143.78
143.32
140.23
140.02
139.89
136.65
136.25
136.08
132.98
132.85
131.63
130.88
130.80
130.75
130.38
129.80
129.28
129.23
128.77
128.25
128.20
128.12
127.12
126.97
126.89
126.52
125.60
123.88
123.48
123.35
106.74
106.64
106.49
106.40
95.62
95.33
62.39
62.26
51.75
50.94
47.81
46.39
46.20
46.01
44.30
39.47
38.94
38.69
38.36
32.09
31.91
31.83
28.12
27.25
26.92
26.90
26.36
B67b
f1 (ppm)

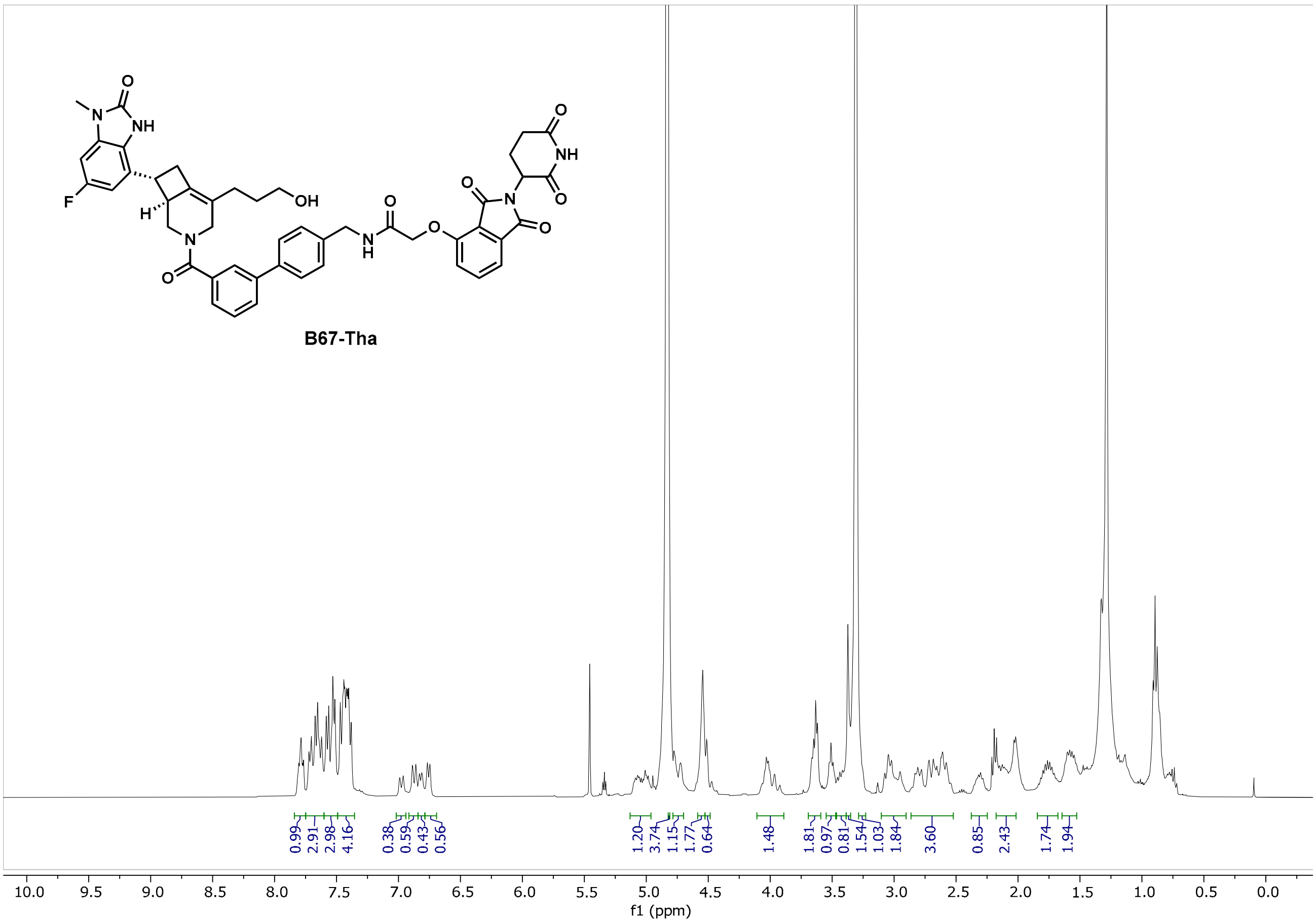
B67-Tha
f1 (ppm)

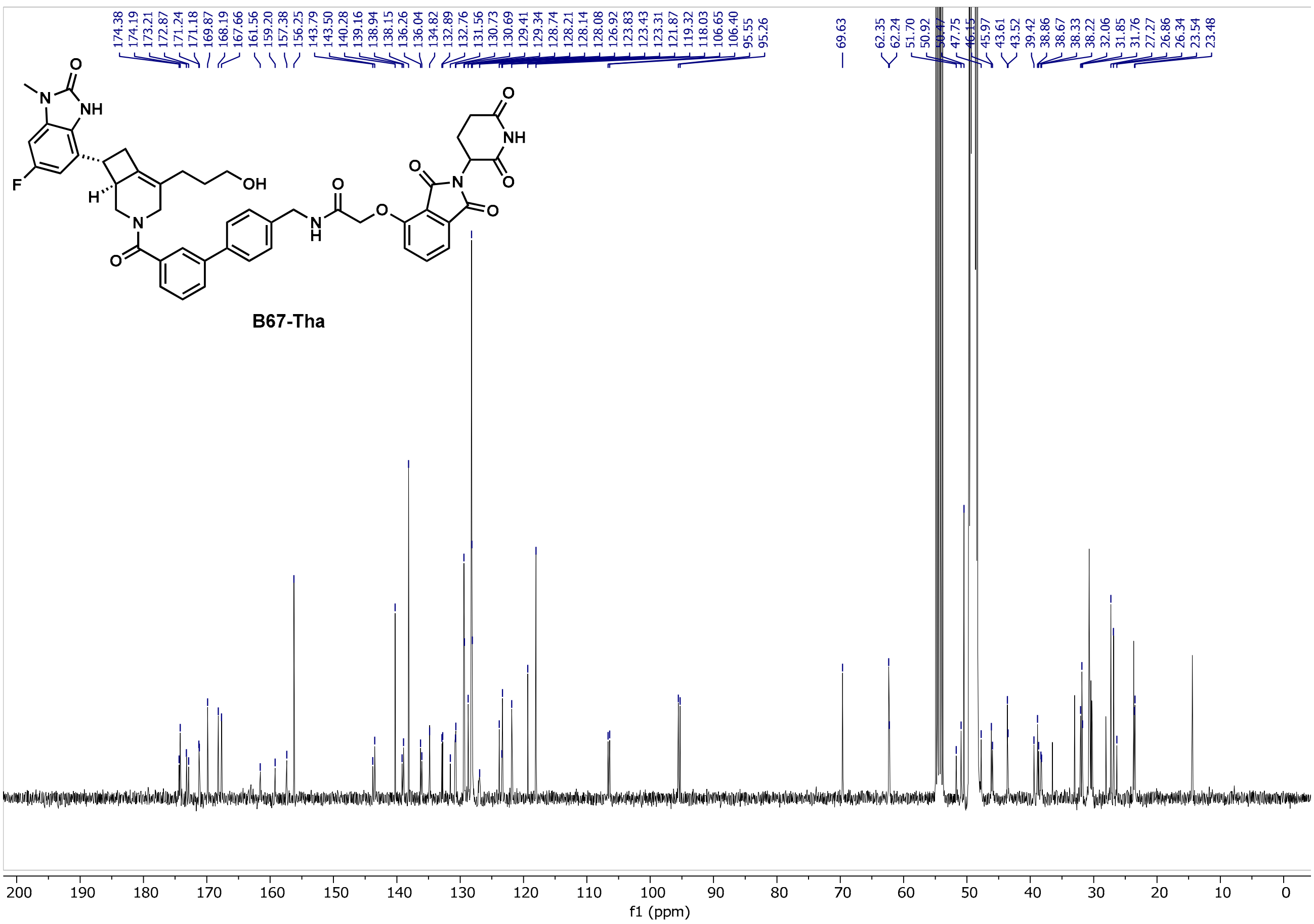
B67-Tha
f1 (ppm)

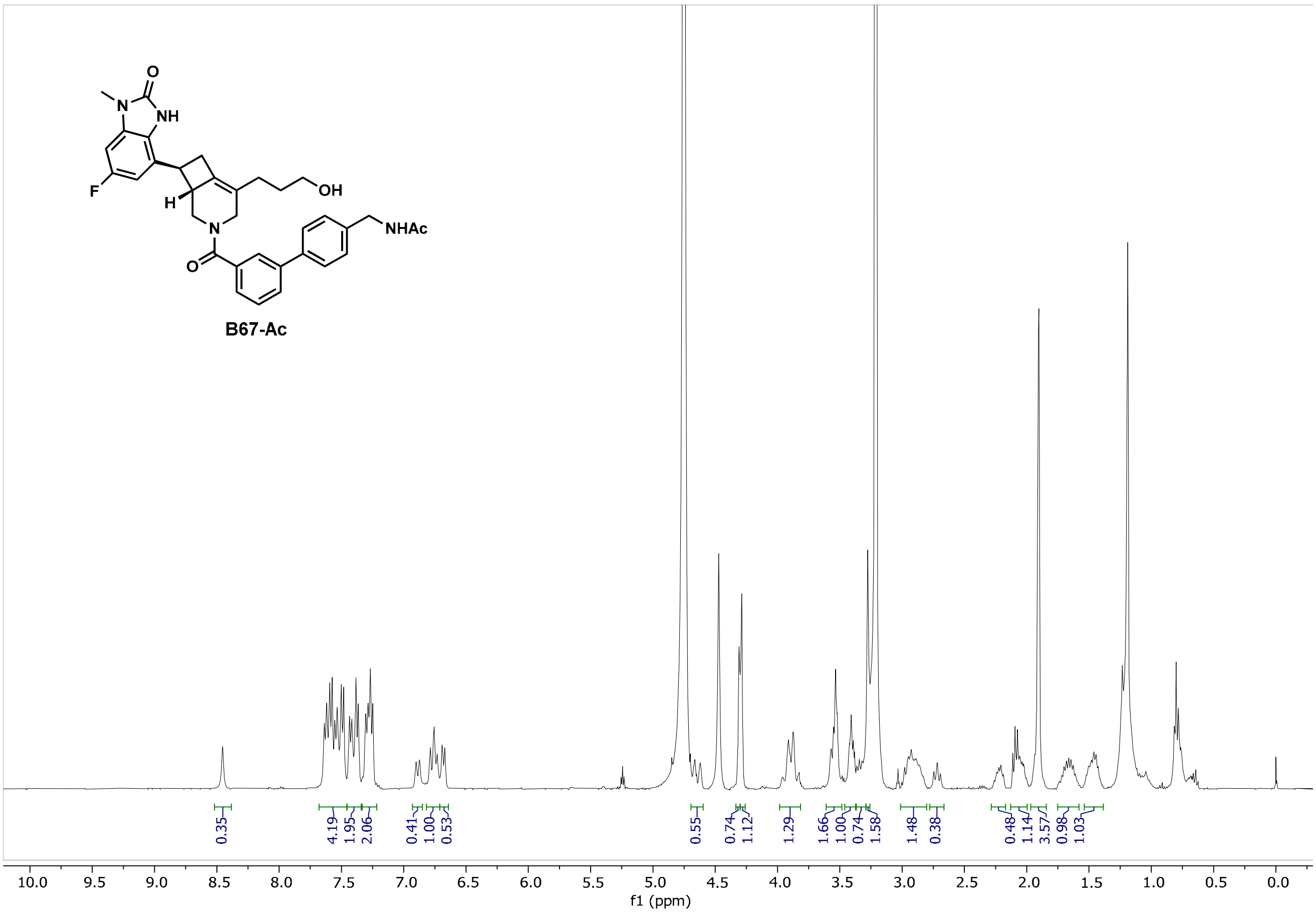

B67-Ac
f1 (ppm)

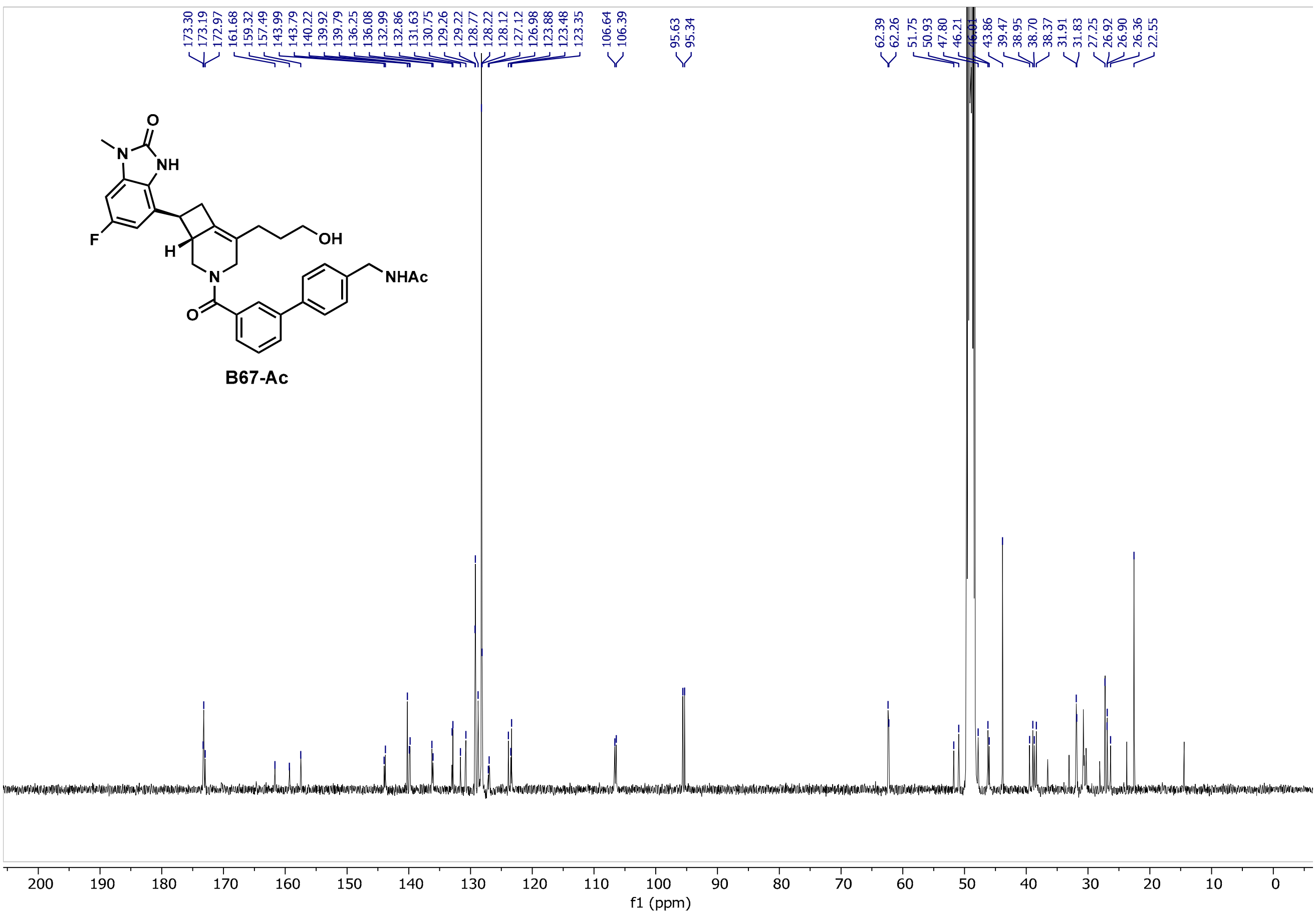

B67-Ac
f1 (ppm)

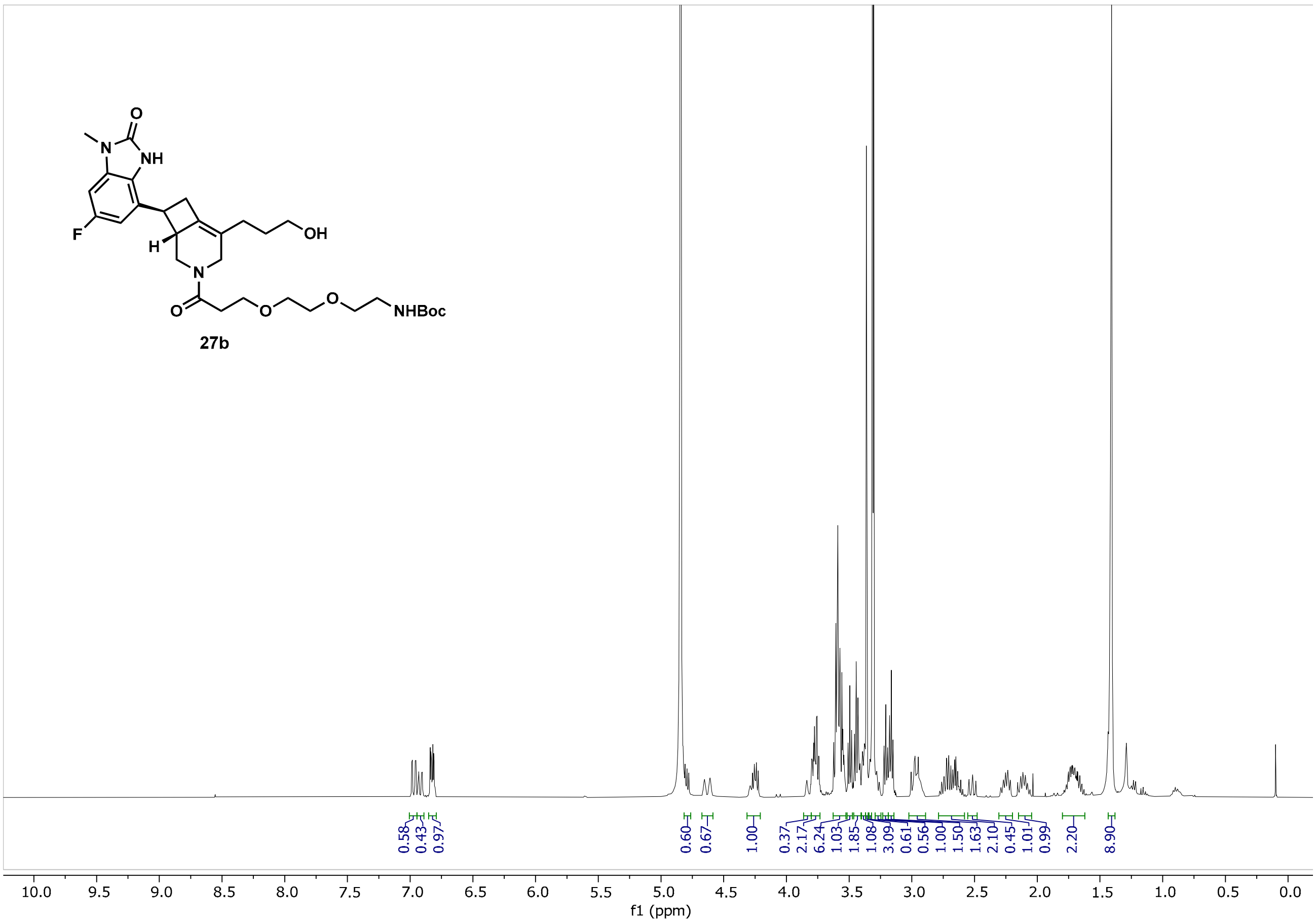
O
N
NH
F
H
OH
N
O
O
O
NHBoc
27b
0.58
0.43
0.97
0.60
0.67
1.00
0.37
2.17
6.24
1.03
1.85
1.08
3.09
0.61
0.56
1.00
1.50
1.63
2.10
0.45
1.01
0.99
2.20
8.90
10.0 9.5 9.0 8.5 8.0 7.5 7.0 6.5 6.0 5.5 5.0 4.5 4.0 3.5 3.0 2.5 2.0 1.5 1.0 0.5 0.0
f1 (ppm)

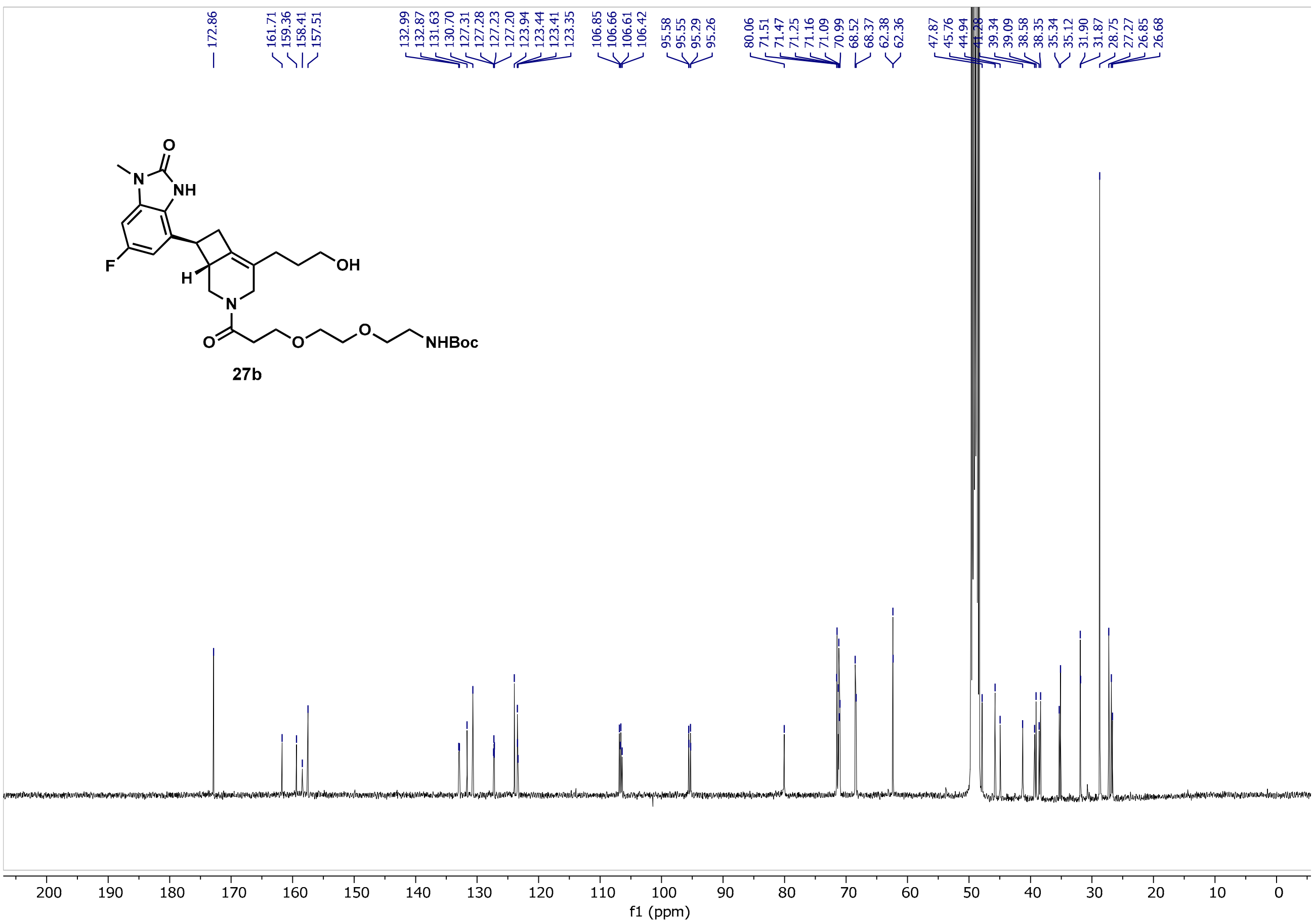
172.86
161.71
159.36
158.41
157.51
132.99
132.87
131.63
130.70
127.31
127.28
127.23
127.20
123.94
123.44
123.41
123.35
106.85
106.66
106.61
106.42
95.58
95.55
95.29
95.26
80.06
71.51
71.47
71.25
71.16
71.09
70.99
68.52
68.37
62.38
62.36
47.87
45.76
44.94
39.34
39.09
38.58
38.35
35.34
35.12
31.90
31.87
28.75
27.27
26.85
26.68
O
N
NH
F
H
OH
N
O
O
O
NHBoc
27b
200 190 180 170 160 150 140 130 120 110 100 90 80 70 60 50 40 30 20 10 0
f1 (ppm)

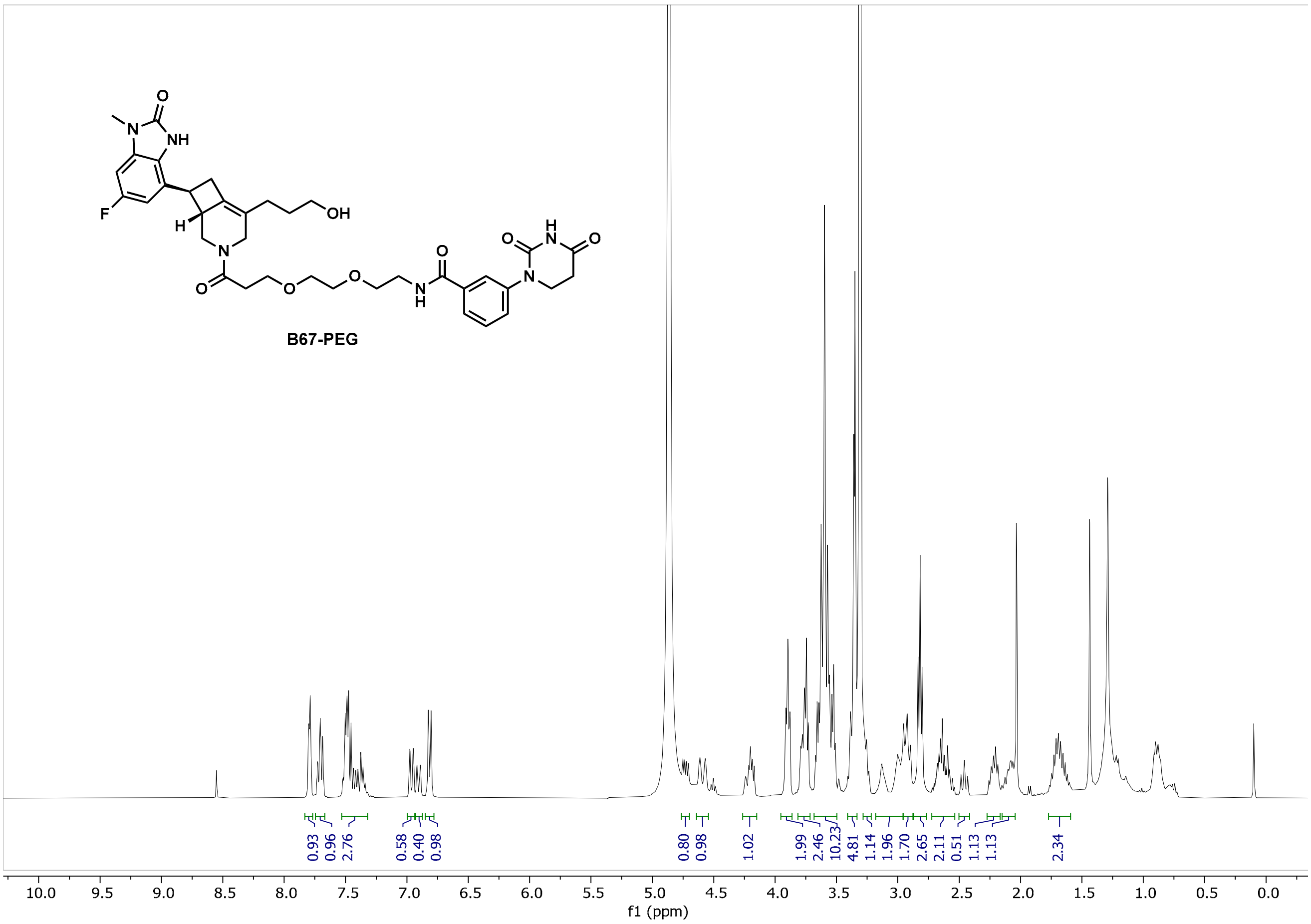
B67-PEG
0.93
0.96
2.76
0.58
0.40
0.98
0.80
0.98
1.02
1.99
2.46
10.23
4.81
1.14
1.96
1.70
2.65
2.11
0.51
1.13
1.13
2.34
10.0 9.5 9.0 8.5 8.0 7.5 7.0 6.5 6.0 5.5 5.0 4.5 4.0 3.5 3.0 2.5 2.0 1.5 1.0 0.5 0.0
f1 (ppm)

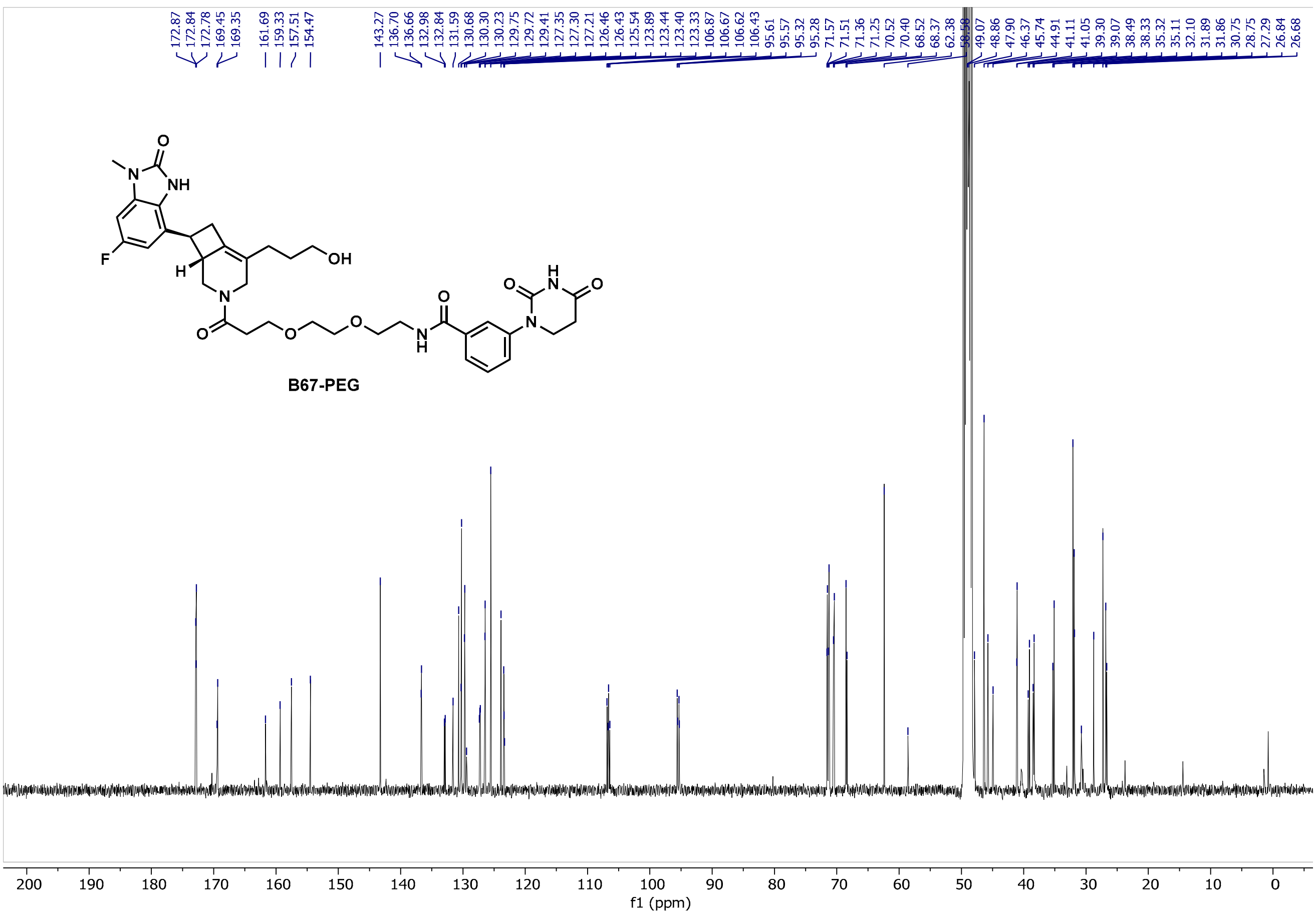
172.87
172.84
172.78
169.45
169.35
161.69
159.33
157.51
154.47
143.27
136.70
136.66
132.98
132.84
131.59
130.68
130.30
130.23
129.75
129.72
129.41
127.35
127.30
127.21
126.46
126.43
125.54
123.89
123.44
123.40
123.33
106.87
106.67
106.62
106.43
95.61
95.57
95.32
95.28
71.57
71.51
71.36
71.25
70.52
70.40
68.52
68.37
62.38
58.58
49.07
48.86
47.90
46.37
45.74
44.91
41.11
41.05
39.30
39.07
38.49
38.33
35.32
35.11
32.10
31.89
31.86
30.75
28.75
27.29
26.84
26.68
B67-PEG
200 190 180 170 160 150 140 130 120 110 100 90 80 70 60 50 40 30 20 10 0
f1 (ppm)
